# Catalytic and Photoluminescence Properties of the First‐ and Second‐Sphere Coordination of Lanthanide Complexes

**DOI:** 10.1002/chem.202502338

**Published:** 2025-10-08

**Authors:** Giau Le‐Hoang, Laure Guénée, Clémence Delage de Luget, Julien Chong, Claude Piguet

**Affiliations:** ^1^ Department of Inorganic and Analytic Chemistry University of Geneva 30 quai E. Ansermet Geneva CH‐1211 Switzerland; ^2^ Laboratory of Crystallography University of Geneva 24 quai E. Ansermet Geneva CH‐1211 Switzerland

**Keywords:** bifunctional catalyst, europium complexes, metal‐template, michael reaction, photoluminescence quantum yield

## Abstract

The efficient binding of receptors **L*k*** (**L*k*** = **L5**‐**L8**) to [Eu(hfac)_3_] (H‐hfac = 1,1,1,5,5,5‐hexafluoropentane‐2,4‐dione) allows (i) to gather the basic unit‐containing tridentate ligands **L*k*** and the acidic unit‐bearing bidentate hfac^−^ co‐ligands into a stable single molecular [**L*k*** Eu(hfac)_3_] adduct working as a catalyst for Michael C─C bond formations, (ii) to enhance the acidity of the N‐H units connected to the benzimidazole side arms of the bound ligands **L5** and **L7**, and (iii) to reorganize their geometries into *cis*‐*cis* conformation for activating Michael substrates **1** and **2** through H‐bonding interactions on the second‐sphere coordination to provide the target product **3** (up to 92% yield). A maximum enantiomeric excess of 21% could be achieved due to the long distance between the chiral sources and the catalytic sites in the europium complexes. Stepwise distortions from planarity of the terdentate **L*k*** ligands in the yttrium complexes [**L*k*** Y(hfac)_3_] (**L*k*** = **L2**, **L3**, and **L5**‐**L10**) can be tuned by addition of specific substituent into the benzimidazole side arms, thus realizing some control over their dual fluorescence and phosphorescence emission properties. All [**L*k*** Eu(hfac)_3_] (**L*k*** = **L2**, **L3**, and **L5**‐**L10**) complexes display europium‐centered photoluminescence properties induced by the antenna effect and modulated by the specific design of the light‐harvesting aromatic ligand located in the first coordination sphere.

## Graphical abstract

The Michael donor and the Michael acceptor are activated via hydrogen bonding interactions within the second‐sphere coordination of europium complexes.

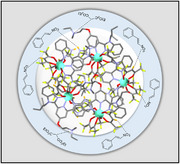

## 1 Introduction

**Scheme 1:**
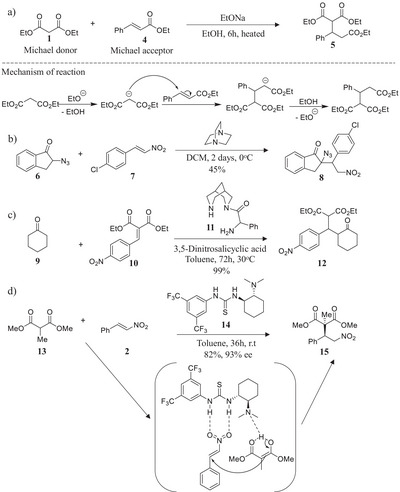
a) Michael reaction reported by Athur Michael in 1887, b) DABCO‐catalyzed reaction between **6** and **7**, c) addition of **9** to **10** in the coexistence of acid and base catalysts, d) asymmetric addition of **13** to **2** with the help of the chiral bifunctional catalyst **14**.

Among the carbon‐carbon bond‐forming techniques, the Michael reaction discovered by Arthur Michael in 1887, exhibits a recognizable role in providing critical precursors for constructing bioactive molecules and natural products in organic synthesis.^[^ ^1^, ^2^, ^3^, ^4^ ^]^ This transition‐metal‐free transformation is based on conjugate addition, or 1,4‐addition, of a carbon nucleophile (Michael donor) to an activated olefin (Michael acceptor) in the presence of a Brønsted base to form a new carbon‐carbon bond with perfect atom economy (Scheme 1a).^[^ ^1^, ^2^ ^]^ Moreover, it requires mild conditions and tolerates a wide range of functional groups which are molecularly attached to the Michael substrates.^[^ ^5^, ^6^ ^]^ However, the Michael reaction still represents certain difficulties, including competing reactions (such as polymerization of Michael acceptors, self‐aldolization of Michael donors, and 1,2‐addition reaction), poor reaction yields without an acid co‐catalyst as well as the lack of enantiocontrol of newly formed quaternary carbon center in the Michael product.^[^ ^7^, ^8^, ^9^, ^10^, ^11^, ^12^ ^]^

As illustrations: (i) the DABCO‐catalyzed addition of **6** to **7** gave the Michael adduct **8** in moderate 45% reaction yield due to the competing polymerization of nitroolefin **7** (Scheme 1b),^[^ ^10^ ^]^ (ii) the Michael reaction of cyclohexanone **9** to alkylidene malonate **10** in the presence of the base catalyst **11** afforded the desired product **12** in only 38% yield, while the catalytic combination of **11** and 3,5‐dinitrosalicyclic acid maximized the reaction yield up to 99% (Scheme 1c).^[^ ^12^ ^]^ A huge effort has been therefore focused on the design of chiral bifunctional organocatalysts such as proline derivatives, cinchona alkaloids, urea/thiourea‐containing tertiary amine, peptides for controlling chemical conversion as well as enantioselectivity in the asymmetric Michael addition.^[^ ^13^, ^14^, ^15^, ^16^, ^17^ ^]^ These chiral dual‐site organocatalysts carry two different functional groups for simultaneously activating both Michael donor and Michael acceptor substrates to improve reaction rate and stereocontrol of the chemical process.^[^ ^6^, ^18^, ^19^ ^]^ As an example, Takemoto and coworkers successfully positioned a tertiary amine and a thiourea unit on molecule **14** for catalyzing the Michael reaction between malonate **13** and nitroolefin **2** to generate compound **15** with excellent productivity and enantioselectivity (Scheme 1d).^[^ ^17^ ^]^ In this case, the tertiary amine unit in **14** promotes the enolization of malonate **13**, while the thiourea moiety in **14** activates nitroolefin **2** via hydrogen interactions; altogether accelerating the reaction rate as well as the chirality transfer from the catalyst to the formed compound **15**.

**Scheme 2:**
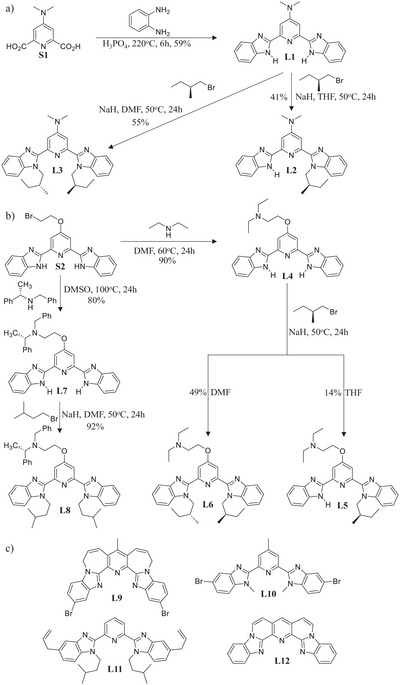
Synthesis of the target ligands a) **L1**‐**L3**, b) **L4‐L8**; and c) chemical structures of previously prepared ligands **L9**‐**L12** discussed in this contribution,.

The binding of a metal ion to organic ligands has been exploited for more than 40 years to gather and structurally reorganize the coordinated ligands into specific orientations required for their subsequent inter/intramolecular reactions.^[^ ^20^, ^21^, ^22^ ^]^ In this context, Sauvage and coworkers used Cu(I) to appropriately link two aromatic bidentate ligands for directing the synthesis of interlocked molecules.^[^ ^20^ ^]^ The groups of Gunnlaugsson and Leigh exploited the high coordination numbers (up to 12) in lanthanide complexes to increase the ligand density connected to Ln(III) center for promoting intercomponent reactions between the bound ligands.^[^ ^23^, ^24^ ^]^ Recently, our group reported the use of trivalent lanthanide ions to (i) benefit from the *trans*‐*trans* to *cis*‐*cis* conformation change of *free* to *bound* ligand for positioning the reactive end groups on the same side and accelerating the ring‐closing metathesis reaction of the bound ligand,^[^ ^25^ ^]^ and (ii) suppress the confinement of Rh/Ru catalysts into the nitrogen cavity of tridentate ligands and facilitate Rh/Ru‐based intermolecular couplings.^[^ ^26^, ^27^ ^]^ Trivalent europium complexes coordinating organic ligand chromophores have emerged as superior candidates in multifaceted fields such as light‐emitting diodes, biomedical probes, temperature sensors due to their pure red luminescence and highly selective sensing.^[^ ^28^, ^29^, ^30^, ^31^ ^]^ Ligand‐centered singlet excited state in europium complexes generated upon light absorption populates the ligand‐based spin‐forbidden triplet state, which then intramolecularly transfers its energy to the central europium ion for producing metal‐induced red emissions.^[^ ^32^ ^]^ This energy transfer process is therefore mainly governed by the antenna ligand structure. When the ligand‐based triplet state level is located close in energy to the receiving levels of trivalent europium ion, a competing back energy transfer from metal to bound ligand can occur, leading to a reduced sensitized photoluminescence quantum yield.^[^ ^33^, ^34^ ^]^ Adjusting this energy gap by ligand design to enhance luminescence properties of the europium complexes continues to attract the attention of organic chemists.^[^ ^35^, ^36^, ^37^ ^]^ Tridentate 2,6‐bis‐(benzimidazol‐2‐yl)pyridine derivatives (Scheme 2) and bidentate β‐diketonates are well‐known chelating sensitizer agents to efficiently induce photoluminescence of Eu(III) due to their strong coordination behaviors, their tunable molecular architecture (substituted, flexible, preorganized, rigid, extended conjugated, planar) as well as their attractive properties, such as thermal stability and volatility.^[^ ^38^, ^39^, ^40^ ^]^ In this contribution, we report the use of trivalent europium ion (i) to integrate the basic unit‐bearing tridentate ligand **L*k*** (Scheme 2) and the acidic unit‐containing bidentate hfac^−^ into a single molecule for developing new bifunctional catalysts and promoting the Michael reaction, (ii) to enhance the reactivity of functional group in the bound ligands and appropriately dispose the reactive groups into favorable directions required for their catalytic activity, and (iii) to reexamine the influence of aromatic ligand structures on the Eu‐centered photoluminescence quantum yields.

## 2 Results and Discussion

Synthesis and structural characterization of tridentate ligands **L** ***k*** (**L** ***k*** = **L1**‐**L8**) and their europium complexes [**L** ***k*** Eu(hfac)_3_].

**Figure 1:**
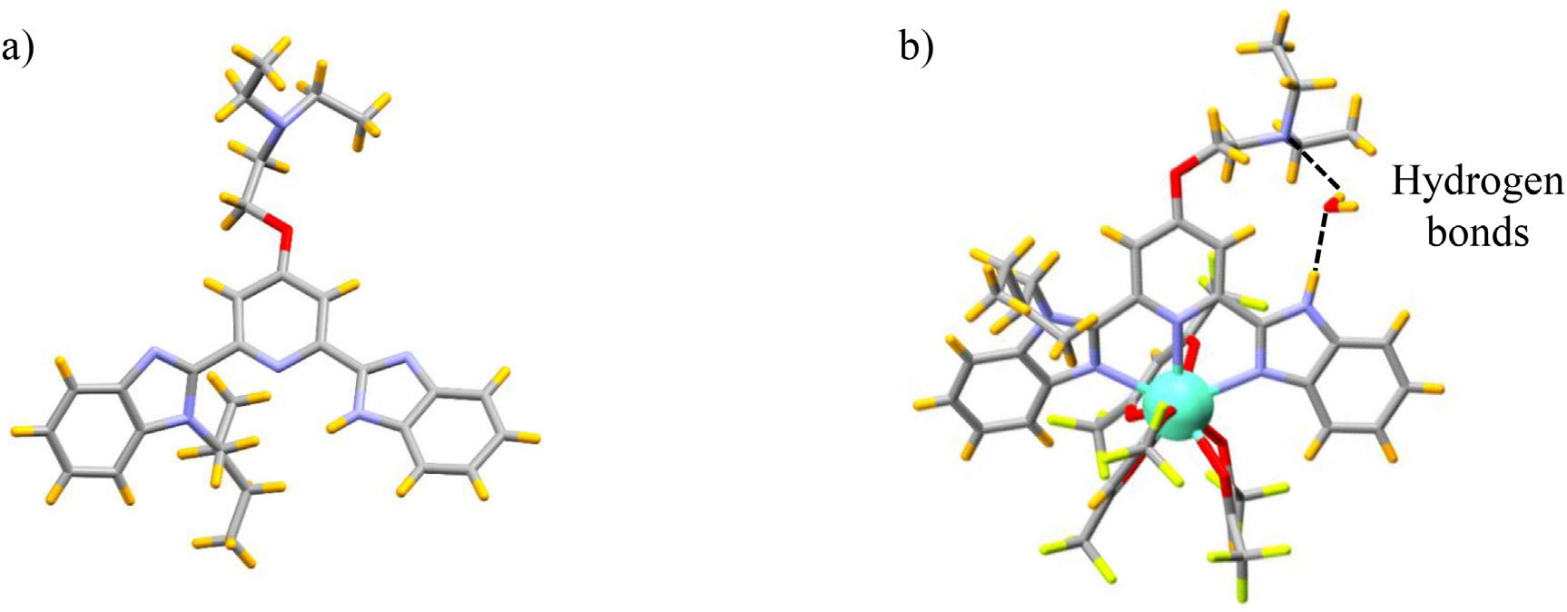
Crystal structures of a) one of the two independent molecules in the asymmetric unit of free **L5** ligand, and b) one of the four molecules in the asymmetric unit of [**L5**Eu(hfac)_3_]·H_2_O. Color codes: C = gray, N = blue, O = red, F = light yellow, Eu = green, H = orange.

**Figure 2:**
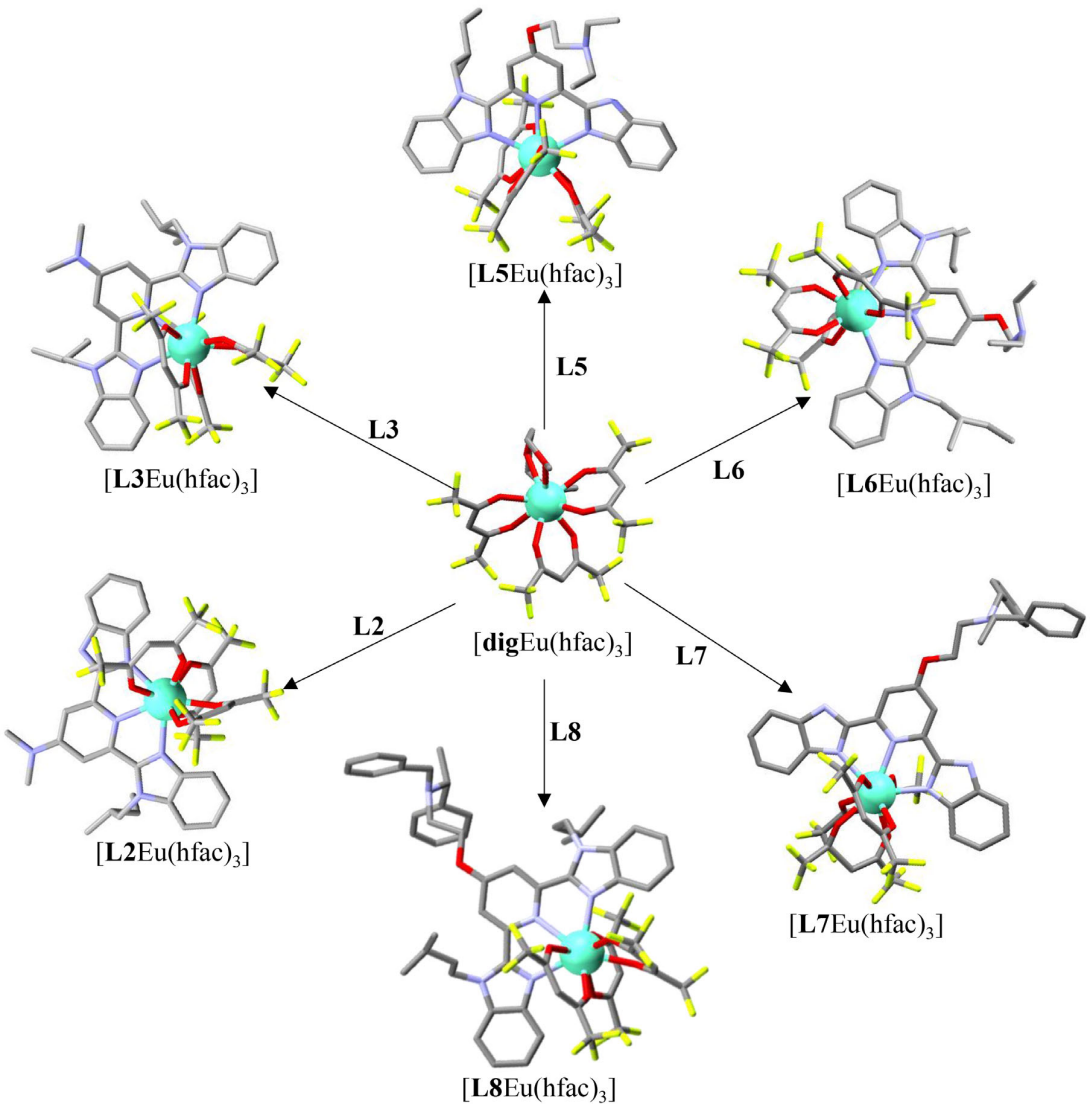
Synthesis and molecular structure of [**L*k*** Eu(hfac)_3_] (**L*k*** = **L2**, **L3**, and **L5**‐**L8**) complexes. Color codes: C = gray, N = blue, O = red, F = yellow, Eu = green. Hydrogen atoms have been omitted for clarity.

The synthesis of the tridentate building blocks **L1‐L8** (Scheme 2) is detailed in the Supporting Information. The synthetic strategy is based on (i) the Philips condensation reaction between a diamine and a dicarboxylic acid to build the 2,6‐bis‐(benzimidazol‐2‐yl)pyridine framework and (ii) substitution reactions at the benzimidazole side arms to control the number of remaining N‐H acid units. Two commercial compounds, (*S*)‐(+)1‐bromo‐2‐methylbutane and (*S*)‐(−)‐N‐benzyl‐α‐methylbenzylamine, were used as chiral sources to introduce the chirality into the target ligands. Slow evaporation of dichloromethane/hexane solutions gave single crystals of **L1**·CH_2_Cl_2_, **L2**·0.5CH_2_Cl_2_, **L3**, **L4**·CH_3_OH·2H_2_O, and **L5** suitable for X‐ray analysis (Appendix 2). Their solid‐state structures display *trans‐trans* conformations which (i) optimize inter‐site dipole interactions^[^ ^41^, ^42^ ^]^ and (ii) avoid steric repulsions between the N‐H/N‐alkyl groups attached to the benzimidazole side arms and the aromatic protons of the pyridine center (Figure 1a). The lack of NOESY correlation between the aromatic protons of the pyridine ring and those of the N‐substituent in CD_2_Cl_2_ confirms the maintain of similar *trans‐trans* organization in solution (Appendix 1). The crystal structures of ligands **L1** and **L4** display nearly planar geometries reflected in the minor dihedral angle (average of 5.22^0^) between the pyridine and the nonsubstituted benzimidazole side arms (Tables A2‐3 and A2‐14). Introduction of alkyl groups into the benzimidazole side arms in **L2**, **L3**, **L5** leads to a distortion visualized by increased pyridine‐benzimidazole interplanar angles ranging from 13.92(5)° to 44.65(12)° (Tables A2‐7, A2‐11, and A2‐18). The quality of the crystal structure of the amphoteric ligand **L5** is sufficient to unambiguously establish that the benzimidazole ring is protonated and the terminal trialkyl‐amine is deprotonated as illustrated in Figure 1a. This proton distribution is maintained in solution since both the associated NOESY spectrum (Figure A1‐28) and the HMBC spectrum (Figure A1‐30) point to the N(H) acid unit being localized within the benzimidazole side arm. The treatment of **L*k*** (**L*k*** = **L2**, **L3**, and **L5**‐**L8**) with [**dig**Eu(hfac)_3_] (**dig** = diglyme or bis(2‐methoxyethyl)) results in the formation of the target complexes [**L*k*** Eu(hfac)_3_], as suggested by the downfield shift of the aromatic protons located close to the paramagnetic europium(III) center (Figure 6a,b), which can be isolated in 66–92% yields. Recrystallization by slow evaporation of dichloromethane/hexane solutions affords single crystals of [**L2**Eu(hfac)_3_]·0.25CH_2_Cl_2_·0.5C_6_H_14_, [**L3**Eu(hfac)_3_], [**L5**Eu(hfac)_3_]·H_2_O, [**L6**Eu(hfac)_3_], [**L7**Eu(hfac)_3_], and [**L8**Eu(hfac)_3_]·0.25CH_2_Cl_2_ suitable for X‐ray analysis (Figure 2 and Appendix 2). All crystal structures exhibit nine‐coordinate complexes, in which the Eu(III) center is encompassed by three nitrogen atoms of the bound aromatic ligand **L*k*** (**L*k*** = **L2**, **L3**, and **L5**‐**L8**) and by six oxygen atoms from three bidentate hfac^−^ co‐ligands. The coordinated aromatic ligands adopt the *cis‐cis* arrangement required for chelate coordination, which is evidenced by their molecular structures observed in the solid state. In CD_2_Cl_2_ solution, related cis‐cis arrangements are ascertained by the detection of NOESY correlations between the proton of N‐alkyl groups connected to the lateral benzimidazole rings and the protons of the central pyridine unit. The crystal structure of [**L5**Eu(hfac)_3_]·H_2_O is worth being highlighted because one guest water molecule is doubly hydrogen bound to (i) the basic exocyclic nitrogen atom of the bound ligand **L5** and (ii) the acidic (N)H of the benzimidazole side arm (Figure 1b). This host‐guest interaction suggests a potential substrate accommodation in this cavity and some acid‐base bifunctional catalytic ability of the bound aromatic ligand **L5** toward the Michael reaction. The replacement of the hydrogen atoms connected to the nitrogen atoms of the benzimidazole side arms with alkyl substituents induces steric repulsions with the hydrogen atoms of the central pyridine ring, which results in twists of the benzimidazole‐pyridine units (average dihedral angle = 3(3)° for (HN)benzimidazole‐pyridine and 23(8)° for (alkyl‐N)benzimidazole‐pyridine) in [**L*k*** Eu(hfac)_3_] (**L*k*** = **L2**, **L3**, **L5**, **L6**, **L8**) complexes (Tables A2‐22, A2‐25, A2‐28, A2‐32, A2‐42). The latter intramolecular interaction is lacking in [**L7**Eu(hfac)_3_] complex, and a nearly planar structure is found for the bound ligand **L7** (dihedral angle = 2.8(2)°, Table A2‐35). Finally, the chirality of each synthesized compound was confirmed by measuring its specific rotation in CH_2_Cl_2_ (Table A2‐43). The nonzero specific rotations indicate that all final compounds are optically active in solution (Table A2‐43).

### 2.1 Thermodynamic Behavior of L7 and L8 Hosts With [Eu(hfac)_3_] Guest in Dichloromethane Solution

The stability constants of [**L7**Eu(hfac)_3_] and [**L8**Eu(hfac)_3_] complexes were determined by ^1^H NMR titrations of the free ligands **L7** and **L8** with [**dig**Eu(hfac)_3_] in CD_2_Cl_2_ solution according to Eq. 1.

(1) $$\left[\mathrm{dig}\mathrm{Eu}{\left(\mathrm{hfac}\right)}_{3}\right]+Lk↔\left[Lk\mathrm{Eu}{\left(\mathrm{hfac}\right)}_{3}\right]+\mathrm{dig}\beta_{1,1,\mathrm{exch}}^{Lk,\mathrm{Eu}}$$

Using an excess of **dig** (0.14 M) to stabilize the activity coefficients of chemical substances at millimolar concentrations upon the ^1^H NMR titration,^[^ ^43^ ^]^ the ligand exchange constant $\beta_{1,1,\mathrm{exch}}^{Lk,\;\mathrm{Eu}}$ described in eq. 1 transforms into the conditional binding constant $\beta_{1,1,\mathrm{cond}}^{Lk,\;\mathrm{Eu}}\;$ shown in Eq. 2.

(2) $$\begin{array}{ccc} & & \left[\mathrm{Eu}{\left(\mathrm{hfac}\right)}_{3}\right]+Lk↔\left[Lk\mathrm{Eu}{\left(\mathrm{hfac}\right)}_{3}\right]\beta_{1,1,\mathrm{cond}}^{Lk,\mathrm{Eu}}\\ & & \;\text{With}\;\beta_{1,1,\mathrm{cond}}^{Lk,\mathrm{Eu}}=\frac{\;\beta_{1,1,\mathrm{exch}}^{Lk,\mathrm{Eu}}}{{\left|\mathrm{dig}\right|}_{\mathrm{tot}}}=\frac{\left|Lk\mathrm{Eu}\right|}{\left|\mathrm{Eu}||Lk\right|} \end{array}$$

**Figure 3:**
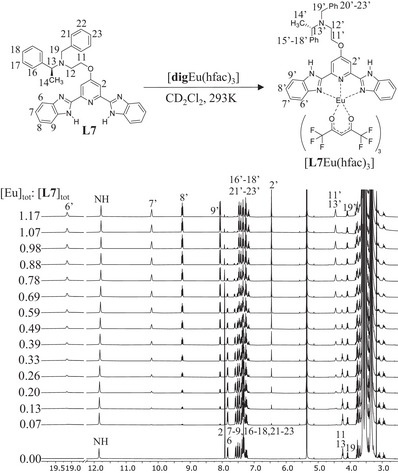
^1^H NMR titration of **L7** with [**dig**Eu(hfac)_3_] in CD_2_Cl_2_ + 0.14 M **dig** at 293 K.

**Figure 4:**
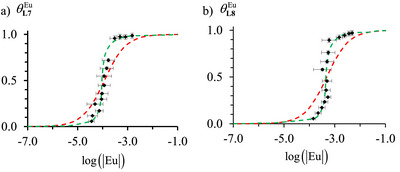
Experimental (black diamonds) and fitted binding isotherms using eq. 3 (dashed‐red trace) and eq. A3‐3 (dashed‐green trace) for the titrations of a) **L7**, and b) **L8** with [**dig**Eu(hfac)_3_] in CD_2_Cl_2_ + 0.14 M **dig** at 293 K.

Experimentally, at each point of the host‐guest titration, the recorded ^1^H NMR spectrum (Figure 3) affords reliable integrations for the same proton corresponding to the coordinated $\left(I_{Lk\mathrm{Eu}}\right)$ and to the free $\left(I_{Lk}\right)$ ligands, from which the associated occupancy factor $\theta_{Lk}^{\mathrm{Eu}}$ (Eq. 3) and the free concentration $\left|\mathrm{Eu}\right|={\left|\mathrm{Eu}\right|}_{\mathrm{tot}}-\theta_{Lk}^{\mathrm{Eu}}{\left|Lk\right|}_{\mathrm{tot}}$ of the trivalent lanthanide guest in solution can be calculated for building the experimental binding isotherms (black diamonds in Figure 4).

(3) $$\begin{array}{ccc} \theta_{Lk}^{\mathrm{Eu}} & = & \frac{{\left|\mathrm{Eu}\right|}_{\mathrm{bound}}}{{\left|Lk\right|}_{\mathrm{tot}}}=\;\frac{\left|Lk\mathrm{Eu}\right|}{{\left|Lk\right|}_{\mathrm{tot}}}=\frac{I_{Lk\mathrm{Eu}}}{I_{Lk\mathrm{Eu}}+I_{Lk}\;}\\ & = & \frac{{\left|\mathrm{Eu}\right|}_{\mathrm{tot}}-\;\left|\mathrm{Eu}\right|}{{\left|Lk\right|}_{\mathrm{tot}}}=\frac{\beta_{1,1,\;\mathrm{cond}}^{Lk,\;\mathrm{Eu}}\left|\mathrm{Eu}\right|}{1+\beta_{1,1,\;\mathrm{cond}}^{Lk,\;\mathrm{Eu}}\left|\mathrm{Eu}\right|} \end{array}$$

The conditional stability constants $\beta_{1,1,\mathrm{cond}}^{Lk,\;\mathrm{Eu}}$ can be estimated for [**L7**Eu(hfac)_3_] and [**L8**Eu(hfac)_3_] complexes by nonlinear least‐squares fits of $\theta_{Lk}^{\mathrm{Eu}}$ versus |Eu| (red trace in Figure 4) with the help of eq. 3 (second line). They are collected in Table 1.

**Table 1:** Thermodynamic conditional and exchange stability constants determined for the titration of **L7** and **L8** ligands with [**dig**Eu(hfac)_3_] in CD_2_Cl_2_ + 0.14 M **dig** at 293 K.

	[L7Eu(hfac)_3_]	[L8Eu(hfac)_3_]
$\beta_{1,1,\mathrm{cond}}^{Lk,\;\mathrm{Eu}}$	9700(400)	2100(200)
$\beta_{1,1,\mathrm{exch}}^{Lk,\;\mathrm{Eu}}$	1358(60)	290(24)

The removal of steric congestion between the hydrogen atoms of the central pyridine and the isopentyl groups bound to the benzimidazole side arms in going from [**L8**Eu(hfac)_3_] to [**L7**Eu(hfac)_3_] results in a significant increase (∼5x) of the association constant (Table 1). The synthesized europium complexes are used as catalysts for Michael reactions conducted at 0.025 M (see next section) in dry dichloromethane solution. Based on the stability constants $\beta_{1,1,\mathrm{exch}}^{Lk,\;\mathrm{Eu}}$ collected in Table 1, one can estimate that 99.9% of the target heteroleptic europium complexes [**L*k*** Eu(hfac)_3_] are present in solution at this concentration during the Michael reactions.

### 2.2 Catalytic Activity of Ligands Lk and Their Europium Complexes

The Michael reaction between conjugated β‐nitrostyrene **2** (1eq.) and diethyl malonate **1** (2 eq.) has been selected to evaluate the catalytic behavior of the free ligands **L*k*** and their europium complexes (Table 2).

**Table 2:** Results of Michael reactions between malonate **1** and nitrostyrene **2**

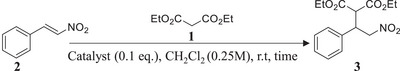				
Entry	Catalyst	Time /h	Yield /%^[^ ^a^ ^]^	ee /%^[^ ^b^ ^]^
1	DMAP	24	9	ND^[^ ^c^ ^]^
2	[**dig**Eu(hfac)_3_]	168	NR^[^ ^d^ ^]^	‐
3	Et_3_N	24	23	ND
4	**L1** ^[^ ^e^ ^]^	168	NR	‐
5	**L2**	168	NR	‐
6	**L3**	168	NR	‐
7	**L4**	168	NR	‐
8	**L5**	168	NR	‐
9	**L6**	168	NR	‐
10	**L7**	336	NR	‐
11	**L8**	336	NR	‐
12	[**L2**Eu(hfac)_3_]	168	NR	‐
13	[**L3**Eu(hfac)_3_]	168	NR	‐
14	[**L5**Eu(hfac)_3_]	24	55 (57)^[^ ^f^ ^]^	7
15	[**L5**Eu(hfac)_3_]	72	92 (92)	9
16	[**L6**Eu(hfac)_3_]	24	38 (42)	0
17	[**L6**Eu(hfac)_3_]	72	72 (83)	3
18	**L6**+[**dig**Eu(hfac)_3_]	72	47^[^ ^f^ ^]^	ND
19	**L6**+[Eu(tfc)_3_]	72	NR	‐
20	[**L7**Eu(hfac)_3_]^[^ ^g^ ^]^	336	25 (28)	21
21	[**L8**Eu(hfac)_3_]^[^ ^g^ ^]^	336	18^[^ ^h^ ^]^ (12)	20
22	[**L11**Eu(hfac)_3_]	168	NR	‐

^[a]^ Isolated yield.

^[b]^ Enantiomeric excess obtained by chiral HPLC (Figure A1‐140).

^[c]^ Not determined (ND).

^[d]^ No reaction (NR) (Figures A1‐130, A1‐131, A1‐132, and A1‐139).

^[e]^ Reaction conducted in CH_2_Cl_2_/CH_3_OH (1/1).

^[f]^ Determined from the ^1^H NMR spectrum of reaction mixture (Figures A1‐131 and A1‐132).

^[g]^ The reaction was conducted with 5 equivalents of compound **1**.

^[h]^ A small amount of impurity is present in the isolated product.

The reaction is first conducted at room temperature in dry dichloromethane solution (0.25 M) for 24 hours by using 4‐dimethylamino‐pyridine (DMAP) as Bronsted base catalyst (entry 1) at a fixed loading of 10 mol % to generate the desired product **3** in only 9% yield. This reaction is not completed within 24 hours, visualized by the signals of the unreacted starting Michael acceptor **2** in the ^1^H NMR spectrum of the reaction mixture shown in Figure A1‐130c. However, the DMAP‐bearing ligands **L1‐L3** display no catalytic activity in the discussed reaction despite the reaction time being extended for 168 hours (entries 4–6). Their catalytic deficiencies could be accounted for by the reduced basicity of amine‐nitrogen atoms implied in the π‐delocalization between the central DMAP unit and the lateral benzimidazole rings. Replacement of the aromatic nitrogenous base DMAP with the stronger aliphatic base Et_3_N results in a slightly higher 23% yield (entry 3), together with the detection of an insoluble solid side‐product resulting from the polymerization of β‐nitrostyrene (Figure A1‐129). The polymeric by‐product exhibits an extremely poor solubility in organic solvent, implying difficulties in structural characterization of polymer product in solution by spectroscopies. To clarify the formation of the side product, two additional reactions were carried out: one between compounds **2** and **1**, and another between compound **2** and Et_3_N. After 24 hours, a formed solid was only observed in the mixture of nitrostyrene **2** and Et_3_N (Figure A1‐129). The ^1^H NMR spectrum of this precipitate resembled that obtained from the Michael reaction catalyzed by Et_3_N (Figure A1‐134), indicating that the insoluble solid results from the polymerization of monomeric precursor **2**.^[^ ^11^, ^44^ ^]^ Unexpectedly, all Et_3_N‐containing ligands **L4‐L6** do not show any catalytic behavior for both the Michael reaction and for polymerization despite the presence of complementary acid N‐H groups borne by the benzimidazole side arms in **L4** and **L5** (entries 7–9). The failure of the Michael reaction could be explained by (i) the reduced reactivity of tertiary amine moiety caused by the negative inductive effect of oxygen atom attached to the pyridine ring, and (ii) the weak hydrogen‐binding ability of acid N‐H units belonging to the benzimidazole side arm.

**Figure 5:**
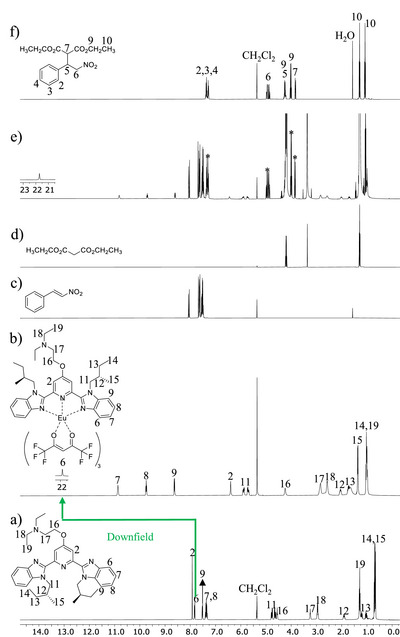
^1^H NMR spectra of a) ligand **L6**, b) complex [**L6**Eu(hfac)_3_], c) *β*‐nitrostyrene **2**, d) diethyl malonate **1**, e) reaction mixture from entry 16 (*: signals of formed compound), and f) desired product **3** in CD_2_Cl_2_.

**Figure 6:**
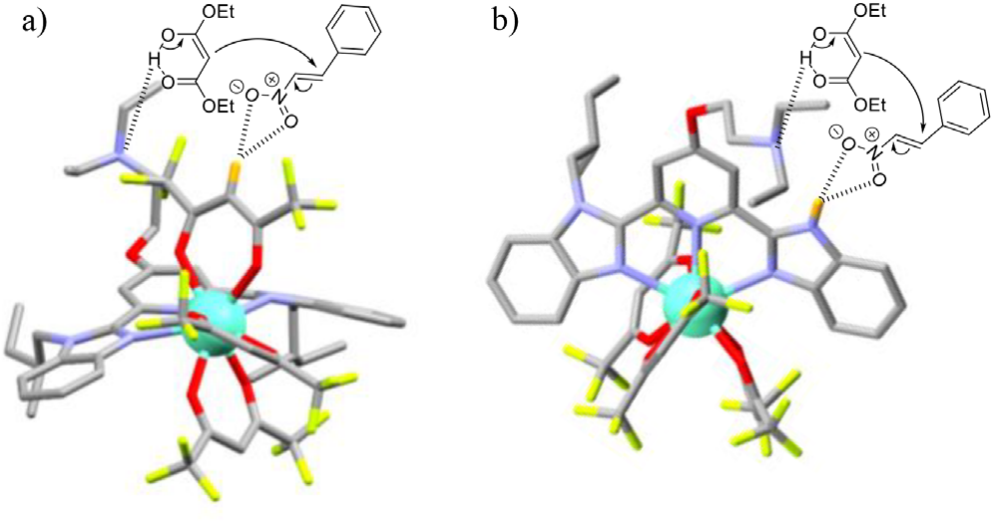
Activations of the Michael substrates by the aliphatic tertiary amine group and a) the acid unit C‐H of hfac^−^ in [**L6**Eu(hfac)_3_], and b) the acidic N‐H group of the benzimidazole side arm in [**L5**Eu(hfac)_3_] complex.

Next, we evaluate the catalytic capability of the europium complexes. When [**L6**Eu(hfac)_3_] complex is employed as the catalyst, the Michael product **3** is formed in 38% yield after 24 hours (entry 16) and in 72% after 72 hours (entry 17). The formation of the desired compound is visualized by the appearance of new signals marked with an asterisk in the ^1^H NMR spectrum of reaction mixture (Figure 5). The [**L6**Eu(hfac)_3_] catalyst remains stable as confirmed by its characteristic signals in the ^1^H and ^19^F NMR spectra of the reaction mixtures (Figures 5e, A1‐132c, A1‐133e, and A1‐133f). The DOSY spectrum of reaction mixture recorded after 72 hours (Figure A1‐137) separates the NMR signals into three components according to their molecular translational diffusion coefficients: the Michael substrates, the Michael product, and the catalyst, further confirming the absence of catalyst decomposition during the 1,4‐addition process. Moreover, the catalyst [**L6**Eu(hfac)_3_] is recovered in 62% yield from the Michael reaction (entry 17) by size exclusion chromatography thanks to the significant difference in molecular weights between the catalyst and the Michael substrates/product. In the reaction mechanism, the aliphatic tertiary amine group of the aromatic ligand assists the enolization of the Michael donor **1**, while the two oxygen atoms in NO_2_ group of the Michael acceptor **2** noncovalently interact with the acidic proton (C)‐H of the coordinated hfac‐ through hydrogen bonding connection Figure 6a).

The target Michael reaction attempted in the absence of exocyclic amine‐nitrogen atom in [**L11**Eu(hfac)_3_] complex (entry 22, Figure A1‐139) displays no conversion, proving the important role of basic moiety attached to the pyridine ring. The combination of **L6** and [**dig**Eu(hfac)_3_] is also found to drive the discussed reaction to the Michael product **3** in 47% yield after 72 hours (entry 18). The ^1^H NMR spectrum of the latter reaction mixture (Figure A1‐132d) shows the characteristic signals of [**L6**Eu(hfac)_3_] and **dig**, revealing the reaction of **L6** with [**dig**Eu(hfac)_3_] for generating [**L6**Eu(hfac)_3_] which subsequently catalyzes the Michael reaction. The combination of **L6** and commercially available europium tris[3‐(trifluoromethylhydroxymethylene)‐(+)‐camphorate [Eu(tfc)_3_] displays no catalytic activity in the Michael reaction (entry 19, Figure A1‐132e), denoting the role of acidic proton (C)‐H of the coordinated hfac‐ in [Eu(hfac)_3_] containers. Interestingly, [**L5**Eu(hfac)_3_] shows an improved catalytic activity reflected in higher reaction yields after 24 hours (entry 14) and 72 hours (entry 15) as compared to those promoted by [**L6**Eu(hfac)_3_] complex (entries 16 and 17). The N‐H group connected to the benzimidazole side arm in the bound aromatic ligand available in [**L5**Eu(hfac)_3_] catalyst becomes more acidic upon complexation to the central metal. It can capture the electrophilic NO_2_ group of compound **2** and therefore enhance the Michael reaction performance (Figure 6b). In this case, Eu(III) plays a considerable role in (i) facilitating the assembly of two base and acid components into a single scaffold, (ii) enhancing the reactivity of the functional group N‐H for catching electrophilic reagents, and (iii) reorganizing the bound ligand **L5** into the *cis‐cis* conformation required for the catalytic process. However, the level of chirality transfer from these europium‐based scaffolds to the Michael product **3** is disappointing due to the long distance between the chiral carbon center in [**L*k*** Eu(hfac)_3_] complexes (**L*k*** = **L5** and **L6**) and the hydrogen‐binding sites.

Recrystallization by slow evaporation of dichloromethane/hexane solution of compound **3** from entry 17 affords its single crystal suitable for X‐ray analysis (Figure A2‐14). Compound **3** displays a reversible phase transition upon cooling during X‐ray diffraction analysis, causing crystal lattice rearrangement and consequently slightly damaged crystals at low temperature 120 K (Figures A2‐15 and A2‐17). Its crystal structure can be solved at all temperatures (180 K, 150 K and 120 K) and exhibits a racemic arrangement (Figure A2‐16). Positioning the chiral center closer to the basic amine nitrogen atom in the bound tridentate ligands **L7** and **L8** leads to slightly improved chirality transfer from [**L*k*** Eu(hfac)_3_] complexes to the Michael product **3** with 21% ee. However, the reaction yields are dramatically reduced due to the steric hindrance induced by the bulky substituents close to the active nitrogen and should be improved for practical applications.

### 2.3 Photophysical Properties of Free Ligands and Their Lanthanide [L*k*Ln(hfac)_3_] Complexes

**Figure 7. a):**
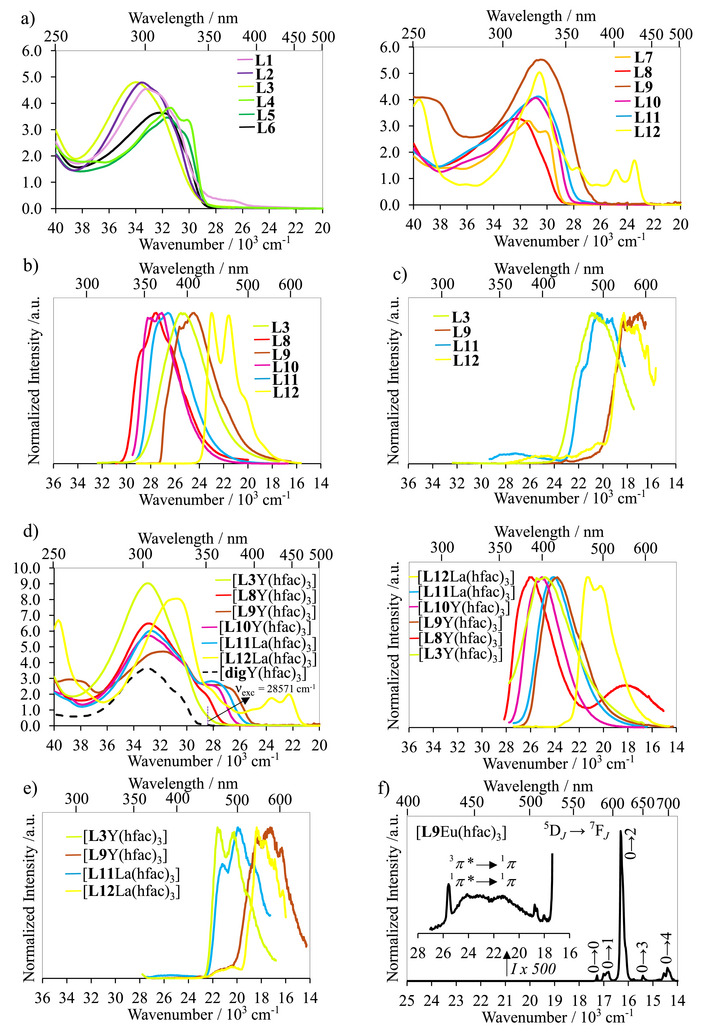
Absorption spectra of ligands **L1**‐**L12**. b) Normalized 293K‐emission spectra of **L3** (λ_exc_ = 310 nm), **L8** (λ_exc_ = 326 nm), **L9** (λ_exc_ = 435 nm), **L10** (λ_exc_ = 429 nm), **L11**, and **L12**. c) Normalized 77K‐phosphorescence spectra of **L3** (λ_exc_ = 310 nm), **L9** (λ_exc_ = 435 nm), **L11**, and **L12**.^[^ ^27^ ^]^ d) 293K‐Absorption (left) and normalized emission (right, λ_exc_ = 350 nm) spectra of [**L*k*** Y(hfac)_3_] (**L*k*** = **L3**, **L8**‐**L10**), and [**L*k*** La(hfac)_3_] (**L*k*** = **L11**, **L12**)^[^ ^27^ ^]^ complexes. e) Normalized 77k‐phosphorescence spectra (λ_exc_ = 350 nm) of [**L*k*** Y(hfac)_3_] (**L*k*** = **L3**, **L9**), and [**L*k*** La(hfac)_3_] (**L*k*** = **L11**, **L12**)^[^ ^27^ ^]^ complexes. f) Normalized 293K‐emission spectrum (λ_exc_ = 350 nm) of [**L9**Eu(hfac)_3_] complex.

The UV‐Vis absorption spectra of **L1‐L8** recorded in CH_2_Cl_2_ solution at room temperature are dominated by the broad bands in the region of 32 260–32 790 cm^−1^ assigned to the spin‐allowed ^1^π_1_*←^1^π transition (Figure 7a). The gradual introduction of substituent at the nitrogen atom of the benzimidazole side arms leads to a somewhat blue‐shift of the lowest‐energy ^1^π_1_*←^1^π transition along the **L1**→**L2**→**L3**, **L4**→**L5**→**L6**, and **L7**→**L8** series due to the less efficient electronic connection between the central pyridine and the lateral benzimidazole rings caused by the distortion in the ligand scaffold. A blue‐shift in the ^1^π_1_*←^1^π transition is also observed upon connecting an electron donating heteroatoms (O and N) to the 4‐position of the pyridine center along the series **L11** (30 675 cm^−1^) → **L8** (32 362 cm^−1^) ≈ **L6** (32 154 cm^−1^) → **L3** (34 014 cm^−1^). The room temperature emission spectra of ligands **L1**‐**L10** are acquired under excitation at absorption maxima of each antenna ligand (Figures 7b and A4‐3), from which the observed ligand‐based singlet excited ^1^π_1_*→^1^π transitions are determined and collected in Table A4‐1. Despite differences in structural distortion in the ground state, identical emission spectra are observed along the **L1**→**L2**→**L3**, **L4**→**L5**→**L6**, and **L7**→**L8** series (Figure A4‐3). Interestingly, in frozen dichloromethane solution at 77 K, all heteroatom‐(Br, N, O)‐substituent‐bearing **L1**‐**L10** ligands display both ^1^π_1_*→^1^π fluorescence and ^3^π_1_*→^1^π phosphorescence emissions (Figure A4‐3), while the peripheral heteroatom‐free **L11** and **L12** models exhibit only fluorescence bands. This phenomenon could be explained by the presence of the heavy‐atom Br and the lone‐pair electrons in (N, O) atoms, which allow the spin‐forbidden singlet‐triplet interaction to proceed more efficiently by increasing spin‐orbit coupling in agreement with previous studies.^[^ ^45^, ^46^, ^47^ ^]^ Moreover, at cryogenic temperature, nonradiative processes (intermolecular motion, vibrational relaxation, solvent quenching) are significantly reduced, allowing the detection of long‐lived phosphorescence emission. The intensity of phosphorescence emission at low temperature is enhanced along the **L3**→**L2**→**L1**, **L6**→**L5**→**L4**, and **L8**→**L7** series while increasing planarity degree in the ligand frameworks. A planar ligand backbone can facilitate overlap of π‐orbitals upon singlet‐triplet interactions, improving intersystem crossing efficiency and triplet state population.^[^ ^48^, ^49^ ^]^ The close‐to‐planar ligands **L1**, **L4**, and **L7** display exclusively phosphorescence signal at low temperature, while emission profiles of their *di*‐alkylated distorted **L3**, **L6**, and **L8** analogues are dominated by fluorescence bands under similar condition. Control over the emission properties from phosphorescence to fluorescence in **L*k*** (**L*k*** = **L1**‐**L8**) emitters is realized by stepwise introduction of substituent onto the benzimidazole side arms for tuning the planarity of the polyaromatic ligands. Remarkably, the preorganized ligand **L9** displays a phosphorescence band at much lower energy compared to its halogen‐bearing **L10** model and its halogen‐free **L*k*** (**L*k*** = **L1**‐**L8** and **L11**) analogues. The crystal structure of **L9** shows a distortion of the vinylene unit from the pyridine plane due to the H···H repulsion between the methyl group bound to the pyridine ring and the vinylene unit of CH_2_‐CH = CH bridge.^[^ ^25^ ^]^ The planarization of the distorted backbone **L9** upon photoexcitation leads to the electronic communication between the benzimidazole‐pyridine system and the double bond of the CH = CH‐CH_2_ fragments, enhancing the triplet state stability and lowering its energy level.^[^ ^50^, ^51^, ^52^ ^]^ The distorted (10π+2π+6π+2π+10π = 30π)‐containing **L9** and the planar (30π)‐bearing **L12** ligands thus exhibit a similar time‐gated phosphorescence profile recorded in glassy solution at 77 K with a pulsed xenon lamp source (Figure 7c). A red shift of the phosphorescence transition is observed along the **L3**→**L11**→(**L9 **≈ **L12**) series (Figure 7c) according to the electronic localization/delocalization within ligand scaffold.

**Figure 8:**
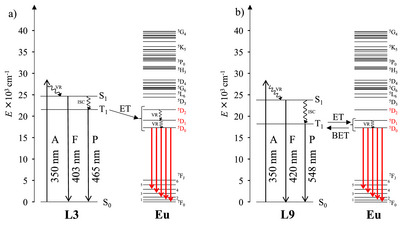
Jablonski diagrams built for a) [**L3**Eu(hfac)_3_], and b) [**L9**Eu(hfac)_3_] complexes.

In order to estimate the ligand‐centered singlet and triplet excited energy levels in the [**L*k*** Eu(hfac)_3_] (**L*k*** = **L2**, **L3**, and **L5**‐**L10**) complexes, the ligand‐based photophysical properties are investigated in the corresponding yttrium complexes [**L*k*** Y(hfac)_3_] (**L*k*** = **L2**, **L3**, and **L5**‐**L10**, synthesis is detailed in Appendix 1), which possess no accessible f‐electron level for being involved in intramolecular energy communication with the ligand‐based excited levels. The comparison with the electronic absorption profile of [**dig**Y(hfac)_3_] represented in Figures 7d and A4‐1 demonstrates that excitation at 350 nm (28 571 cm^−1^) into the aromatic ligands **L*k*** (**L*k*** = **L2**, **L3**, and **L5**‐**L10**) in [**L*k*** Y(hfac)_3_], for performing emission measurements, prevents the contribution of hfac^−^ to the light conversion process. The emission spectra of these yttrium complexes are shown in Figures 7d (right) and A4‐4, from which the bound ligand‐centered singlet excited transitions are established and gathered in Table A4‐2. Remarkably, heavy‐metal‐induced spin‐orbit coupling in the [**L7**Y(hfac)_3_] and [**L8**Y(hfac)_3_] complexes leads to dual fluorescence and phosphorescence emissions at room temperature; a rare behavior in these lanthanide adducts, which broadens potential practical applications in optoelectronic devices, smart anti‐counterfeiting, data encryption, and biomedicine due to their long lifetime and large shift between absorption and emission spectra.^[^ ^53^ ^]^ The emission spectra of the [**L*k*** Y(hfac)_3_] (**L*k*** = **L2**, **L3**, and **L5**‐**L10**) complexes, now recorded at 77 K, display both fluorescence and phosphorescence signals, while the related closed‐shell lanthanum complexes [**L11**La(hfac)_3_]^[^ ^27^ ^]^ and [**L12**La(hfac)_3_]^[^ ^27^ ^]^ exhibit only fluorescence profile in agreement with observations found in the corresponding free ligand (Figure A4‐4). Time‐resolved phosphorescence spectra of [**L3**Y(hfac)_3_] and [**L9**Y(hfac)_3_] are recorded in glassy solution at 77 K with a pulsed xenon lamp source (Figure 7e), allowing the detection of ligand‐based triplet excited ^3^π_1_*→^1^π transitions and completing the Jablonski diagrams for light conversion process in lanthanide complexes (Figure 8). When Y^3+^ is replaced with Eu^3+^ in the [**L*k*** Eu(hfac)_3_] complexes (**L*k*** = **L2**, **L3**, and **L5**‐**L10**), photoexcitation at 350 nm into the aromatic ligand‐centered excited states produces systematically the five characteristic Eu‐centered f‐f transitions: ^5^D_0_→^7^F_0_, ^5^D_0_→^7^F_1_, ^5^D_0_→^7^F_2_, ^5^D_0_→^7^F_3_, and ^5^D_0_→^7^F_4_ (Figures 7f and A4‐5a). The intense narrow signal at 614 nm is responsible for the pure red emission color of the Eu(III) ion. The excitation spectra monitored at 614 nm of the europium complexes shown in Figure A4‐6 are like their ground‐state absorption spectra, confirming that the bound ligand absorbs light and transfers its energy to the metal center via the antenna mechanism. The overall Eu‐centered quantum yield $\Phi_{\mathrm{tot}}^{Lk,\mathrm{Eu}}$ of [**L*k*** Eu(hfac)_3_] (**L*k*** = **L2**, **L3**, and **L5**‐**L10**) complexes upon ligand excitation is determined using the relative method with 9,10‐diphenylanthracene as the fluorescence standard (Table 3). [**L*k*** Eu(hfac)_3_] (**L*k*** = **L2**, **L3**, **L5**‐**L8**, and **L10**) complexes show similar and remarkable total photoluminescence quantum yield $(\Phi_{\mathrm{tot}}^{Lk,\mathrm{Eu}}\approx$ 60%) due to their comparable triplet excited state energy levels, which dominate the sensitization process (Figure 7a). The connection of electron‐donating (O, N) and electron‐withdrawing (Br) groups to the tridentate ligand framework does not affect the sensitization efficiency of the antenna ligand for europium‐centered luminescence. However, [**L9**Eu(hfac)_3_] displays a dramatically decreased photoluminescence quantum yield of $\Phi_{\mathrm{tot}}^{L9,\mathrm{Eu}}=9\left(1\right)$% upon photoexcitation. This behavior could be attributed to back energy transfer (BET) from the emissive Eu‐centered levels to the low‐energy triplet excited ^3^π_1_*→^1^π state due to their minor energy difference (Figure 7b), thereby reducing the ligand‐sensitized photoluminescence quantum yield. The room temperature emission spectrum of [**L9**Eu(hfac)_3_] (Figure 7f) displays residual ^1^π_1_*→^1^π and ^3^π_1_*→^1^π transitions compatible with BET process, which vanishes when the temperature is reduced to 77 K (Figure A4‐5b).

**Table 3:** Photoluminescence quantum yield of the target europium complexes.

Complex	$\Phi_{\mathrm{tot}}^{Lk,\mathrm{Eu}}$ [%]	Complex	$\Phi_{\mathrm{tot}}^{Lk,\mathrm{Eu}}$ [%]
[**L2**Eu(hfac)_3_]	65(2)	[**L8**Eu(hfac)_3_]	64(2)
[**L3**Eu(hfac)_3_]	59(2)	[**L9**Eu(hfac)_3_]	9(1)
[**L5**Eu(hfac)_3_]	59(2)	[**L10**Eu(hfac)_3_]	54(2)
[**L6**Eu(hfac)_3_]	61(2)	[**L11**Eu(hfac)_3_]	58.8^[^ ^27^ ^]^
[**L7**Eu(hfac)_3_]	66(2)	[**L12**Eu(hfac)_3_]	1.6^[^ ^27^ ^]^

## 3 Conclusion

All (Brønsted base unit)‐bearing ligands **L1**‐**L8** display no catalytic behavior for the Michael reaction between the Michael donor **1** and the Michael acceptor **2** due to the weak reactivity or the absence of an acidic group in their scaffolds. Trivalent europium ion is therefore exploited to not only (i) enhance the acidity of N‐H unit of the bound aromatic ligand in [**L*k*** Eu(hfac)_3_] (**L*k*** = **L5** and **L7**), but also (ii) structurally constrain the bound ligands in the *cis‐cis* orientation required for bifunctional catalytic ability and (iii) introduce additional acidic bidentate *β*‐diketonate co‐ligands into the molecular assemblies for activating simultaneously the two Michael substrates **1** and **2** on the second‐sphere coordination of the central metal for producing compound **3** with a yield of up to 92% after 72 hours. However, these bifunctional catalysts display a limited enantioselectivity, attributed to the significant spatial separation between the chiral carbons and the hydrogen‐binding sites in the catalysts scaffold.

Emission parameters in the free ligands **L*k*** (**L*k*** = **L2**, **L3**, and **L5**‐**L10**) and in their yttrium complexes [**L*k*** Y(hfac)_3_] can be tuned from fluorescence to phosphorescence by controlling the planarity of their backbones in the ground state. At room temperature, [**L7**Y(hfac)_3_] and [**L8**Y(hfac)_3_] display a rare dual fluorescence/phosphorescence emission promoted by the heavy‐metal effect. The direct attachment of electron‐donating (O, N) and electron‐withdrawing (Br) groups to the aromatic ligands scaffold displays no effect on the sensitization efficiency of the bound ligands for europium‐based luminescence. Planarization of the bound **L9** ligand in the triplet excited state induces electronic delocalization within the sensitizer scaffold in the europium complex and consequently lowers its triplet excited state energy level, causing a poor photoluminescence quantum yield for [**L9**Eu(hfac)_3_] in comparison with its [**L*k*** Eu(hfac)_3_] (**L*k*** = **L2**, **L3**, **L5**‐**L8**, and **L10**) analogues.

This work highlights a rare case of dual functionality for lanthanide complexation, which induces catalytic properties on one part (Michael addition) and efficient light downshifting on the other side.

## 4 Experimental Section

### General

All reagents were purchased from Alfa Aesar, FluroChem, Acros, Fischer Chemicals AG, and Sigma‐Aldrich, and used as received. Compounds **S1** ^[^ ^54^ ^]^ and [**dig**Eu(hfac)_3_]^[^ ^55^ ^]^ were prepared according to literature. THF was distilled over sodium/benzophenone under nitrogen atmosphere. The CCDC deposition numbers for the crystal structures of the synthesized compounds can be found in ref. [56].

### L1

A mixture of **S1** (1.4 g, 6.7 mmol) and 1,2‐phenyldiamine (1.65 g, 15 mmol) in 85% H_3_PO_4_ (25 mL) was stirred at 220 °C under nitrogen atmosphere for 6 hours and then poured into 300 mL of cold water. The pH was adjusted to pH 9 with 25% NH_4_OH. The formed precipitate was filtered off and redissolved in hot DMSO (300 mL). H_2_O (300 mL) was added. The precipitate was filtered and dried overnight under reduced pressure to give **L1** as a white solid (1.38 g, 59%).^1^H NMR (400 MHz, DMSO‐*d6*): 12.87 (s, 2H), 7.74 (d, *J* = 7.9 Hz, 2H), 7.69 (d, *J* = 1.1 Hz, 2H), 7.58 (s, 2H), 7.33 (t, *J* = 1.3 Hz, 2H), 7.26 (t, *J* = 1.2 Hz, 2H), 3.18 (s, 6H). ^13^C NMR (101 MHz, DMSO‐*d6*): 156.06, 151.89, 148.10, 144.36, 134.62, 123.85, 122.54, 119.81, 112.09, 103.98. ESI‐MS calculated for [C_21_H_18_N_6_ + H]^+^ ([**L1** + H]^+^): m/z 355.2; found: 355.6.

### L2

A mixture of **L1** (1.50 g, 4.23 mmol) and 60% NaH (0.51 g, 12.75 mmol) in dry THF (50 mL) was stirred at r.t for 1 hour. (*S*)‐(+)1‐bromo‐2‐methylbutane (1.96 g, 12.98 mmol) and KI (catalytic amount) were added. The solution was heated at 50 °C for 24 hours and then quenched with water (50 mL). The mixture was extracted with CH_2_Cl_2_ (3×50 mL), dried over Na_2_SO_4_ and evaporated to dryness. The residue was purified by column chromatography (SiO_2_, CH_2_Cl_2_/MeOH 100/0 to 100/3) to afford **L2** (0.73 g, 41%) as a white solid. ^1^H NMR (400 MHz, CD_2_Cl_2_): 12.71 (s, 1H), 8.01 – 7.94 (m, 1H), 7.90 (d, *J* = 7.9 Hz, 1H), 7.81 (d, *J* = 8.0 Hz, 1H), 7.47 – 7.30 (m, 5H), 7.25 – 7.18 (m, 1H), 7.15 (d, *J* = 2.6 Hz, 1H), 4.00 – 3.77 (m, 2H), 2.80 (s, 6H), 1.67 – 1.52 (m, 1H), 1.11 – 0.80 (m, 2H), 0.63 (t, *J* = 7.4 Hz, 3H), 0.50 (d, *J* = 6.7 Hz, 3H). ^13^C NMR (101 MHz, CD_2_Cl_2_): 155.04, 152.25, 151.82, 150.04, 147.86, 144.45, 142.42, 136.37, 134.76, 123.10, 122.55, 122.41, 121.99, 119.44, 119.27, 111.48, 111.36, 108.01, 104.02, 49.56, 38.85, 35.43, 26.86, 16.58, 10.76. ESI‐MS calculated for [C_26_H_28_N_6_ + H]^+^ ([**L2** + H]^+^): m/z 425.3; found: 425.6. Elemental analysis calculated for C_26_H_28_N_6_·0.3CH_2_Cl_2_ (**L2**·0.3CH_2_Cl_2_) (%): C 70.19, H 6.41, N 18.67; found (%): C 70.47, H 6.25, N 18.57. ${\left[\alpha \right]}_{D}^{20}$ = + 3.15 (c = 1.05, CH_2_Cl_2_).

### [**L2**Eu(hfac)_3_]

A mixture of **L2** (0.2 g, 0.47 mmol) and [**dig**Eu(hfac)_3_] (0.47 g, 0.52 mmol) in CH_2_Cl_2_ (5 mL) was stirred at r.t for 30 minutes and then evaporated to dryness. The residue was purified by precipitation in pentane to afford [**L2**Eu(hfac)_3_] as a white solid (0.52 g, 92%). ^1^H NMR (400 MHz, CD_2_Cl_2_): 24.21 (br, 1H), 18.87 (br, 1H), 11.42 (br, 1H), 10.69 (br, 1H), 10.43 (br, 1H), 9.86 (t, *J* = 7.7 Hz, 1H), 9.67 (t, *J* = 7.6 Hz, 1H), 8.59 (d, *J* = 7.1 Hz, 1H), 8.49 (d, *J* = 8.4 Hz, 1H), 5.56 (s, 1H), 4.87 (dd, *J* = 15.8, 7.0 Hz, 1H), 4.74 (dd, *J* = 15.7, 8.3 Hz, 1H), 3.97 (br, 1H), 3.17 (s, 6H), 3.11 (s, 3H), 2.25 (br, 1H), 1.91–1.82 (m, 1H), 1.63–1.48 (m, 1H), 1.17 (d, *J* = 6.5 Hz, 3H), 0.95 (t, *J* = 7.4 Hz, 3H). ^13^C NMR (101 MHz, CD_2_Cl_2_): 160.84, 160.51, 160.17, 159.84, 159.58, 157.68, 156.32, 152.98, 144.13, 142.07, 132.83, 131.99, 130.88, 130.23, 129.68, 127.83, 127.74, 118.49, 112.90, 111.54, 75.95, 74.55, 63.92, 61.93, 61.07, 58.22, 55.37, 50.47, 38.76, 37.36, 27.28, 16.60, 10.97. ESI‐MS calculated for [C_41_H_31_N_6_O_6_F_18_Eu – hfac]^+^ ([[**L2**Eu(hfac)_3_] – hfac]^+^): m/z 991.1; found: 991.7. Elemental analysis calculated for C_41_H_31_N_6_O_6_F_18_Eu ([**L2**Eu(hfac)_3_]) (%): C 41.12, H 2.61, N 7.02; found (%): C 40.81, H 2.63, N 6.95. ${\left[\alpha \right]}_{D}^{20}$ = ‐3.97 (c = 0.37, CH_2_Cl_2_).

## Supporting Information

The authors have cited additional references within the Supporting Information (SI).^[^ ^55^, ^57^, ^58^, ^59^, ^60^, ^61^, ^62^, ^63^, ^64^ ^]^ SI contains the following information: Experimental Section, 1D, and 2D NMR spectra, crystal structures, thermodynamic data, and photophysical data for synthesized compounds.

## Conflict of Interest

The authors declare no conflict of interest

## Supporting information



Supporting Information

Supporting Information

## Data Availability

The data that support the findings of this study are available from the corresponding author upon reasonable request.
