# Supplementary material for: Catalytic and Photoluminescence Properties of the First‐ and Second‐Sphere Coordination of Lanthanide Complexes
Source: Chemistry. 2025 Oct 8;31(61):e02338. doi: 10.1002/chem.202502338 (PMC12587021; doi:10.1002/chem.202502338)
Supplement: Supplementary file 1 — Supporting Information [file CHEM-31-e02338-s001.pdf]

Catalytic and Photoluminescence Properties of the First- and Second-Sphere Coordination of Lanthanide Complexes

Giau Le-Hoang,*[a] Laure Guénée,[b] Clémence Delage de Luget,[a] Julien Chong,[a] and Claude Piguet*[a]

[a] Department of Inorganic and Analytical Chemistry, University of Geneva, 30 quai E. Ansermet, CH-1211 Geneva 4, Switzerland.

Emails: Hoang.Le@unige.ch, Claude.Piguet@unige.ch

[b] Laboratory of Crystallography, University of Geneva, 24 quai E. Ansermet, CH-1211 Geneva 4, Switzerland.

Supporting Information

(130 pages)

General. All reagents were purchased from Alfa Aesar, FluroChem, Acros, Fischer Chemicals AG and Sigma-Aldrich, and used as received. Compounds **L9**,[26] **L10**,[26] [**L9**La(hfac)$_3$],[26] [**L10**La(hfac)$_3$],[26] [**dig**Eu(hfac)$_3$],[58] [**dig**Y(hfac)$_3$],[59] and **S2** [57] were prepared according to literature. Dichloromethane was distilled over calcium hydride. Enantiomers ratios were determined by chiral HPLC Shimadzu using Chiralpak IC column (1 cm x 25 cm) and hexane/EtOH 98/2 as eluent.

Spectroscopic and analytical measurements

^{1}H and ^{13}C NMR spectra were recorded at 298 K on a Bruker Avance 400 MHz spectrometer equipped with BCU temperature control for variable temperature measurements. Electrospray (ESI-MS) mass spectra were recorded on an Applied Biosystems API 150EX LC/MS System equipped with a Turbo Ionspray source. Elemental analyses were performed by K. L. Paglia from the Microchemical Laboratory of the University of Geneva. Electronic spectra in the UV-Vis region were recorded at 293 K from solutions in CH_2Cl_2 with a Perkin-Elmer Lambda 1050. The emission spectra were recorded using a Fluorolog (Horiba Jobin-Yvon) instrument equipped with an iHR320 imaging spectrometer, a 450 W xenon lamp illuminator (FL-1039A/40A) and a Peltier-cooled photomultiplier tube (PMT Hamamatsu R928P). The emission spectra were corrected for the wavelength-dependent sensitivity of the PMT. Time-resolved data were collected using a digital oscilloscope (Tektronix MDO4104C) coupled to a peltier-cooled photomultiplier tube (PMT Hamamatsu R928P) or to a time-gated photomultiplier module (Hamamatsu H11526-20-NF). Pulsed excitation at 355 nm was achieved using the third harmonic of a pulsed Nd:YAG laser (Quantel Q-Smart 850). Complexes of known molecular weight were dissolved in CH_2Cl_2 to obtain ~10^{-5} M solution in 1 cm cylindrical quartz cuvettes for photophysical measurements. The standard xenon lamp of the Horiba Scientific Fluorolog 3 spectrofluorometer has been used for UV excitation of the samples to record the pertinent emission spectra, where the appropriate longpass

filters (Thorlabs) have been placed after the sample to remove the second-order Rayleigh scattering of the xenon lamp. The emission spectra were corrected for the instrumental response function. The mathematical analyses were performed by using Origin 2017 (OriginLab Corporation) and Excel® (Microsoft) software.

Experimental X-ray crystallography

Single crystals were mounted on Hampton cryoloops with protection oil. X-ray data collections were performed with a XtaLAB Synergy-S diffractometer equipped with a hybrid pixel array "hypix arc 150" detector (Cu[Kα] radiation). Structures were solved by using dual-space methods (ShelXT)[S1]. Full-matrix least-square refinements on F^2 were performed with SHELXL[60,61] and all other calculations were performed with OLEX2[62] and ORTEP[63] programs.

CCDC 2472888-2472901 contain the supplementary crystallographic data for this paper. These data can be obtained free of charge from The Cambridge Crystallographic Data Centre via www.ccdc.cam.ac.uk/data_request/cif.

Appendix 1: Synthesis of target ligands L1-L8.

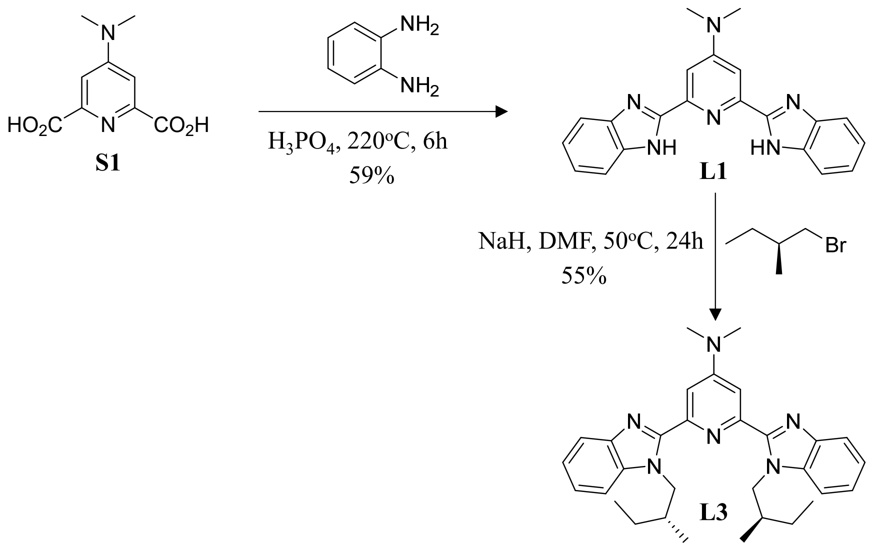

Scheme A1-1. Synthesis of the tridentate ligand **L3**.

L3. A mixture of **L1** (3.0 g, 8.46 mmol) and 60% NaH (1.0 g, 25.0 mmol) in dry DMF (50 mL) was stirred at r.t for 1 h. (*S*)-(+)1-bromo-2-methylbutane (3.67 g, 24.30 mmol) and KI (catalytic amount) were added. The solution was heated at 50 °C for 24 h and then quenched with water (50 mL). The mixture was extracted with CH_2Cl_2 (3 x 50 mL), dried over Na_2SO_4 and evaporated to dryness. The residue was purified by column chromatography (SiO_2, CH_2Cl_2/MeOH 100/0 to 100/10) to afford **L3** (2.31 g, 55%) as a white solid. ^{1}H NMR (400 MHz, CD_2Cl_2): 7.86 – 7.77 (m, 2H), 7.59 (s, 2H), 7.53 – 7.45 (m, 2H), 7.41 – 7.27 (m, 4H), 4.77 (dd, *J* = 13.9, 6.8 Hz, 2H), 4.63 (dd, *J* = 13.9, 8.1 Hz, 2H), 3.23 (s, 6H), 1.98 – 1.84 (m, 2H), 1.30 – 1.15 (m, 2H), 1.09 – 0.98 (m, 2H), 0.73 – 0.64 (m, 12H). ^{13}C NMR (101 MHz, CD_2Cl_2): 155.50, 151.75, 150.36, 142.69, 136.90, 122.67, 122.03, 119.68, 110.70, 107.88, 50.58, 39.35, 35.69, 26.92, 16.68, 10.79. ESI-MS calculated for $[C_{31}H_{38}N_6 + H]^+$ ($[\mathbf{L3} + H]^+$): *m/z* 495.3; found: 495.8. Elemental analysis calculated for $C_{31}H_{38}N_6$ (**L3**) (%): C 75.27, H 7.74, N 16.99; found (%): C 75.02, H 8.16, N 16.65. $[\alpha]_D^{20}$ = -2.34 (c = 1.08, CH_2Cl_2).

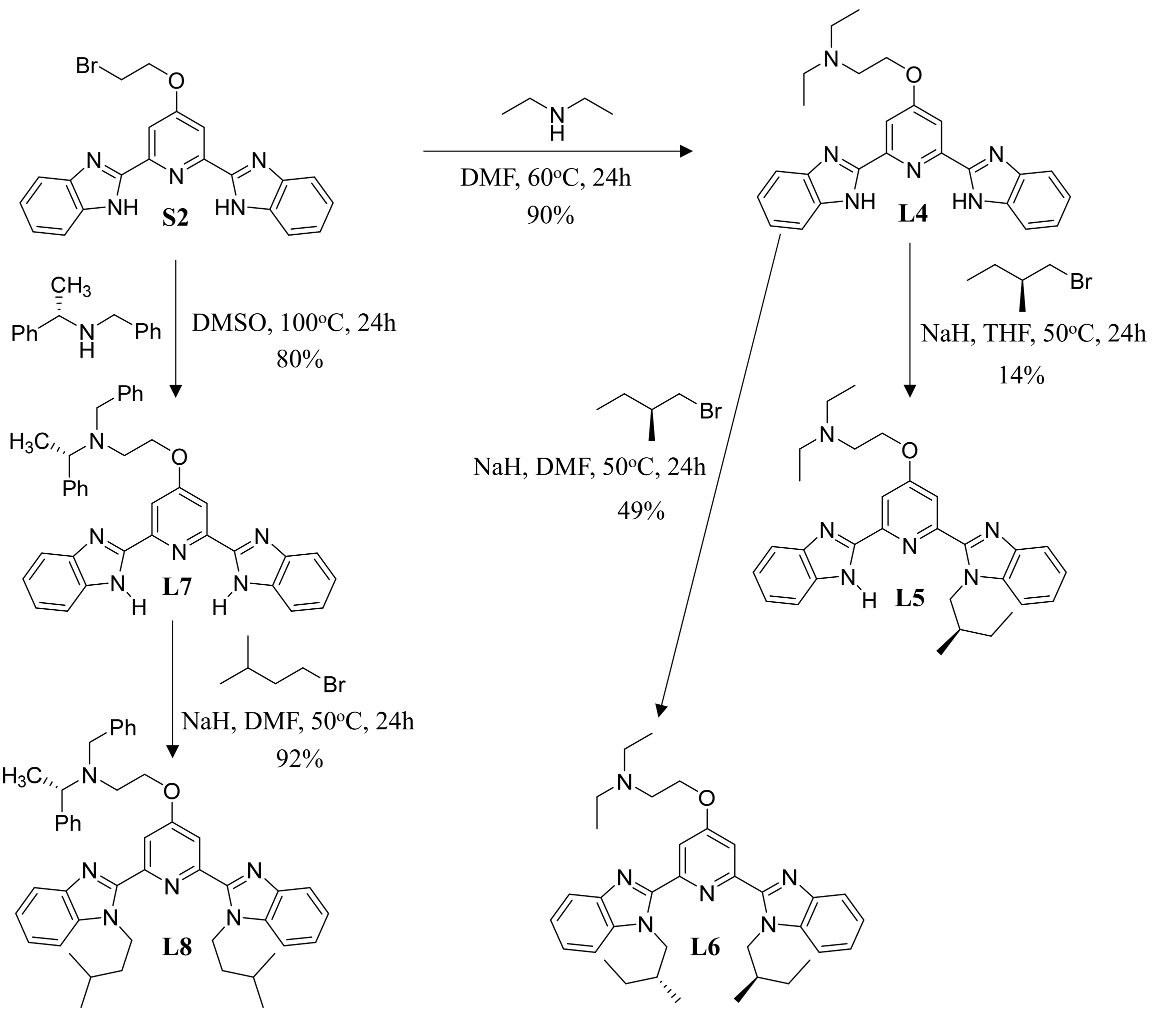

Scheme A1-2. Synthesis of the tridentate ligands **L5**-**L8**.

L4. A mixture of **S2** (1.2 g, 2.76 mmol) and Et_2NH (2.12 g, 28.99 mmol) in DMF (5 mL) was stirred at 50 °C for 24 h and then added to water (50 mL). The mixture adjusted to pH 9 with 37% HCl. The solid was filtered off and purified precipitation in pentane to afford **L4** (1.06 g, 90%) as a white solid. ^{1}H NMR (400 MHz, DMSO-*d6*): 12.95 (s, 2H), 7.83 (s, 2H), 7.78 (d, *J* = 8.0 Hz, 2H), 7.74 (d, *J* = 8.0 Hz, 2H), 7.36 (t, *J* = 7.7 Hz, 2H), 7.29 (t, *J* = 7.7 Hz, 2H), 4.36 (t, *J* = 5.8 Hz, 2H), 2.89 (t, *J* = 5.8 Hz, 2H), 2.60 (q, *J* = 7.1 Hz, 4H), 1.02 (t, *J* = 7.1 Hz, 6H). ^{13}C NMR (101 MHz, DMSO-*d6*): 167.10, 150.88, 149.87, 144.51, 134.80, 124.17, 122.68, 120.13, 112.25,

107.93, 67.92, 51.42, 47.53,12.48. ESI-MS calculated for $[C_{25}H_{26}N_6O + H]^+$ ([**L4** + H]$^+$): *m/z* 427.2; found: 427.6.

L5. A mixture of **L4** (2.80 g, 6.56 mmol) and 60% NaH (0.79 g, 19.75 mmol) in dry THF (100 mL) was stirred at r.t for 1 h. (*S*)-(+)1-bromo-2-methylbutane (2.94 g, 19.43 mmol) and KI (catalytic amount) were added. The solution was heated at 50 °C for 24 h and then quenched with water (50 mL). The mixture was extracted with CH_2Cl_2 (3 x 100 mL), dried over Na_2SO_4 and evaporated to dryness. The residue was purified by column chromatography (SiO_2, CH_2Cl_2/MeOH 100/0 to 100/5) to afford **L5** (0.46 g, 14%) as a white solid. ^{1}H NMR (400 MHz, CD_2Cl_2): 13.11 (s, 1H), 8.06 – 7.99 (m, 1H), 7.96 (d, *J* = 7.9 Hz, 1H), 7.86 – 7.80 (m, 1H), 7.68 (d, *J* = 2.4 Hz, 1H), 7.52 – 7.31 (m, 5H), 7.23 – 7.17 (m, 1H), 3.75 (t, *J* = 6.0 Hz, 2H), 3.72 – 3.55 (m, 2H), 2.66 (t, *J* = 5.9 Hz, 2H), 2.47 (q, *J* = 7.1 Hz, 4H), 1.48 (tq, *J* = 14.7, 7.6 Hz, 1H), 1.08 – 1.01 (m, 1H), 0.97 (t, *J* = 7.1 Hz, 6H), 0.84 (dt, *J* = 14.0, 7.3 Hz, 1H), 0.60 (t, *J* = 7.4 Hz, 3H), 0.44 (d, *J* = 6.7 Hz, 3H). ^{13}C NMR (101 MHz, CD_2Cl_2): 166.00, 151.32, 151.05, 150.82, 149.49, 144.46, 142.42, 136.22, 134.98, 123.51, 122.93, 122.76, 122.25, 119.82, 119.46, 111.61, 111.59, 111.53, 108.19, 67.24, 51.15, 49.31, 47.56, 35.38, 26.79, 16.53, 11.85, 10.71. ESI-MS calculated for $[C_{30}H_{36}N_6O + H]^+$ ([**L5** + H]$^+$): *m/z* 497.3; found: 497.4. Elemental analysis calculated for $C_{30}H_{36}N_6O$ (**L5**) (%): C 72.55, H 7.31, N 16.92; found (%): C 72.08, H 7.48, N 16.85. $[\alpha]_D^{20}$ = - 1.18 (c = 1.12, CH_2Cl_2).

L6. A mixture of **L4** (0.72 g, 1.69 mmol) and 60% NaH (0.21 g, 5.25 mmol) in dry DMF (20 mL) was stirred at r.t for 1 h. (*S*)-1-bromo-2-methylbutane (0.80 g, 5.26 mmol) and KI (catalytic amount) were added. The solution was heated at 50 °C for 24 h and then quenched with water (50 mL). The mixture was extracted with CH_2Cl_2 (3 x 50 mL), dried over Na_2SO_4 and evaporated to dryness. The residue was purified by column chromatography (SiO_2, CH_2Cl_2/MeOH 100/0 to 100/3) to afford **L6** (0.46 g, 49%) as a white solid. ^{1}H NMR (400 MHz, CD_2Cl_2): 7.92 (s, 2H), 7.86

– 7.80 (m, 2H), 7.55 – 7.49 (m, 2H), 7.41 – 7.32 (m, 4H), 4.77 (dd, *J* = 13.9, 6.8 Hz, 2H), 4.64 (dd, *J* = 13.9, 8.1 Hz, 2H), 4.56 (t, *J* = 5.4 Hz, 2H), 3.24 (t, *J* = 5.4 Hz, 2H), 2.96 (q, *J* = 7.2 Hz, 4H), 1.96 – 1.85 (m, 2H), 1.29 (t, *J* = 7.2 Hz, 6H), 1.24 – 1.16 (m, 2H), 1.08 – 0.98 (m, 2H), 0.73 – 0.64 (m, 12H). ^{13}C NMR (101 MHz, CD_2Cl_2): 165.77, 151.96, 150.38, 142.67, 136.94, 123.17, 122.37, 119.95, 111.66, 110.85, 51.00, 50.68, 47.74, 35.75, 26.90, 16.64, 10.78. ESI-MS calculated for $[C_{35}H_{46}N_6O + H]^+$ ($[M + H]^+$): *m/z* 567.4; found: 567.7. $[\alpha]_D^{20}$ = - 3.68 (c = 1.58, CH_2Cl_2).

L7. A mixture of **S2** (2.0 g, 4.61 mmol) and *S*-(-)-N-benzyl-α-methylbenzylamine (10 g, 47.32 mmol) in dry DMSO (5 mL) was stirred at 100 °C for 24 h. The mixture was extracted with CH_2Cl_2 (3 x 50 mL), dried over Na_2SO_4 and evaporated to dryness. The residue was purified by column chromatography (SiO_2, CH_2Cl_2/MeOH 100/0 to 100/5) to afford **L7** (2.07 g, 80%) as a white solid. ^{1}H NMR (400 MHz, CD_2Cl_2): 11.78 (br, 2H), 7.91 (s, 2H), 7.83 (br, 2H), 7.45 – 7.11 (m, 16H), 3.97 – 3.85 (m, 3H), 3.68 – 3.52 (m, 2H), 2.91 – 2.67 (m, 2H), 1.37 (dd, *J* = 6.7, 1.2 Hz, 3H). ^{13}C NMR (101 MHz, CD_2Cl_2): 166.00, 151.32, 151.05, 150.82, 149.49, 144.46, 142.42, 136.22, 134.98, 123.51, 122.93, 122.76, 122.25, 119.82, 119.46, 111.61, 111.59, 111.53, 108.19, 67.24, 51.15, 49.31, 47.56, 35.38, 26.79, 16.53, 11.85, 10.71. ESI-MS calculated for $[C_{36}H_{32}N_6O + H]^+$ ([**L7** + H]$^+$): *m/z* 565.3; found: 565.7. Elemental analysis calculated for $C_{36}H_{32}N_6O \cdot 0.4CH_2Cl_2$ (**L7**$\cdot 0.4CH_2Cl_2$) (%): C 73.03, H 5.52, N 14.04; found (%): C 72.98, H 5.20, N 14.04. $[\alpha]_D^{20}$ = - 47.85 (c = 0.79, CH_2Cl_2).

L8. A mixture of **L7** (1.0 g, 1.77 mmol) and 60% NaH (0.21 g, 5.25 mmol) in dry DMF (5 mL) was stirred at r.t for 0.5 h. 1-Bromo-3-methylbutane (5.04 g, 33.37 mmol) was added. The solution was heated at 50 °C for 24 h and then quenched with water (20 mL). The mixture was extracted with CH_2Cl_2 (3 x 100 mL), dried over Na_2SO_4 and evaporated to dryness. The residue was purified by column chromatography (SiO_2, CH_2Cl_2/MeOH 100/0 to 100/5) to afford **L8** (1.15 g, 92%) as a

light-yellow solid. ^{1}H NMR (400 MHz, CD_2Cl_2): 7.86 – 7.81 (m, 2H), 7.77 (s, 2H), 7.55 – 7.50 (m, 4H), 7.49 – 7.45 (m, 2H), 7.42 – 7.31 (m, 8H), 7.29 – 7.20 (m, 2H), 4.82 – 4.74 (m, 4H), 4.26 – 4.21 (m, 2H), 4.10 (q, *J* = 6.9 Hz, 1H), 3.85 – 3.71 (m, 2H), 3.16 – 3.07 (m, 1H), 3.01 – 2.92 (m, 1H), 1.71 – 1.60 (m, 4H), 1.52 (d, *J* = 6.8 Hz, 3H), 1.48 – 1.38 (m, 2H), 0.75 (d, *J* = 6.6 Hz, 12H). ^{13}C NMR (101 MHz, CD_2Cl_2): 166.34, 151.63, 150.30, 143.62, 142.89, 140.50, 136.36, 128.58, 128.20, 128.08, 127.84, 126.80, 126.77, 123.13, 122.33, 119.98, 111.72, 110.32, 67.90, 59.03, 55.43, 48.42, 43.44, 38.73, 25.81, 21.92, 15.01. ESI-MS calculated for $[C_{46}H_{52}N_6O + H]^+$ ([**L8** + H]$^+$): *m/z* 705.4; found: 705.6. Elemental analysis calculated for $C_{46}H_{52}N_6O$ (**L8**) (%): C 78.37, H 7.44, N 11.92; found (%): C 77.96, H 7.67, N 11.85. $[\alpha]_D^{20}$ = -42.74 (c = 0.74, CH_2Cl_2).

General procedure for synthesis of [L*k*Ln(hfac)$_3$] (L*k* = L2, L3, L5-L8, Ln = Eu, Y) complexes

A mixture of ligand and [**dig**Ln(hfac)$_3$] (1.05 eq.) in CH_2Cl_2 was stirred at r.t for 30 min and then evaporated to dryness. The residue was purified by precipitation in pentane to afford [**L*k***Ln(hfac)$_3$] as a white solid.

[L3Eu(hfac)$_3$]. Yield: 85%. ^{1}H NMR (400 MHz, CD_2Cl_2): 21.44 (br, 2H), 10.81 (t, *J* = 6.5 Hz, 2H), 9.71 (t, *J* = 7.0 Hz, 2H), 8.57 (d, *J* = 0.9 Hz, 2H), 5.50 (dd, *J* = 15.4, 7.0 Hz, 2H), 5.35 (dd, 2H), 5.08 (s, 2H), 2.97 (s, 9H), 2.76 – 2.65 (m, 2H), 2.00 – 1.85 (m, 2H), 1.65 – 1.53 (m, 2H), 1.23 (d, *J* = 6.6 Hz, 6H), 0.94 (t, *J* = 7.4 Hz, 6H). ^{13}C NMR (101 MHz, CD_2Cl_2): 159.73, 153.31, 152.11, 146.01, 131.11, 130.41, 127.39, 117.52, 111.73, 78.00, 63.31, 61.42, 60.45, 57.60, 54.74, 51.25, 38.89, 37.68, 27.36, 16.82, 10.99. ESI-MS calculated for $[C_{46}H_{41}F_{18}N_6O_6Eu - hfac]^+$ ([[**L3**Eu(hfac)$_3$] - hfac]$^+$): *m/z* 1061.7; found: 1061.2. Elemental analysis calculated for $C_{46}H_{41}F_{18}N_6O_6Eu$ ([**L3**Eu(hfac)$_3$]) (%): C 43.58, H 3.26, N 6.63; found (%): C 43.51, H 3.28, N 6.62. $[\alpha]_D^{20}$ = - 9.38 (c = 0.98, CH_2Cl_2).

[L5Eu(hfac)$_3$]. Yield: 66%. ^{1}H NMR (400 MHz, CD_2Cl_2): 24.70 (br, 1H), 19.33 (br, 2H), 11.47 (br, 1H), 10.53 (br, 1H), 9.89 (t, J = 7.7 Hz, 1H), 9.78 (t, J = 7.7 Hz, 1H), 8.80 (d, J = 8.3 Hz, 1H), 8.56 (d, J = 8.3 Hz, 1H), 6.97 (s, 2H), 5.26 – 5.21 (m, 2H), 5.04 (dd, J = 15.8, 8.3 Hz, 1H), 4.42 – 4.29 (m, 2H), 3.05 – 2.46 (m, 9H), 2.45 – 2.34 (m, 1H), 1.99 – 1.87 (m, 1H), 1.26 (d, J = 6.5 Hz, 3H), 1.03 (2t, J = 7.4, 7.3 Hz, 9H). ^{13}C NMR (101 MHz, CD_2Cl_2): 173.32, 159.53, 157.98, 156.45, 143.81, 132.81, 131.10, 130.48, 129.69, 128.09, 118.60, 113.33, 111.67, 83.15, 81.47, 67.99, 62.44, 60.77, 59.59, 56.73, 50.54, 47.63, 37.70, 27.34, 16.91, 10.99. ESI-MS calculated for [$C_{45}H_{39}F_{18}N_6O_7Eu$ - hfac]$^+$ ([[**L5**Eu(hfac)$_3$] - hfac]$^+$): *m/z* 1063.2; found: 1063.7. Elemental analysis calculated for $C_{45}H_{39}F_{18}N_6O_7Eu{\cdot}0.25CH_2Cl_2$ ([**L5**Eu(hfac)$_3$]·0.25CH_2Cl_2) (%): C 42.10, H 3.08, N 6.51; found (%): C 41.90, H 3.08, N 6.35. $[\alpha]_D^{20}$ = + 1.39 (c = 0.52, CH_2Cl_2).

[L6Eu(hfac)$_3$]. Yield: 79%. ^{1}H NMR (400 MHz, CD_2Cl_2): 21.85 (br, 2H), 10.87 (d, J = 6.4 Hz, 2H), 9.79 – 9.71 (m, 2H), 8.64 (d, J = 8.3 Hz, 2H), 6.41 (s, 2H), 5.89 (dd, J = 15.4, 6.8 Hz, 2H), 5.71 (dd, J = 15.4, 8.3 Hz, 2H), 4.25 (br, 2H), 2.96 – 2.41 (m, 11H), 2.11 – 1.99 (m, 2H), 1.82 – 1.72 (m, 2H), 1.37 (d, J = 6.5 Hz, 6H), 1.08 – 0.93 (m, 12H). ^{13}C NMR (101 MHz, CD_2Cl_2): 173.60, 158.75, 154.24, 153.58, 144.48, 131.21, 130.76, 127.77, 117.53, 111.92, 84.78, 68.48, 62.09, 60.39, 59.24, 56.39, 51.44, 51.31, 47.84, 38.07, 27.45, 17.03, 11.51, 11.13, 11.02. ESI-MS calculated for [$C_{40}H_{49}N_6O_7F_{18}Eu$ - hfac]$^+$ ([[**L6**Eu(hfac)$_3$] - hfac]$^+$): *m/z* 1133.3; found: 1133.9. Elemental analysis calculated for $C_{40}H_{49}N_6O_7F_{18}Eu$ ([**L6**Eu(hfac)$_3$]) (%): C 44.82, H 3.69, N 6.27; found (%): C 44.25, H 3.79, N 6.13. $[\alpha]_D^{20}$ = - 5.27 (c = 0.57, CH_2Cl_2).

[L7Eu(hfac)$_3$]. Yield: 83%. ^{1}H NMR (400 MHz, CD_2Cl_2): 21.45 (br, 2H), 10.84 (t, J = 6.4 Hz, 2H), 10.06 (s, 2H), 9.68 (t, J = 7.7 Hz, 2H), 8.49 (d, J = 8.1 Hz, 2H), 7.51 – 7.21 (m, 10H), 5.90 (s, 2H), 4.25 (t, J = 6.2 Hz, 2H), 4.05 (q, J = 6.9 Hz, 1H), 3.84 – 3.70 (m, 2H), 3.07 – 2.90 (m, 2H), 2.77 (s, 3H), 1.48 (d, J = 6.8 Hz, 3H). ^{13}C NMR (101 MHz, CD_2Cl_2): 174.09, 159.98, 149.63,

146.57, 144.92, 143.26, 140.49, 130.79, 130.20, 128.54, 128.49, 128.37, 128.32, 128.19, 128.13, 127.82, 127.76, 126.98, 112.96, 112.38, 81.65, 68.73, 60.48, 59.68, 59.09, 56.24, 56.02, 48.76, 14.53. ESI-MS calculated for [$C_{51}H_{35}N_6O_7F_{18}Eu$ - hfac]$^+$ ([[**L7**Eu(hfac)$_3$] - hfac]$^+$): *m/z* 1131.2; found: 1131.0. Elemental analysis calculated for $C_{51}H_{35}N_6O_7F_{18}Eu$ ([**L7**Eu(hfac)$_3$]) (%): C 45.79, H 2.64, N 6.28; found (%): C 45.68, H 2.63, N 6.16. $[\alpha]_D^{20}$ = - 17.01 (c = 0.46, CH_2Cl_2).

[L8Eu(hfac)$_3$]. Yield: 90%. ^{1}H NMR (400 MHz, CD_2Cl_2): 22.24 (br, 2H), 11.29 – 11.02 (m, 2H), 10.00 (t, *J* = 7.7 Hz, 2H), 8.93 (d, *J* = 16.8 Hz, 2H), 7.43 – 6.86 (m, 10H), 5.74 (s, 2H), 5.71 – 5.60 (m, 4H), 3.93 – 3.78 (m, 3H), 3.66 – 3.51 (m, 2H), 2.99 – 2.89 (m, 4H), 2.89 – 2.80 (m, 1H), 2.79 – 2.68 (m, 1H), 2.64 (br, 3H), 2.39 – 2.25 (m, 2H), 1.43 – 1.26 (m, 15H). ^{13}C NMR (101 MHz, CD_2Cl_2): 173.44, 158.91, 158.54, 156.50, 151.95, 144.33, 142.96, 140.09, 132.37, 131.23, 128.38, 128.23, 128.06, 127.97, 127.57, 126.85, 118.44, 111.46, 82.14, 68.62, 62.11, 60.24, 59.53, 59.26, 56.41, 55.90, 48.54, 44.26, 40.79, 26.95, 22.48, 22.33. ESI-MS calculated for [$C_{61}H_{55}N_6O_7F_{18}Eu$ - hfac]$^+$ ([[**L8**Eu(hfac)$_3$] - hfac]$^+$): *m/z* 1271.3; found: 1271.9. Elemental analysis calculated for $C_{61}H_{55}N_6O_7F_{18}Eu \cdot 0.3C_5H_{12}$ ([**L8**Eu(hfac)$_3$]·0.3Pentane) (%): C 50.05, H 3.94, N 5.60; found (%): C 50.18, H 3.93, N 5.60. The peaks of pentane were observed in ^{1}H and ^{13}C NMR spectra (Figures A1-87 and A1-88). $[\alpha]_D^{20}$ = - 14.10 (c = 0.68, CH_2Cl_2).

[L2Y(hfac)$_3$]. Yield: 90%. ^{1}H NMR (400 MHz, CD_2Cl_2): 10.44 (br, 1H), 8.19 (d, *J* = 8.4 Hz, 1H), 8.12 (d, *J* = 8.2 Hz, 1H), 7.56 – 7.31 (m, 6H), 7.20 (d, *J* = 2.2 Hz, 1H), 7.05 (d, *J* = 2.2 Hz, 1H), 5.91 (s, 3H), 4.60 – 4.37 (m, 2H), 3.20 (s, 6H), 2.27 (br, 1H), 1.66 – 1.53 (m, 1H), 1.45 – 1.26 (m, 1H), 1.10 – 0.91 (m, 6H). ^{13}C NMR (101 MHz, CD_2Cl_2): 175.86, 175.52, 175.18, 174.85, 155.81, 150.00, 149.71, 147.01, 146.25, 140.99, 139.63, 136.64, 133.00, 125.11, 124.74, 123.58, 123.47, 122.03, 121.86, 121.69, 119.18, 116.33, 113.48, 110.93, 110.38, 105.25, 102.61, 89.50, 52.09, 39.62, 35.92, 27.24, 16.46, 10.95. ESI-MS calculated for [$C_{41}H_{31}F_{18}N_6O_6Y$ - hfac]$^+$ ([[**L2**Y(hfac)$_3$]

- hfac]$^+$): *m/z* 927.1; found: 927.7. Elemental analysis calculated for $C_{41}H_{31}F_{18}N_6O_6Y$ ([**L2**Y(hfac)$_3$]) (%): C 43.40, H 2.75, N 7.41; found (%): C 43.17, H 2.76, N 7.42.

[L3Y(hfac)$_3$]. Yield: 89%. ^{1}H NMR (400 MHz, CD_2Cl_2): 8.15 (d, *J* = 8.3 Hz, 2H), 7.52 (d, *J* = 8.3 Hz, 2H), 7.43 (ddd, *J* = 8.3, 7.0, 1.1 Hz, 2H), 7.32 (ddd, *J* = 8.3, 7.1, 1.2 Hz, 2H), 7.25 (s, 2H), 5.88 (s, 3H), 4.60 – 4.40 (m, 4H), 3.32 (s, 6H), 2.32 – 2.19 (m, 2H), 1.57 – 1.49 (m, 2H), 1.41 – 1.25 (m, 2H), 1.02 – 0.93 (m, 12H). ^{13}C NMR (101 MHz, CD_2Cl_2): 175.57, 175.23, 174.89, 174.56, 155.76, 150.47, 150.46, 147.67, 139.91, 136.37, 124.51, 123.29, 122.21, 122.04, 119.19, 116.34, 113.48, 110.40, 105.52, 89.27, 52.04, 39.92, 35.84, 27.19, 16.52, 10.95. ESI-MS calculated for [$C_{46}H_{41}F_{18}N_6O_6Y$ - hfac]$^+$ ([[**L3**Y(hfac)$_3$] - hfac]$^+$): *m/z* 997.2; found: 997.7. Elemental analysis calculated for $C_{46}H_{41}F_{18}N_6O_6Y$ ([**L3**Y(hfac)$_3$]) (%): C 45.86, H 3.43, N 6.98; found (%): C 45.89, H 3.27, N 6.87.

[L5Y(hfac)$_3$]. Yield: 36%. ^{1}H NMR (400 MHz, CD_2Cl_2): 8.17 (d, *J* = 8.0 Hz, 1H), 8.11 (d, *J* = 8.4 Hz, 1H), 7.85 (br, 1H), 7.63 (br, 2H), 7.56 – 7.29 (m, 6H), 5.92 (br, 3H), 4.69 – 4.29 (m, 4H), 3.15 (br, 2H), 2.87 (s, 4H), 2.16 (s, 1H), 1.63 – 1.49 (m, 1H), 1.45 – 1.32 (m, 1H), 1.20 (br, 6H), 1.05 – 0.98 (m, 6H). ^{13}C NMR (101 MHz, CD_2Cl_2): 175.47, 167.33, 148.48, 141.29, 139.65, 136.67, 125.12, 123.68, 123.65, 122.04, 119.18, 116.33, 113.48, 111.32, 110.44, 106.04, 89.55, 67.26, 51.66, 50.80, 47.76, 36.00, 27.17, 16.63, 10.92, 10.35. ESI-MS calculated for [$C_{45}H_{39}F_{18}N_6O_7Y$ - hfac]$^+$ ([[**L5**Y(hfac)$_3$] - hfac]$^+$): *m/z* 999.2; found: 999.6. Elemental analysis calculated for $C_{45}H_{39}F_{18}N_6O_7Y$ ([**L5**Y(hfac)$_3$]) (%): C 44.79, H 3.26, N 6.96; found (%): C 43.80, H 3.16, N 6.62.

[L7Y(hfac)$_3$]. Yield: 70%. ^{1}H NMR (400 MHz, CD_2Cl_2): 9.78 (s, 2H), 8.09 (d, *J* = 8.3 Hz, 2H), 7.62 (d, *J* = 8.1 Hz, 2H), 7.54 (d, *J* = 7.2 Hz, 2H), 7.51 – 7.25 (m, 12H), 7.19 (s, 2H), 5.94 (s, 3H), 4.19 (t, *J* = 6.3 Hz, 2H), 4.12 (q, *J* = 6.8 Hz, 1H), 3.93 – 3.77 (m, 2H), 3.18 – 2.97 (m, 2H), 1.55 (d, *J* = 6.8 Hz, 3H). ^{13}C NMR (101 MHz, CD_2Cl_2): 176.01, 175.68, 175.34, 175.00, 168.06, 148.02,

147.27, 143.38, 141.06, 140.58, 132.89, 128.58, 128.39, 128.23, 127.88, 127.01, 125.76, 124.02, 122.01, 121.76, 119.16, 116.31, 113.46, 111.03, 107.23, 89.68, 69.00, 59.73, 56.08, 48.79, 14.66. ESI-MS calculated for [$C_{51}H_{35}N_6O_7F_{18}Y$ - hfac]$^+$ ([[**L7**Y(hfac)$_3$] - hfac]$^+$): *m/z* 997.2; found: 997.7. Elemental analysis calculated for $C_{51}H_{35}N_6O_7F_{18}Y$ ([**L7**Y(hfac)$_3$]) (%): C 48.05, H 2.77, N 6.59; found (%): C 47.95, H 2.66, N 6.60.

[L8Y(hfac)$_3$]. Yield: 31%. ^{1}H NMR (400 MHz, CD_2Cl_2): 8.15 (dd, *J* = 8.4, 1.1 Hz, 2H), 7.57 – 7.13 (m, 18H), 5.89 (s, 3H), 4.60 – 4.47 (m, 4H), 4.17 – 4.06 (m, 3H), 3.88 – 3.74 (m, 2H), 3.25 – 3.12 (m, 1H), 3.09 – 3.01 (m, 1H), 2.02 – 1.93 (m, 4H), 1.92 – 1.83 (m, 2H), 1.55 (d, *J* = 6.9 Hz, 3H), 1.12 (d, *J* = 6.5 Hz, 12H). ^{13}C NMR (101 MHz, CD_2Cl_2): 175.42, 175.09, 167.69, 148.91, 148.73, 143.16, 140.32, 140.12, 135.75, 128.45, 128.28, 128.15, 127.77, 127.05, 127.02, 125.08, 123.73, 122.31, 119.14, 116.29, 113.44, 109.69, 109.31, 89.46, 69.12, 59.76, 56.12, 48.86, 44.63, 38.35, 26.57, 22.19, 14.77. ESI-MS calculated for [$C_{61}H_{55}N_6O_7F_{18}Y$ - hfac]$^+$ ([[**L8**Y(hfac)$_3$] - hfac]$^+$): *m/z* 1207.3; found: 1207.8. Elemental analysis calculated for $C_{61}H_{55}N_6O_7F_{18}Y$ ([**L8**Y(hfac)$_3$]) (%): C 51.78, H 3.92, N 5.94; found (%): C 51.74, H 3.72, N 5.83.

General procedure for the Michael reaction of diethyl malonate to β-nitrostyrene.

A mixture of β-nitrostyrene (1eq.), diethyl malonate (2eq.) and catalyst (10 mol%) in CH_2Cl_2 (0.25 M) was stirred at r.t for 24 h and then evaporated to dryness. The residue was purified by by column chromatography (SiO_2, hexane/Et_2O 100/0 to 80/20) to afford **3** as a white solid. Slow evaporation of hexane/dichloromethane solution provided a single structure of **3**. ^{1}H NMR (400 MHz, CD_2Cl_2): 7.44 – 7.25 (m, 5H), 5.00 – 4.82 (m, 2H), 4.29 – 4.20 (m, 3H), 4.02 (q, *J* = 7.1 Hz, 2H), 3.83 (d, *J* = 9.5 Hz, 1H), 1.29 (t, *J* = 7.2 Hz, 3H), 1.07 (t, *J* = 7.1 Hz, 3H). ^{13}C NMR (101 MHz, CD_2Cl_2): 167.40, 166.69, 136.40, 128.83, 128.27, 128.06, 77.82, 62.19, 61.86, 54.97, 43.03, 13.74, 13.51.

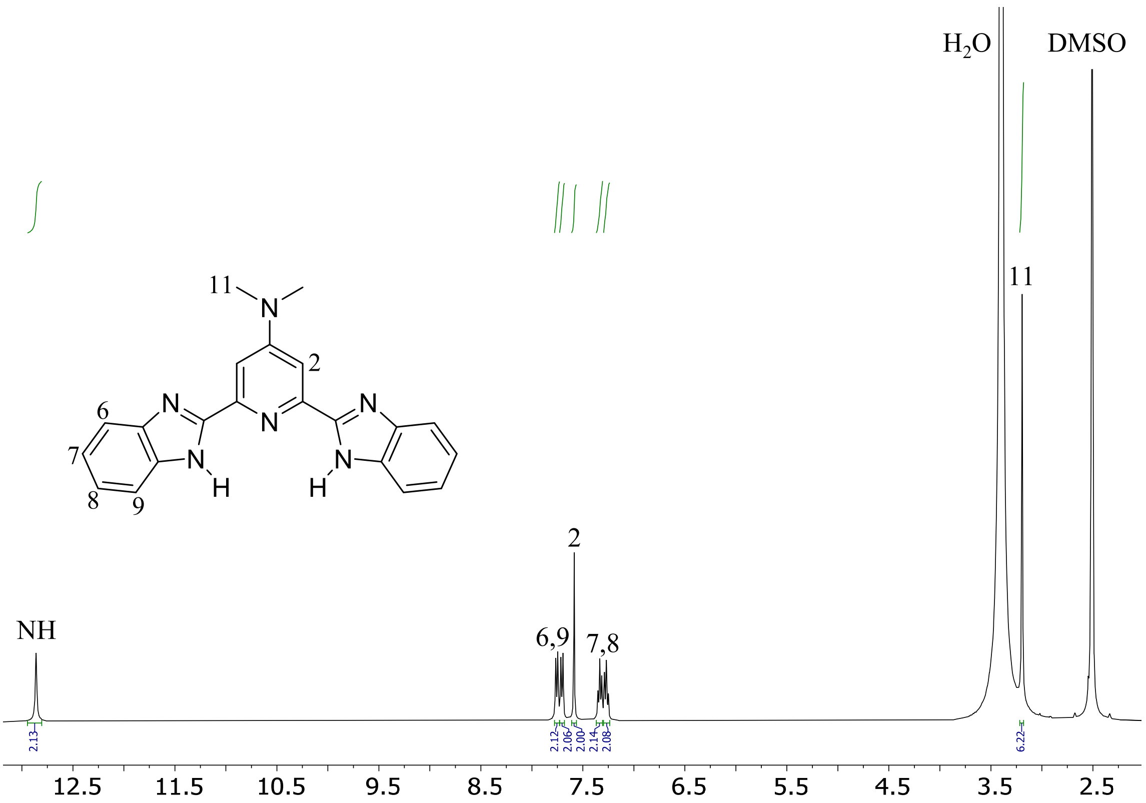

Figure A1-1. ^{1}H NMR spectrum of ligand **L1** in DMSO-*d6*.

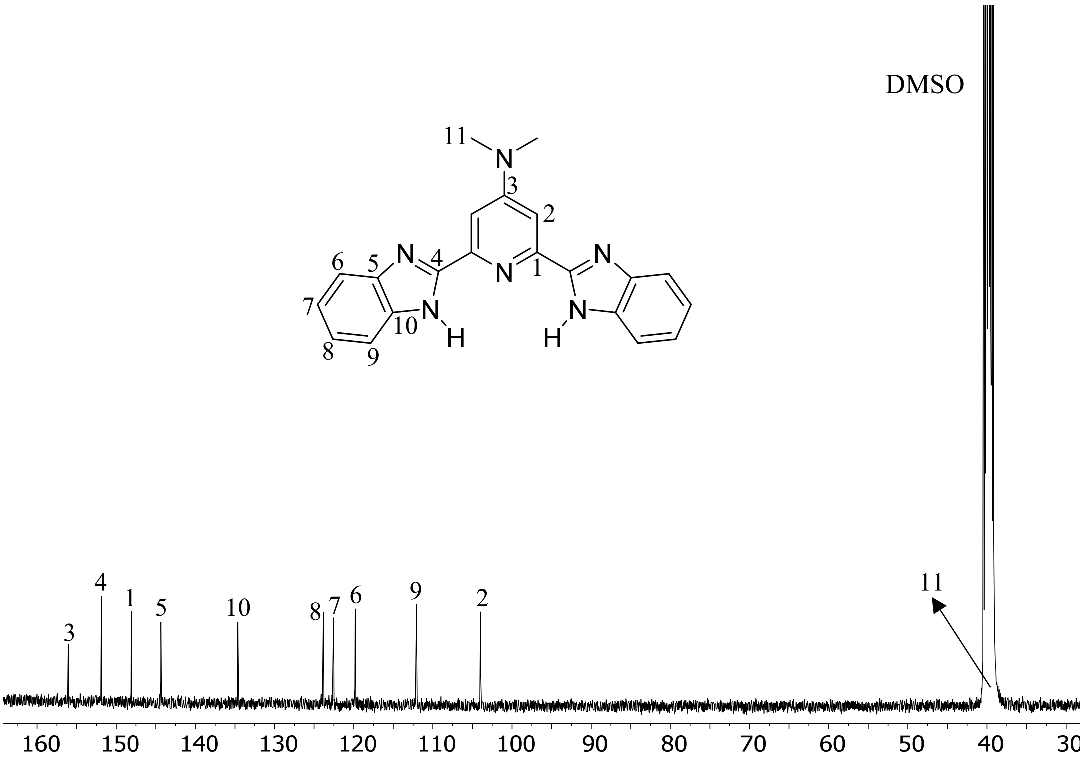

Figure A1-2. ^{13}C NMR spectrum of ligand **L1** in DMSO-*d6*.

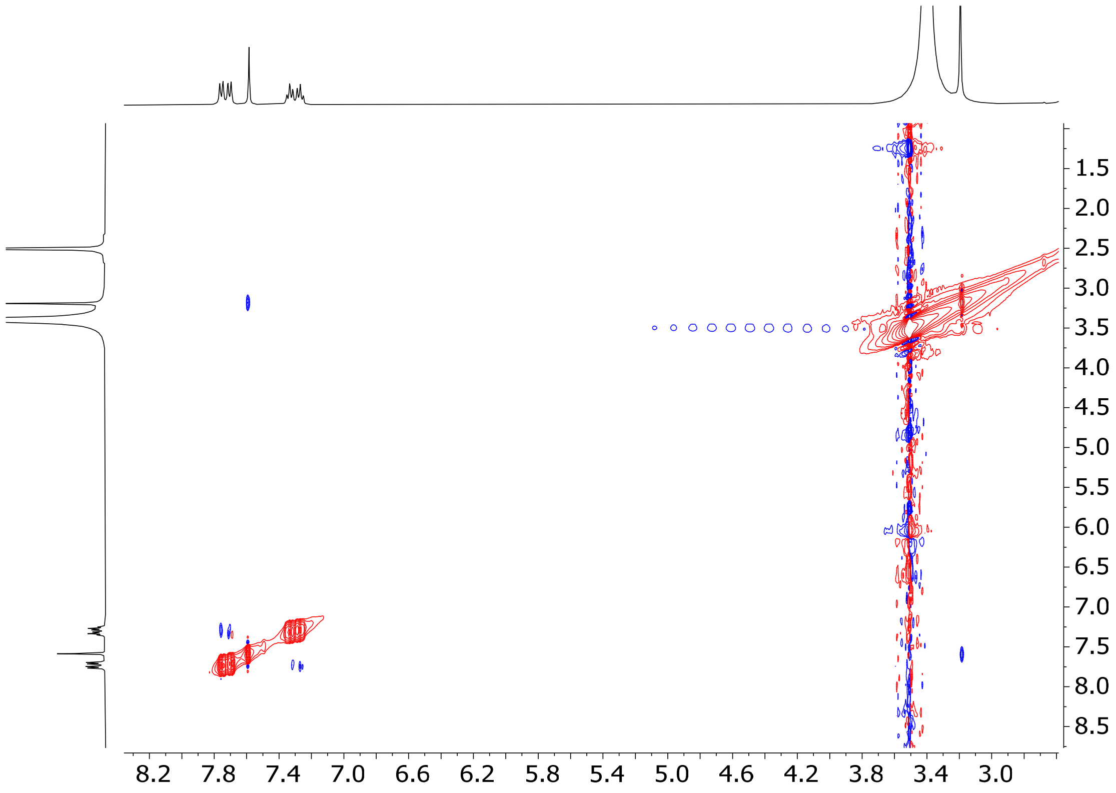

Figure A1-3. NOESY spectrum of ligand **L1** in DMSO-*d6*.

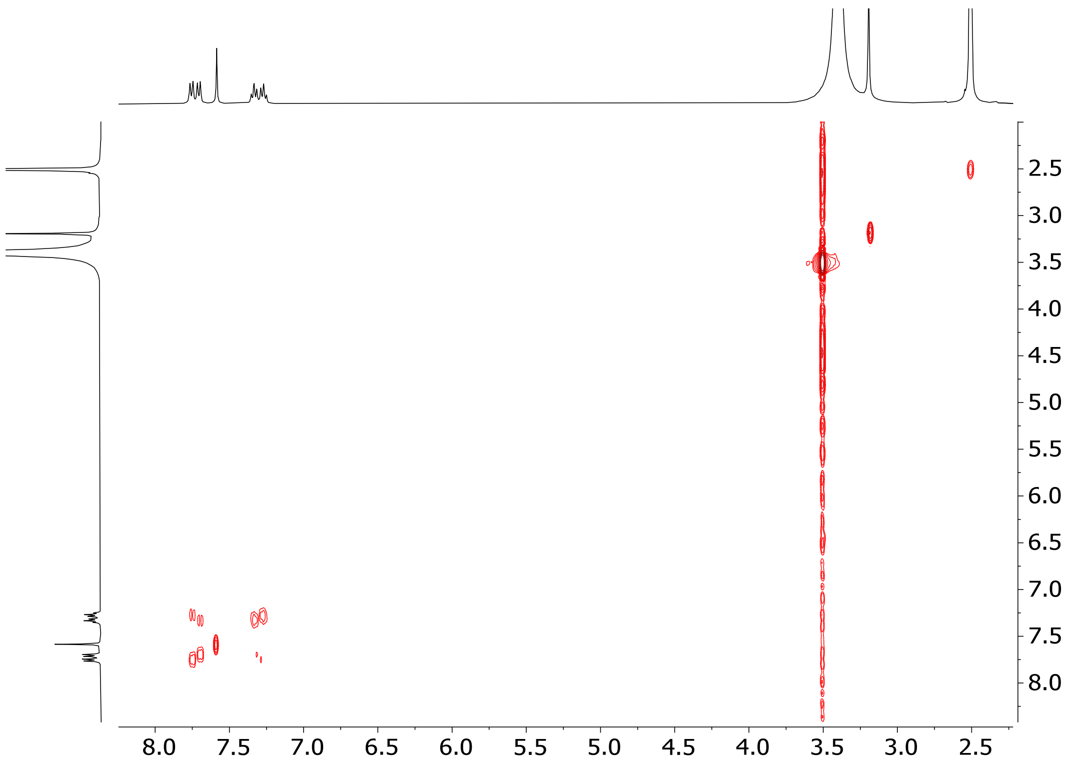

Figure A1-4. COSY spectrum of ligand **L1** in DMSO-*d6*.

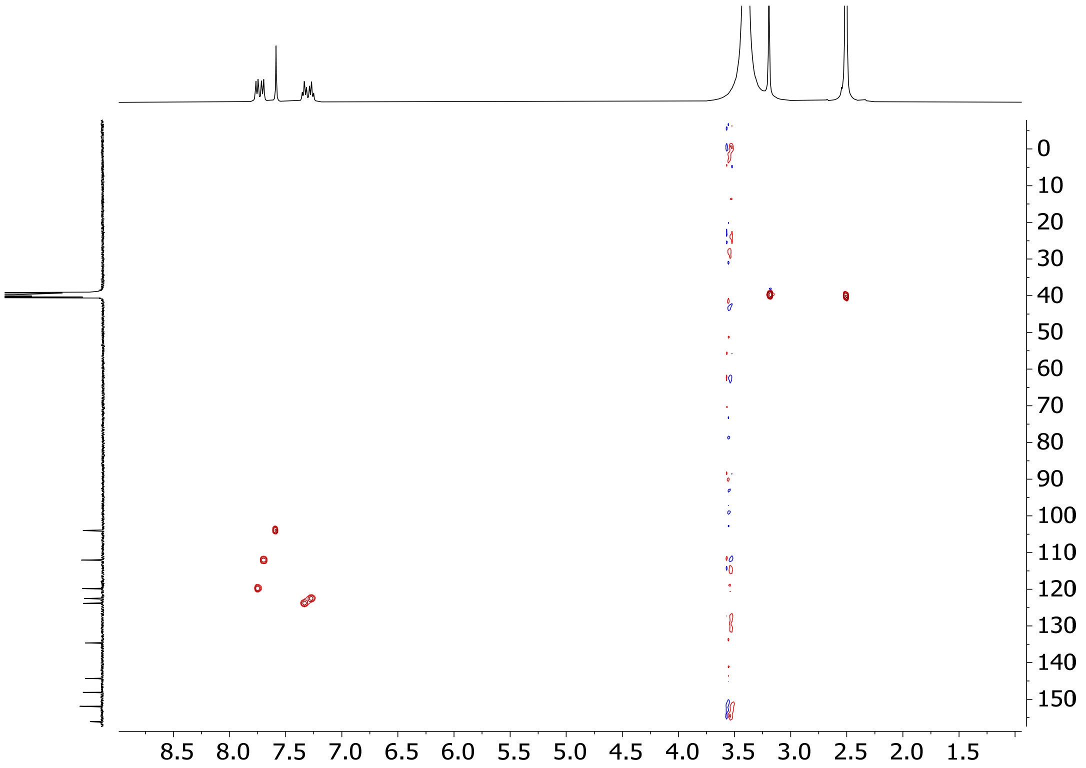

Figure A1-5. HSQC spectrum of ligand **L1** in DMSO-*d6*.

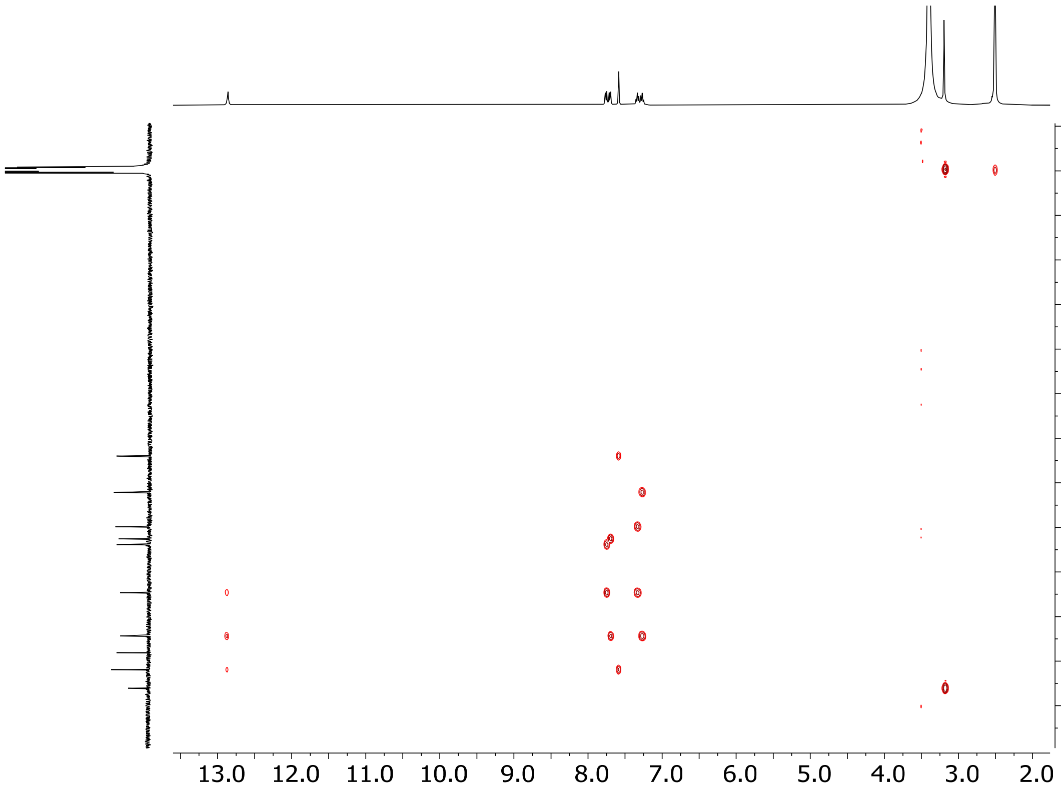

Figure A1-6. HMBC spectrum of ligand **L1** in DMSO-*d6*.

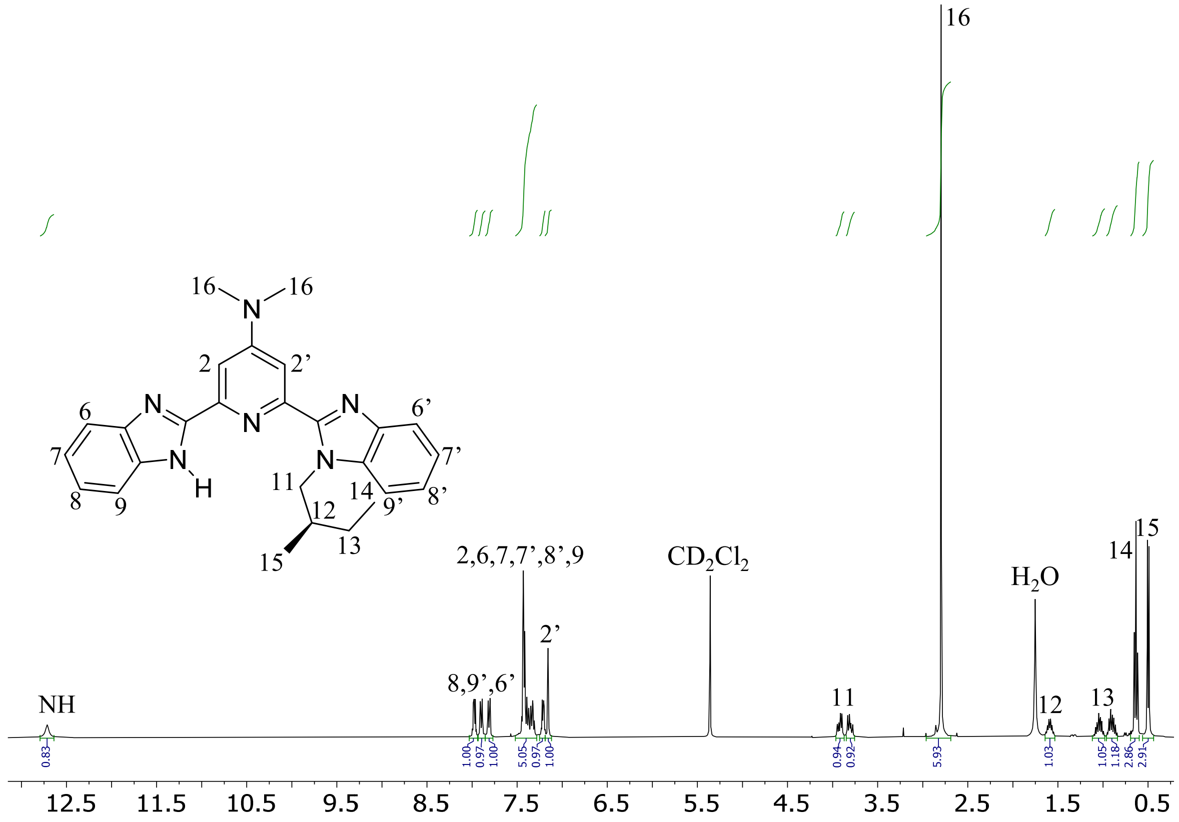

Figure A1-7. ^{1}H NMR spectrum of **L2** in CD_2Cl_2.

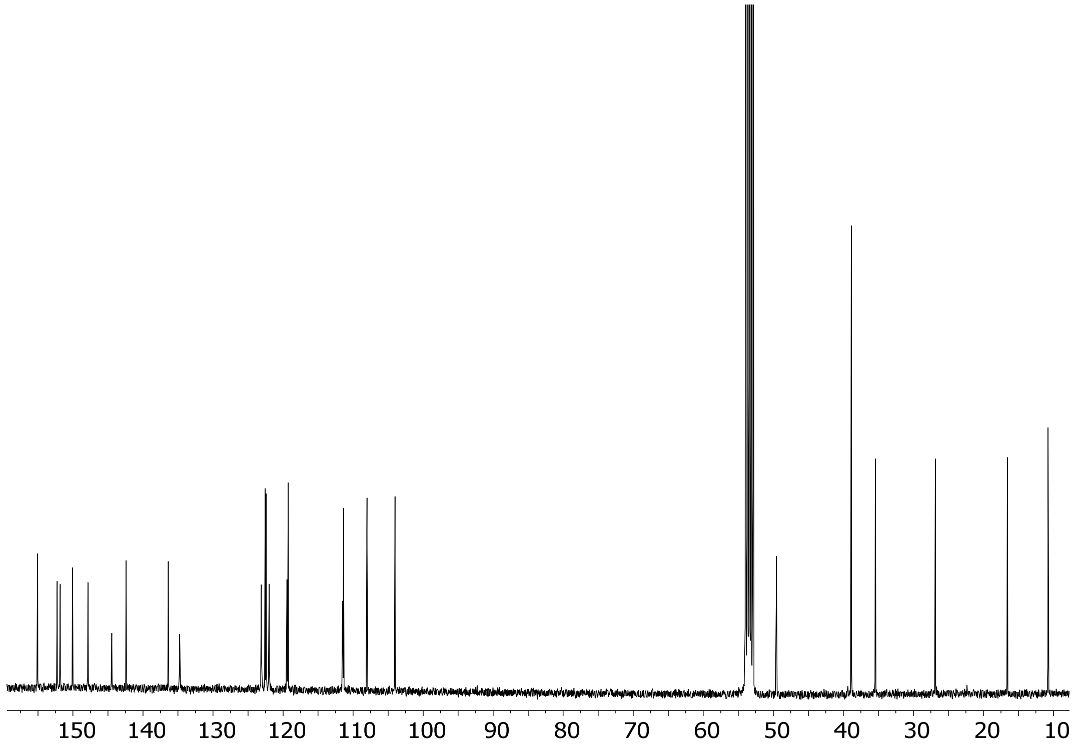

Figure A1-8. ^{13}C NMR spectrum of **L2** in CD_2Cl_2.

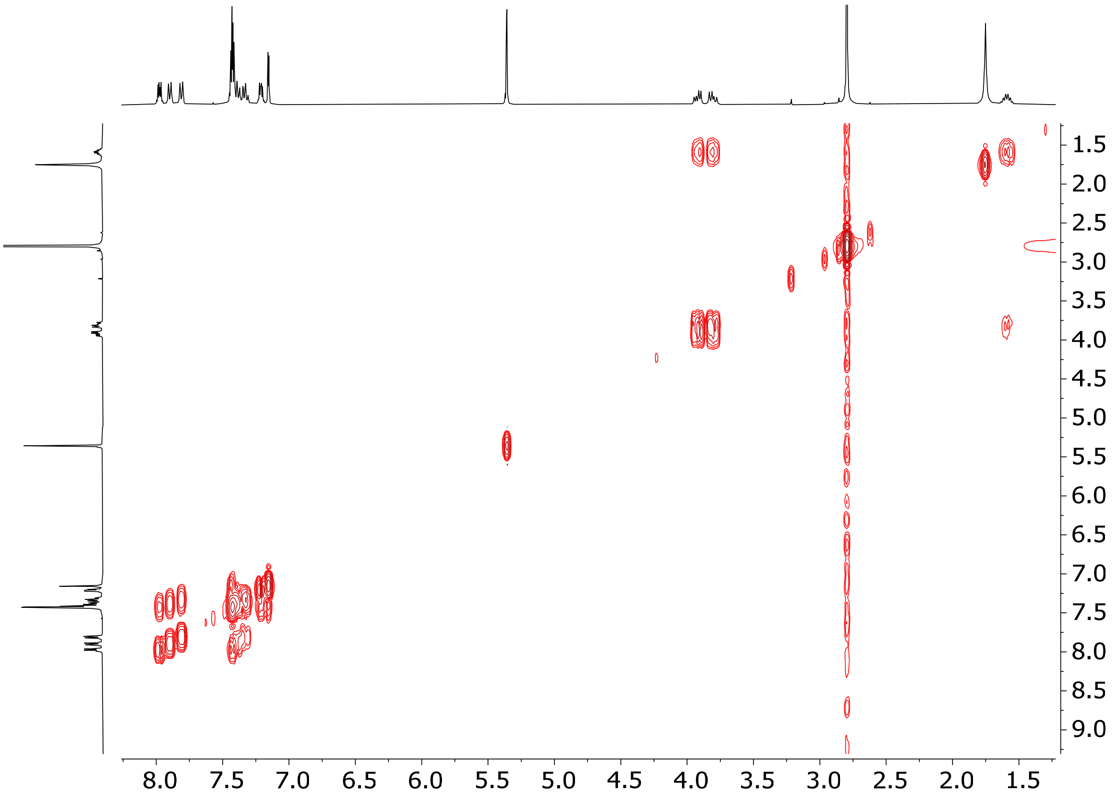

Figure A1-9. COSY spectrum of **L2** in CD_2Cl_2.

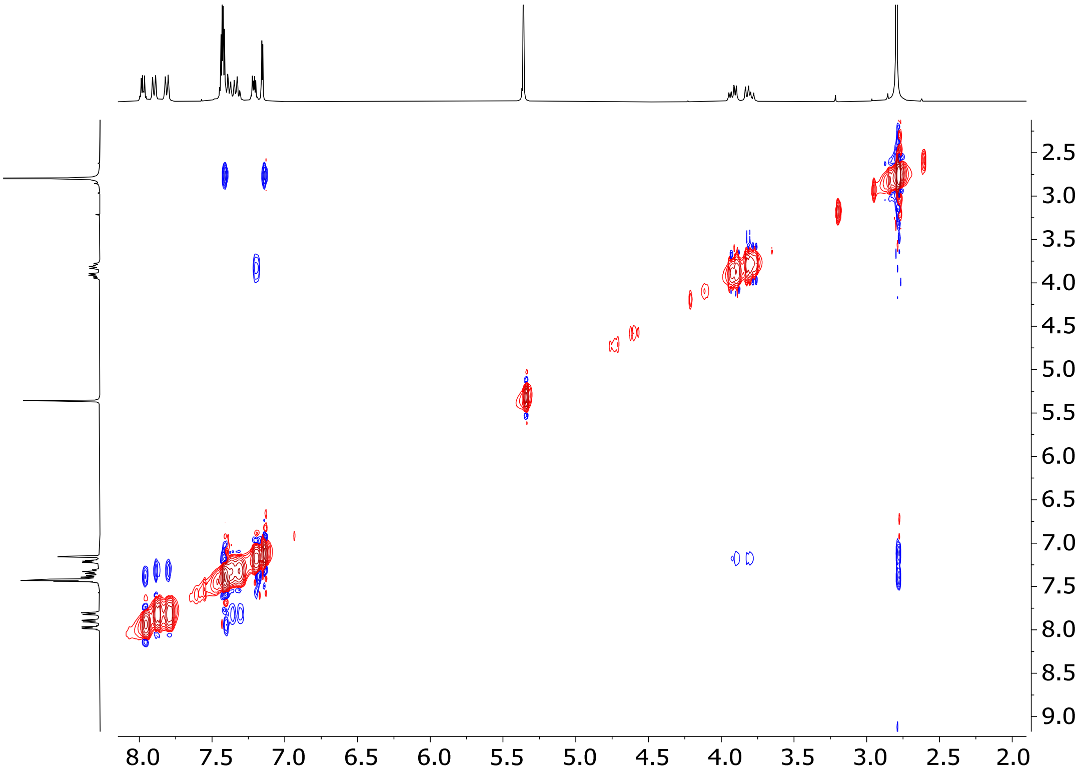

Figure A1-10. NOESY spectrum of **L2** in CD_2Cl_2.

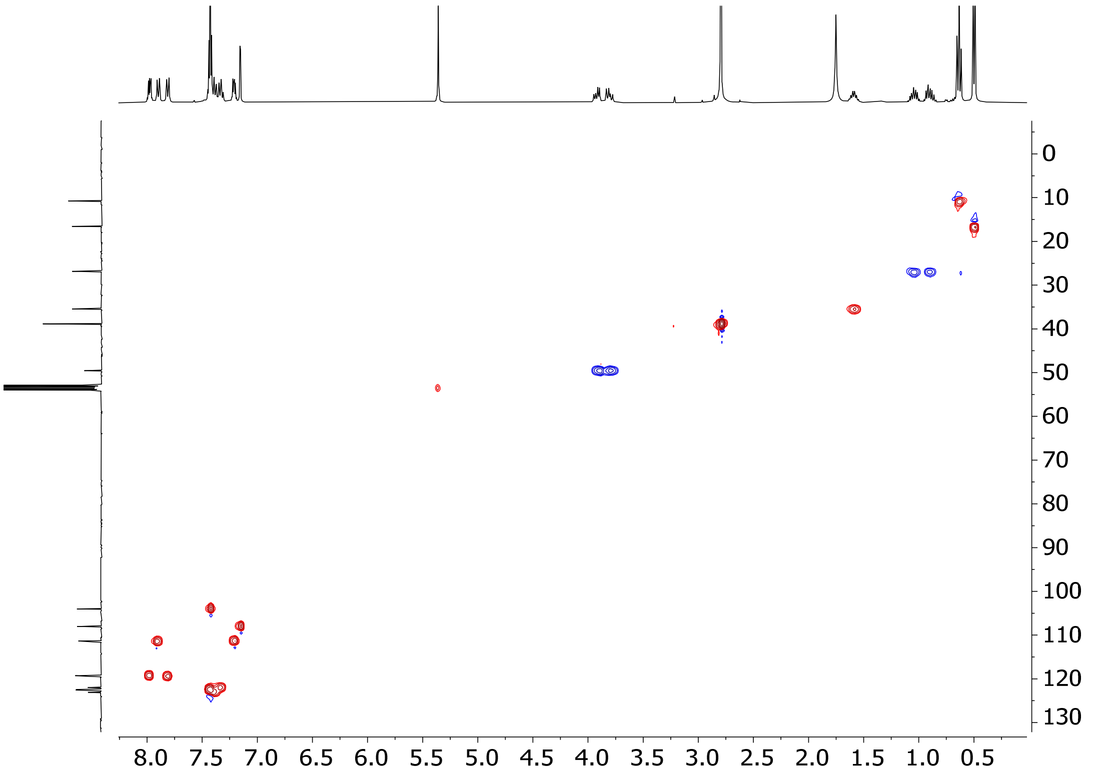

Figure A1-11. HSQC spectrum of **L2** in CD_2Cl_2.

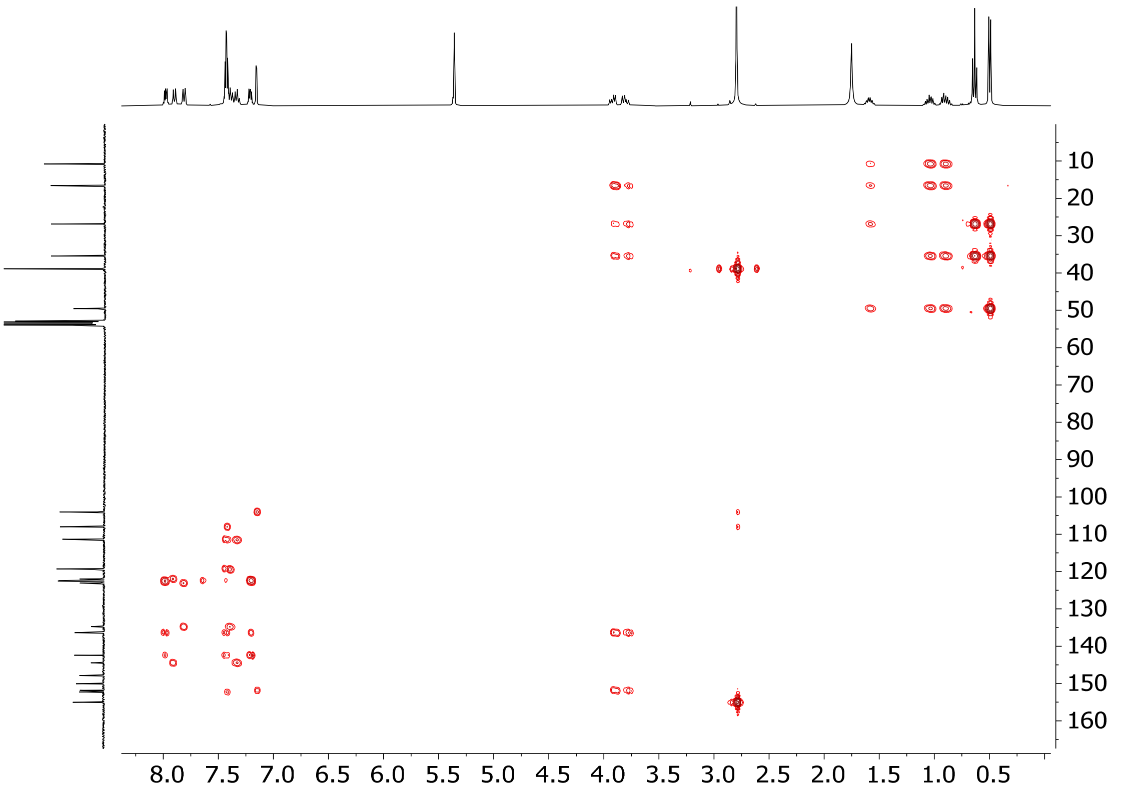

Figure A1-12. HMBC spectrum of **L2** in CD_2Cl_2.

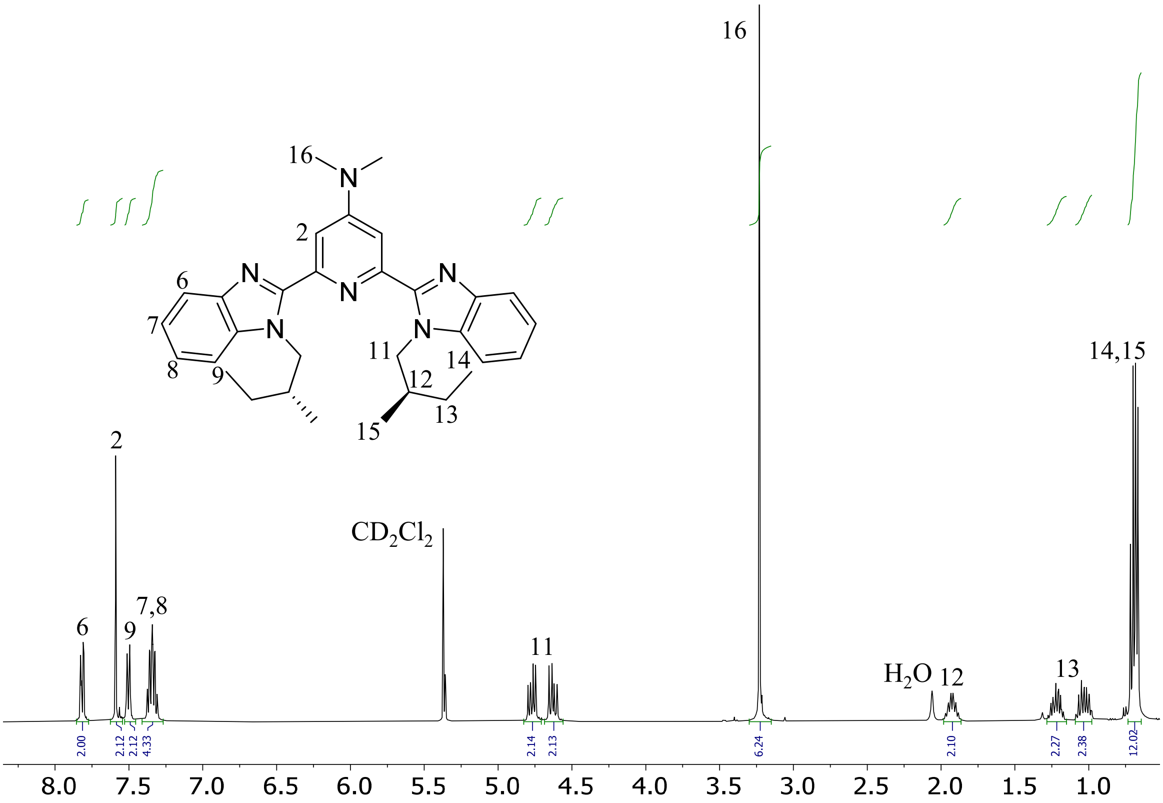

Figure A1-13. ^{1}H NMR spectrum of **L3** in CD_2Cl_2.

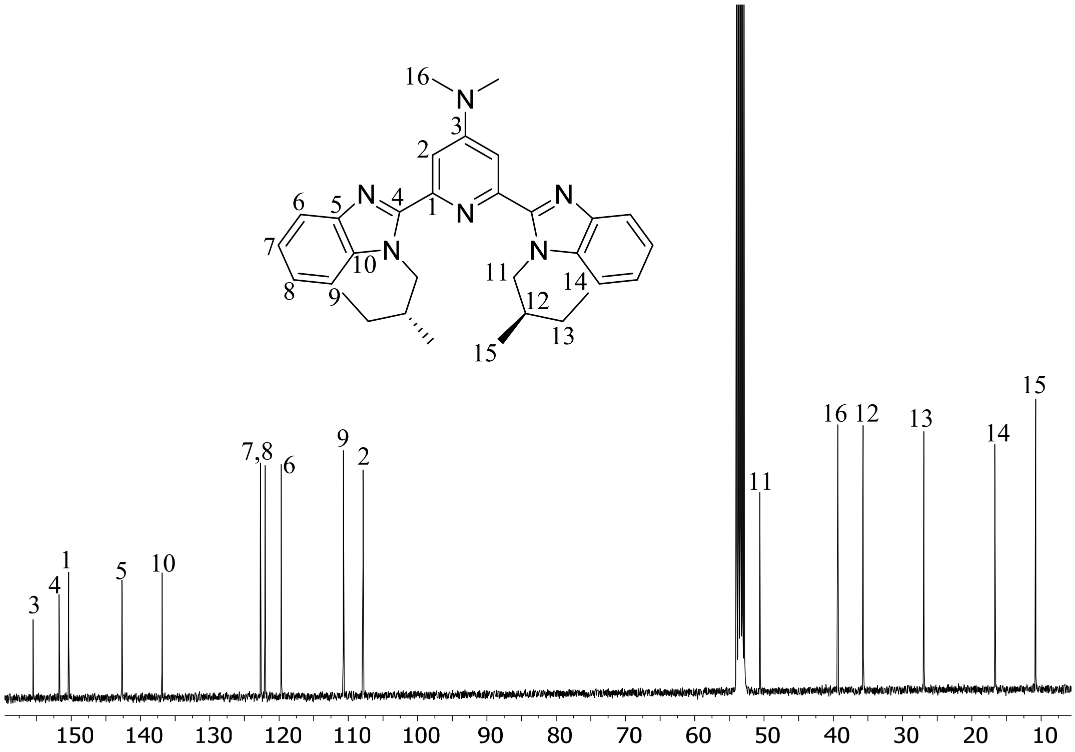

Figure A1-14. ^{13}C NMR spectrum of **L3** in CD_2Cl_2.

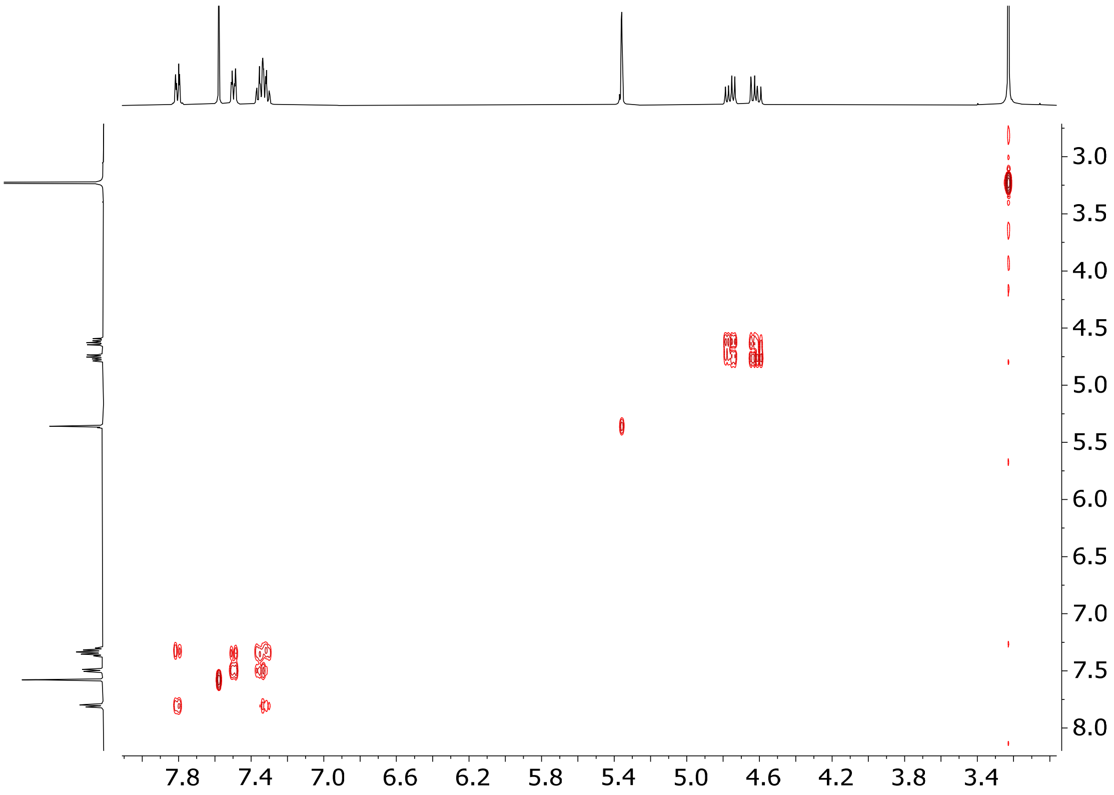

Figure A1-15. COSY spectrum of **L3** in CD_2Cl_2.

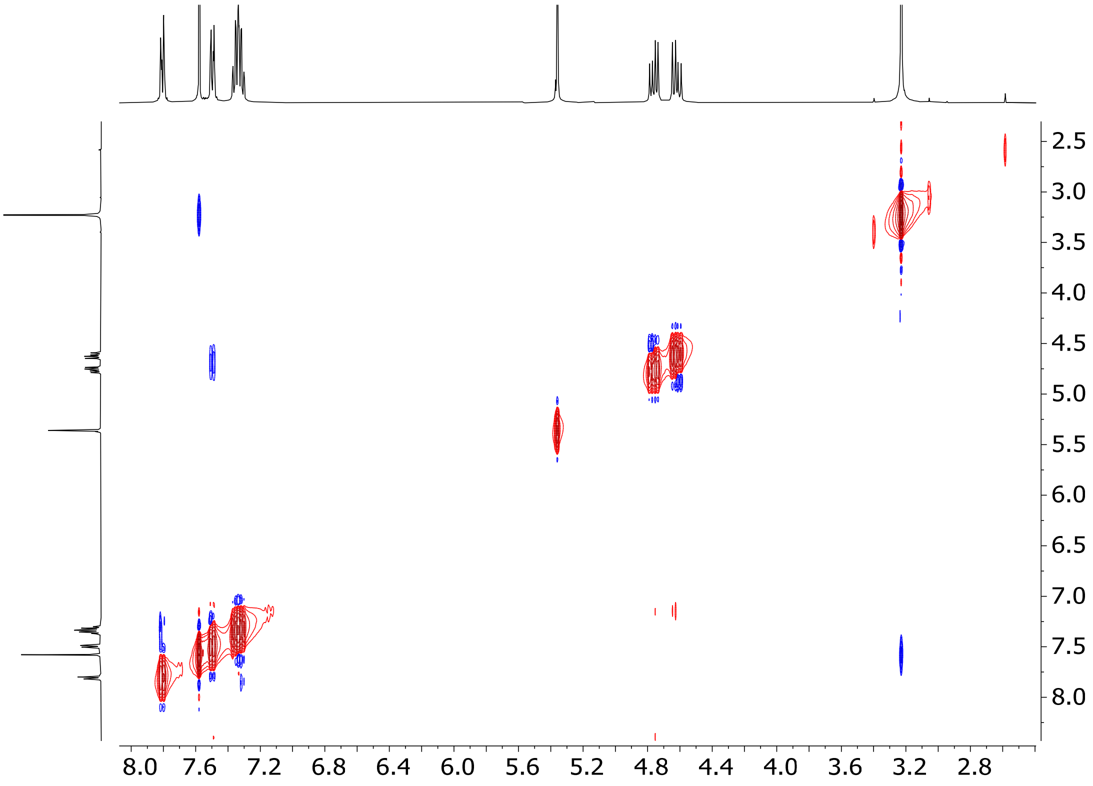

Figure A1-16. NOESY spectrum of **L3** in CD_2Cl_2.

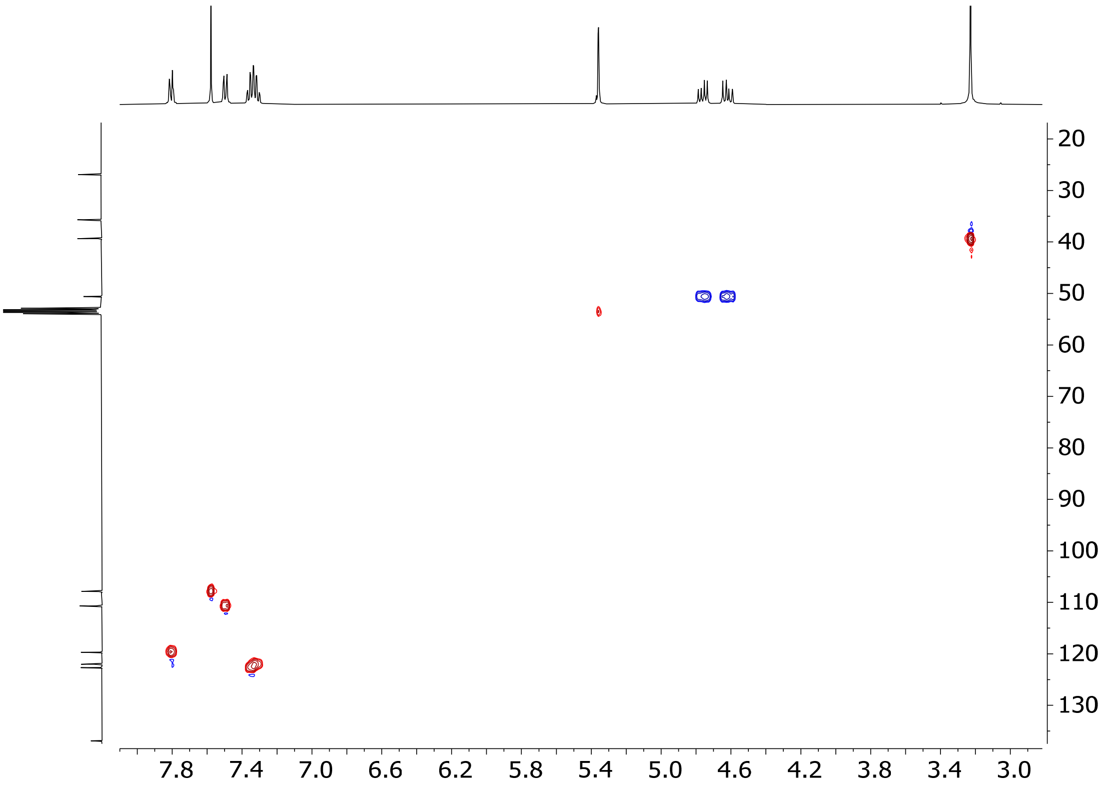

Figure A1-17. HSQC spectrum of **L3** in CD_2Cl_2.

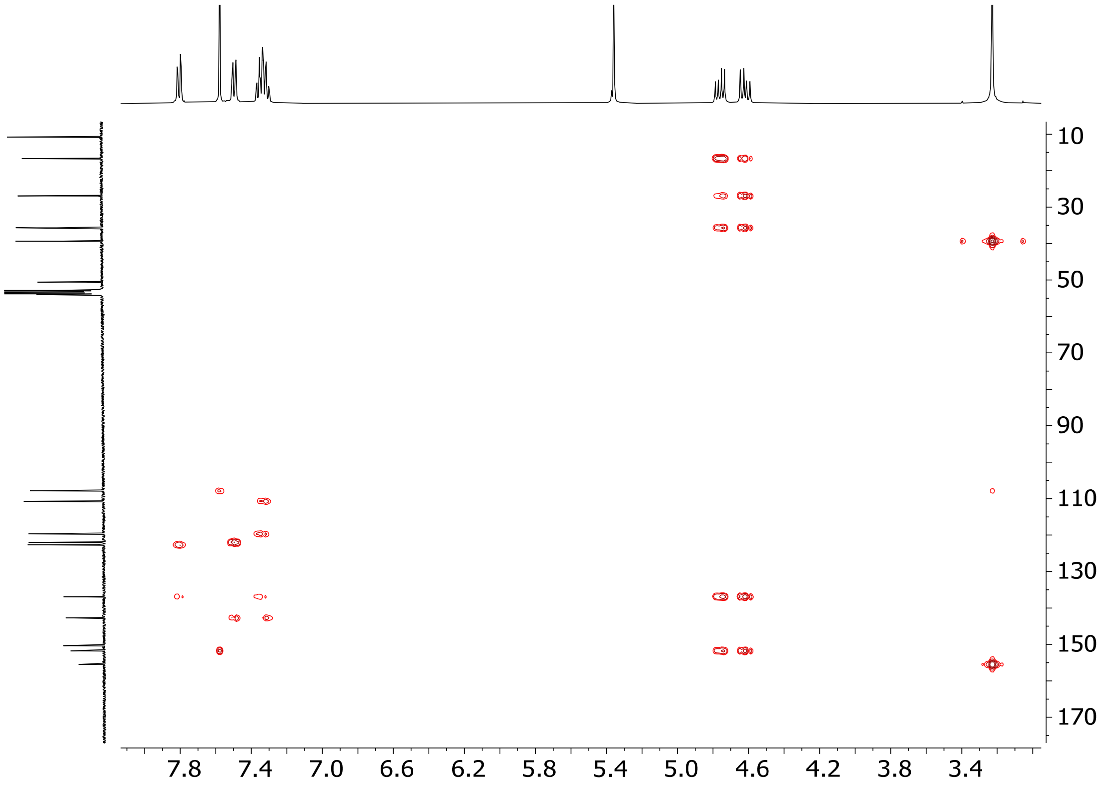

Figure A1-18. HMBC spectrum of **L3** in CD_2Cl_2.

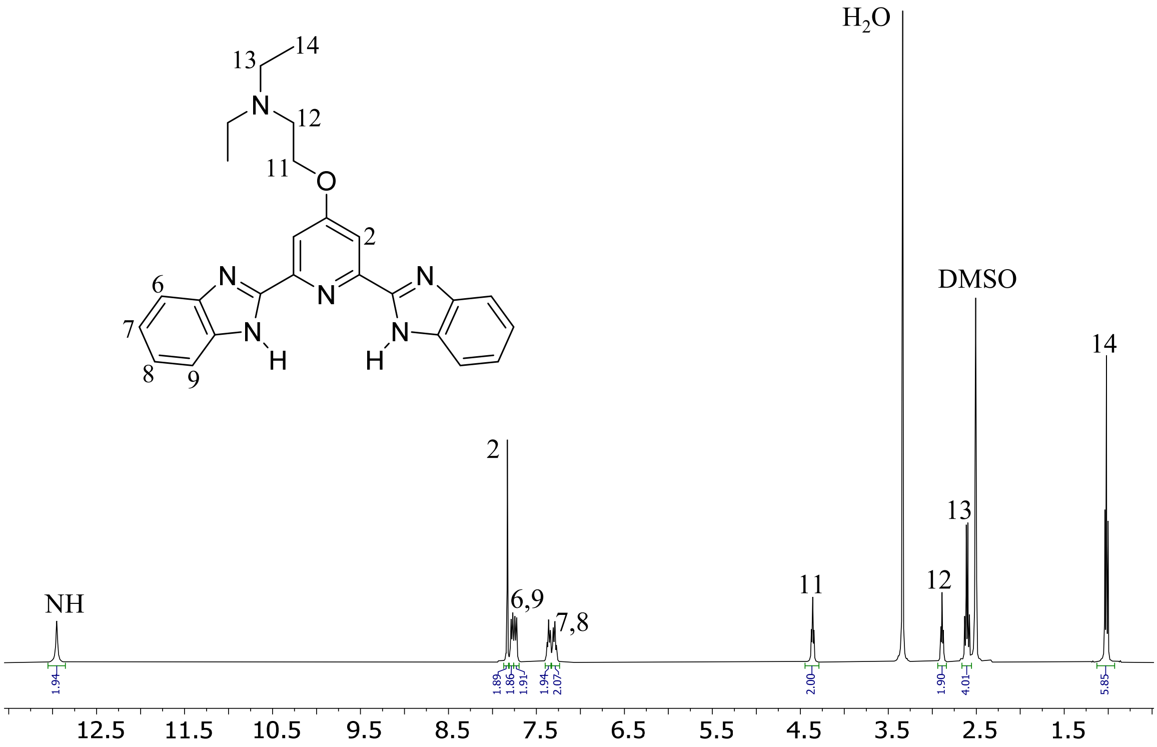

Figure A1-19. ^{1}H NMR spectrum of **L4** in DMSO-*d6*.

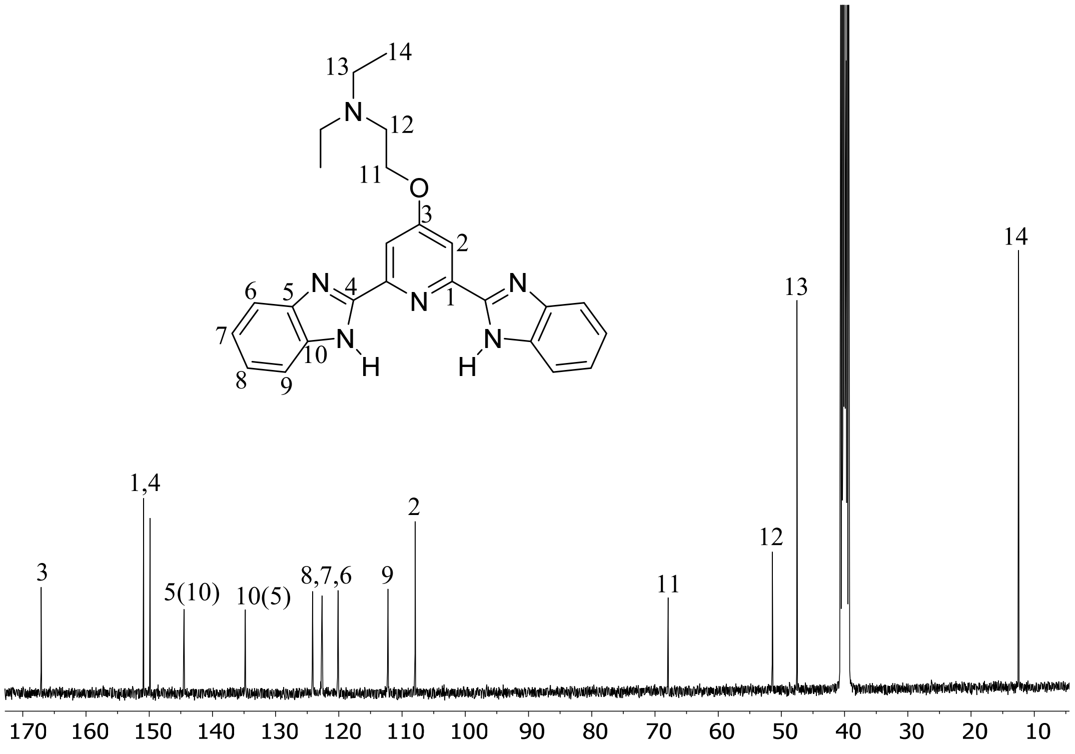

Figure A1-20. ^{13}C NMR spectrum of **L4** in DMSO-*d6*.

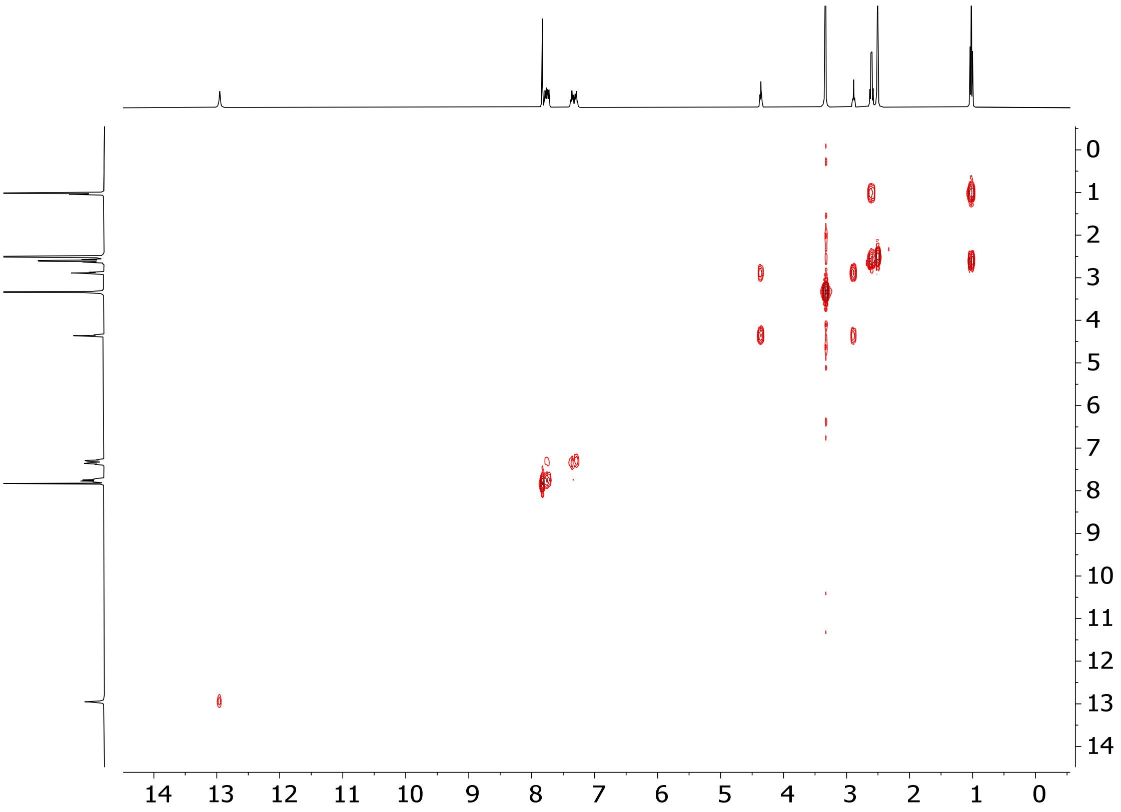

Figure A1-21. COSY spectrum of **L4** in DMSO-*d6*.

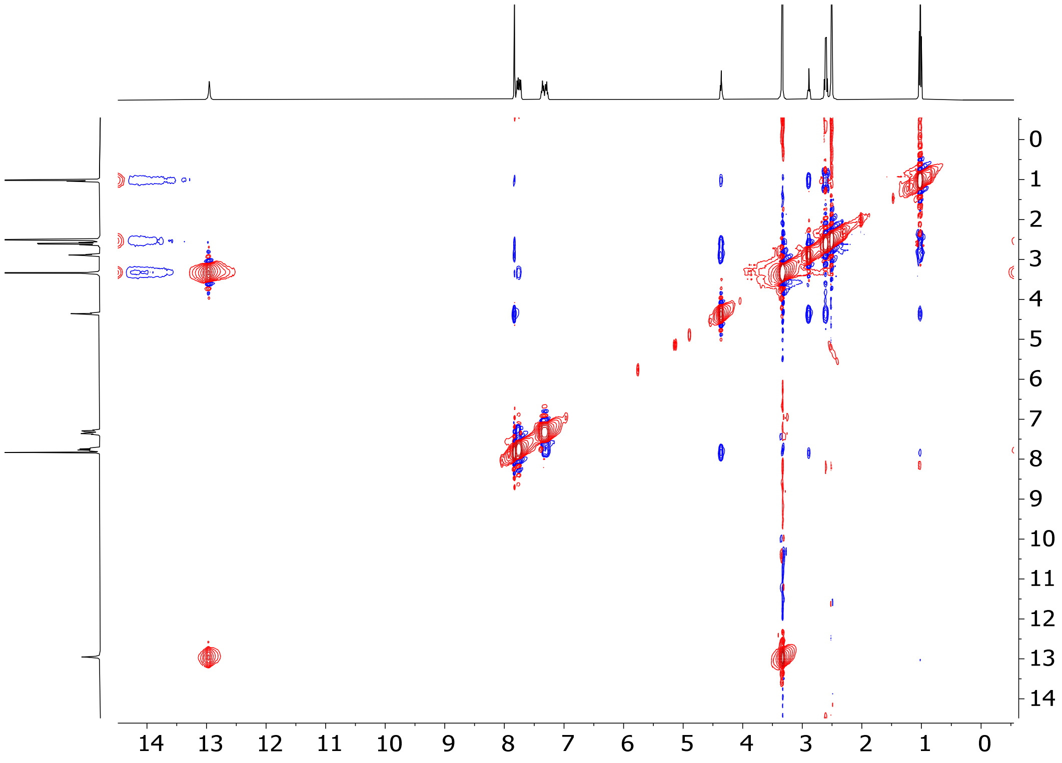

Figure A1-22. NOESY spectrum of **L4** in DMSO-*d6*.

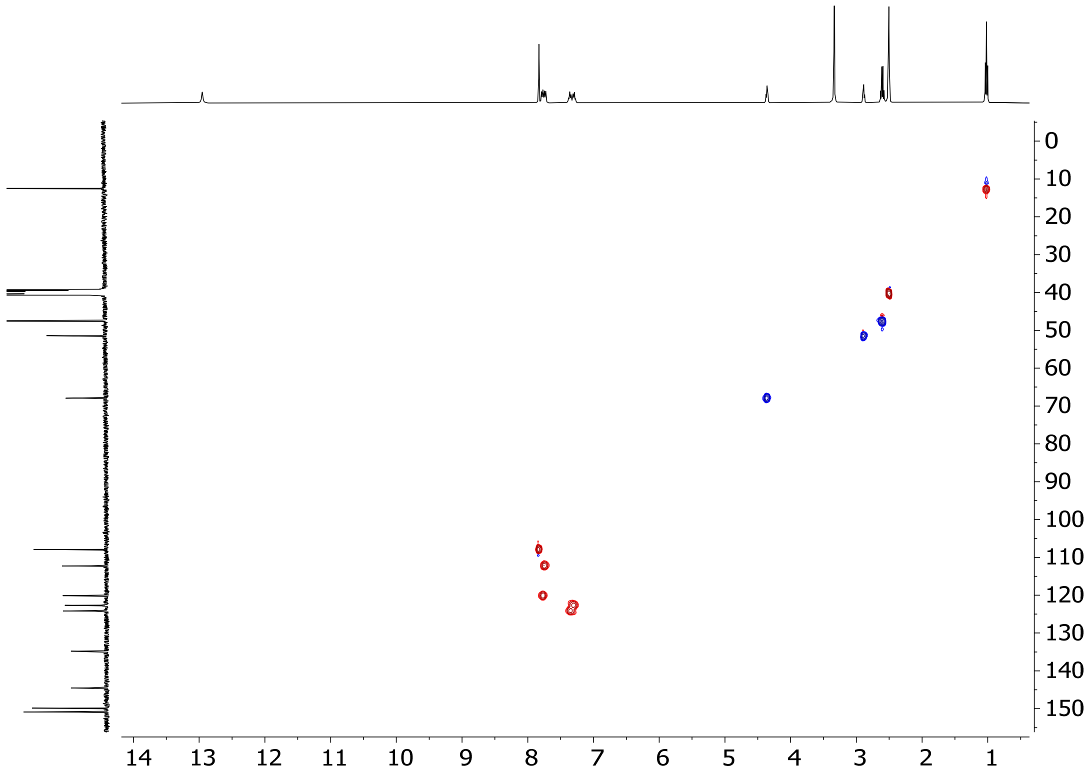

Figure A1-23. HSQC spectrum of **L4** in DMSO-*d6*.

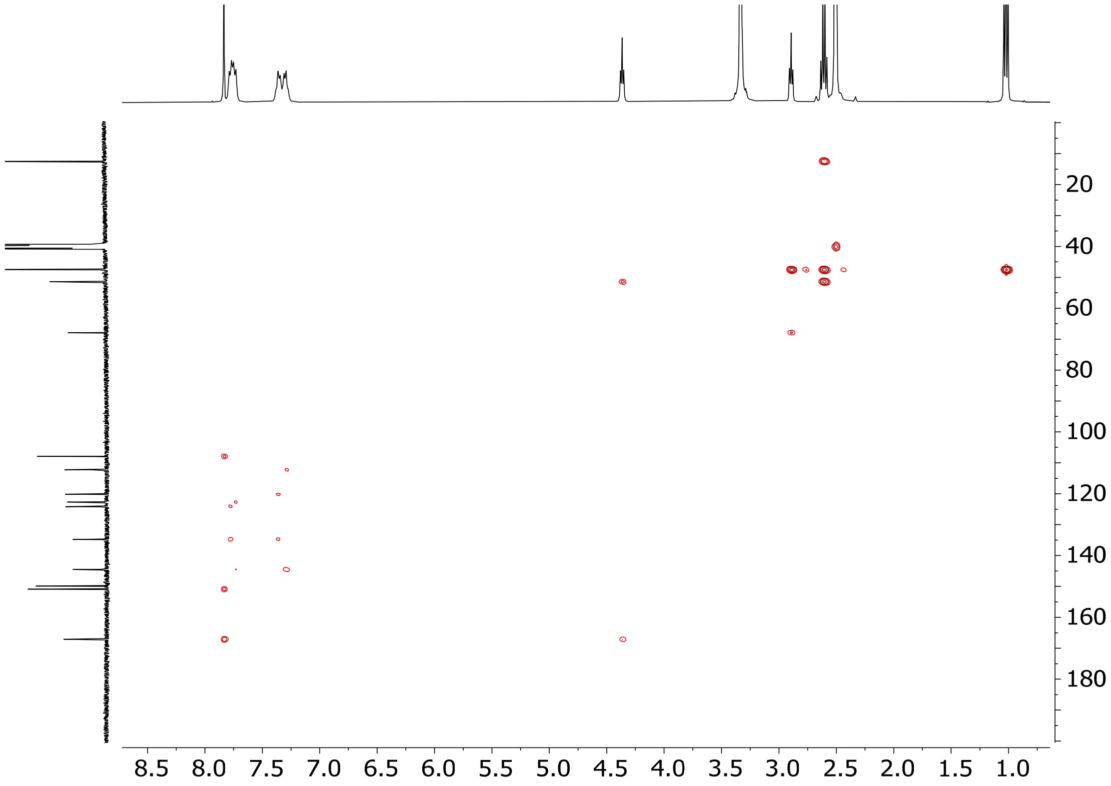

Figure A1-24. HMBC spectrum of **L4** in DMSO-*d6*.

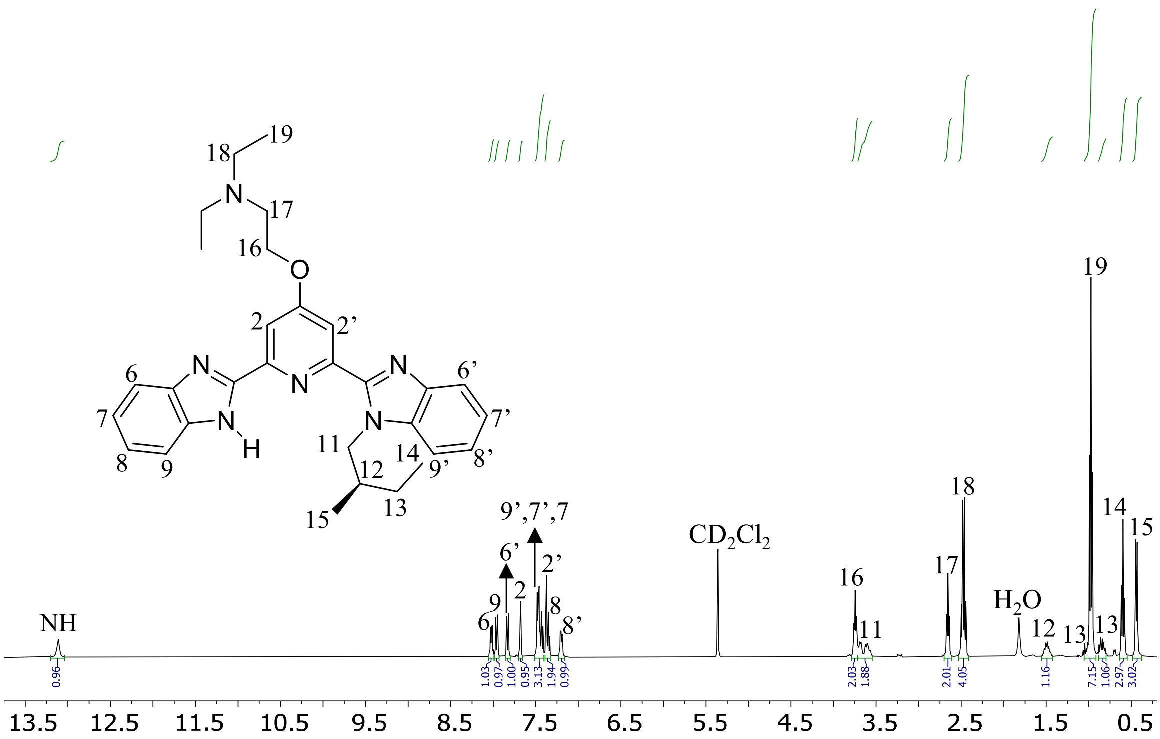

Figure A1-25. ^{1}H NMR spectrum of **L5** in CD_2Cl_2.

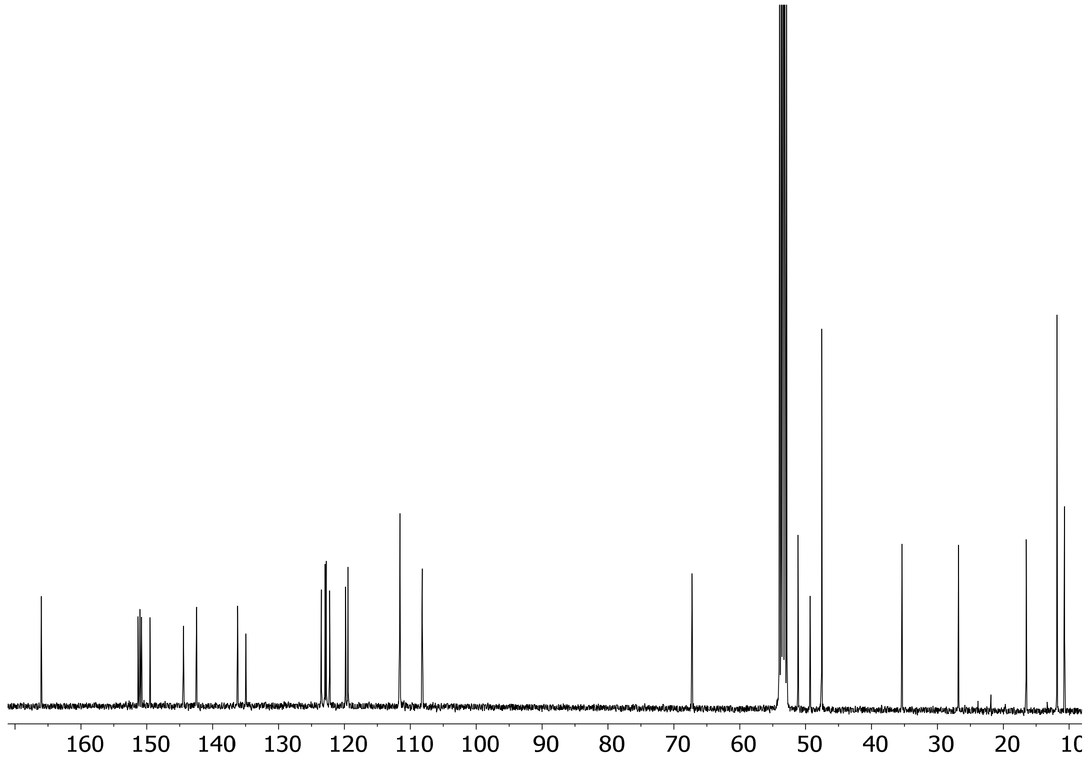

Figure A1-26. ^{13}C NMR spectrum of **L5** in CD_2Cl_2.

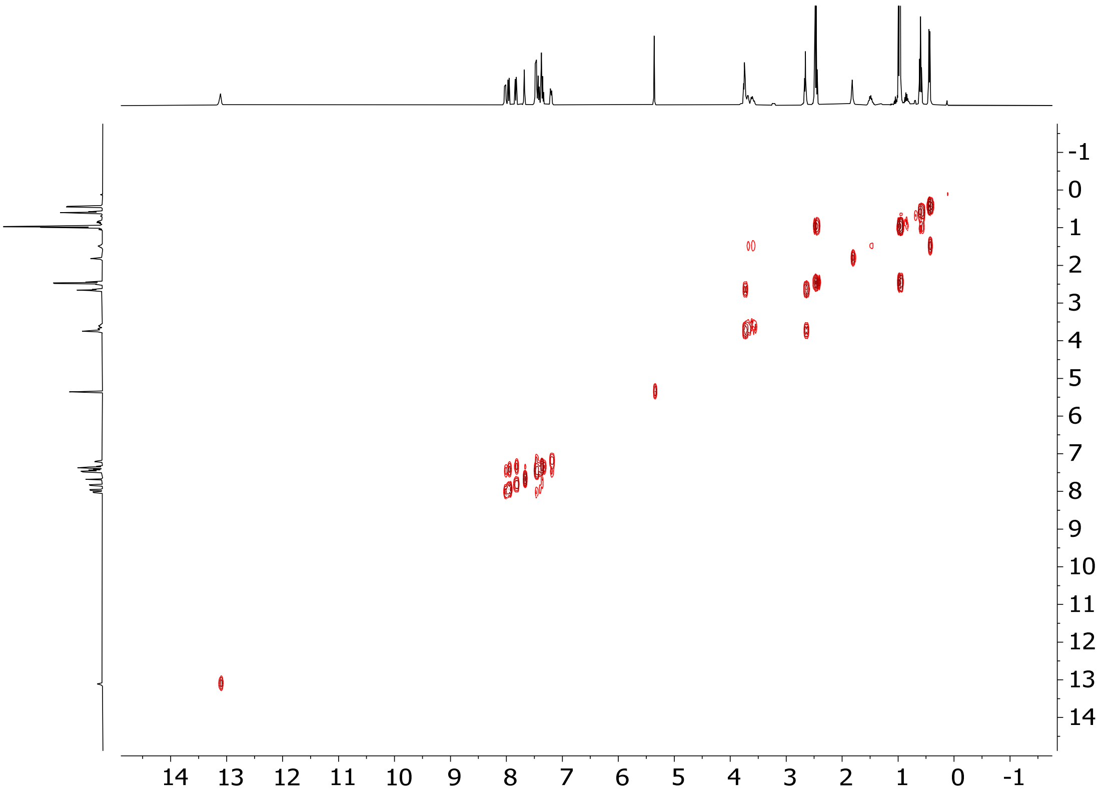

Figure A1-27. COSY spectrum of **L5** in CD_2Cl_2.

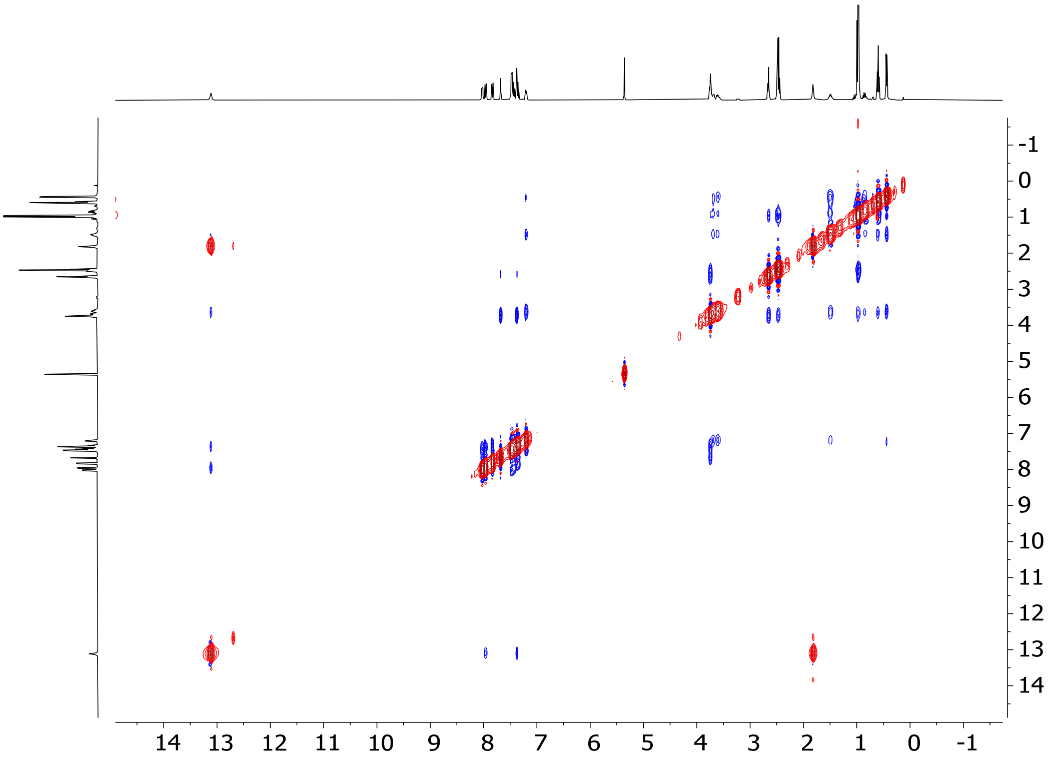

Figure A1-28. NOESY spectrum of **L5** in CD_2Cl_2.

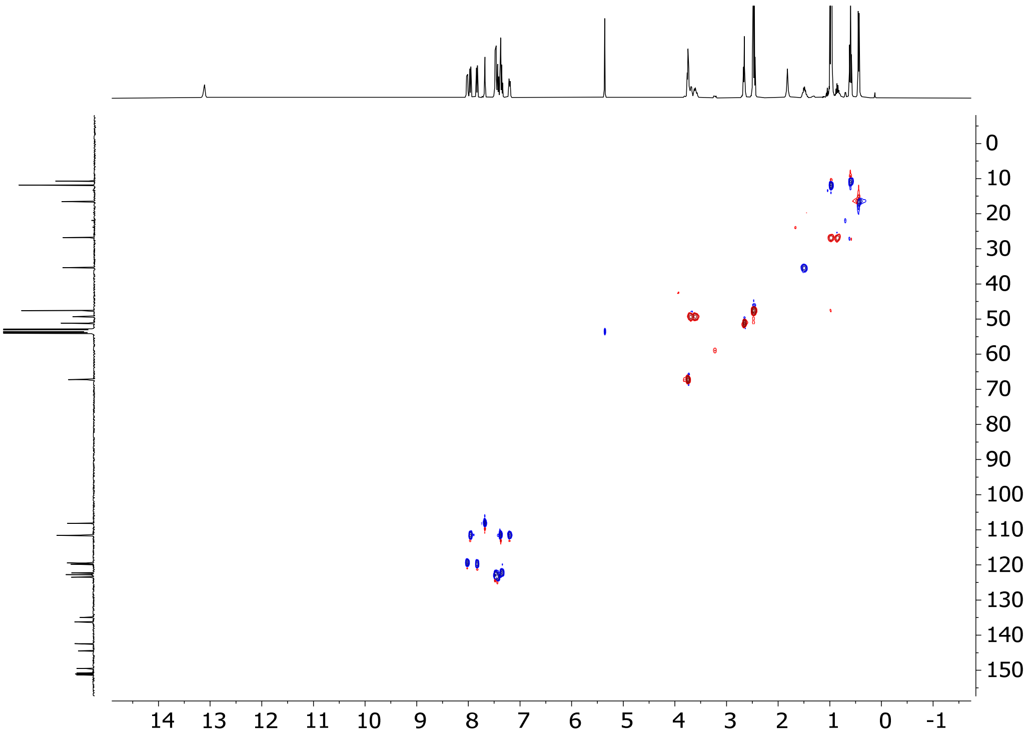

Figure A1-29. HSQC spectrum of **L5** in CD_2Cl_2.

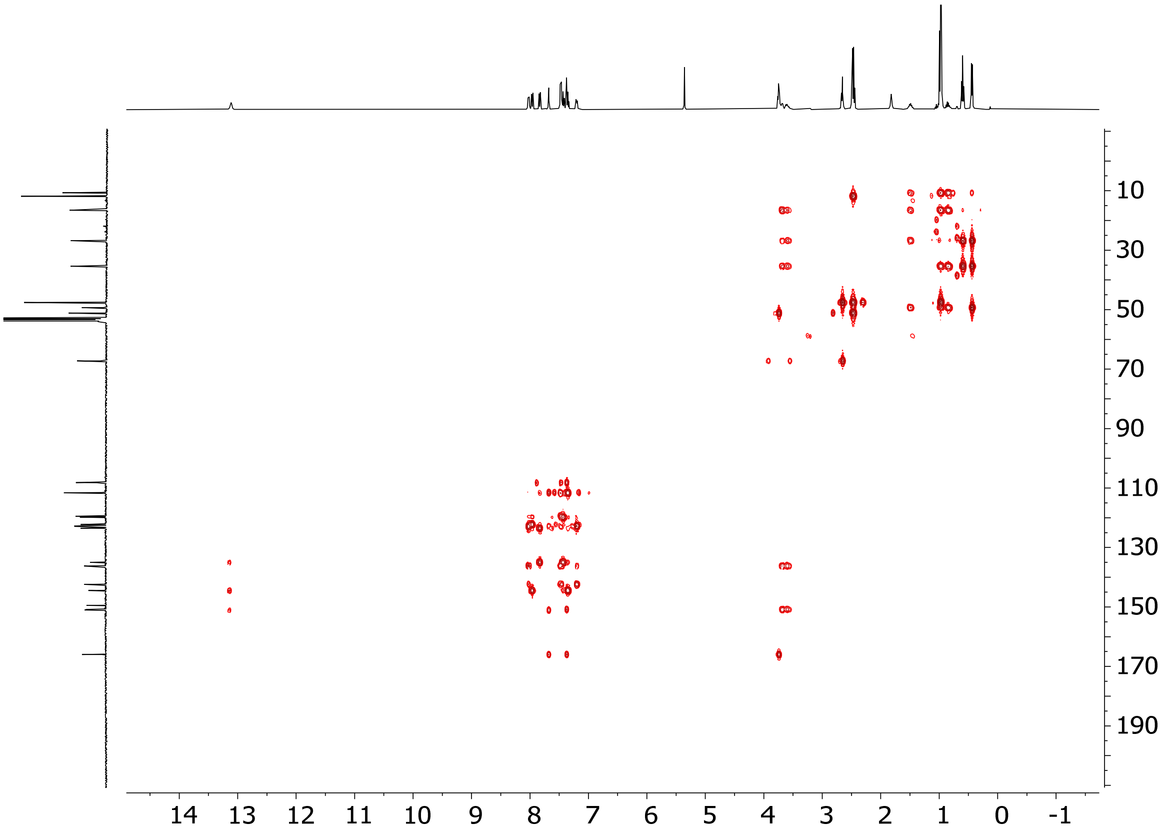

Figure A1-30. HMBC spectrum of **L5** in CD_2Cl_2.

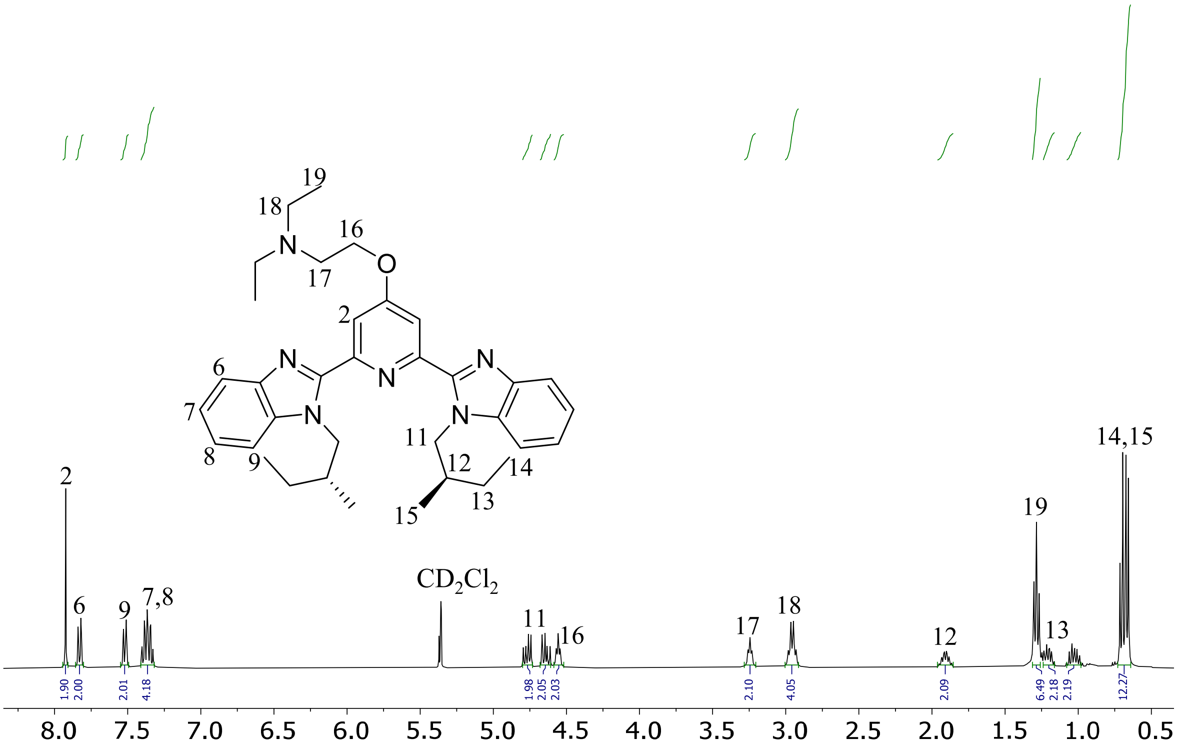

Figure A1-31. ^{1}H NMR spectrum of **L6** in CD_2Cl_2.

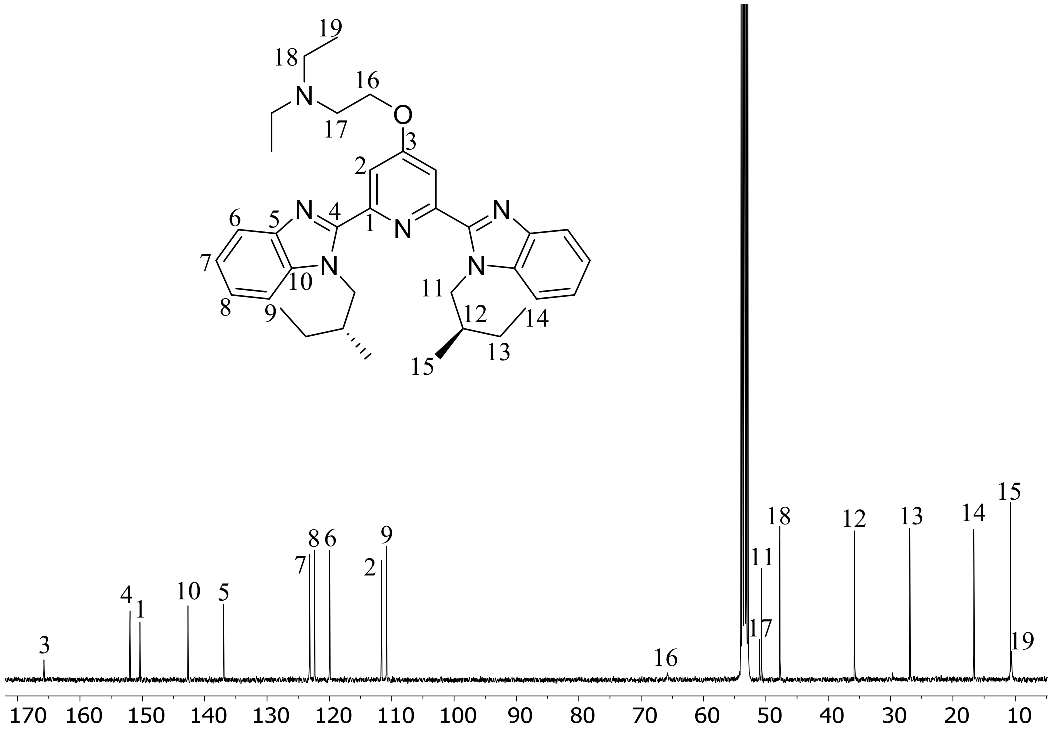

Figure A1-32. ^{13}C NMR spectrum of **L6** in CD_2Cl_2.

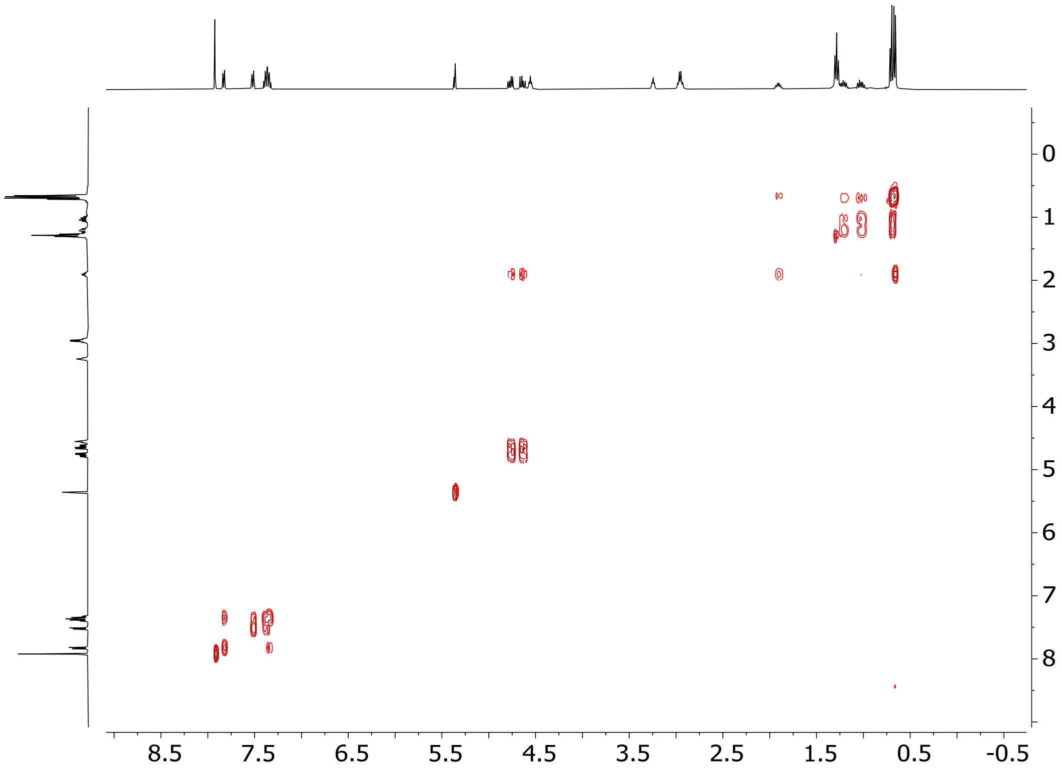

Figure A1-33. COSY spectrum of **L6** in CD_2Cl_2.

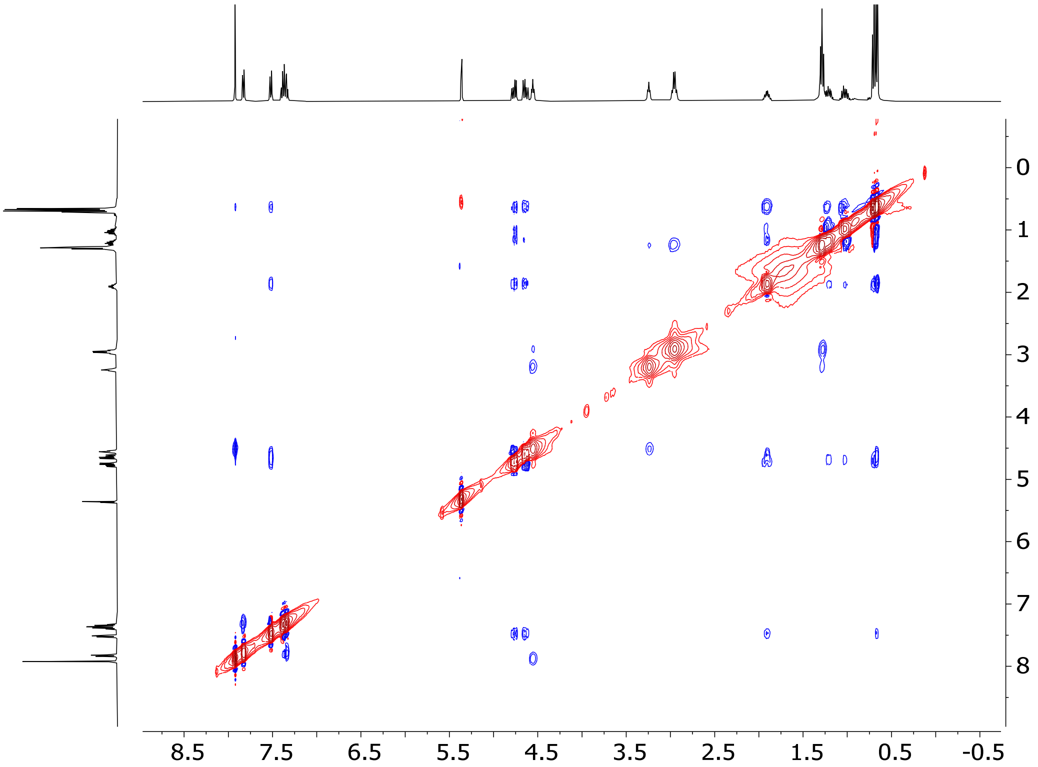

Figure A1-34. NOESY spectrum of **L6** in CD_2Cl_2.

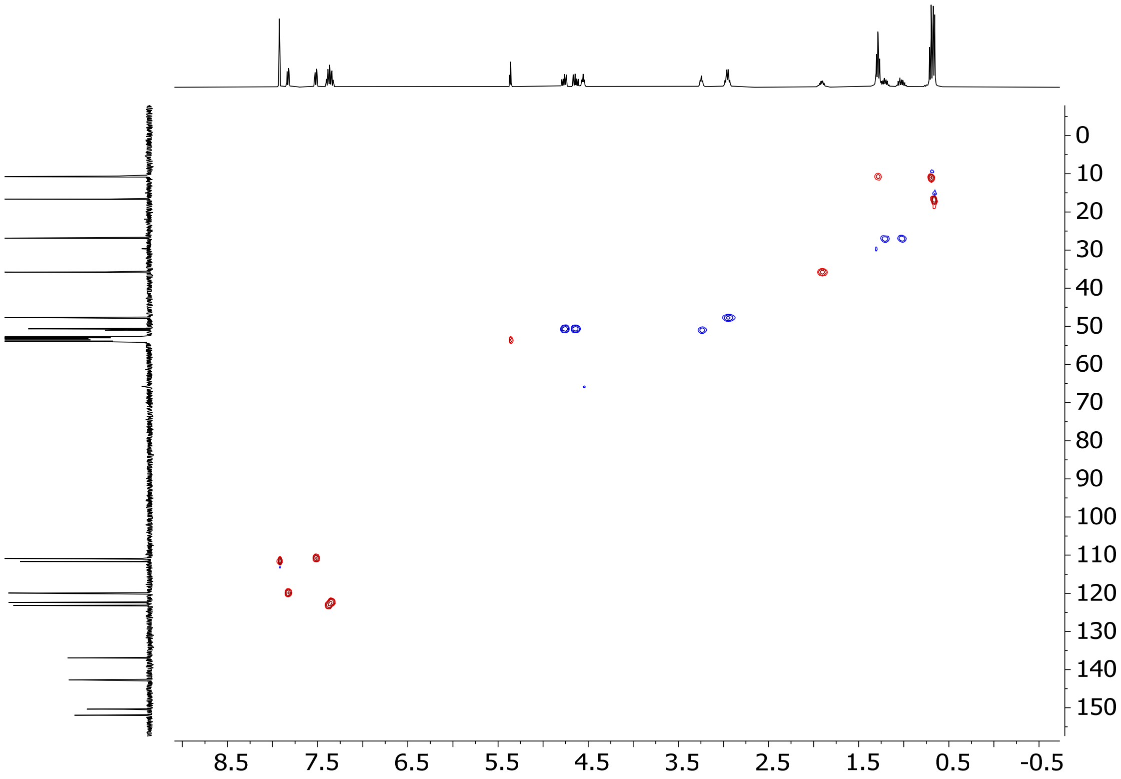

Figure A1-35. HSQC spectrum of **L6** in CD_2Cl_2.

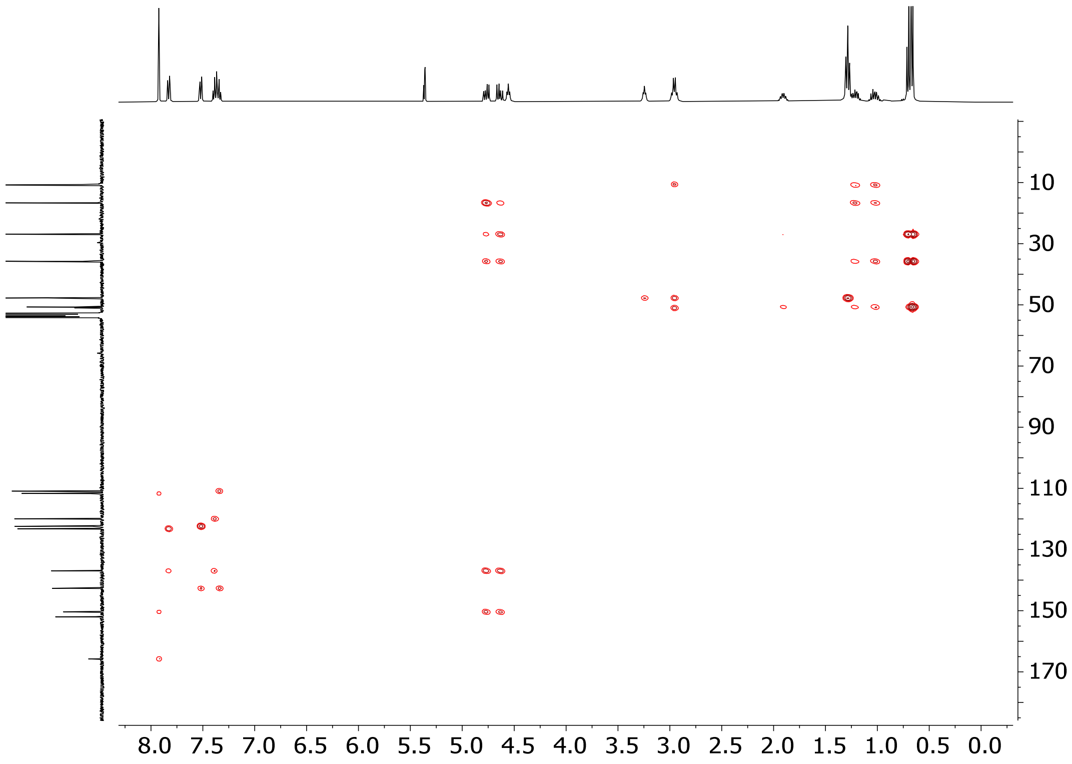

Figure A1-36. HMBC spectrum of **L6** in CD_2Cl_2.

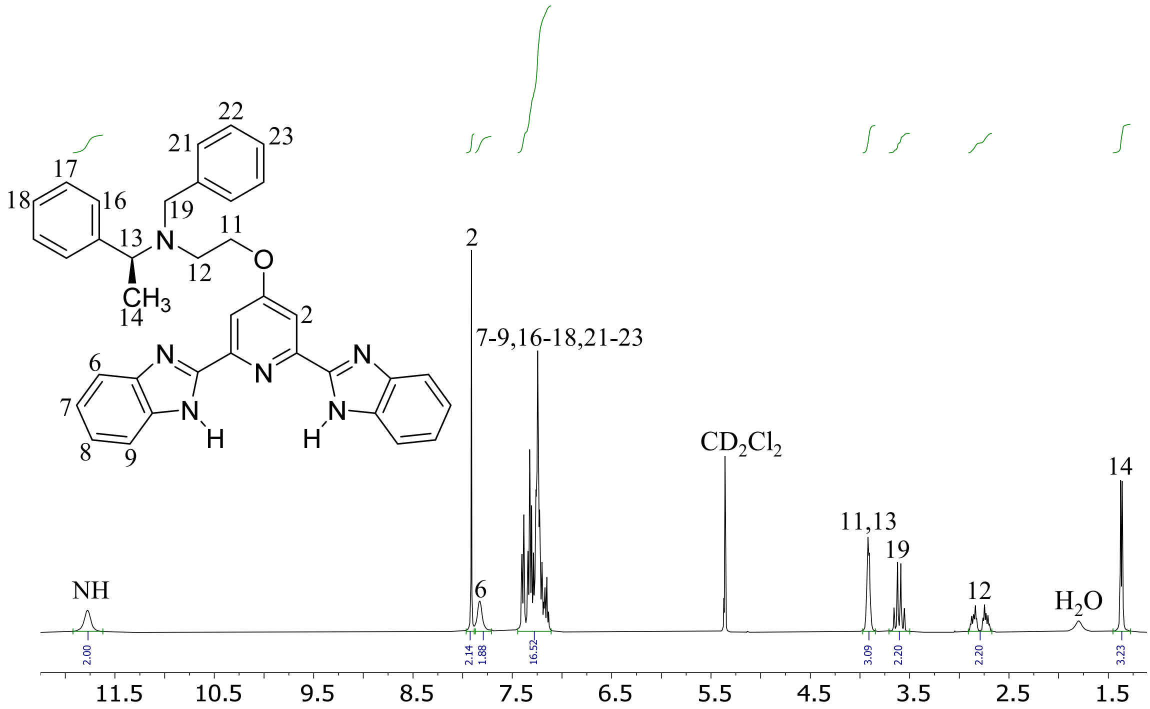

Figure A1-37. ^{1}H NMR spectrum of **L7** in CD_2Cl_2.

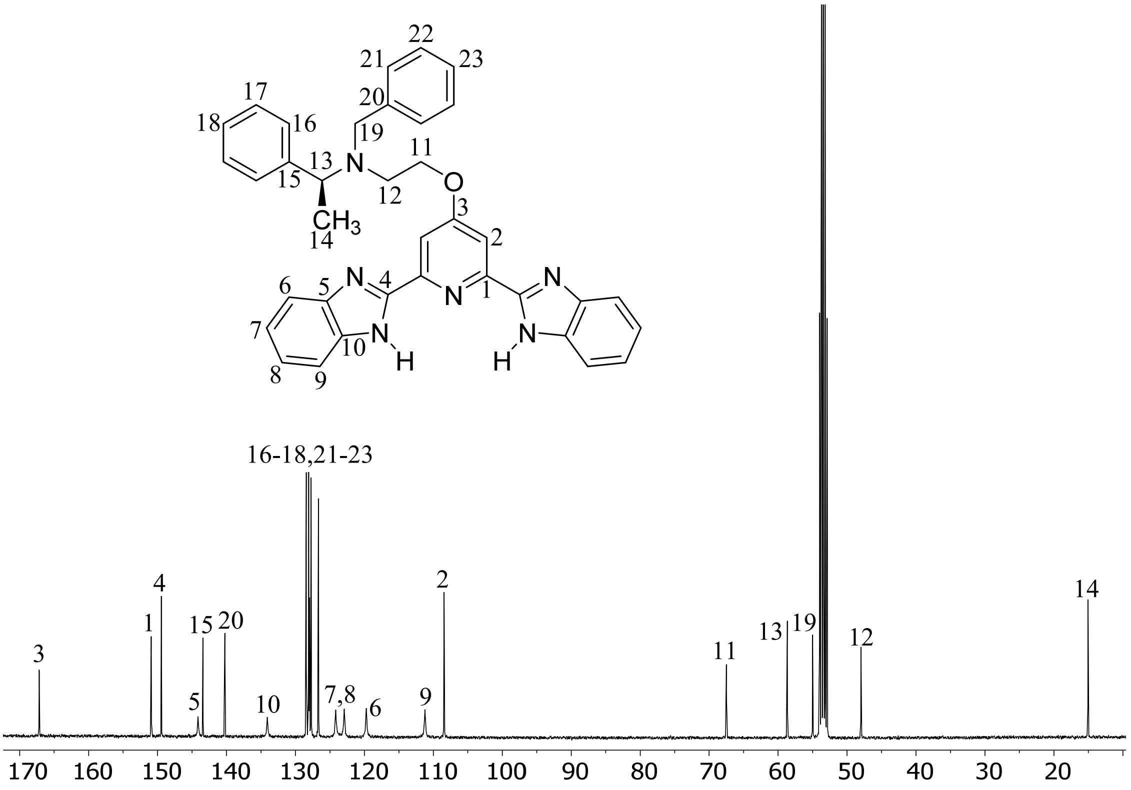

Figure A1-38. ^{13}C NMR spectrum of **L7** in CD_2Cl_2.

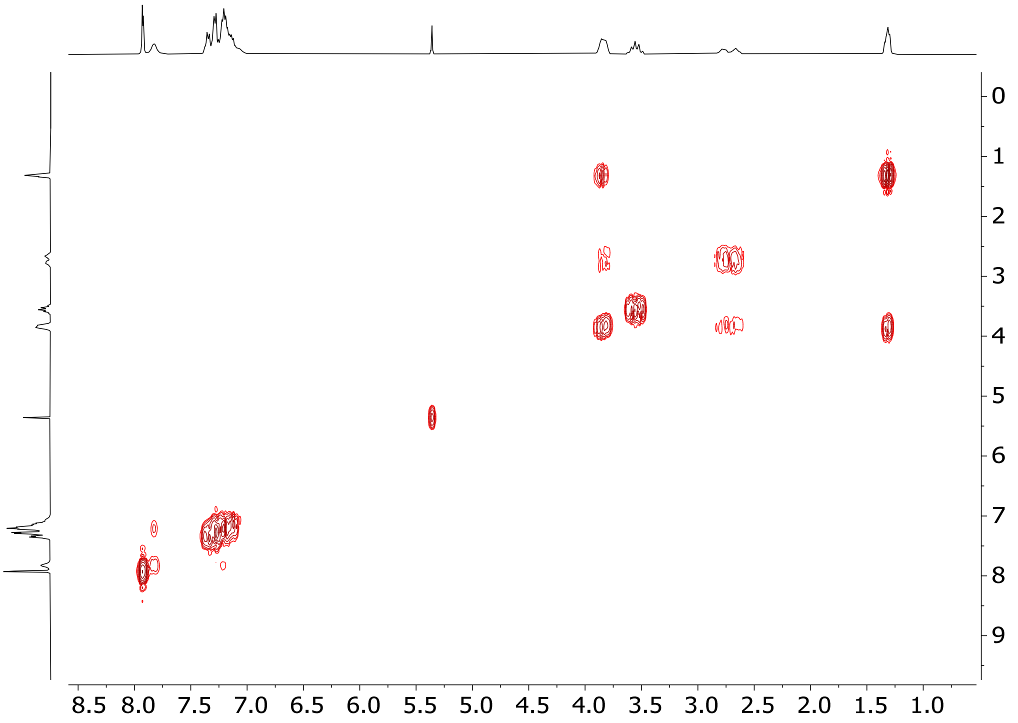

Figure A1-39. COSY spectrum of **L7** in CD_2Cl_2.

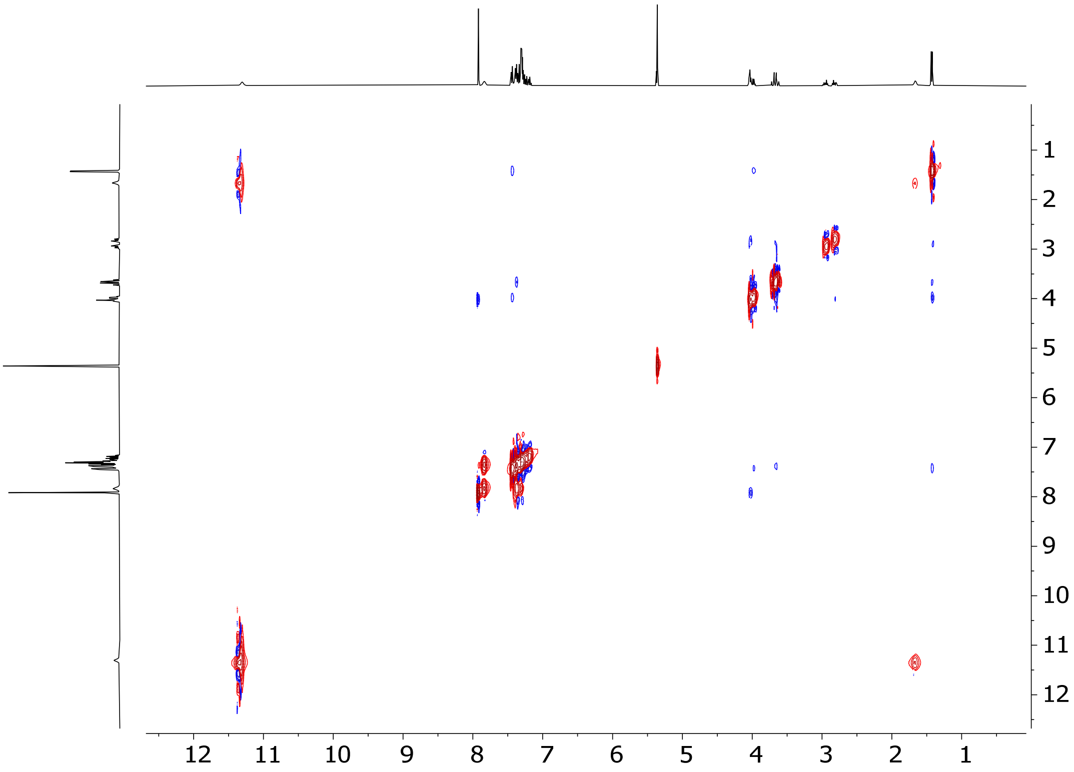

Figure A1-40. NOESY spectrum of **L7** in CD_2Cl_2.

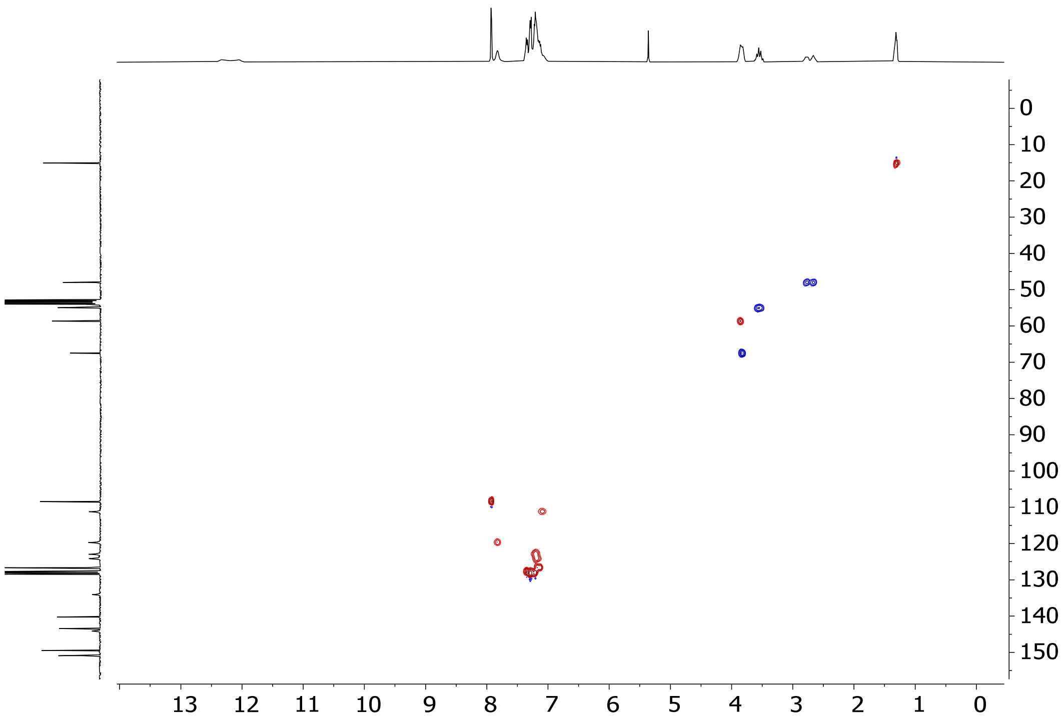

Figure A1-41. HSQC spectrum of **L7** in CD_2Cl_2.

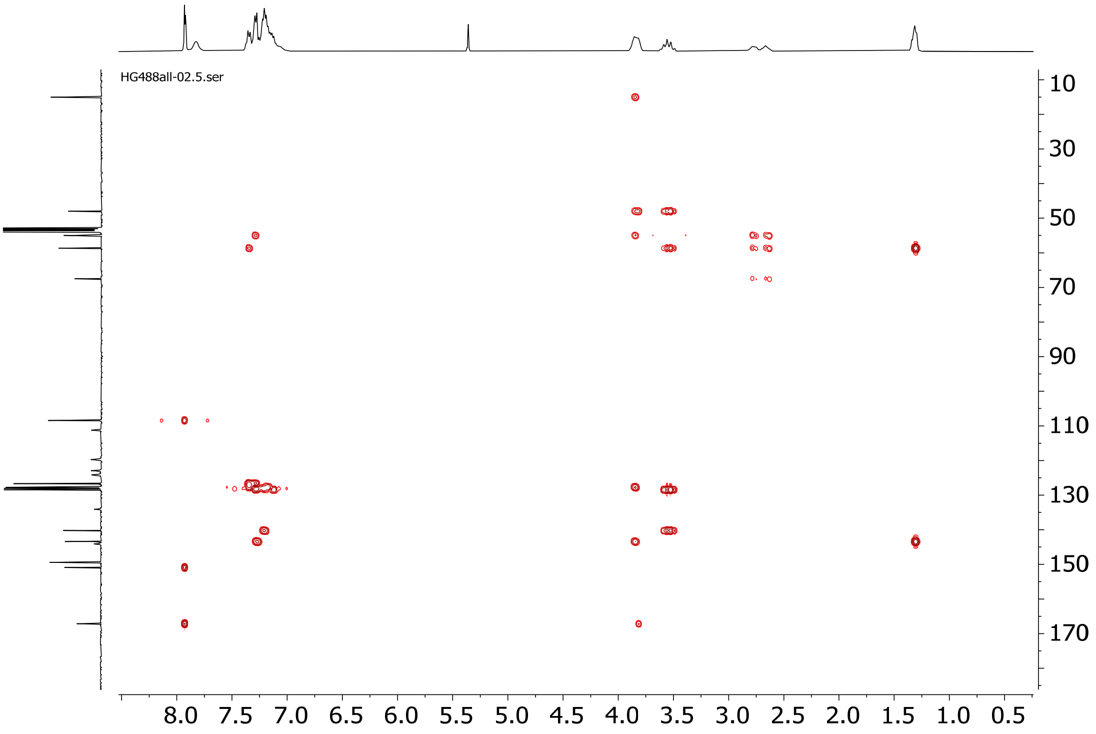

Figure A1-42. HMBC spectrum of **L7** in CD_2Cl_2.

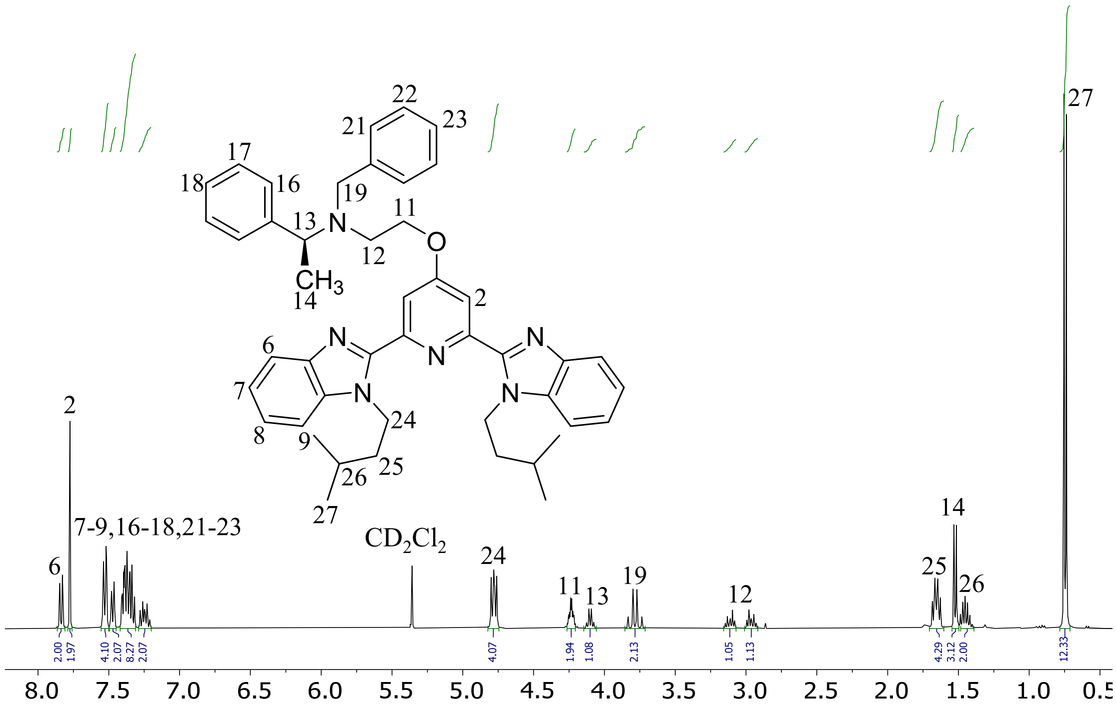

Figure A1-43. ^{1}H NMR spectrum of **L8** in CD_2Cl_2.

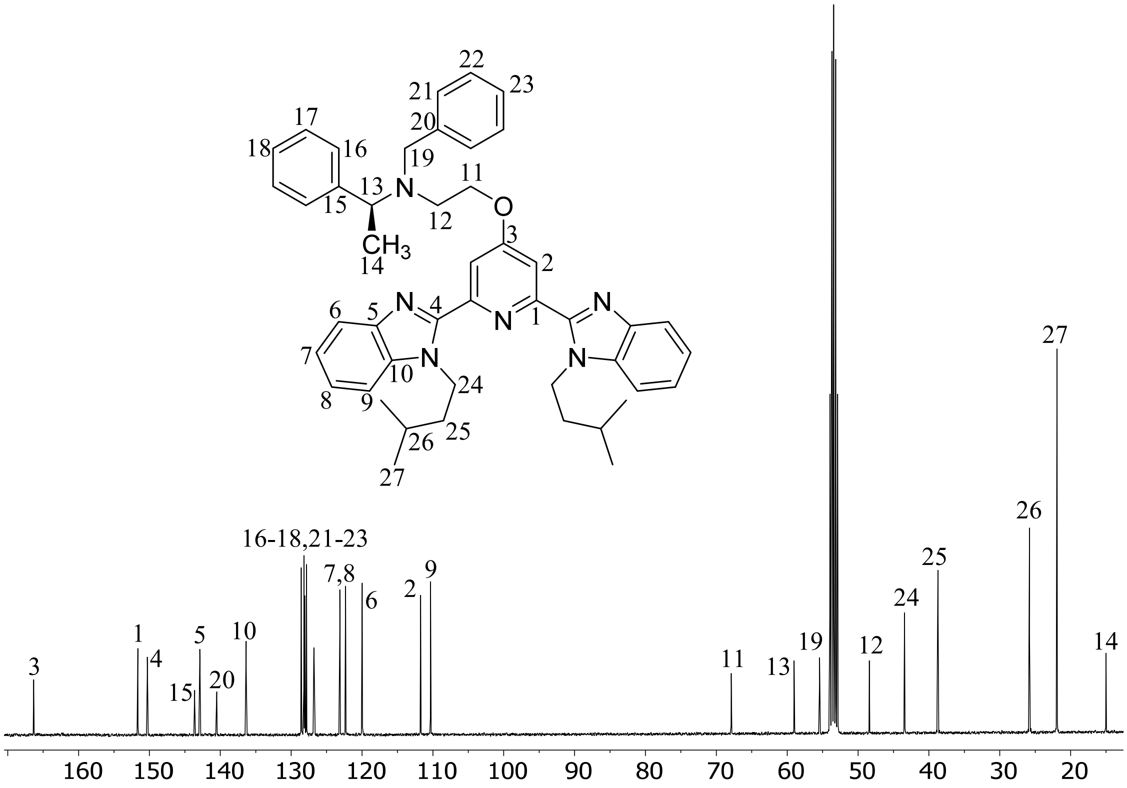

Figure A1-44. ^{13}C NMR spectrum of **L8** in CD_2Cl_2.

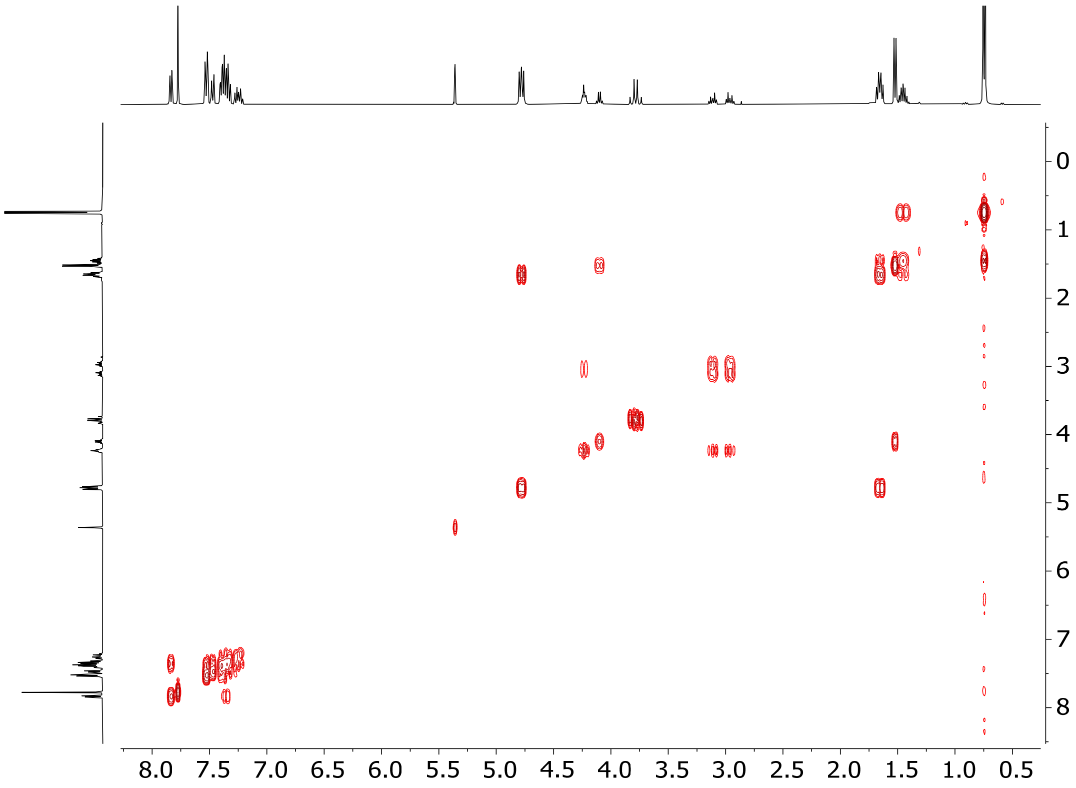

Figure A1-45. COSY spectrum of **L8** in CD_2Cl_2.

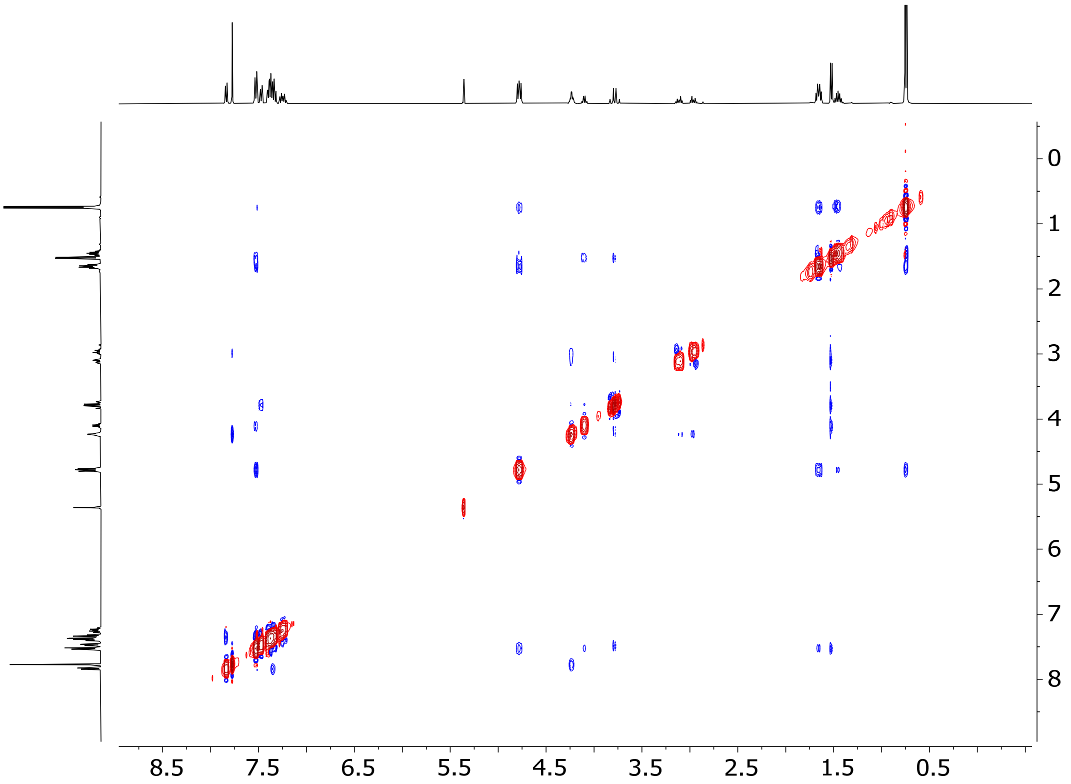

Figure A1-46. NOESY spectrum of **L8** in CD_2Cl_2.

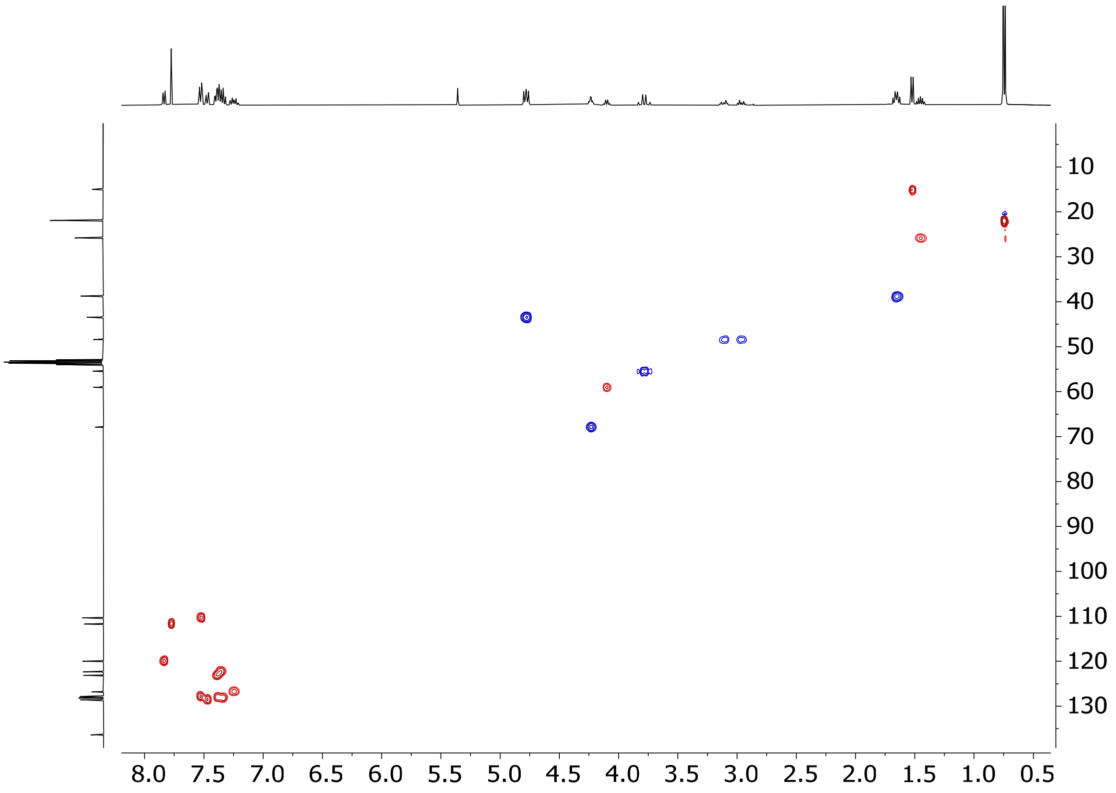

Figure A1-47. HSQC spectrum of **L8** in CD_2Cl_2.

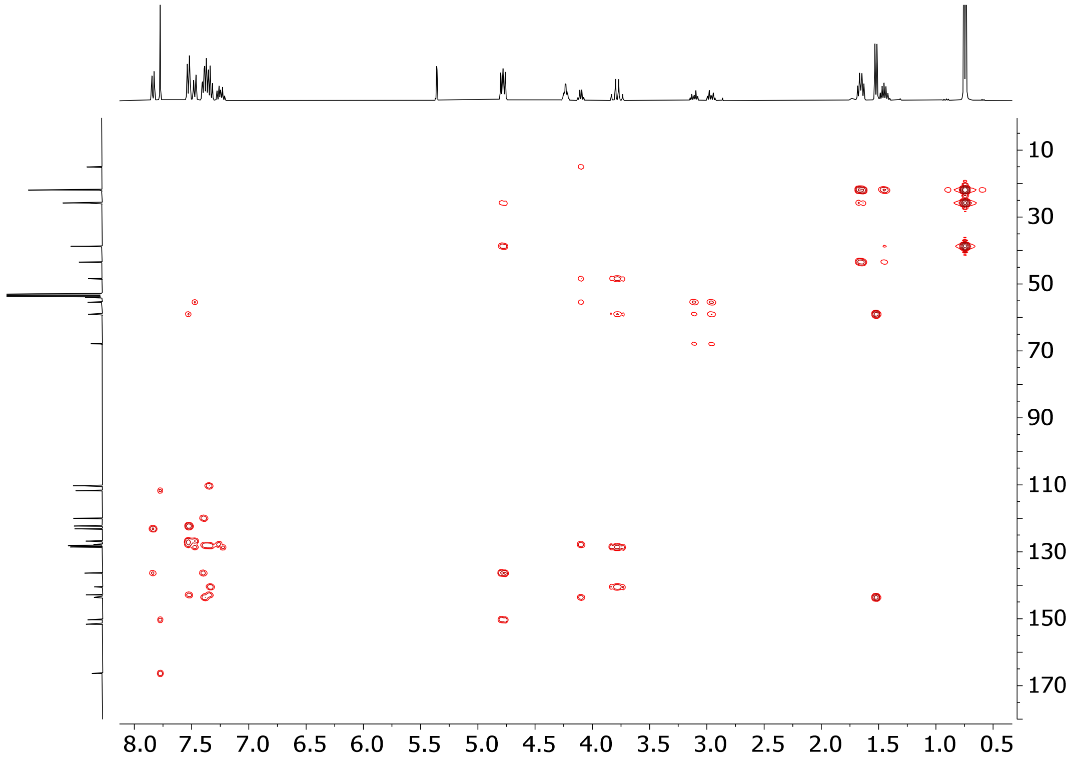

Figure A1-48. HMBC spectrum of **L8** in CD_2Cl_2.

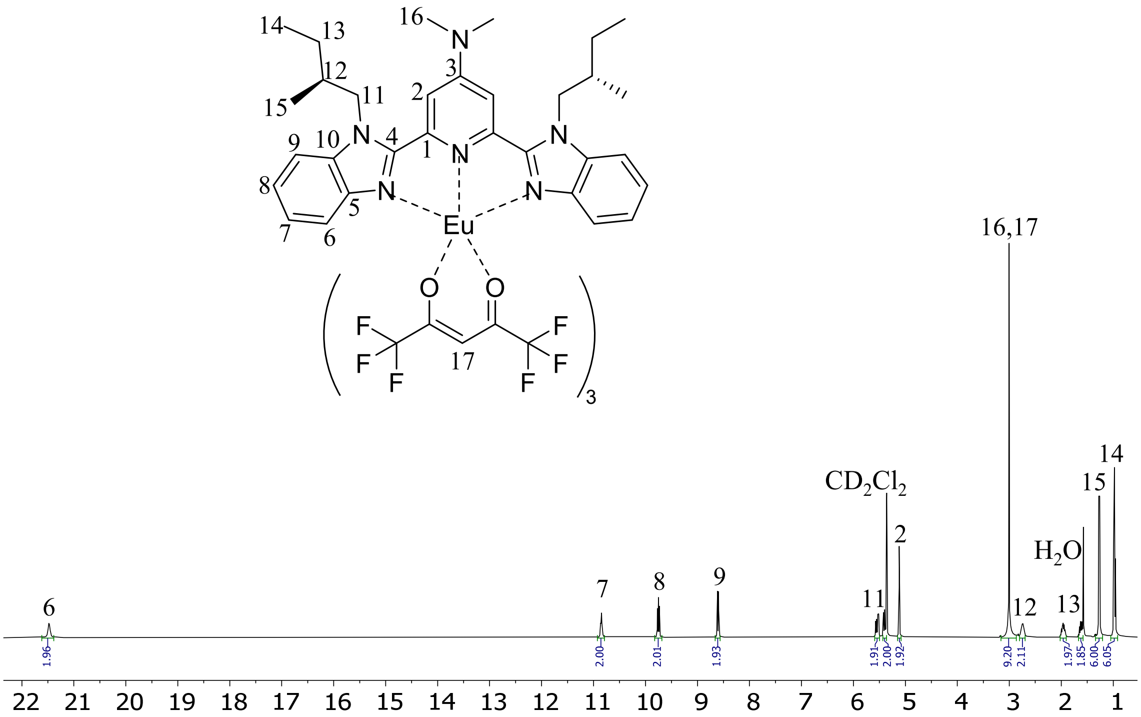

Figure A1-49. ^{1}H NMR spectrum of [**L3**Eu(hfac)$_3$] in CD_2Cl_2.

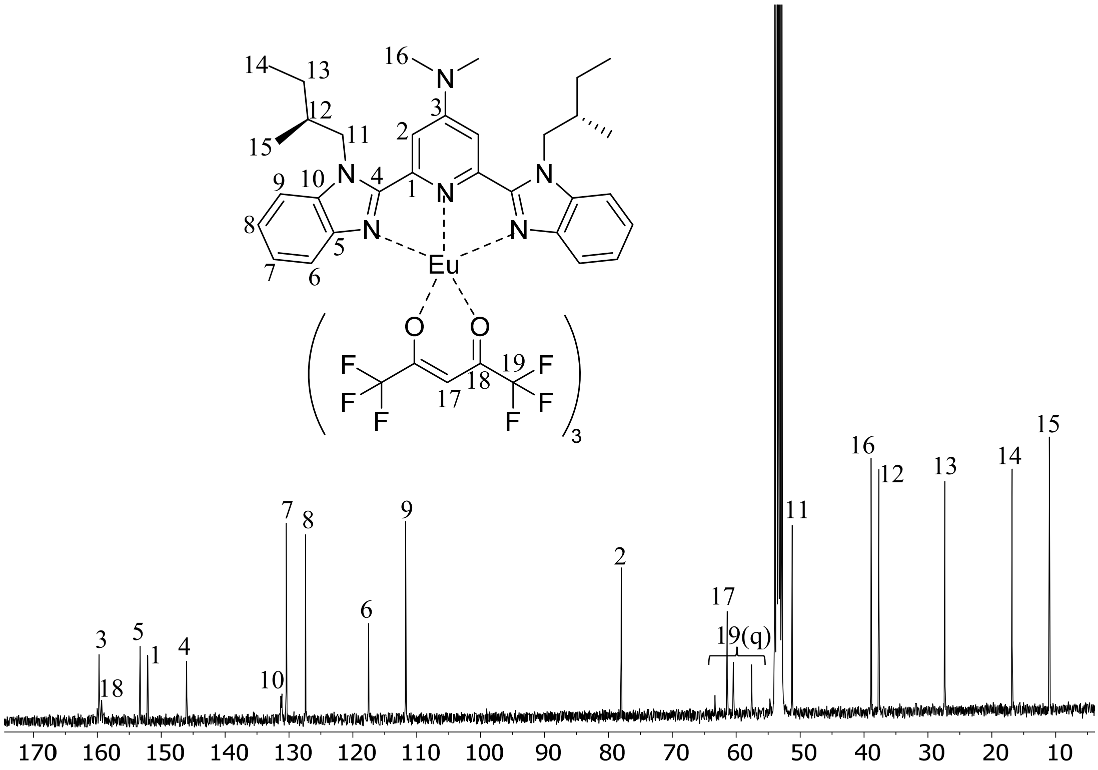

Figure A1-50. ^{13}C NMR spectrum of [**L3**Eu(hfac)$_3$] in CD_2Cl_2.

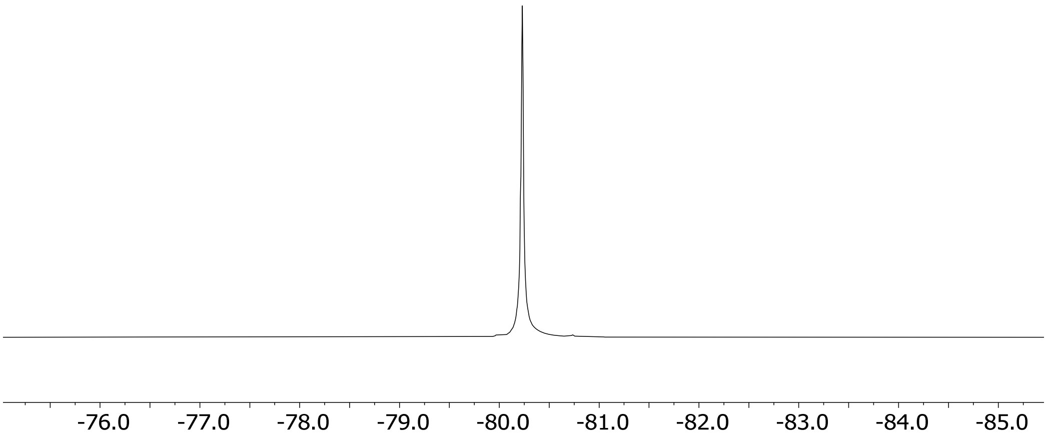

Figure A1-51. ^{19}F NMR spectrum of [**L3**Eu(hfac)$_3$] in CD_2Cl_2.

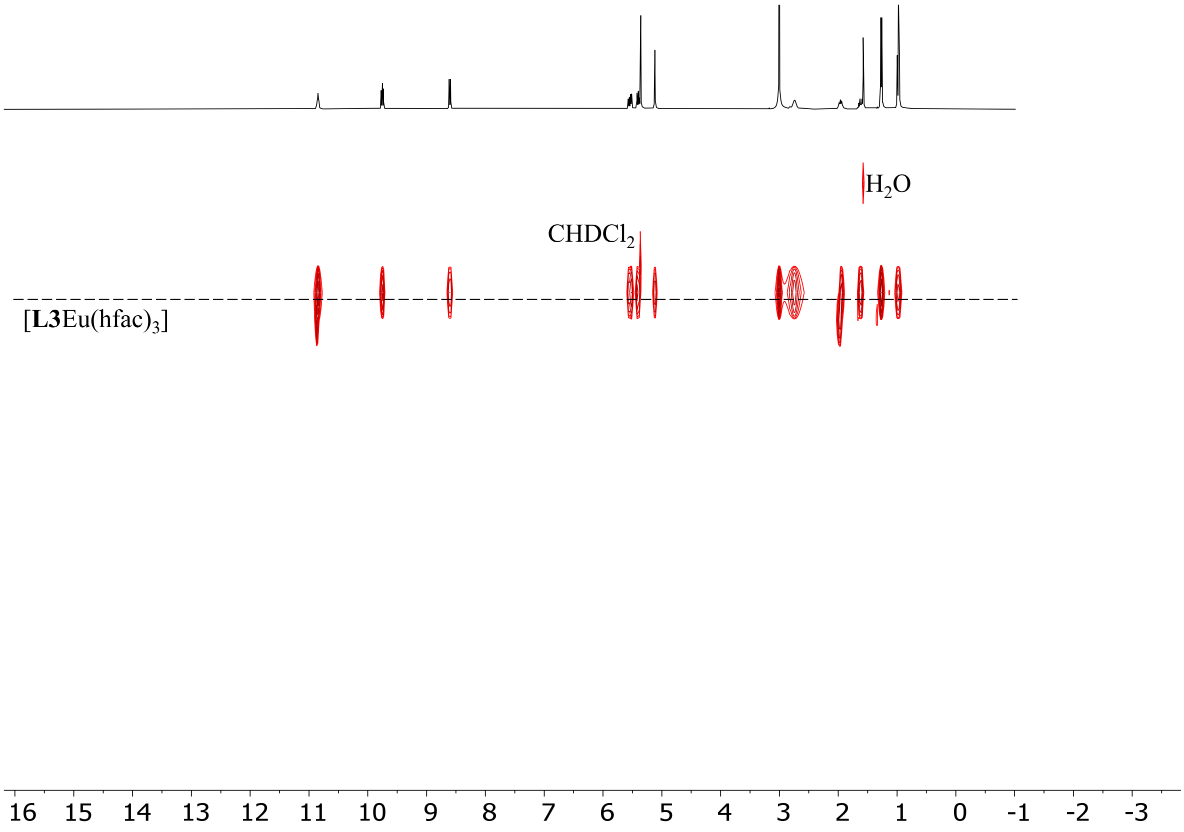

Figure A1-52. DOSY spectrum of [**L3**Eu(hfac)$_3$] in CD_2Cl_2.

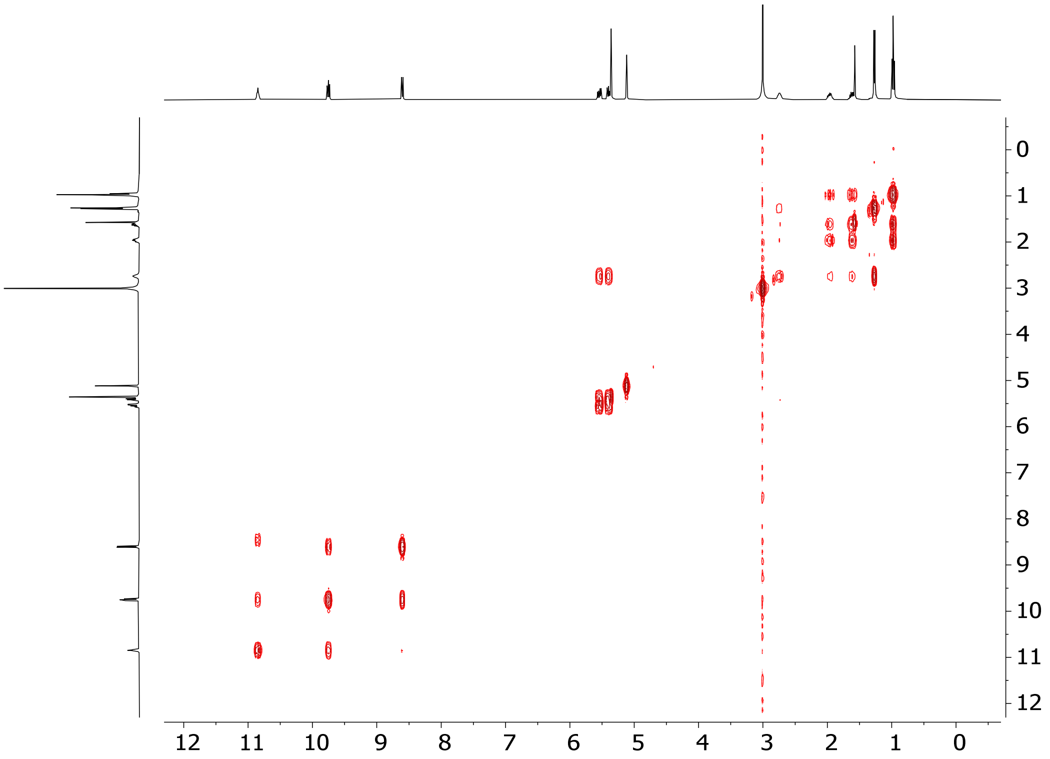

Figure A1-53. COSY spectrum of [**L3**Eu(hfac)$_3$] in CD_2Cl_2.

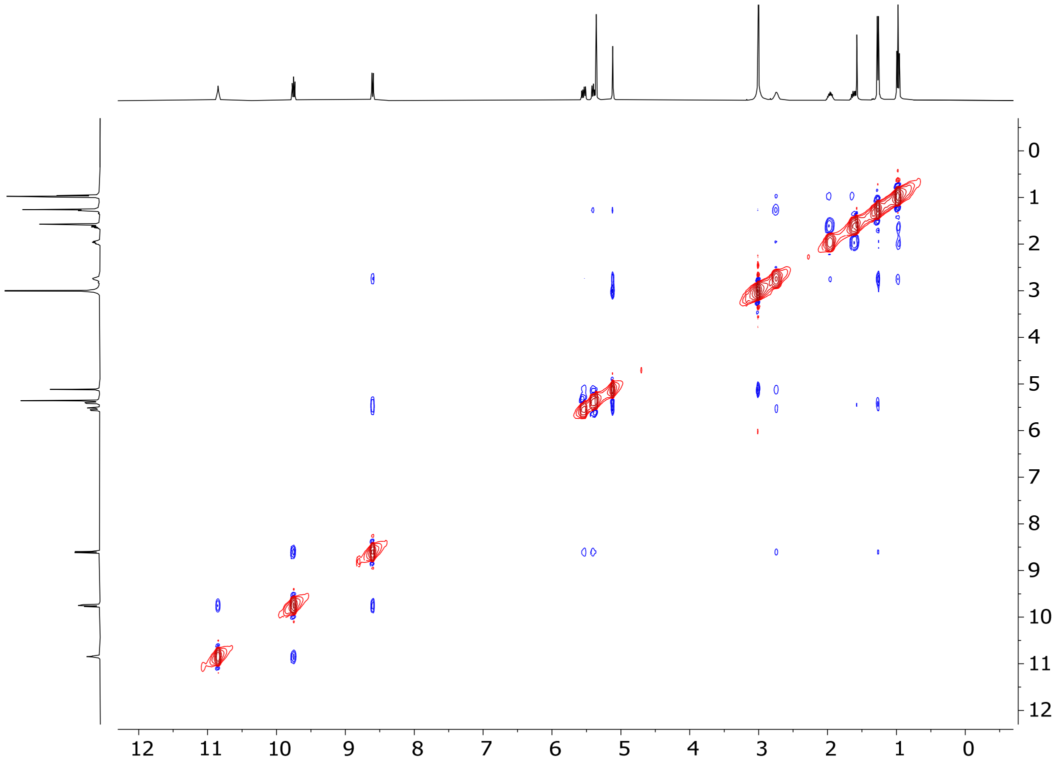

Figure A1-54. NOESY spectrum of [**L3**Eu(hfac)$_3$] in CD_2Cl_2.

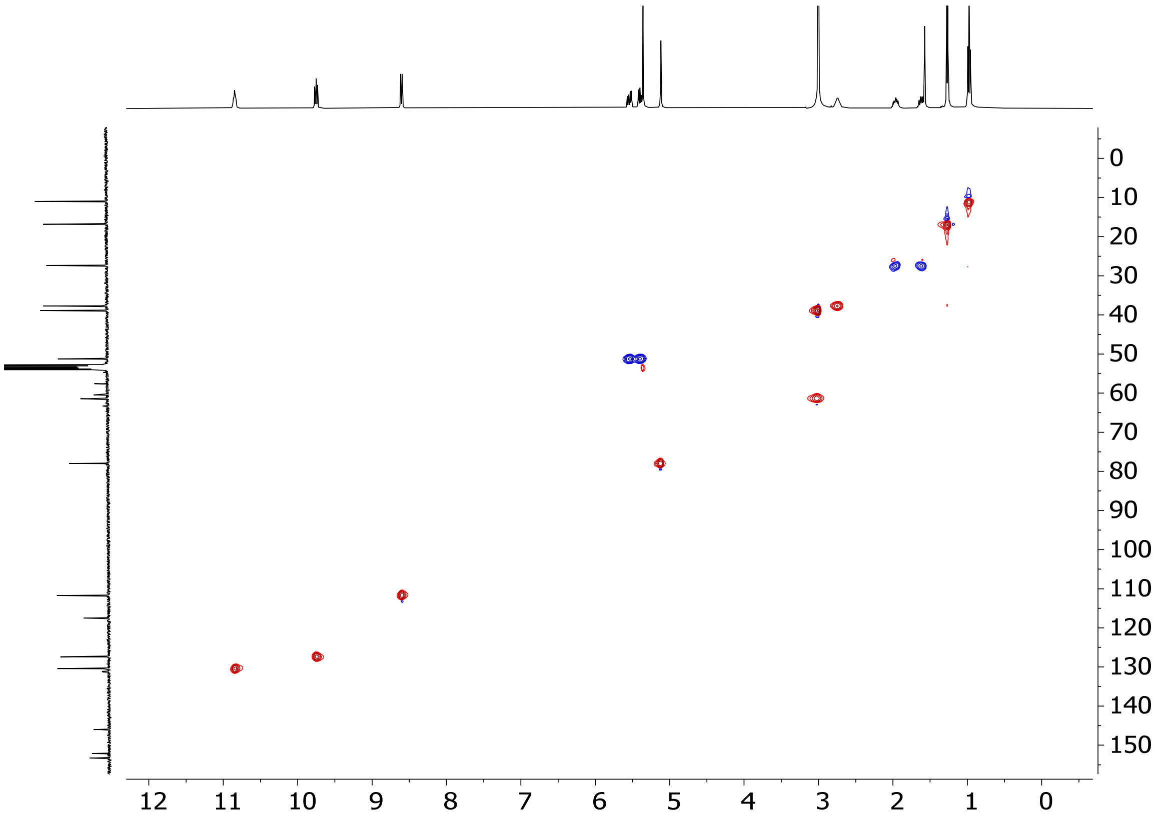

Figure A1-55. HSQC spectrum of [**L3**Eu(hfac)$_3$] in CD_2Cl_2.

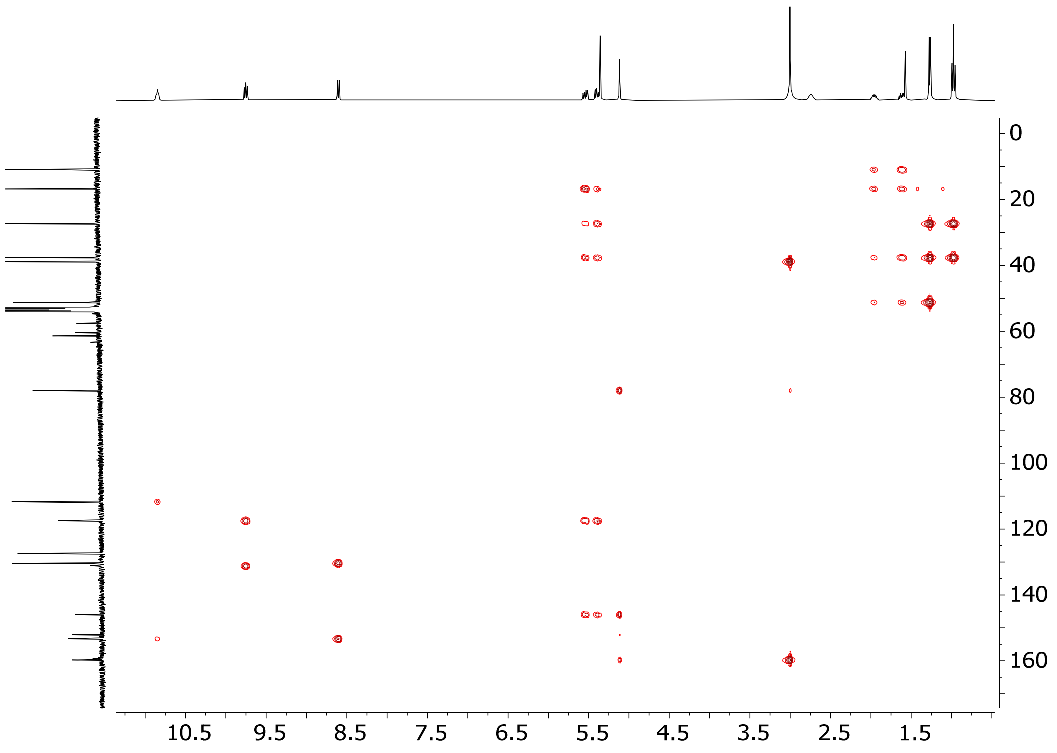

Figure A1-56. HMBC spectrum of [**L3**Eu(hfac)$_3$] in CD_2Cl_2.

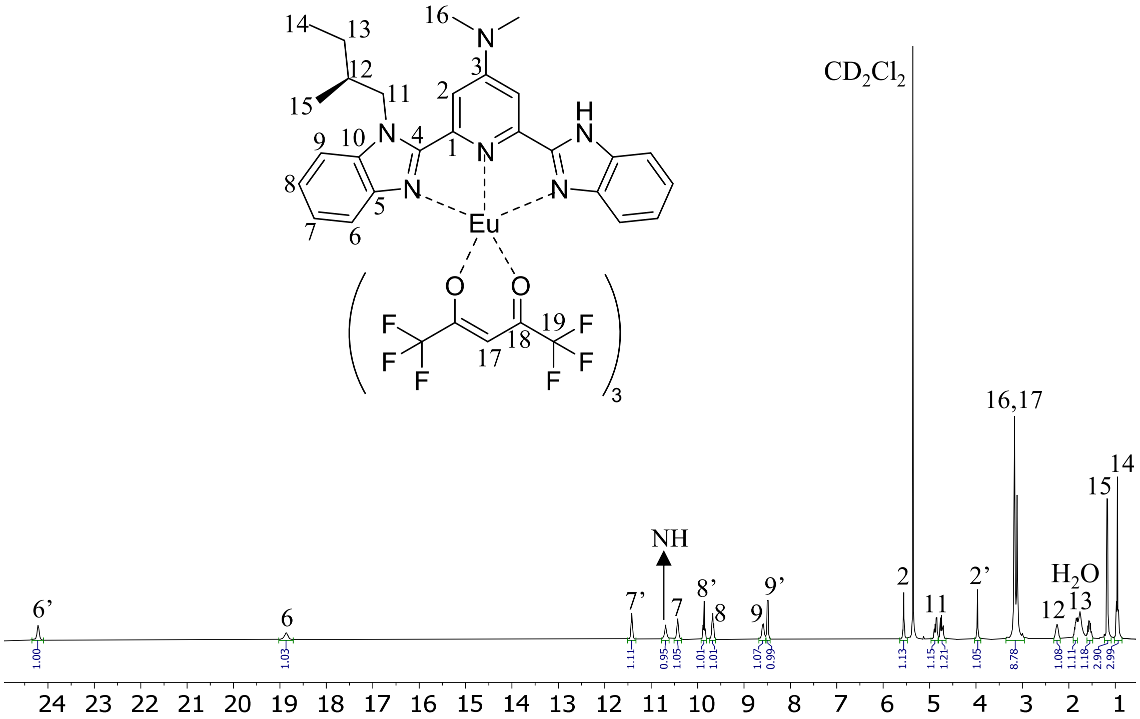

Figure A1-57. ^{1}H NMR spectrum of [**L2**Eu(hfac)$_3$] in CD$_2$Cl$_2$.

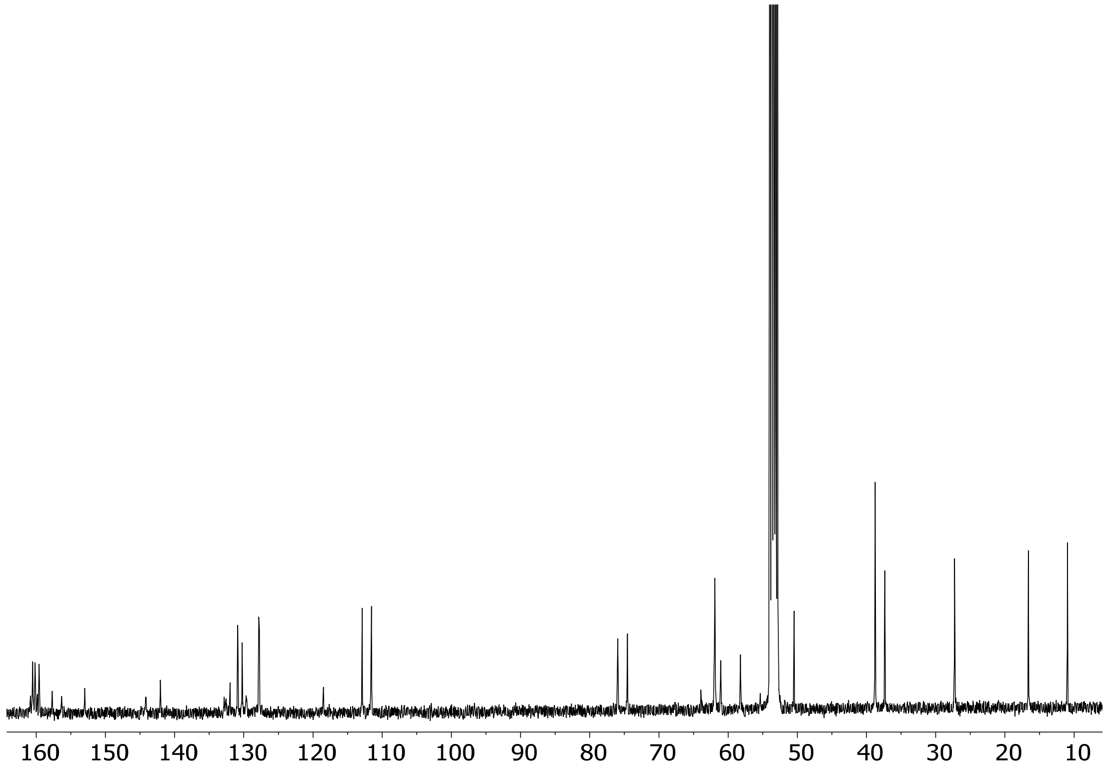

Figure A1-58. ^{13}C NMR spectrum of [**L2**Eu(hfac)$_3$] in CD$_2$Cl$_2$.

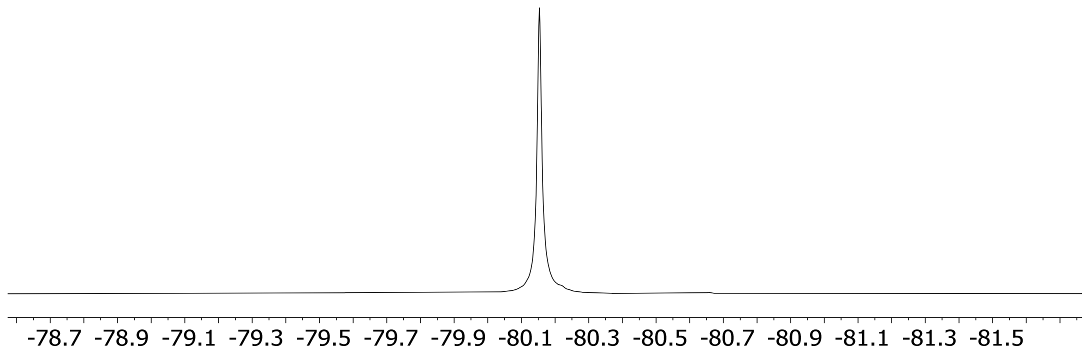

Figure A1-59. ^{19}F NMR spectrum of [**L2**Eu(hfac)$_3$] in CD_2Cl_2.

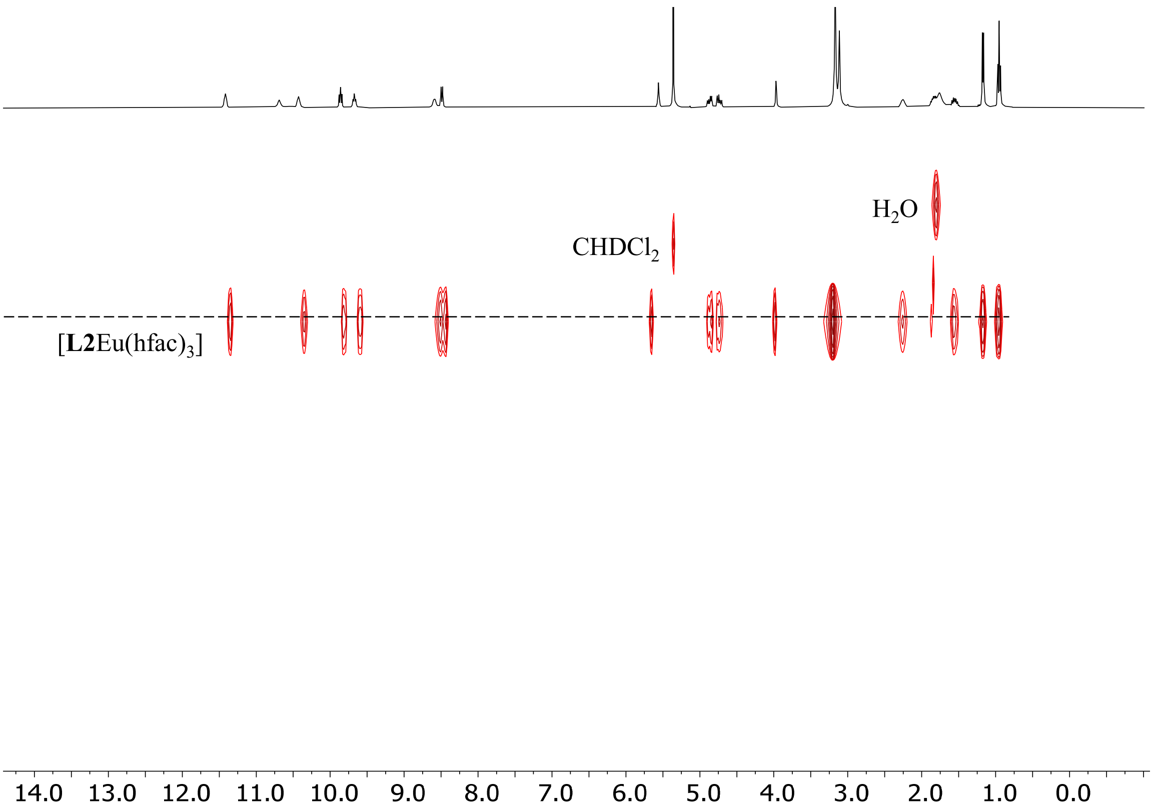

Figure A1-60. DOSY spectrum of [**L2**Eu(hfac)$_3$] in CD_2Cl_2.

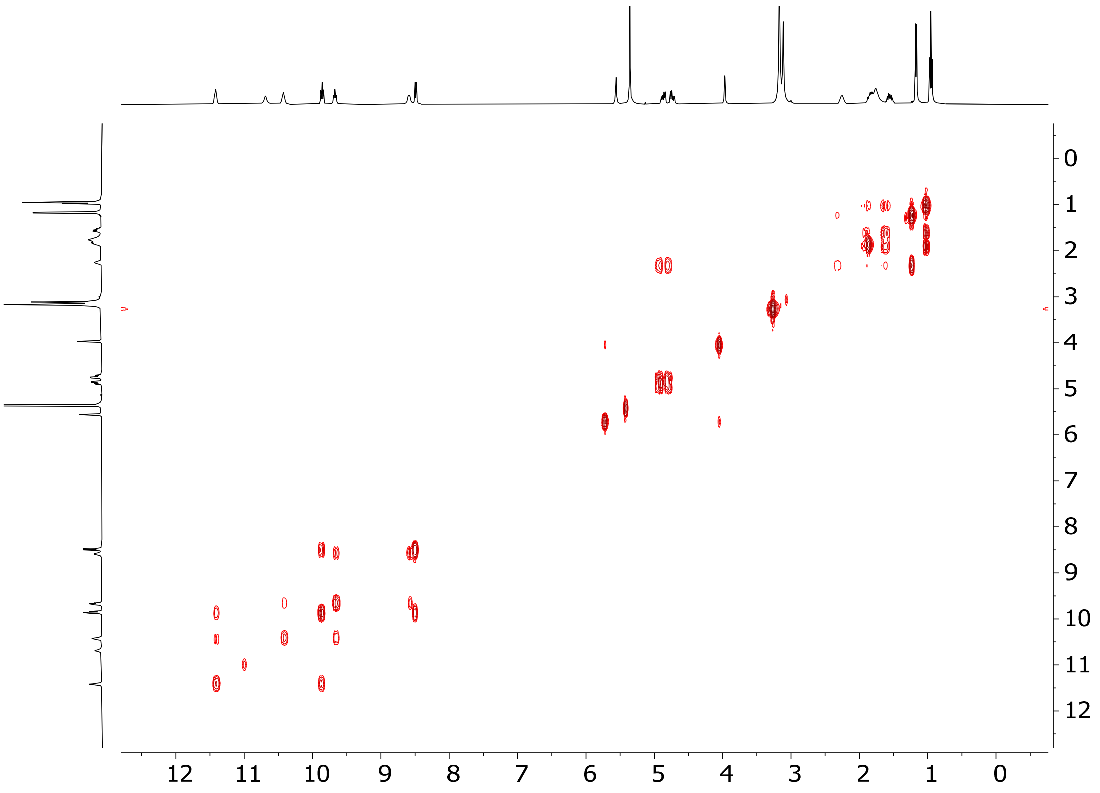

Figure A1-61. COSY spectrum of [**L2**Eu(hfac)$_3$] in CD_2Cl_2.

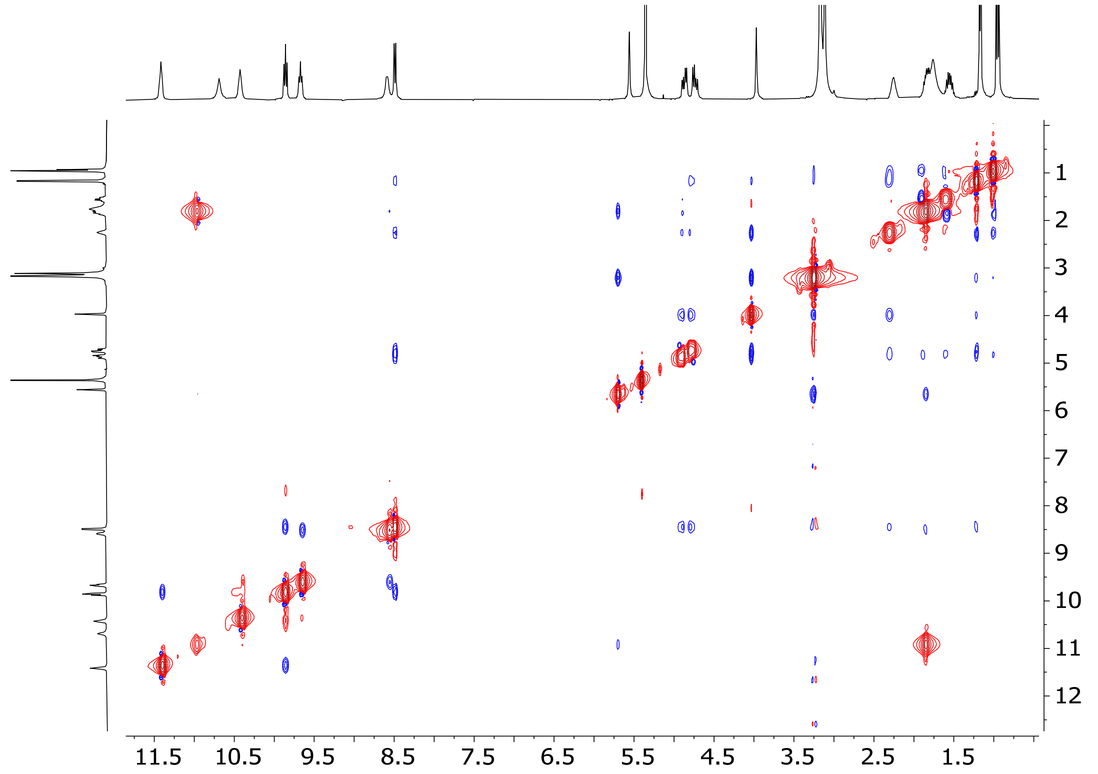

Figure A1-62. NOESY spectrum of [**L2**Eu(hfac)$_3$] in CD_2Cl_2.

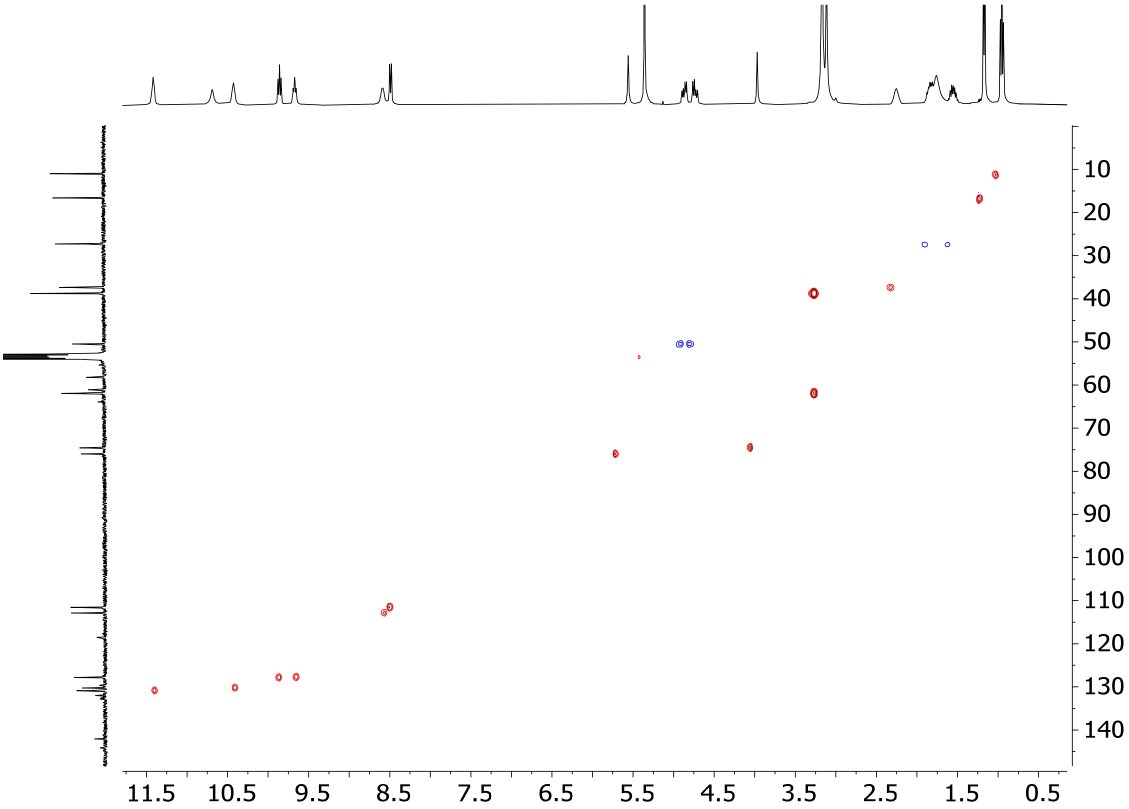

Figure A1-63. HSQC spectrum of [**L2**Eu(hfac)$_3$] in CD_2Cl_2.

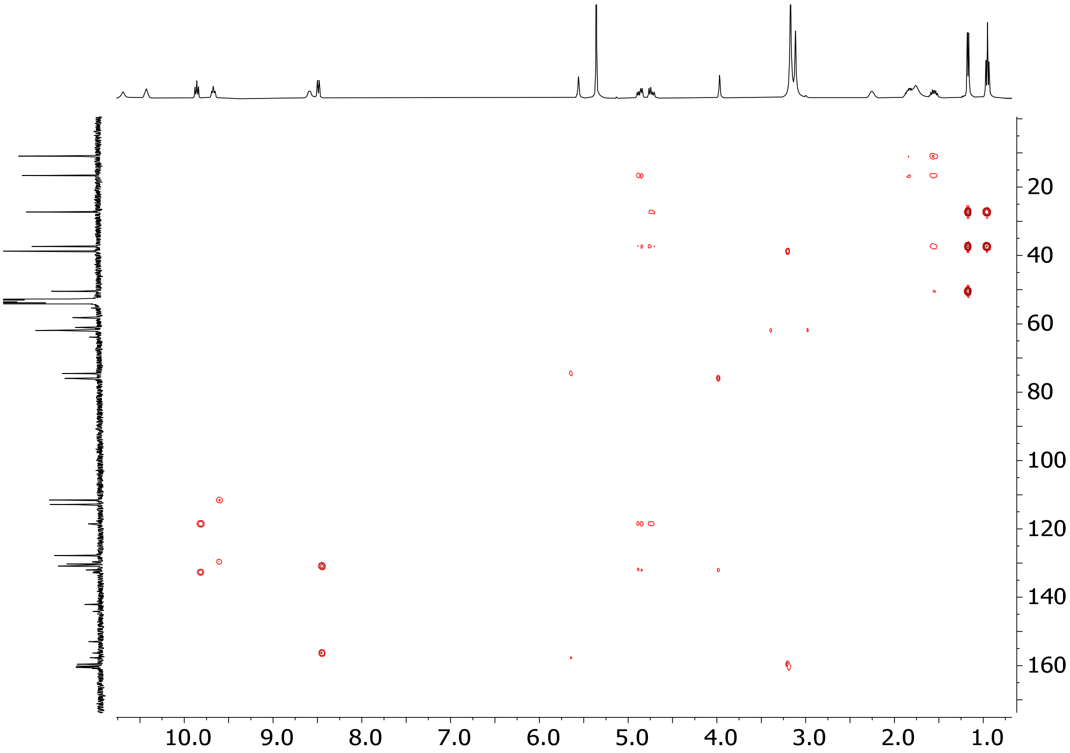

Figure A1-64. HMBC spectrum of [**L2**Eu(hfac)$_3$] in CD_2Cl_2.

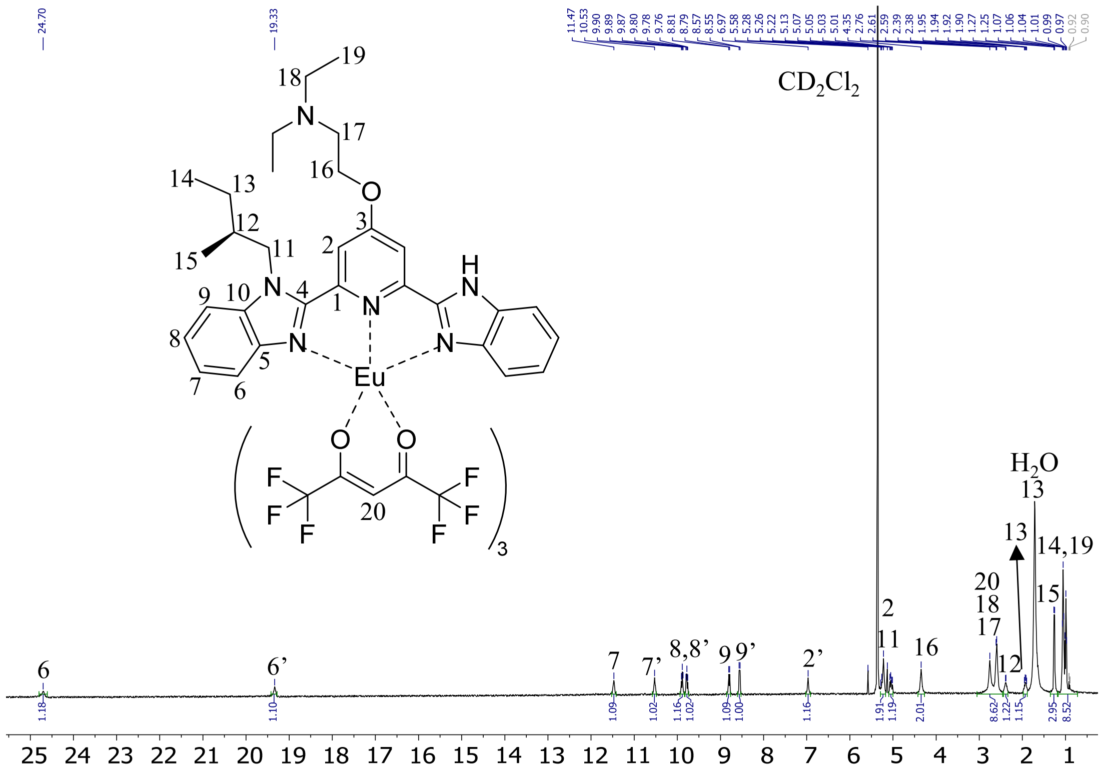

Figure A1-65. ^{1}H NMR spectrum of [**L5**Eu(hfac)3] in CD_2Cl_2.

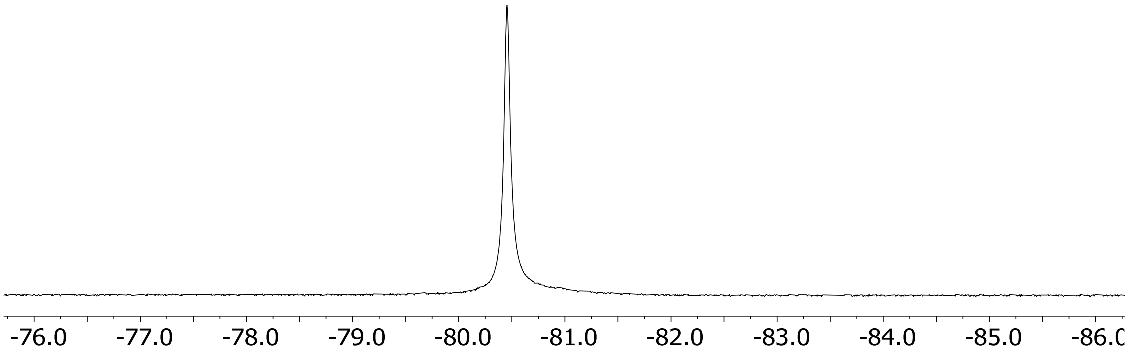

Figure A1-66. ^{19}F NMR spectrum of [**L5**Eu(hfac)3] in CD_2Cl_2.

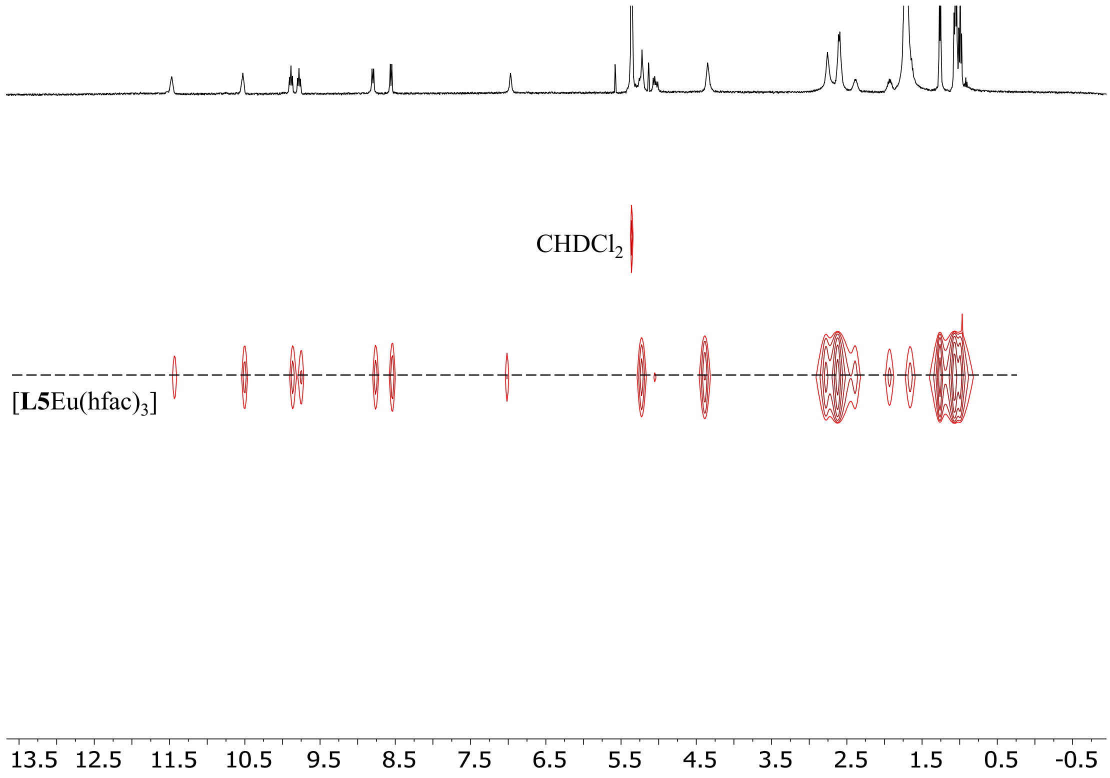

Figure A1-67. DOSY spectrum of [**L5**Eu(hfac)3] in CD_2Cl_2.

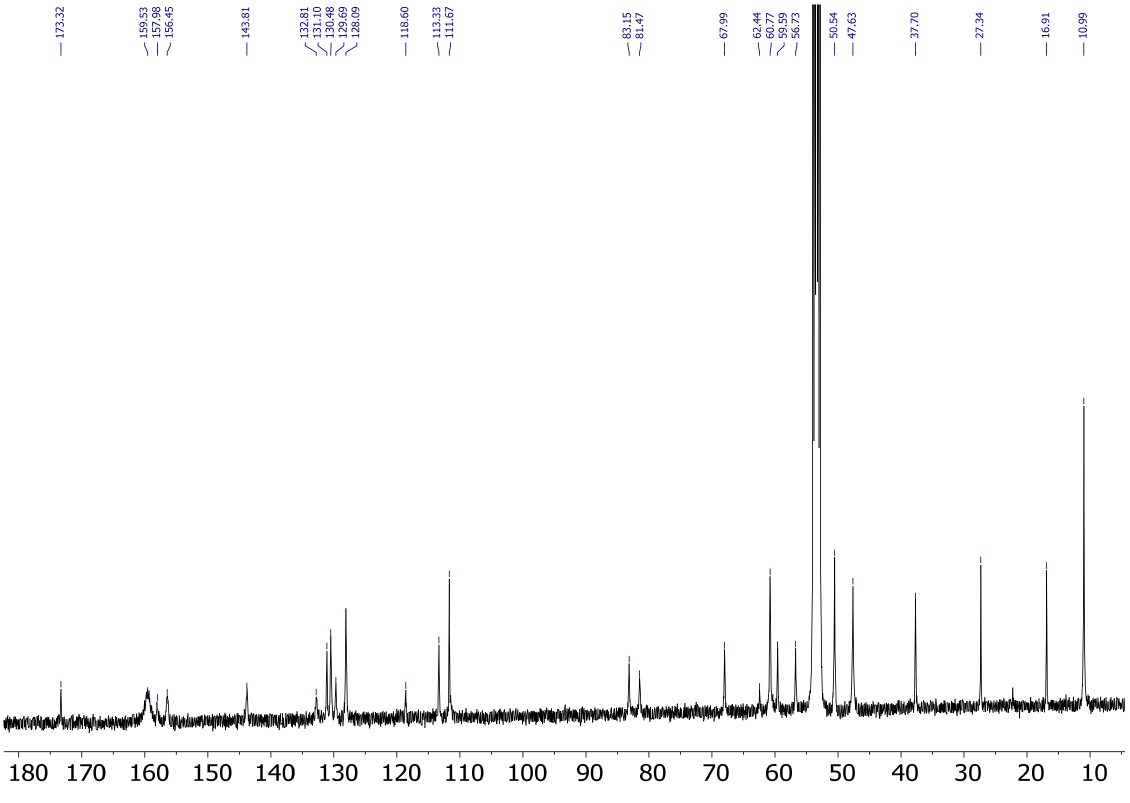

Figure A1-68. ^{13}C NMR spectrum of [**L5**Eu(hfac)3] in CD_2Cl_2.

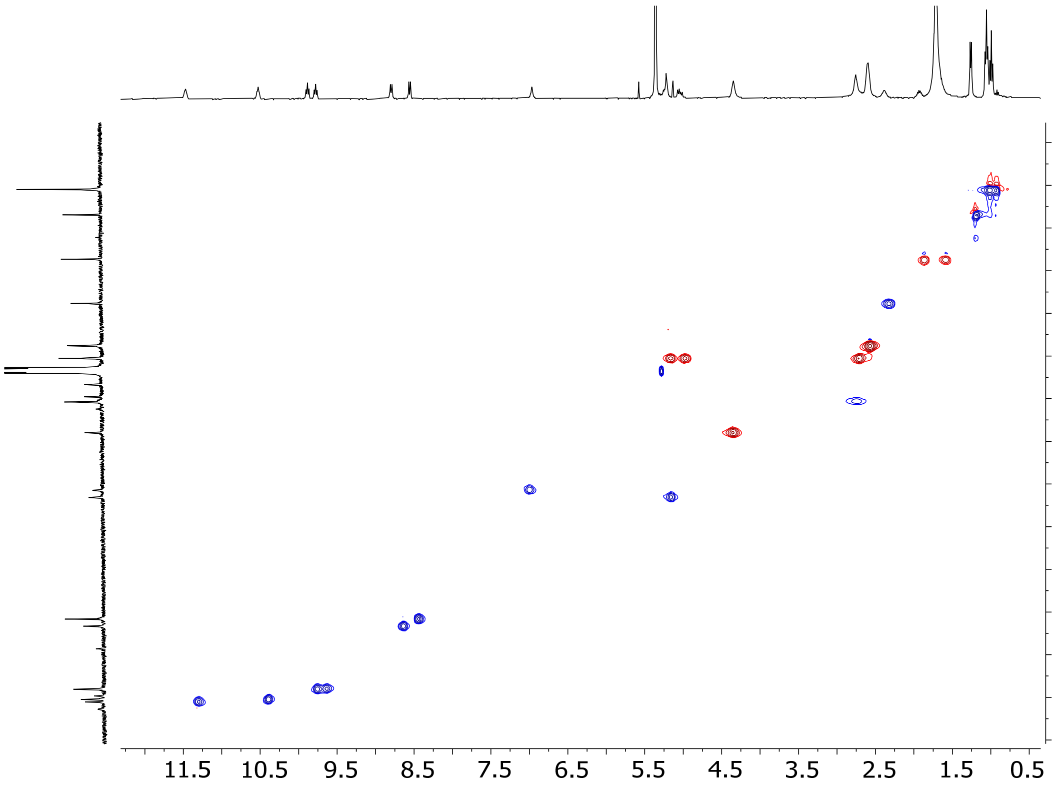

Figure A1-69. HSQC spectrum of [**L5**Eu(hfac)$_3$] in CD_2Cl_2.

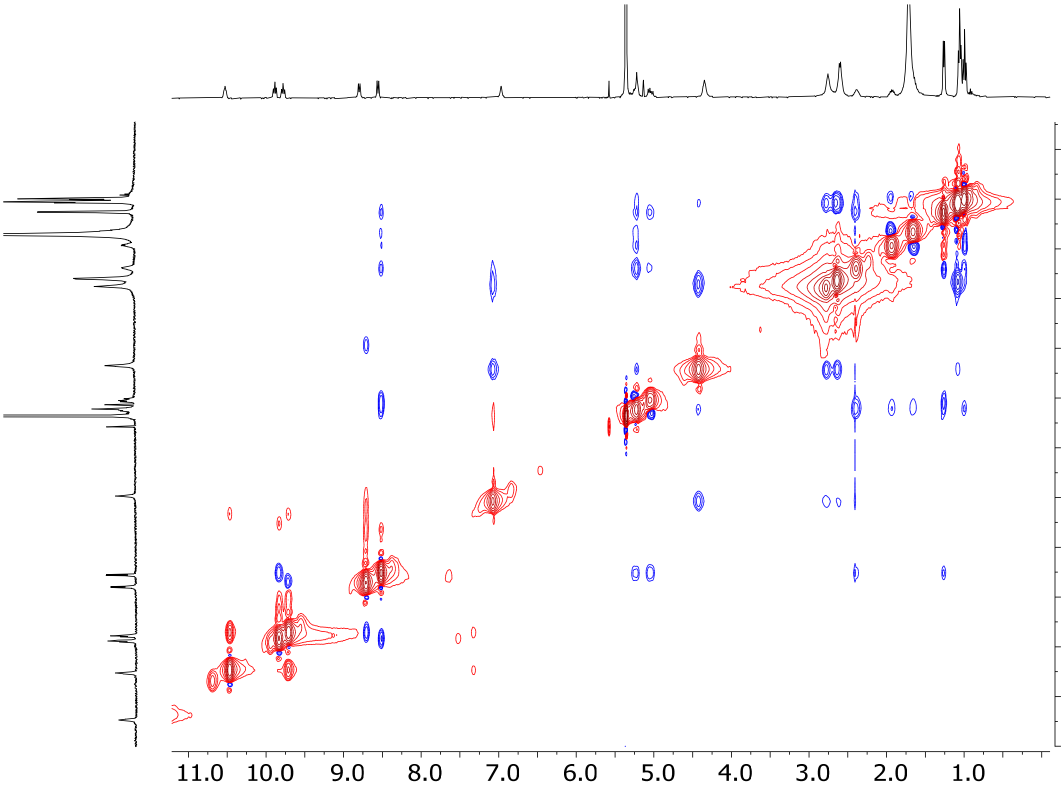

Figure A1-70. NOESY spectrum of [**L5**Eu(hfac)$_3$] in CD_2Cl_2.

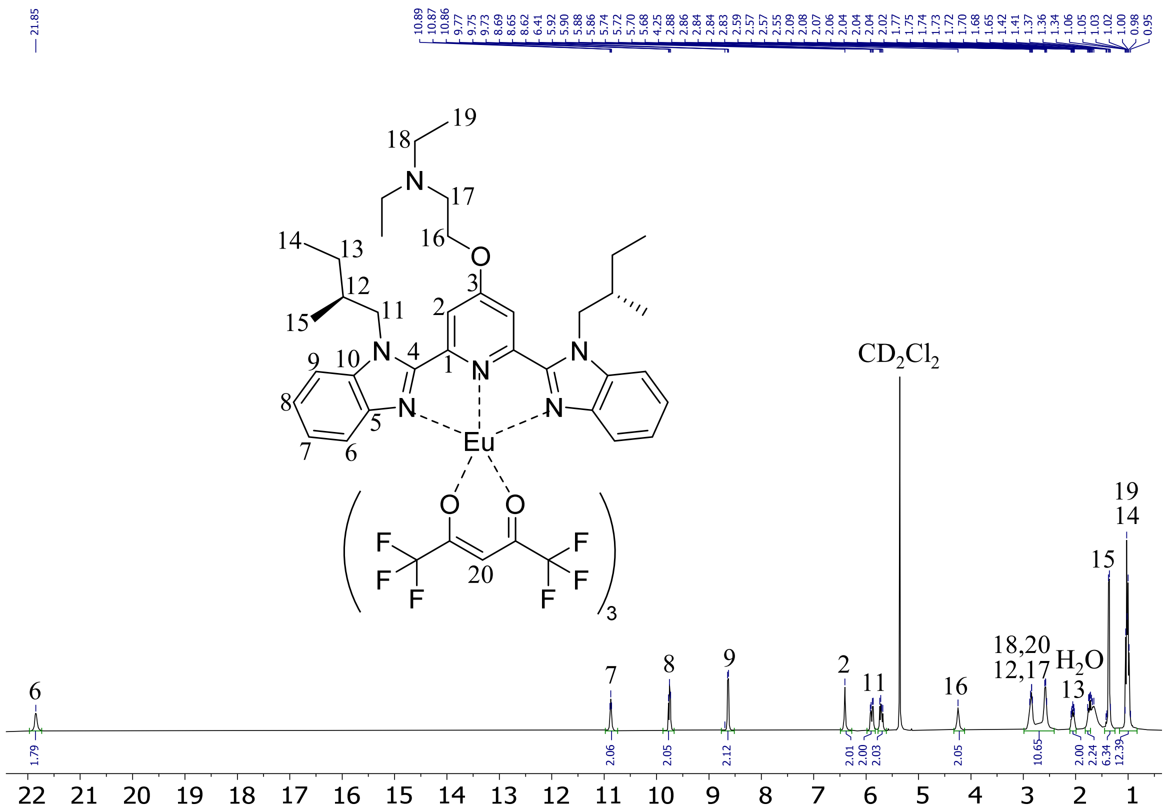

Figure A1-71. ^{1}H NMR spectrum of [**L6**Eu(hfac)$_3$] in CD_2Cl_2.

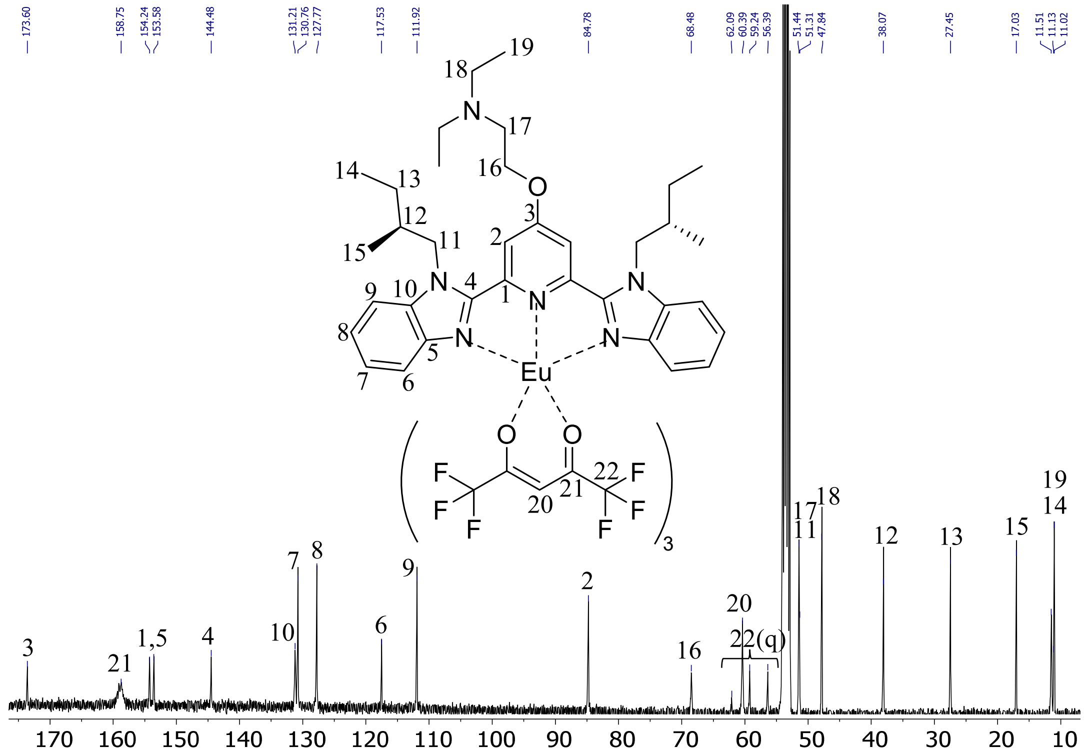

Figure A1-72. ^{13}C NMR spectrum of [**L6**Eu(hfac)$_3$] in CD_2Cl_2.

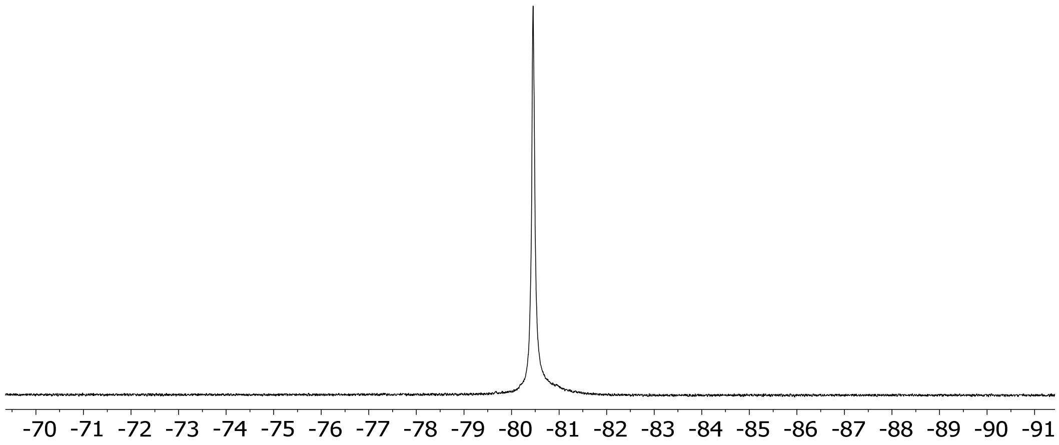

Figure A1-73. ^{19}F NMR spectrum of [**L6**Eu(hfac)$_3$] in CD_2Cl_2.

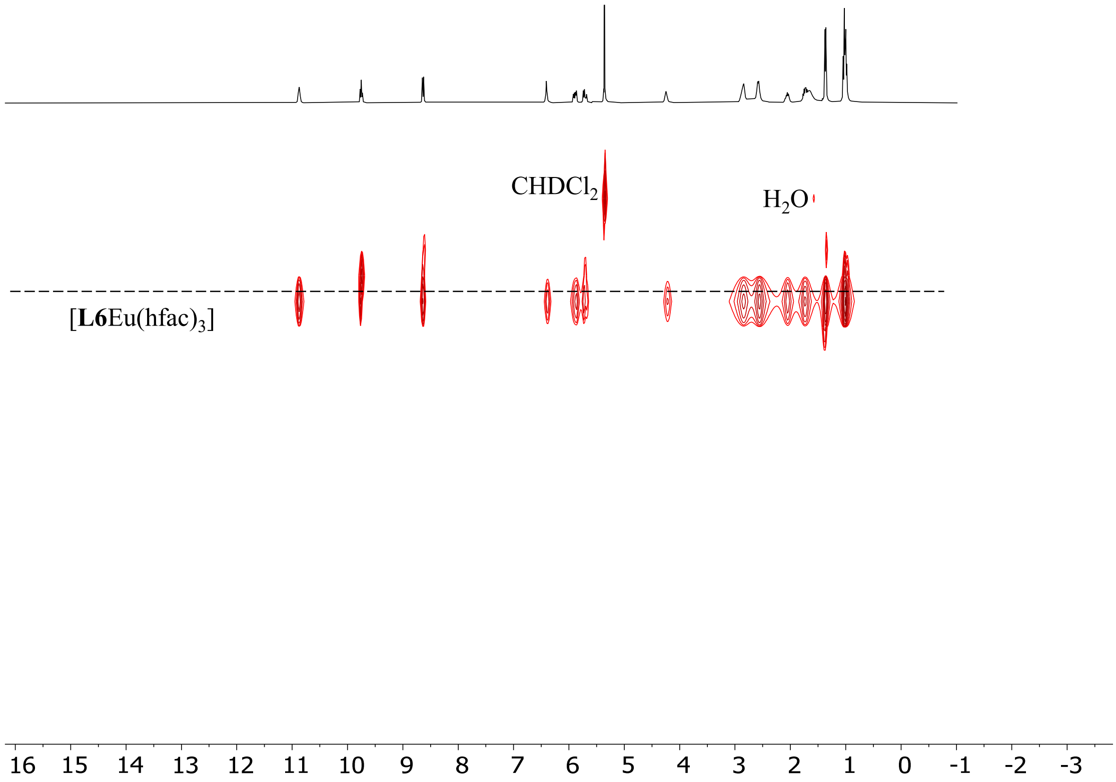

Figure A1-74. DOSY spectrum of [**L6**Eu(hfac)$_3$] in CD_2Cl_2.

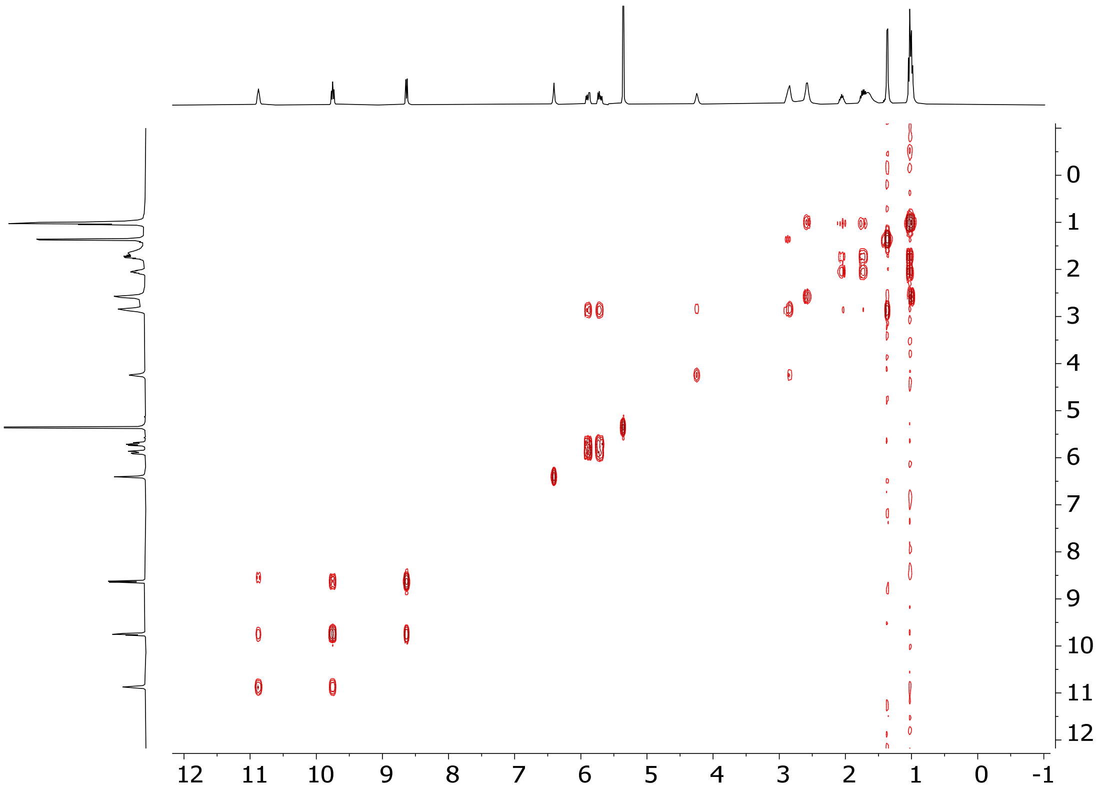

Figure A1-75. COSY spectrum of [**L6**Eu(hfac)$_3$] in CD_2Cl_2.

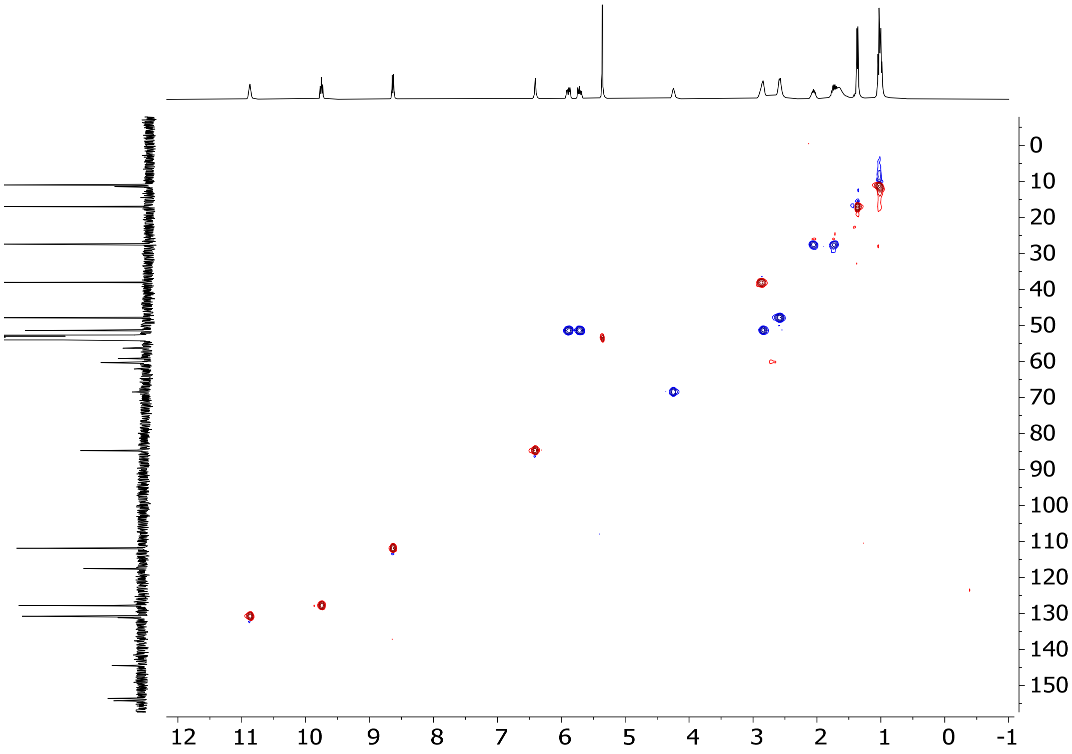

Figure A1-76. HSQC spectrum of [**L6**Eu(hfac)$_3$] in CD_2Cl_2.

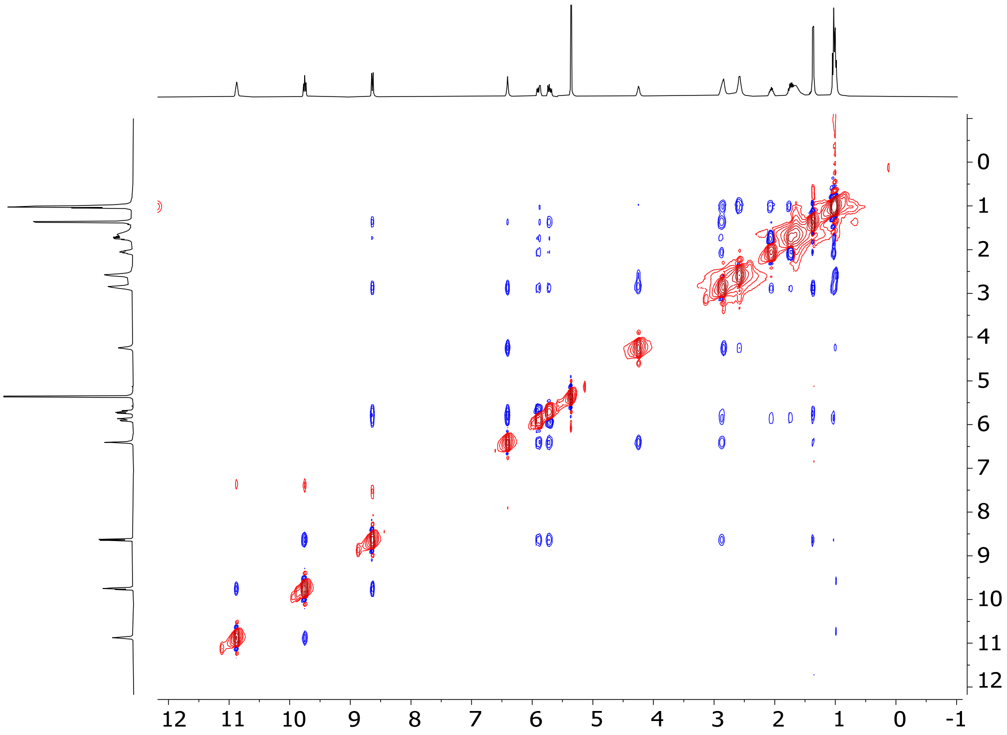

Figure A1-77. NOESY spectrum of [**L6**Eu(hfac)$_3$] in CD_2Cl_2.

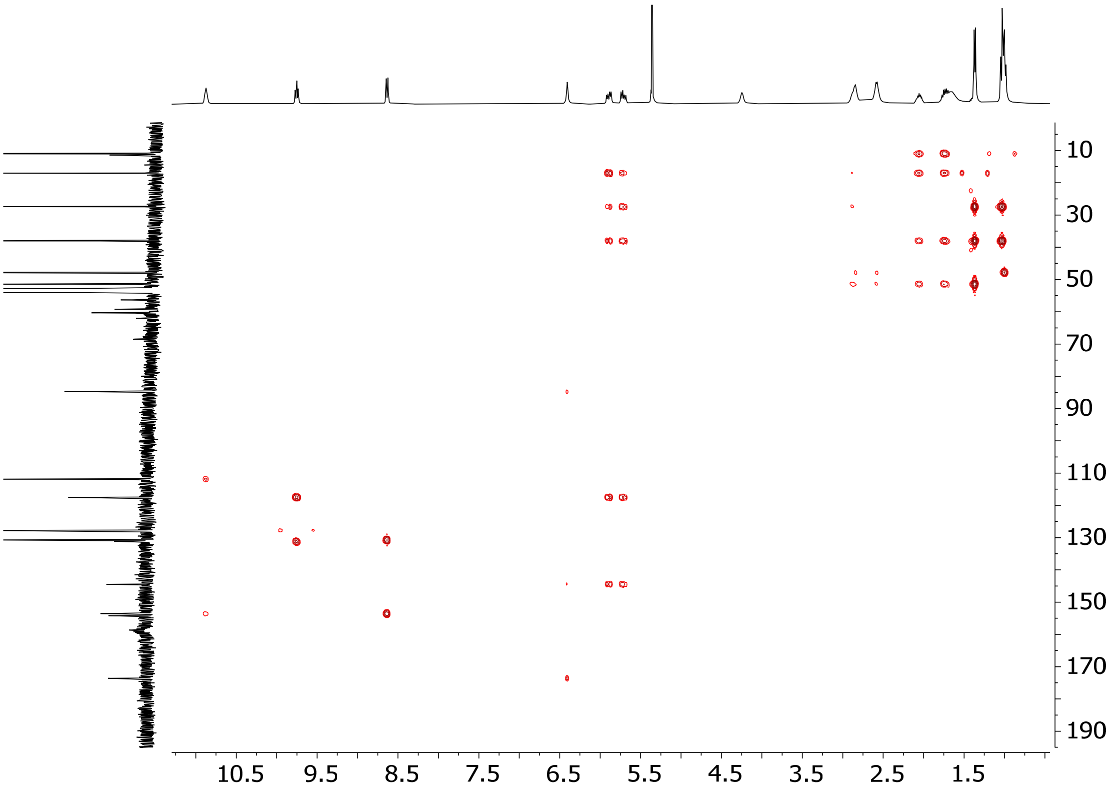

Figure A1-78. HMBC spectrum of [**L6**Eu(hfac)$_3$] in CD_2Cl_2.

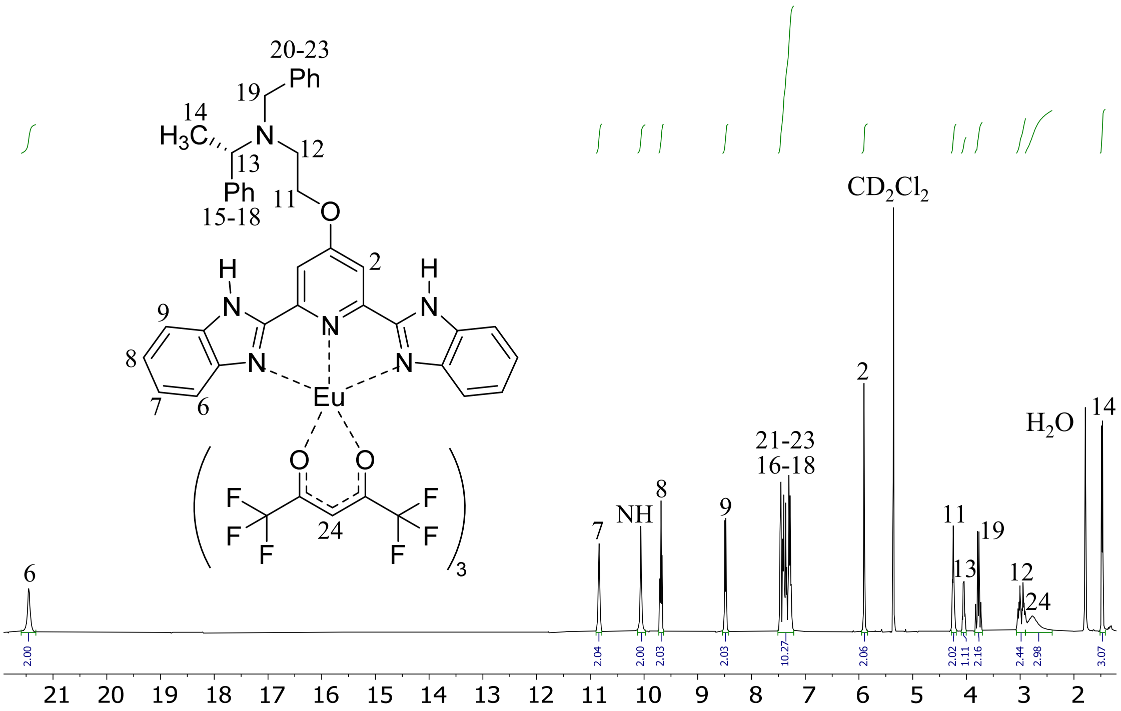

Figure A1-79. ^{1}H NMR spectrum of [**L7**Eu(hfac)$_3$] in CD_2Cl_2.

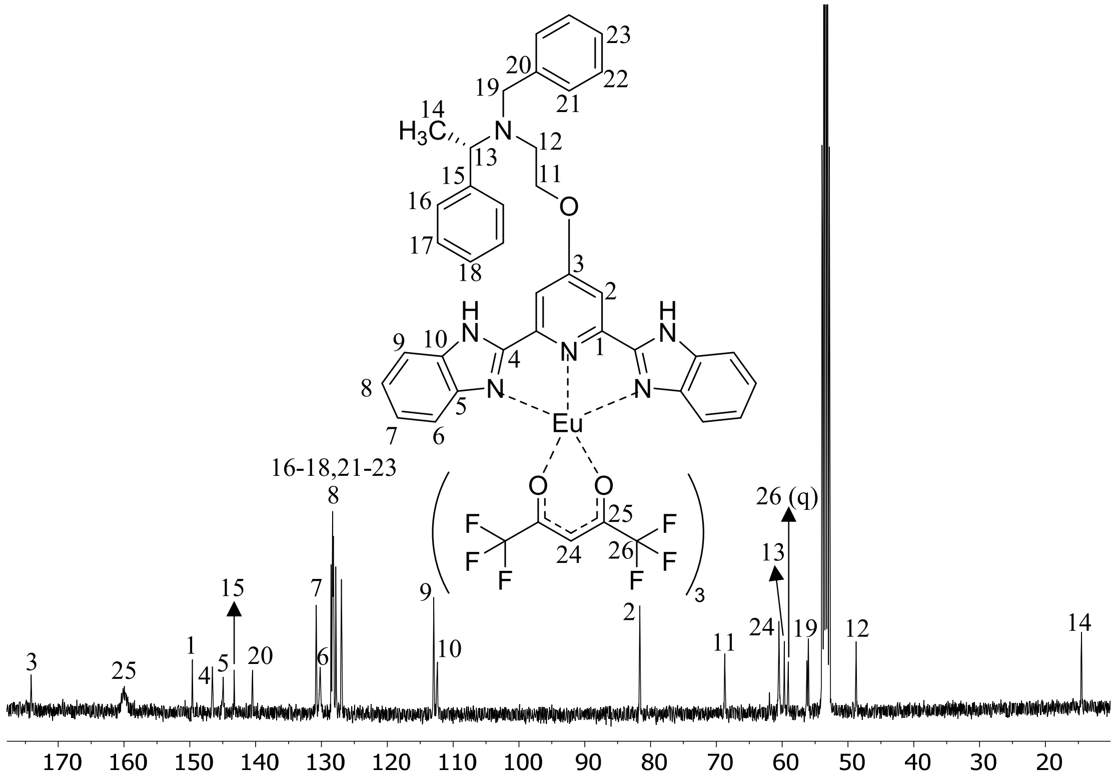

Figure A1-80. HSQC spectrum of [**L7**Eu(hfac)$_3$] in CD_2Cl_2.

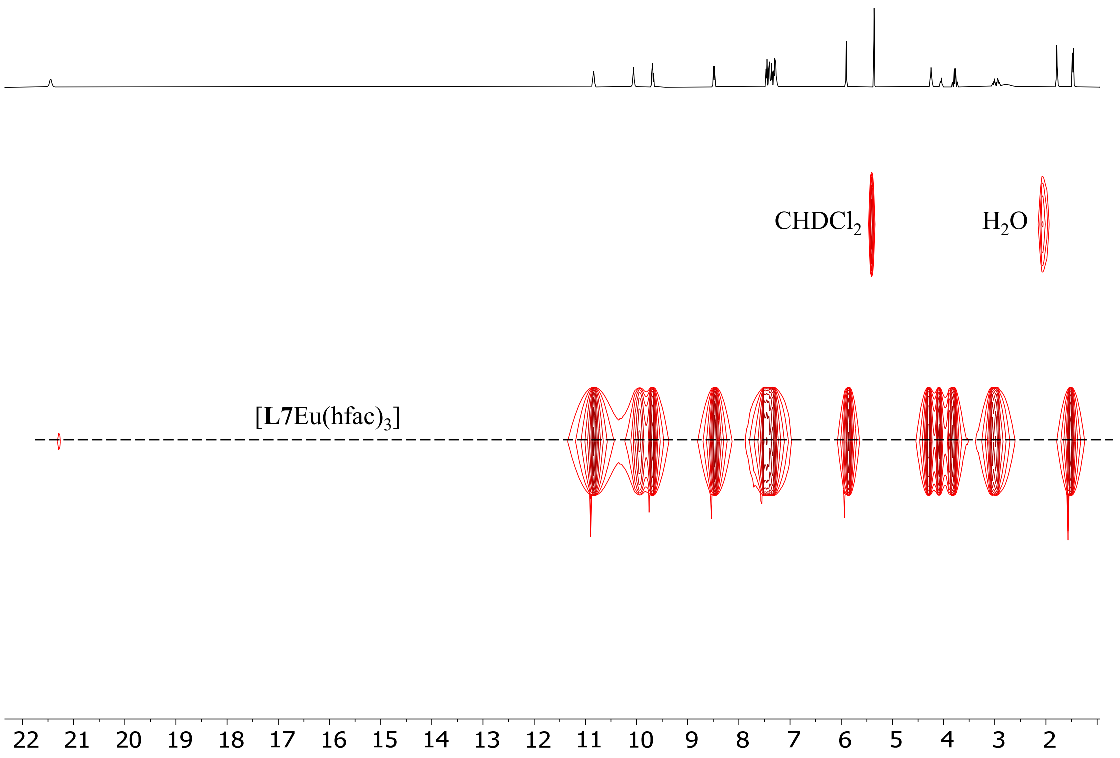

Figure A1-81. DOSY spectrum of [**L7**Eu(hfac)$_3$] in CD_2Cl_2.

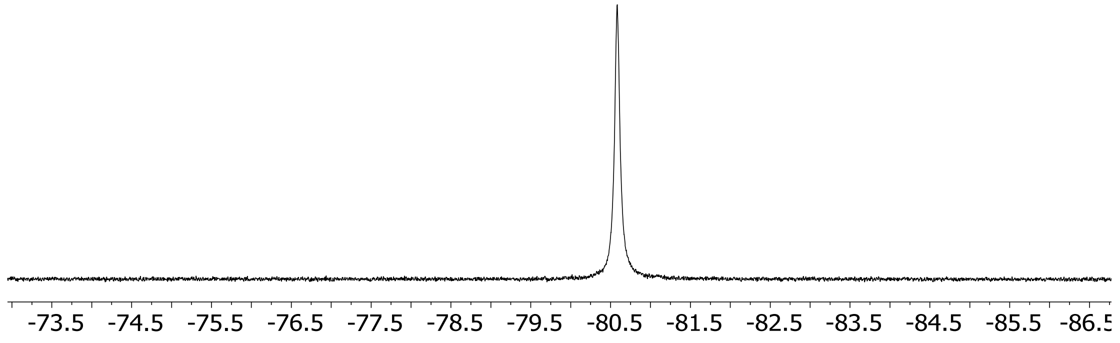

Figure A1-82. ^{19}F NMR spectrum of [**L7**Eu(hfac)$_3$] in CD_2Cl_2.

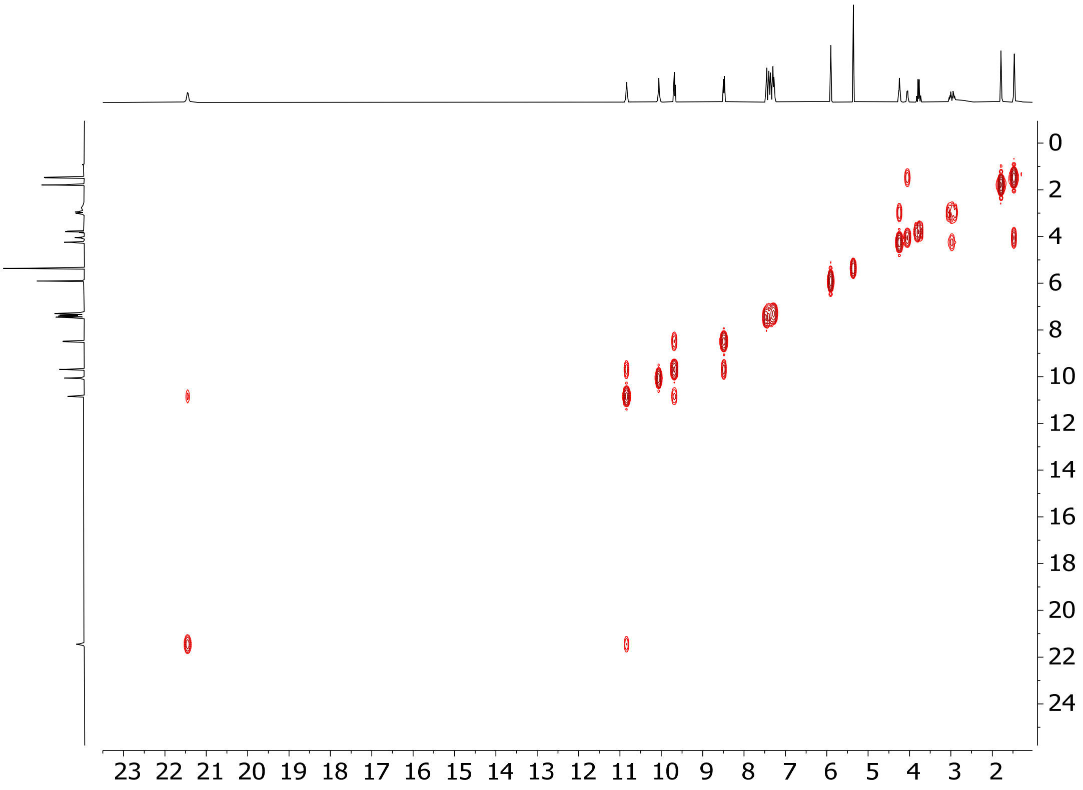

Figure A1-83. COSY spectrum of [**L7**Eu(hfac)$_3$] in CD_2Cl_2.

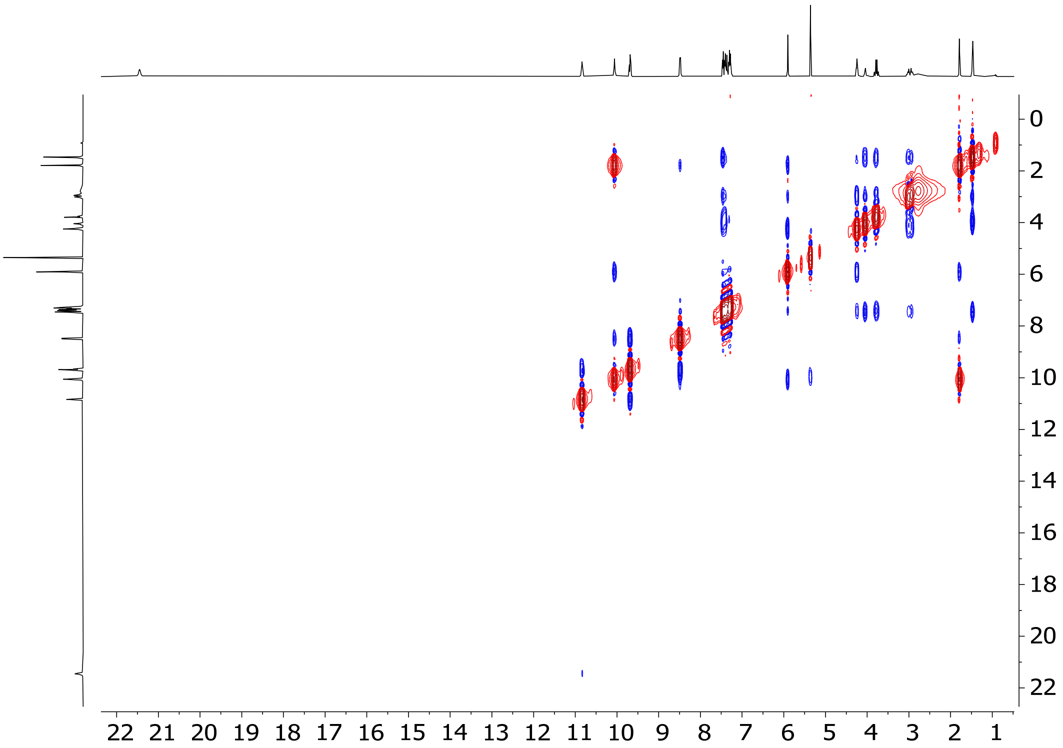

Figure A1-84. NOESY spectrum of [**L7**Eu(hfac)$_3$] in CD_2Cl_2.

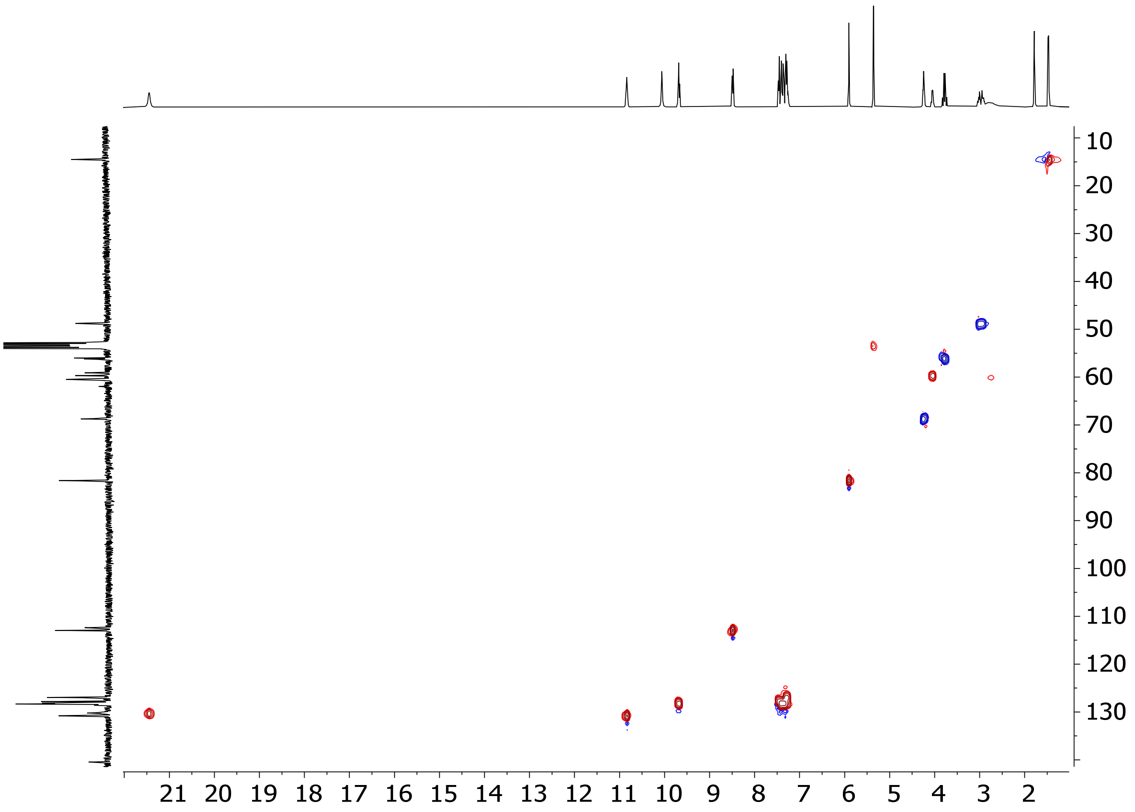

Figure A1-85. HSQC spectrum of [**L7**Eu(hfac)$_3$] in CD_2Cl_2.

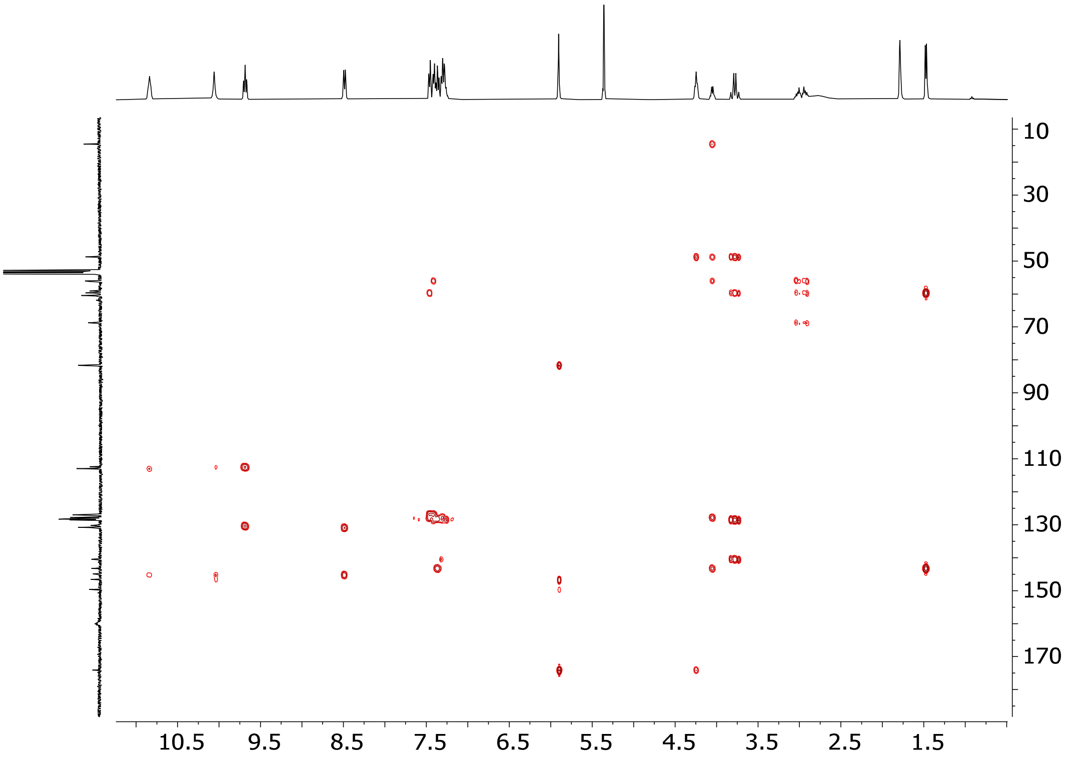

Figure A1-86. HMBC spectrum of [**L7**Eu(hfac)$_3$] in CD_2Cl_2.

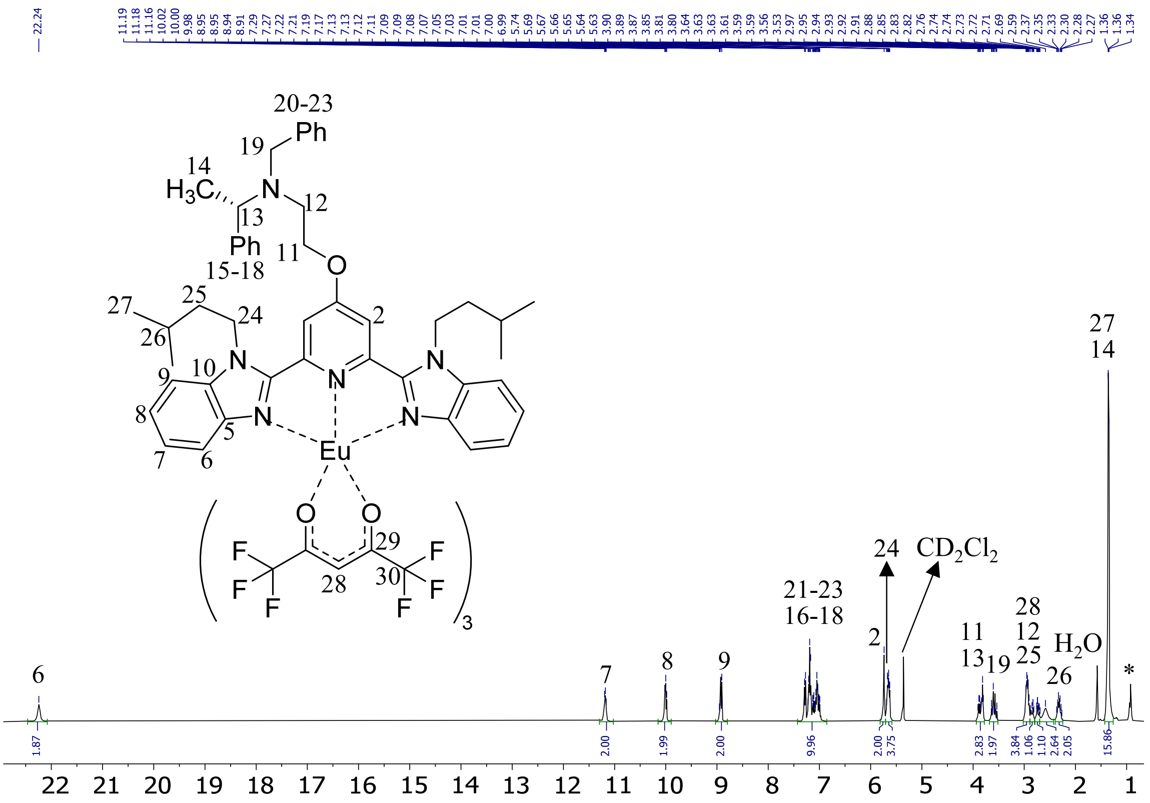

Figure A1-87. ^{1}H NMR spectrum of [**L7**Eu(hfac)3] in CD2Cl2 (*pentane residue).

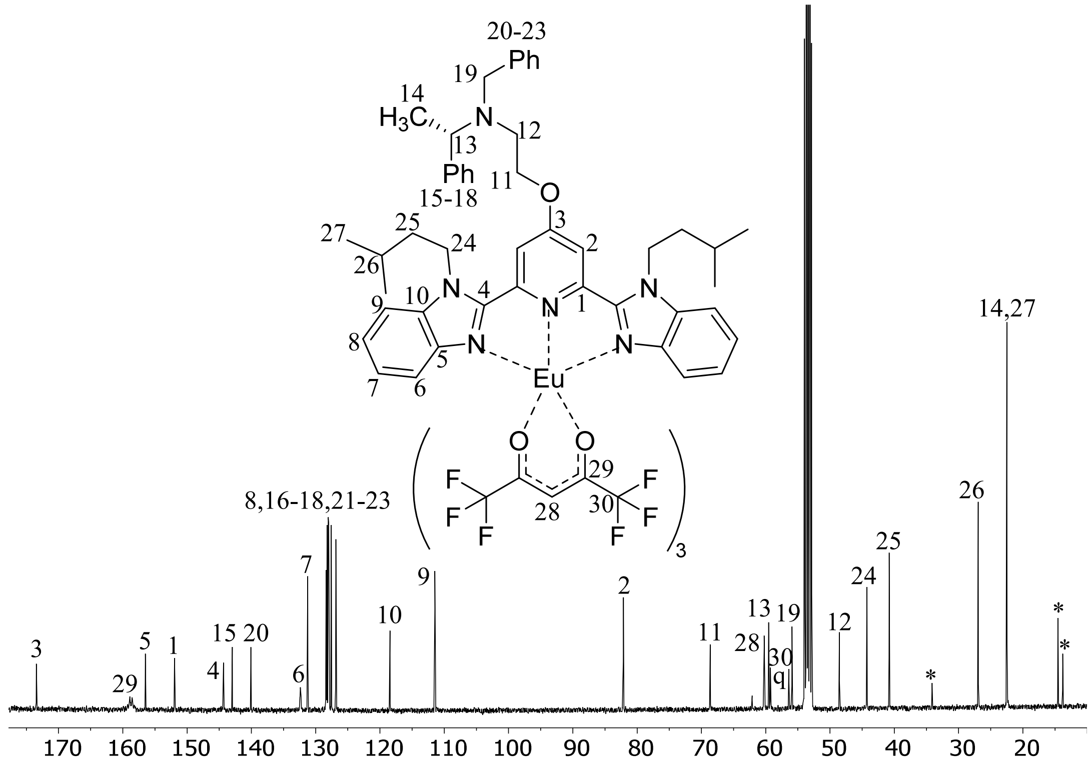

Figure A1-88. ^{13}C NMR spectrum of [**L8**Eu(hfac)3] in (*pentane residue).

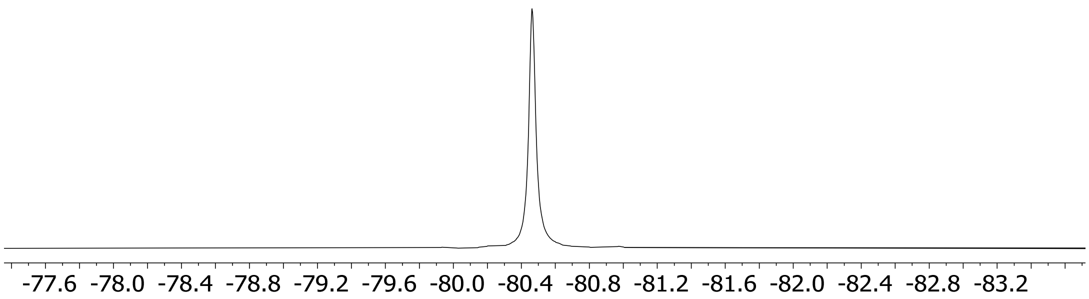

Figure A1-89. ^{19}F NMR spectrum of [**L8**Eu(hfac)$_3$] in CD_2Cl_2.

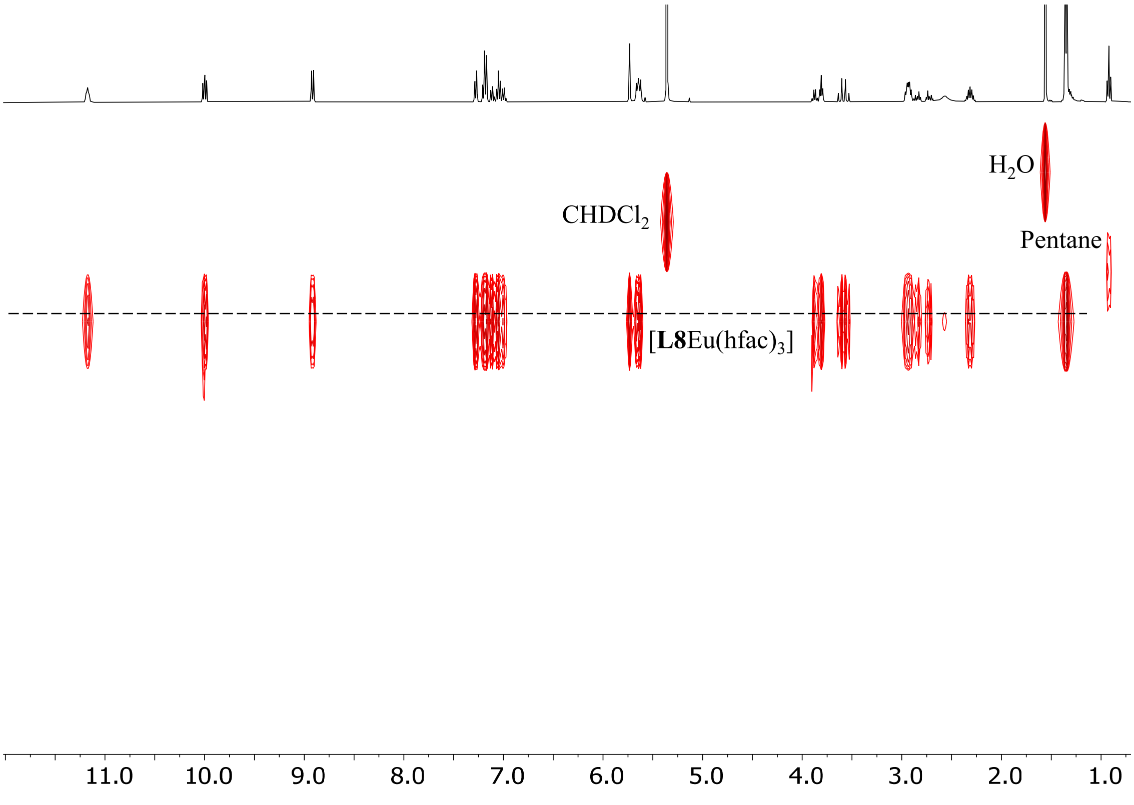

Figure A1-90. DOSY spectrum of [**L8**Eu(hfac)$_3$] in CD_2Cl_2.

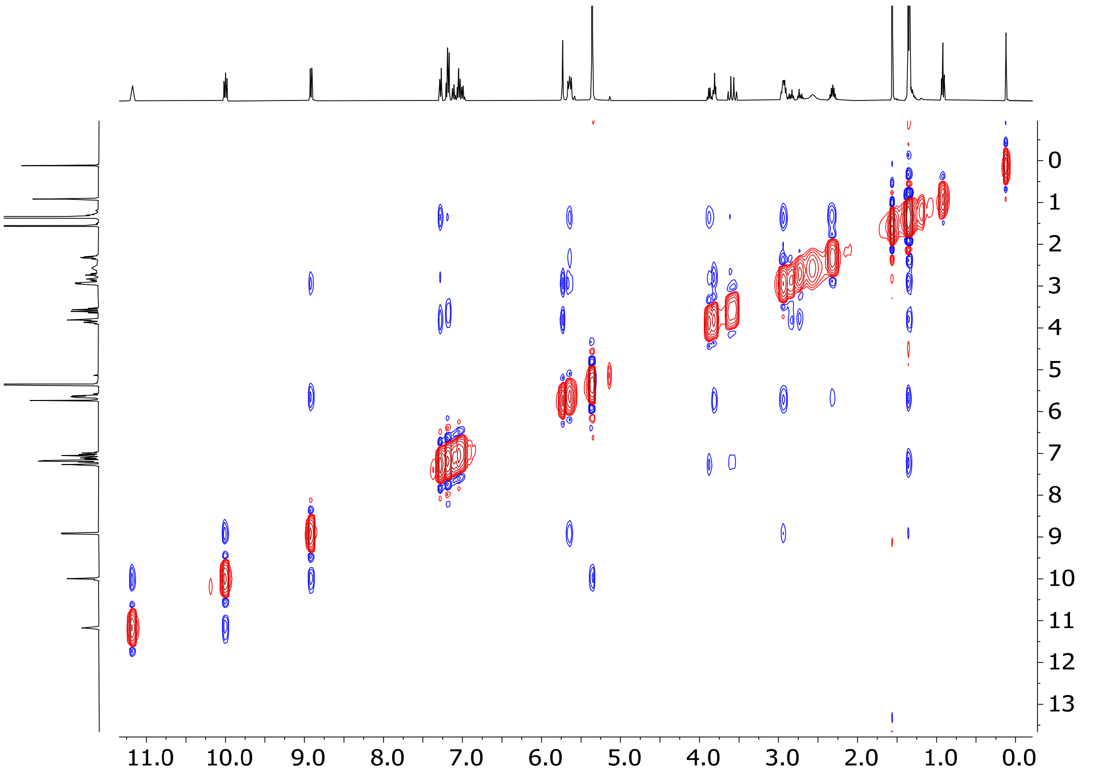

Figure A1-91. NOESY spectrum of [**L8**Eu(hfac)$_3$] in CD_2Cl_2.

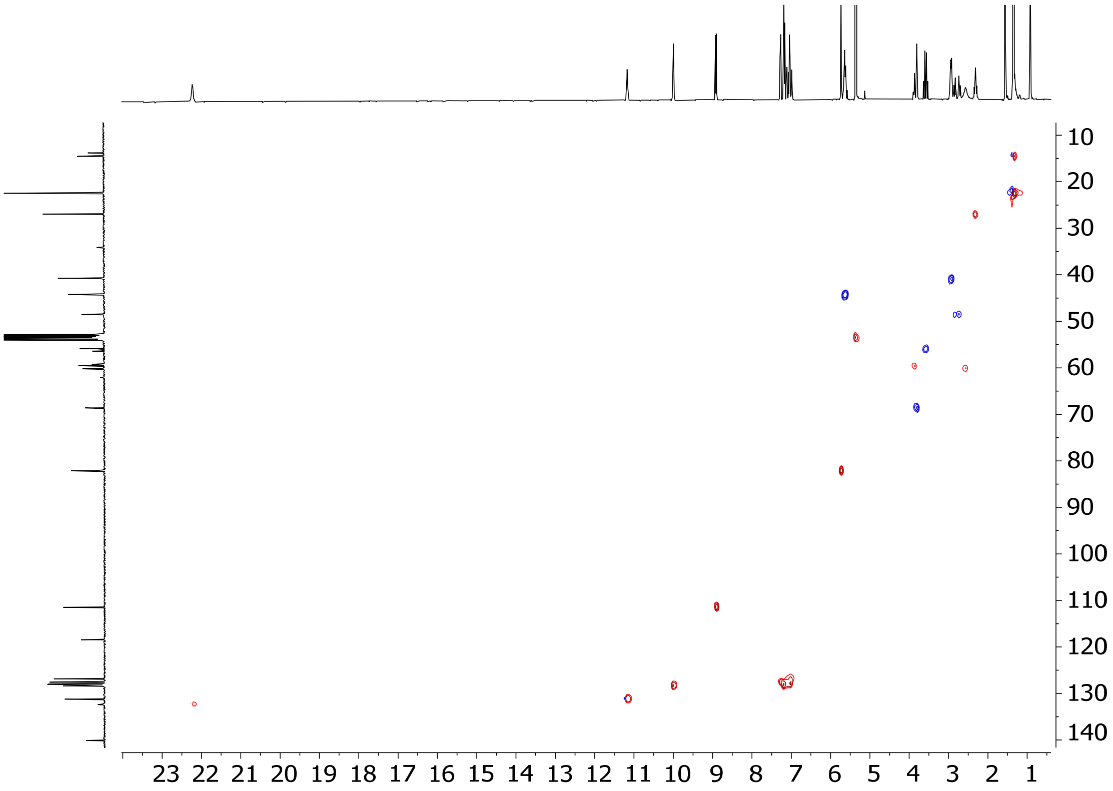

Figure A1-92. HSQC spectrum of [**L8**Eu(hfac)$_3$] in CD_2Cl_2.

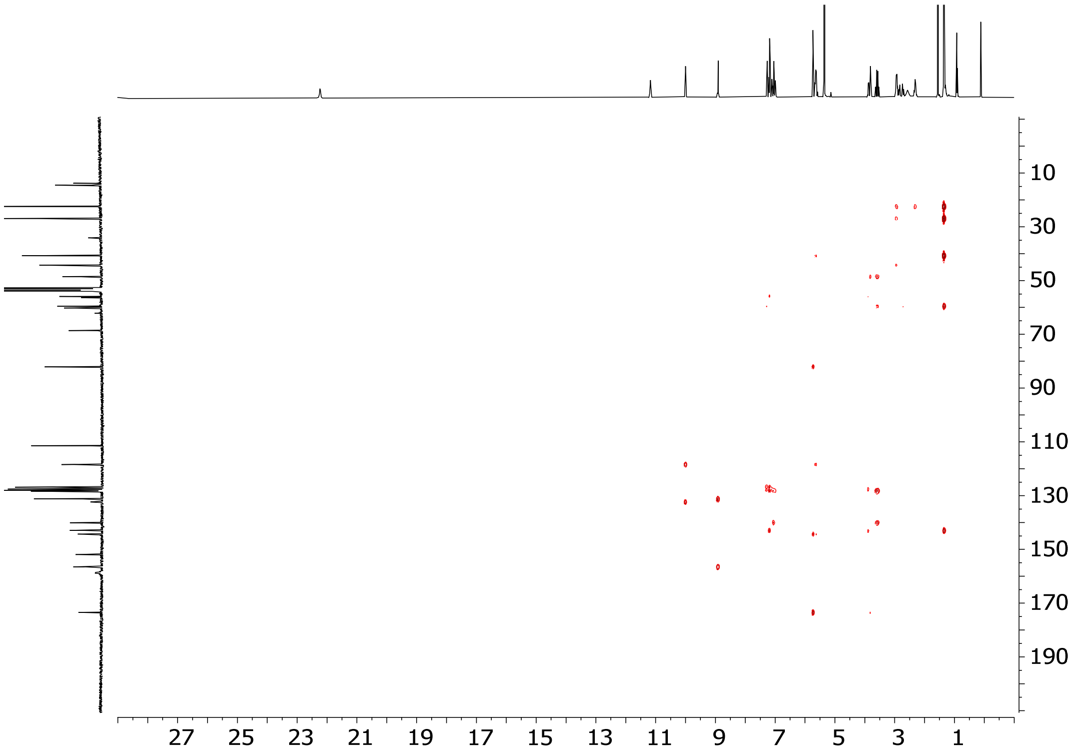

Figure A1-93. HMBC spectrum of [**L8**Eu(hfac)$_3$] in CD_2Cl_2.

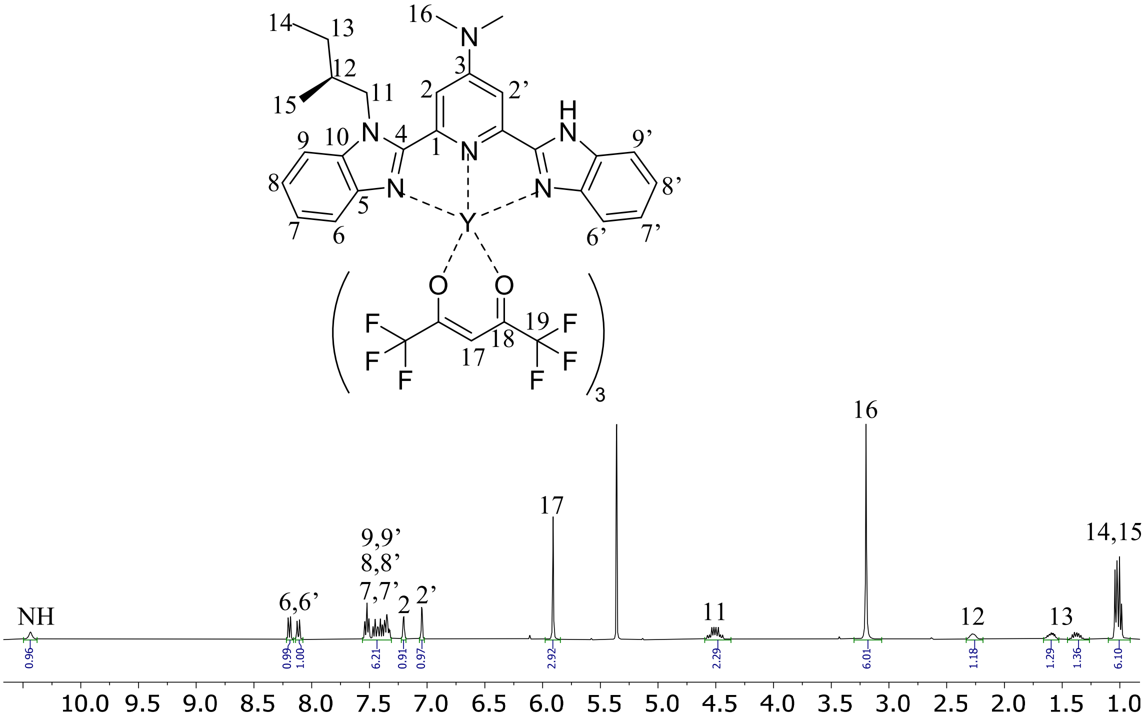

Figure A1-94. ^{1}H NMR spectrum of [**L2**Y(hfac)$_3$] in CD_2Cl_2.

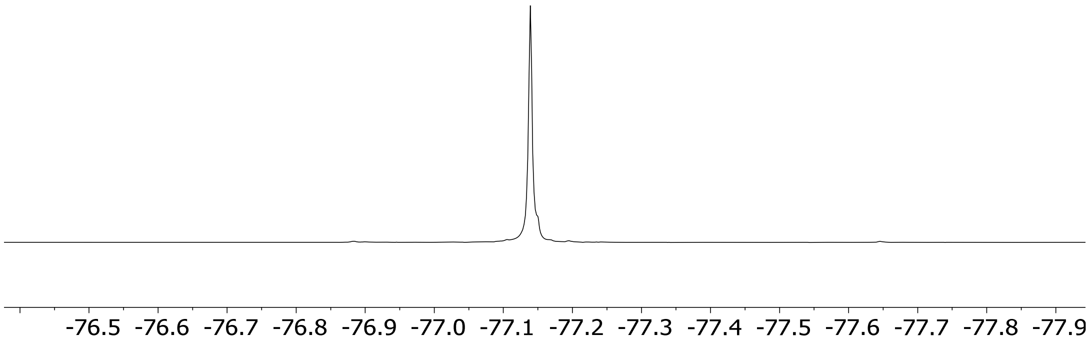

Figure A1-95. ^{19}F NMR spectrum of [**L2**Y(hfac)$_3$] in CD_2Cl_2.

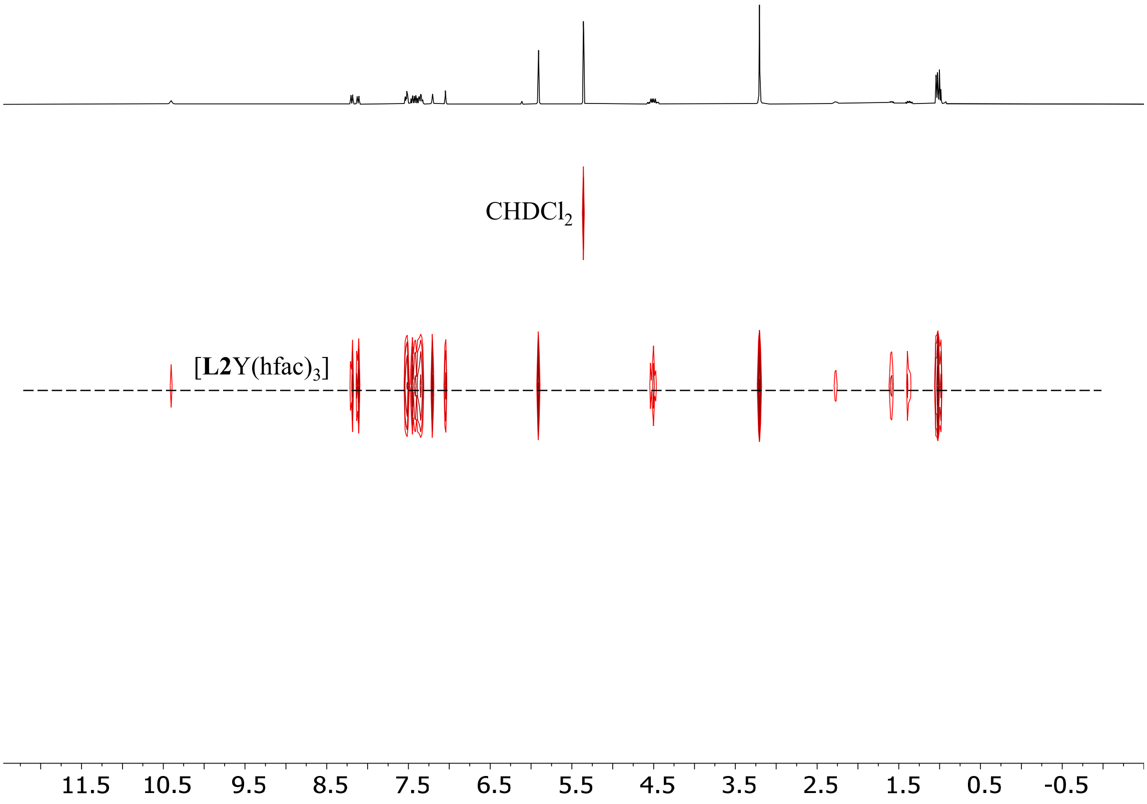

Figure A1-96. DOSY spectrum of [**L2**Y(hfac)$_3$] in CD_2Cl_2

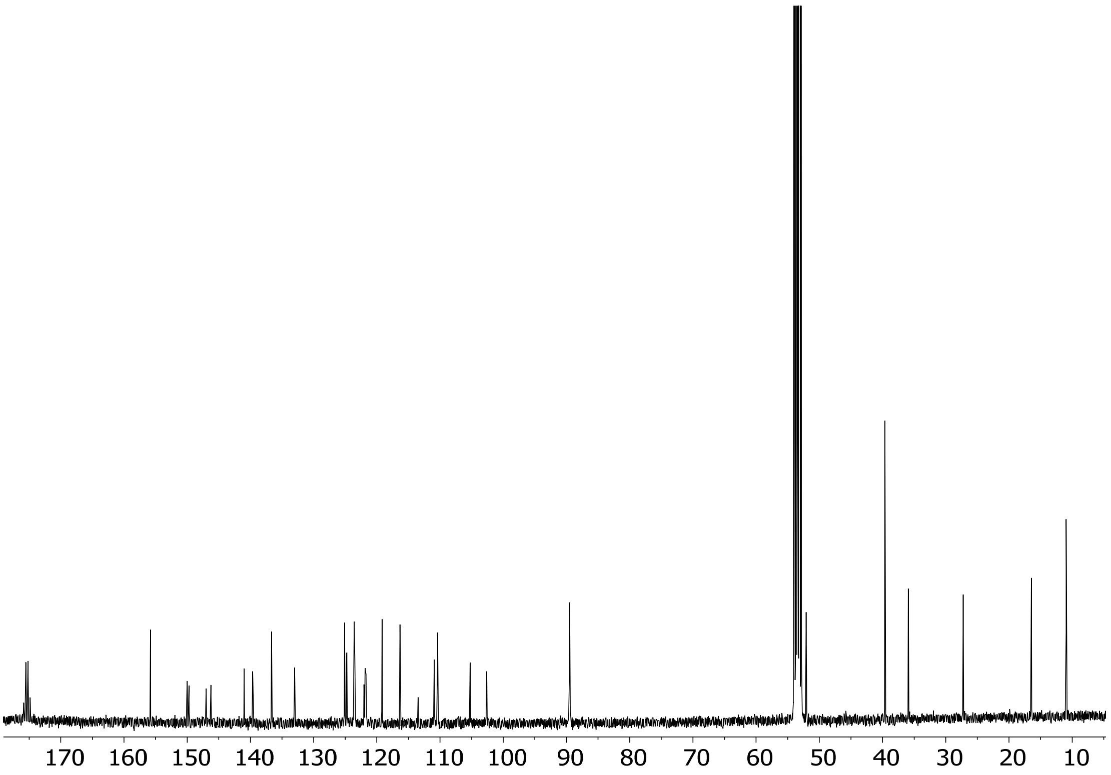

Figure A1-97. ^{13}C NMR spectrum of [**L2**Y(hfac)$_3$] in CD_2Cl_2.

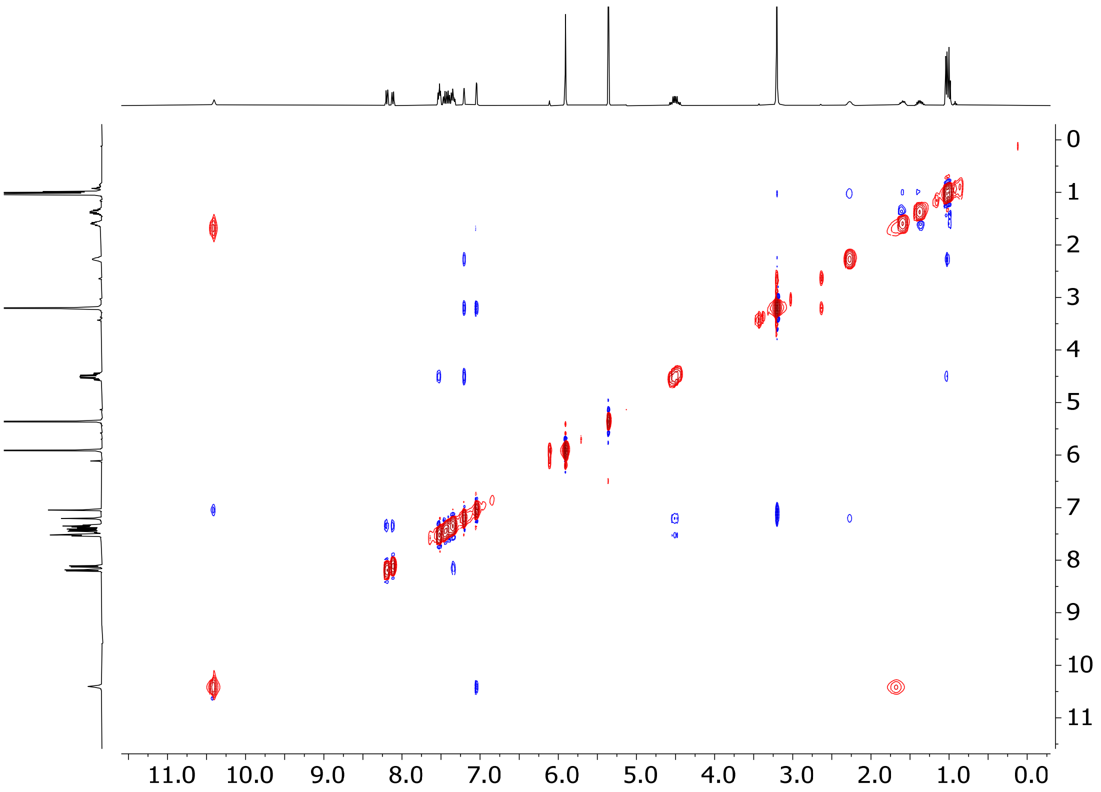

Figure A1-98. COSY spectrum of [**L2**Y(hfac)$_3$] in CD_2Cl_2.

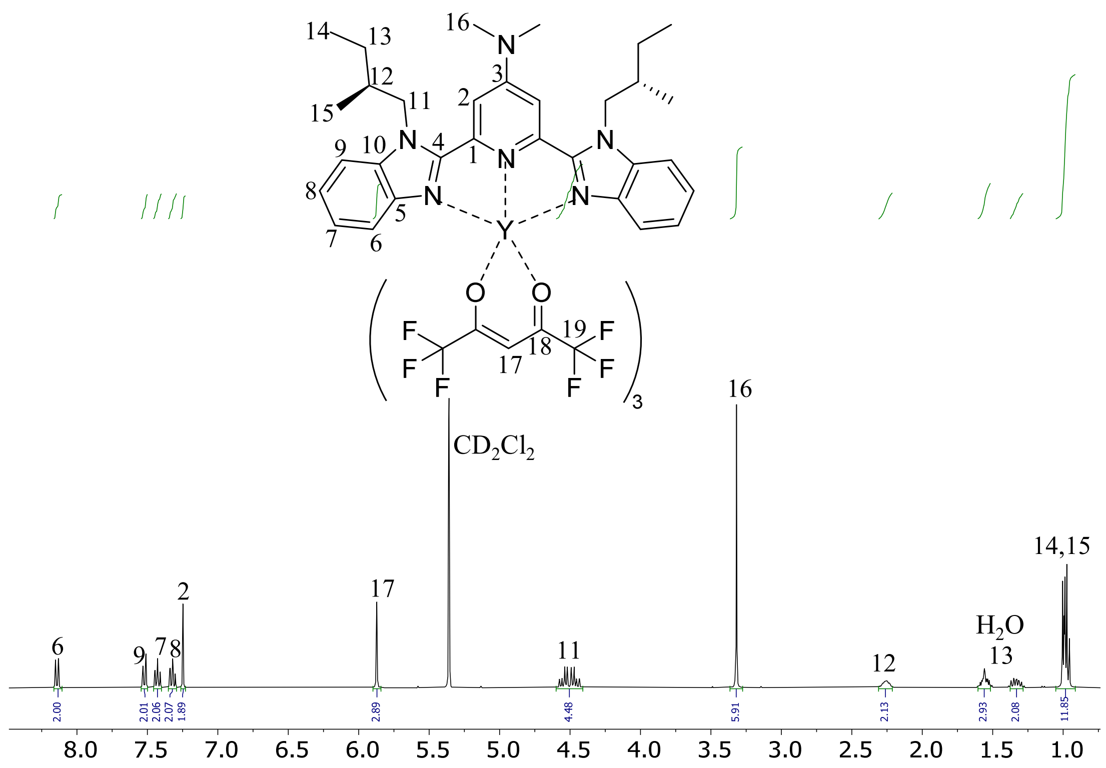

Figure A1-99. ^{1}H NMR spectrum of [**L3**Y(hfac)$_3$] in CD_2Cl_2.

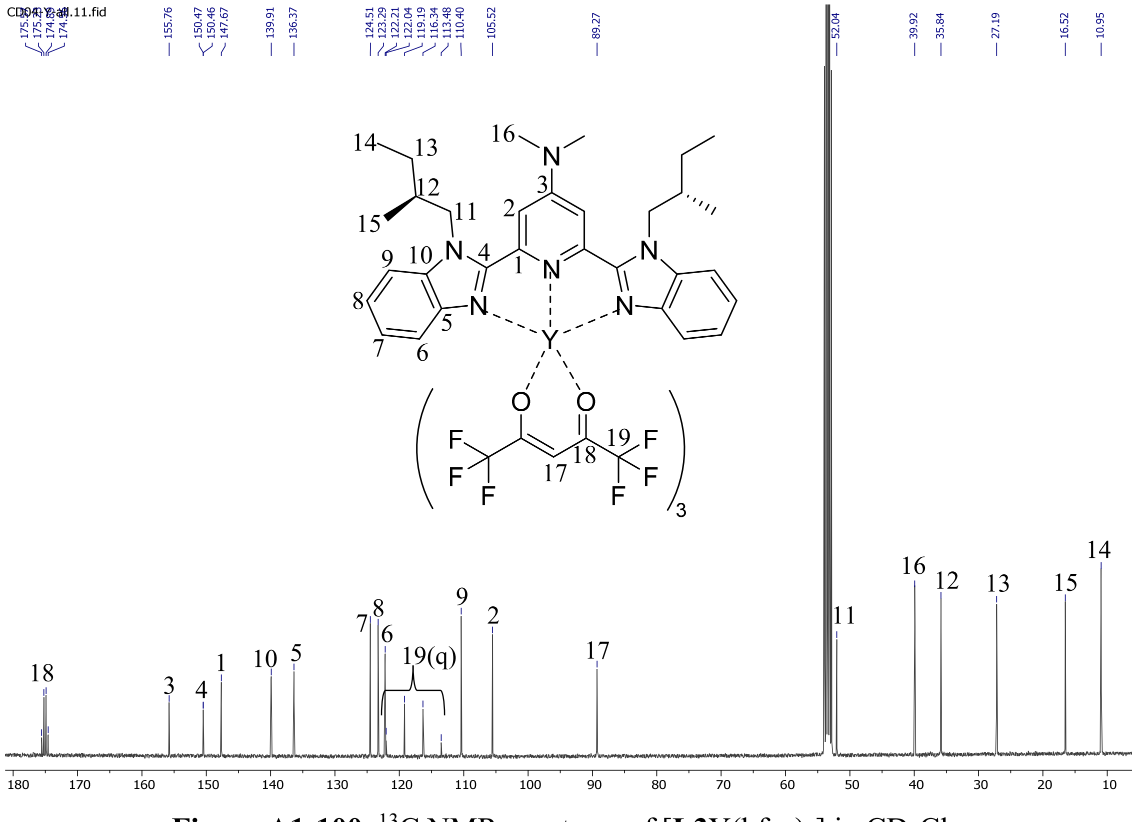

Figure A1-100. ^{13}C NMR spectrum of [**L3**Y(hfac)$_3$] in CD_2Cl_2.

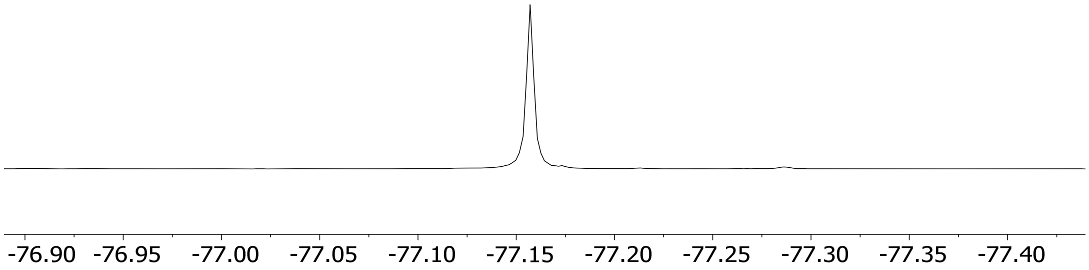

Figure A1-101. ^{19}F NMR spectrum of [**L3**Y(hfac)$_3$] in CD_2Cl_2.

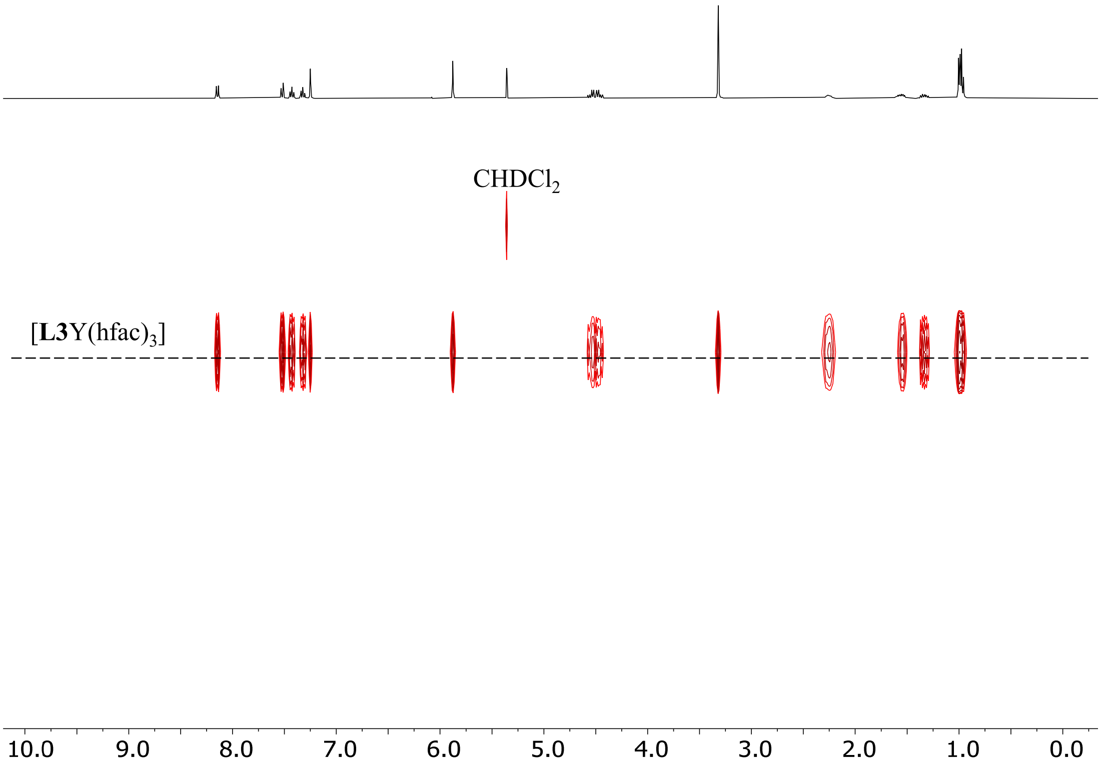

Figure A1-102. DOSY spectrum of [**L3**Y(hfac)$_3$] in CD_2Cl_2.

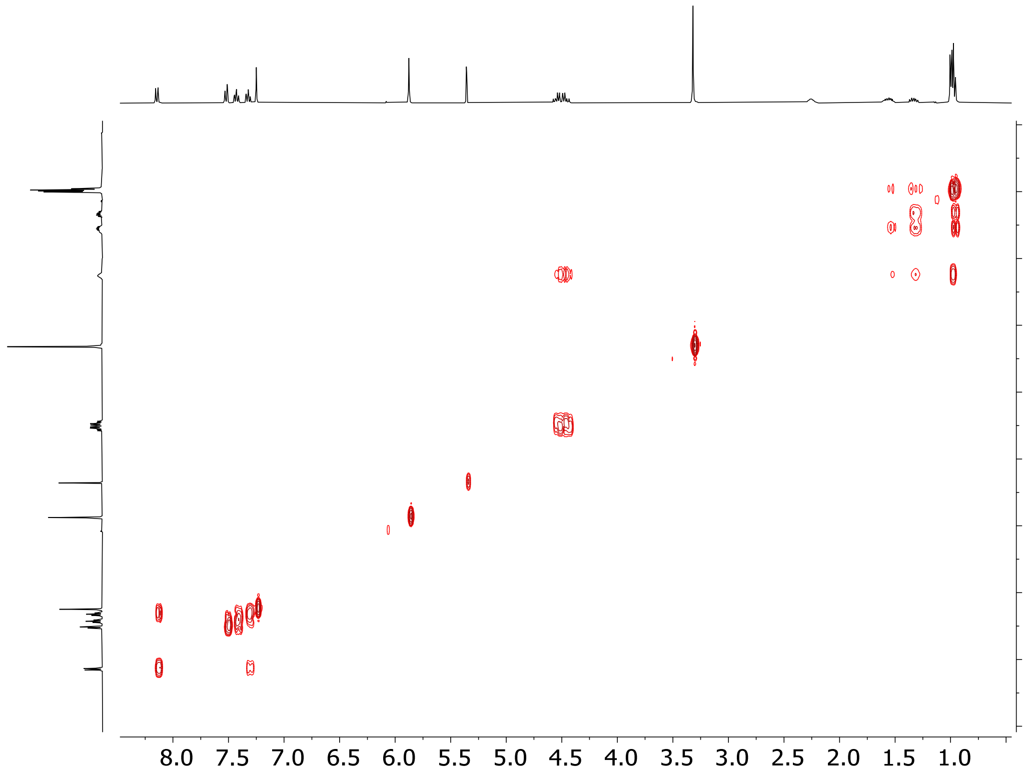

Figure A1-103. COSY spectrum of [**L3**Y(hfac)$_3$] in CD_2Cl_2.

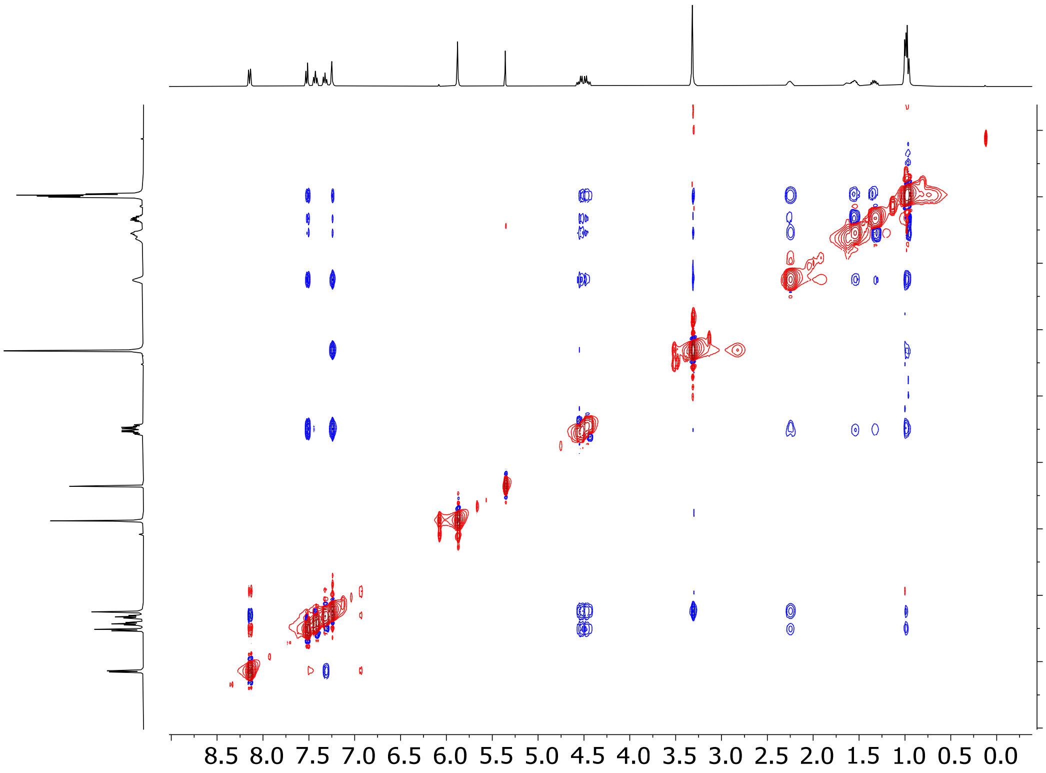

Figure A1-104. NOESY spectrum of [**L3**Y(hfac)$_3$] in CD_2Cl_2.

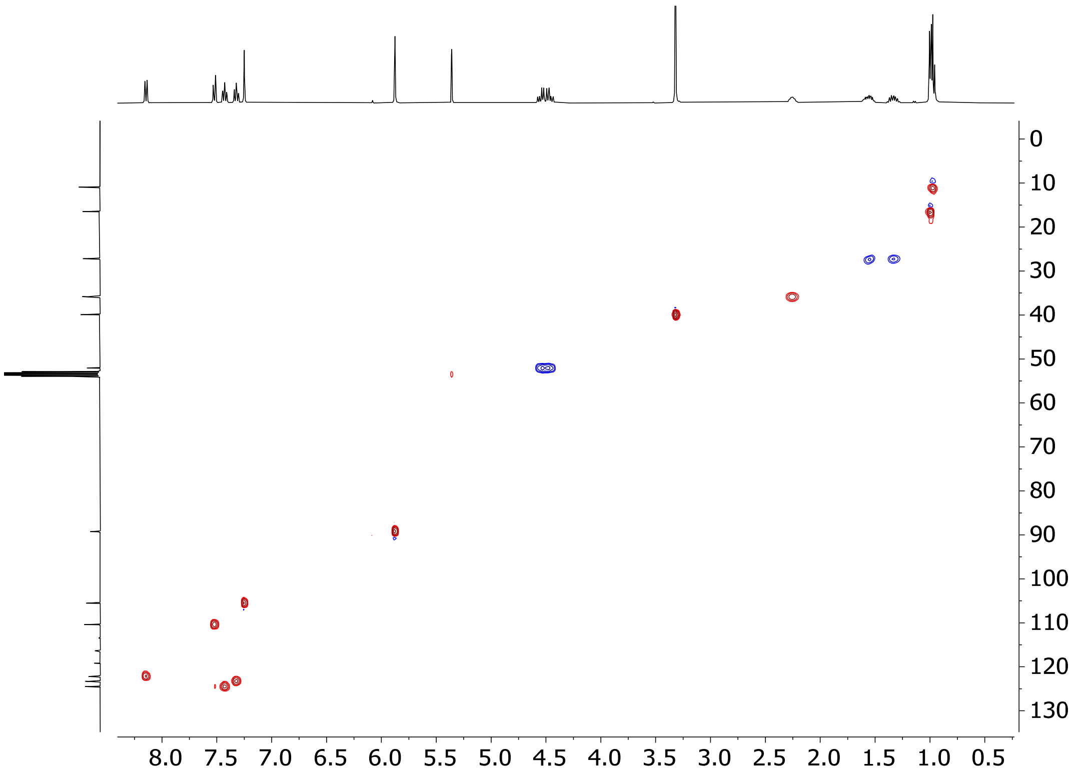

Figure A1-105. HSQC spectrum of [**L3**Y(hfac)$_3$] in CD_2Cl_2.

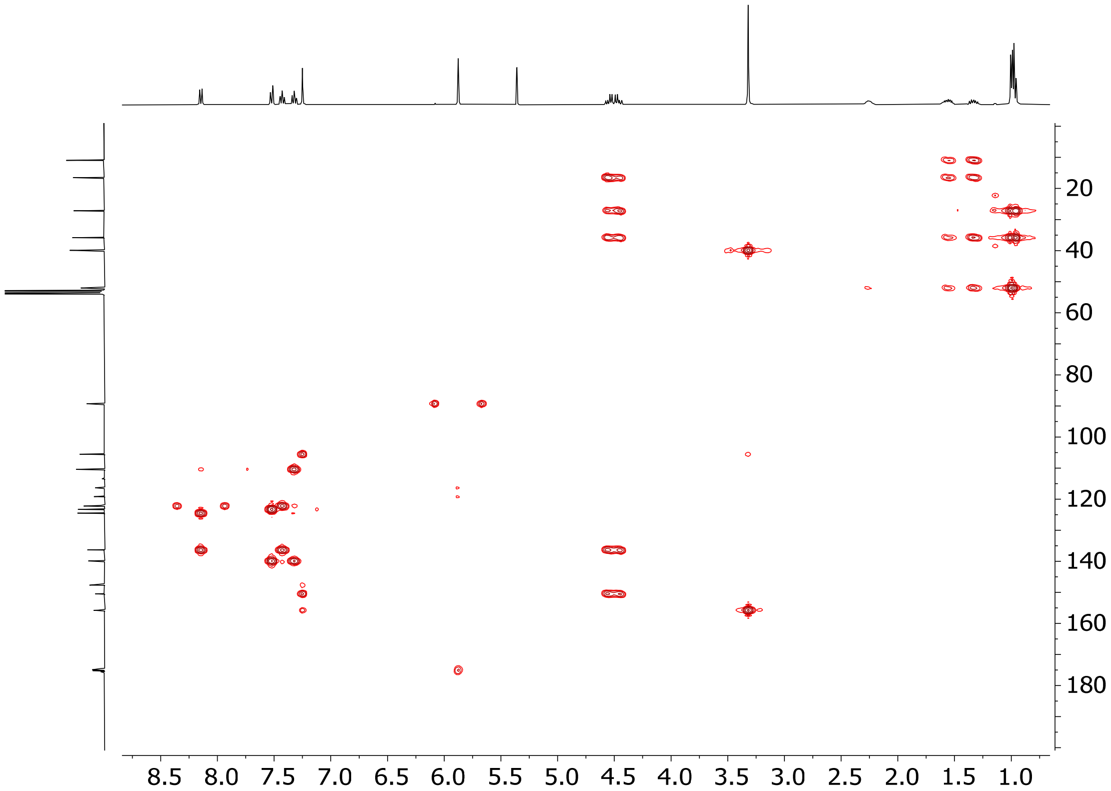

Figure A1-106. HMBC spectrum of [**L3**Y(hfac)$_3$] in CD_2Cl_2.

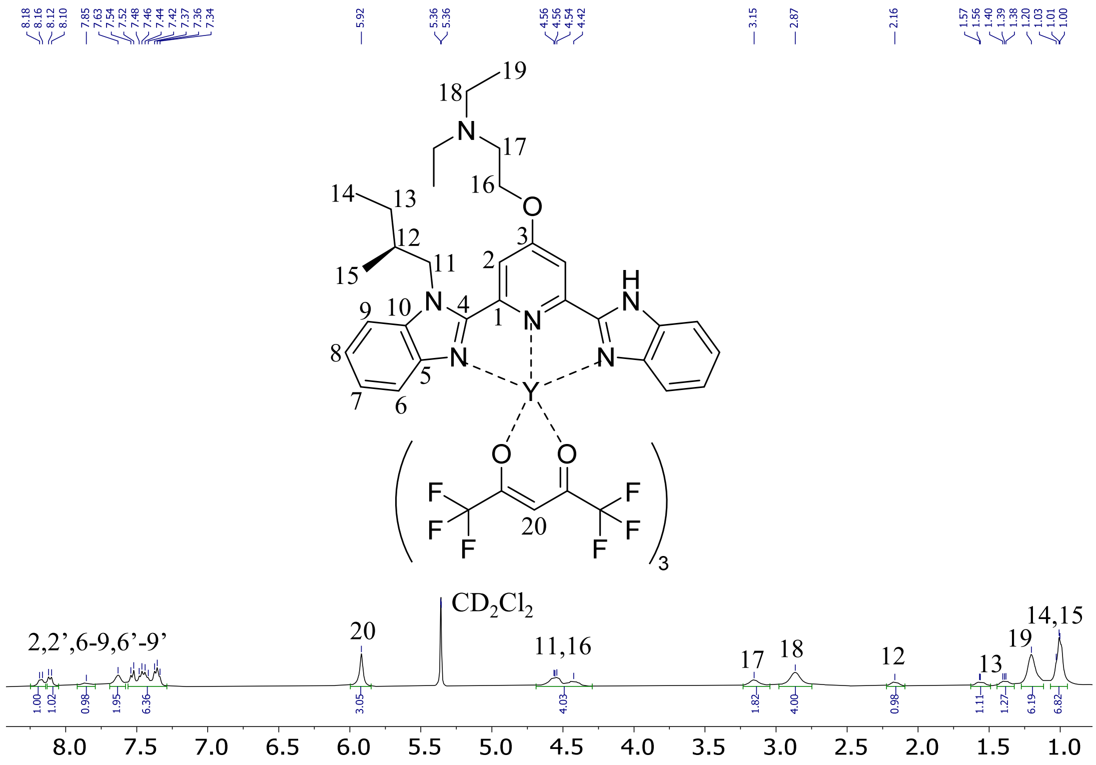

Figure A1-107. ^{1}H NMR spectrum of [**L5**Y(hfac)$_3$] in CD$_2$Cl$_2$.

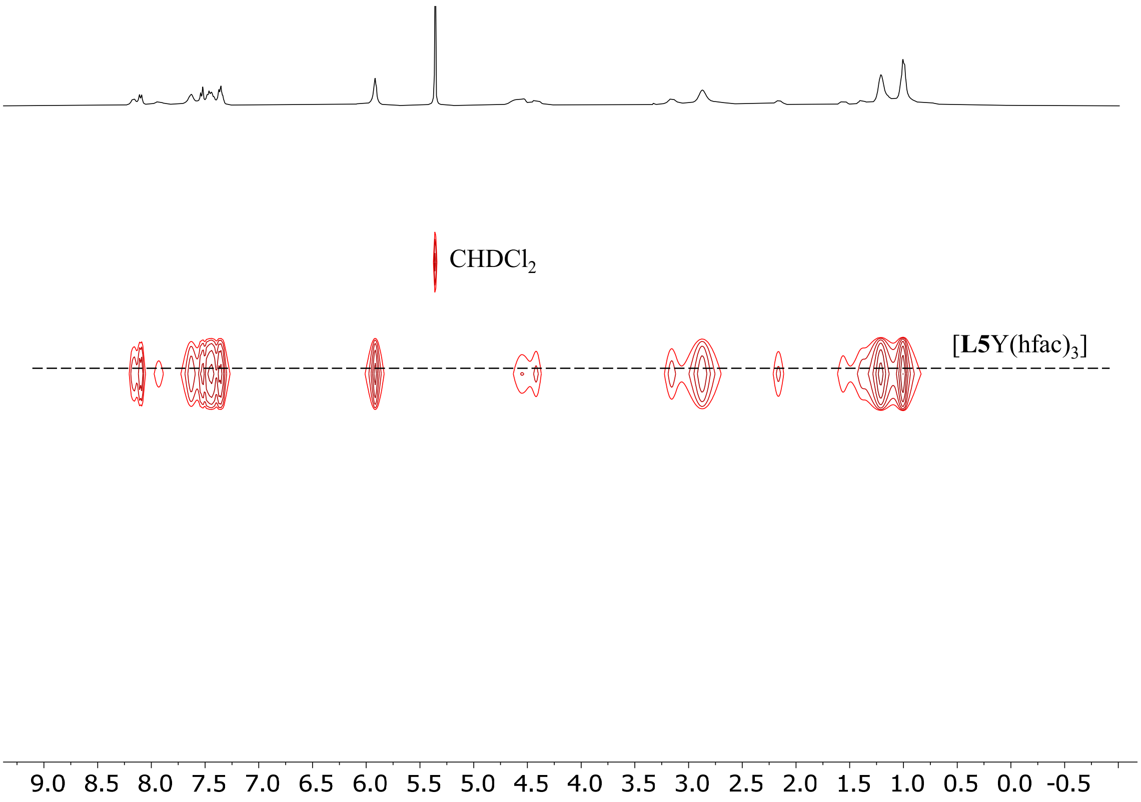

Figure A1-108. DOSY spectrum of [**L5**Y(hfac)$_3$] in CD$_2$Cl$_2$

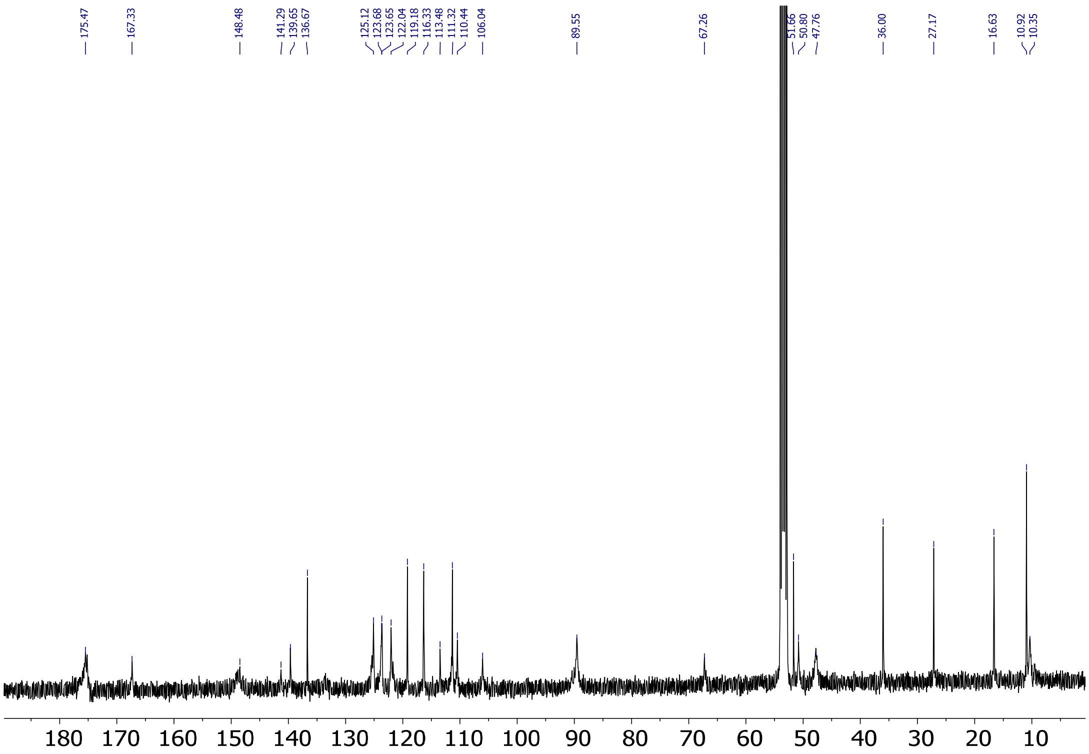

Figure A1-109. ^{13}C NMR spectrum of [**L5**Y(hfac)$_3$] in CD_2Cl_2.

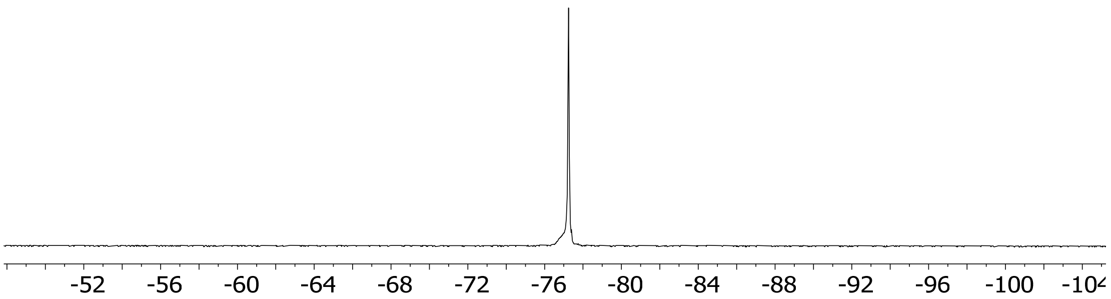

Figure A1-110. ^{19}F NMR spectrum of [**L5**Y(hfac)$_3$] in CD_2Cl_2

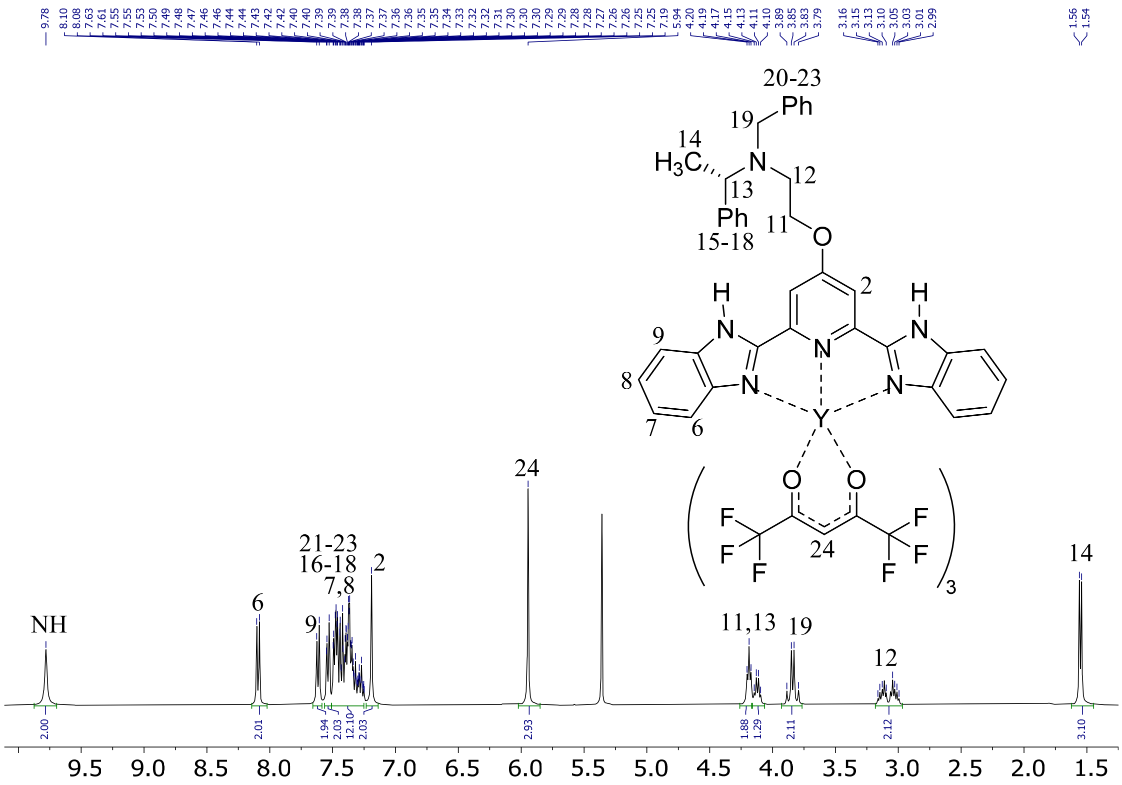

Figure A1-111. ^{1}H NMR spectrum of [**L7**Y(hfac)$_3$] in CD_2Cl_2.

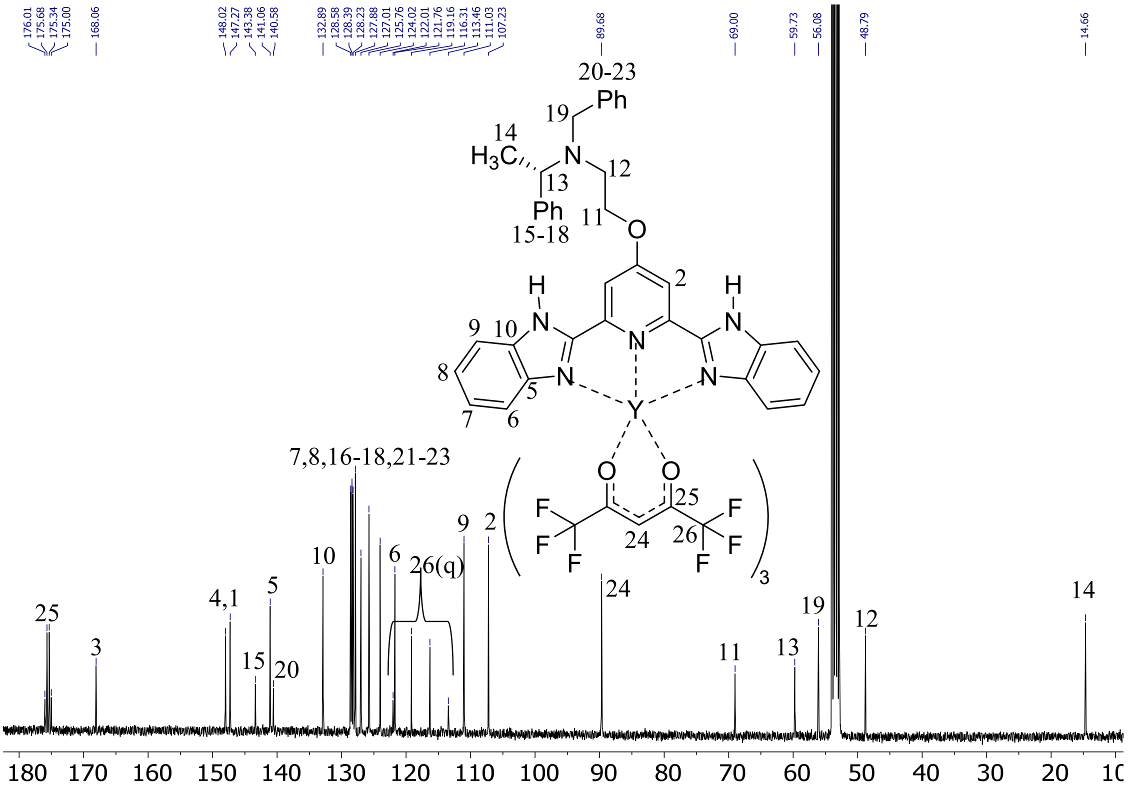

Figure A1-112. ^{13}C NMR spectrum of [**L7**Y(hfac)$_3$] in CD_2Cl_2.

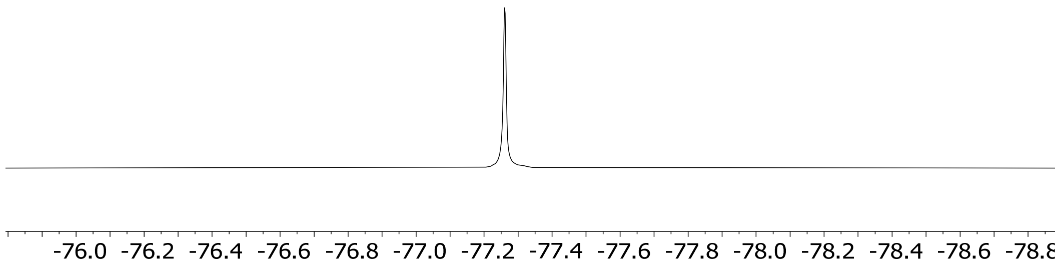

Figure A1-113. ^{19}F NMR spectrum of [**L7**Y(hfac)$_3$] in CD_2Cl_2.

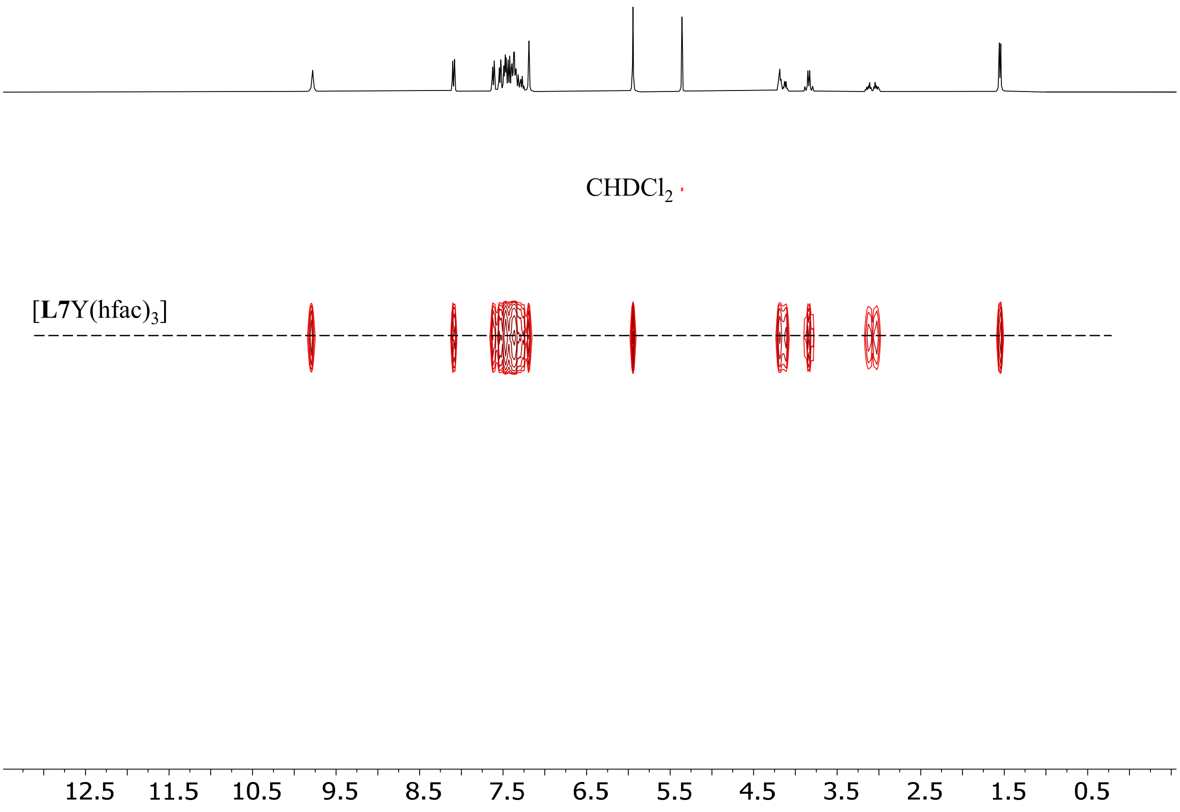

Figure A1-114. DOSY spectrum of [**L7**Y(hfac)$_3$] in CD_2Cl_2.

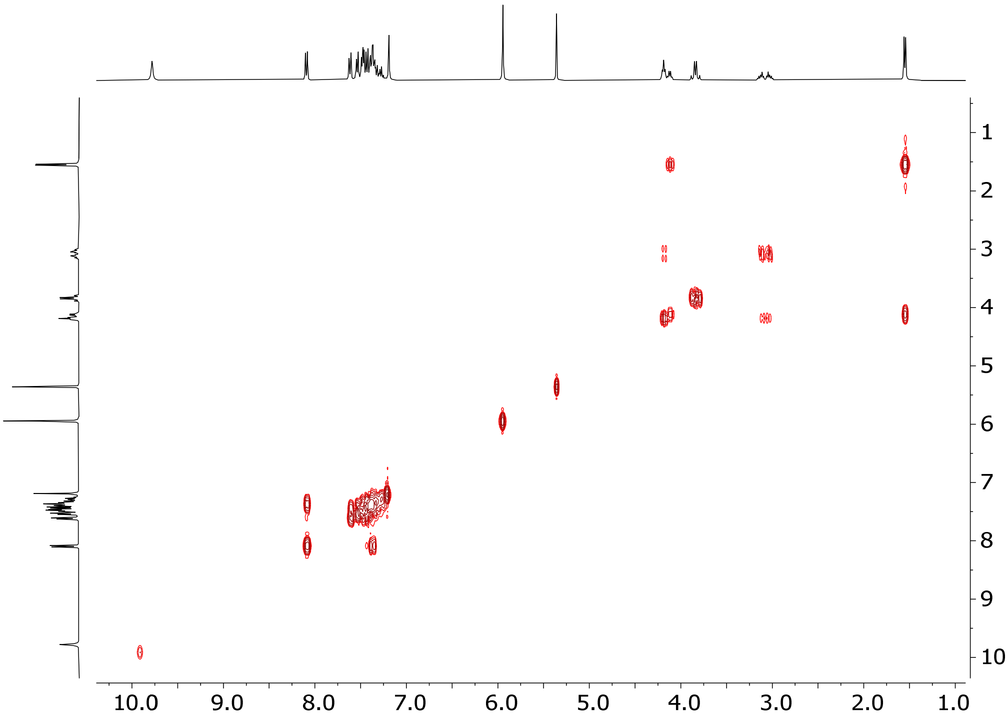

Figure A1-115. COSY spectrum of [**L7**Y(hfac)$_3$] in CD_2Cl_2.

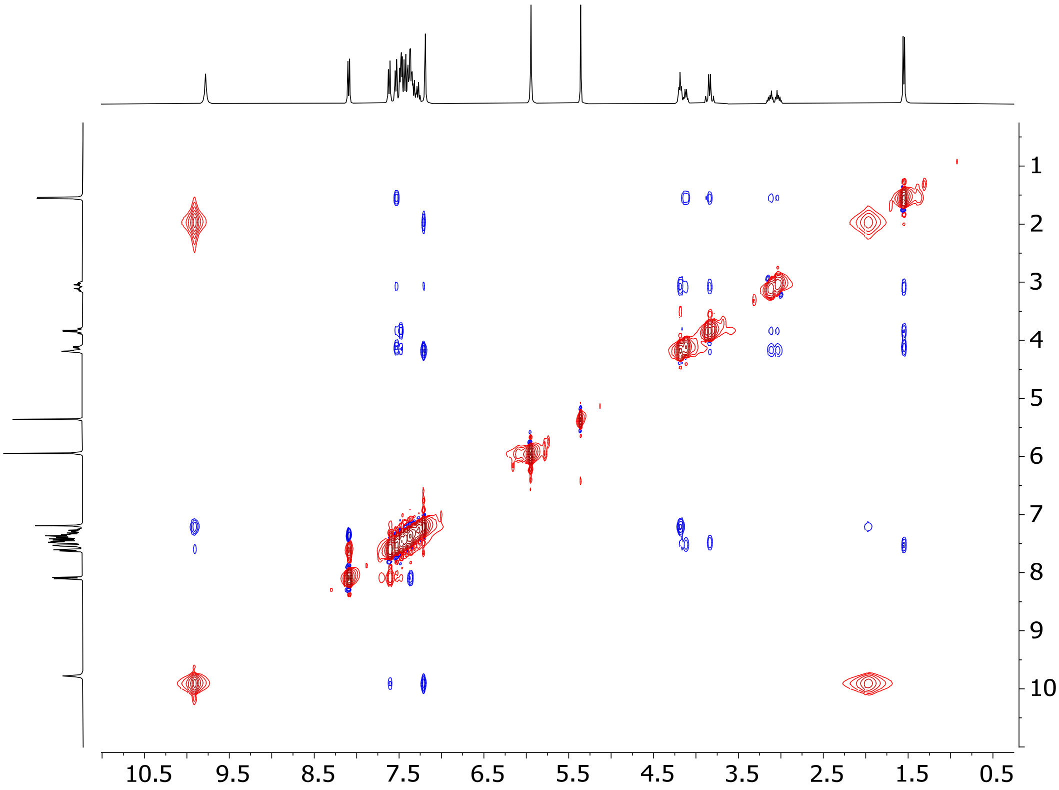

Figure A1-116. NOESY spectrum of [**L7**Y(hfac)$_3$] in CD_2Cl_2.

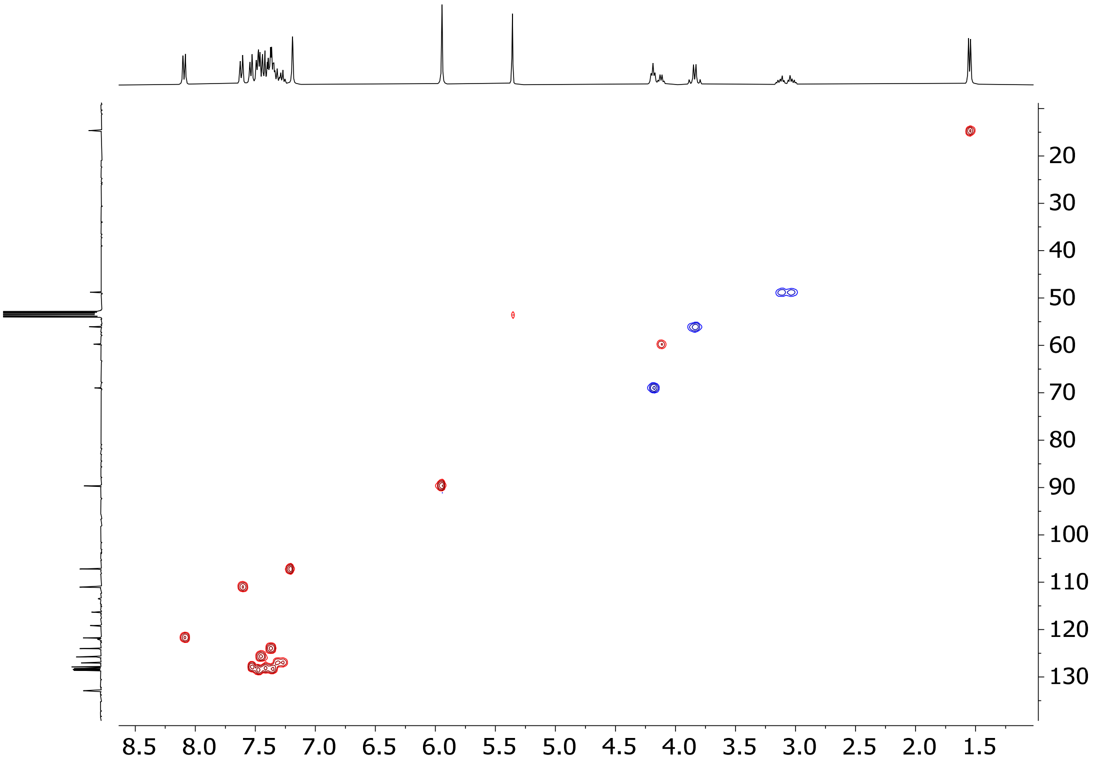

Figure A1-117. HSQC spectrum of [**L7**Y(hfac)$_3$] in CD_2Cl_2.

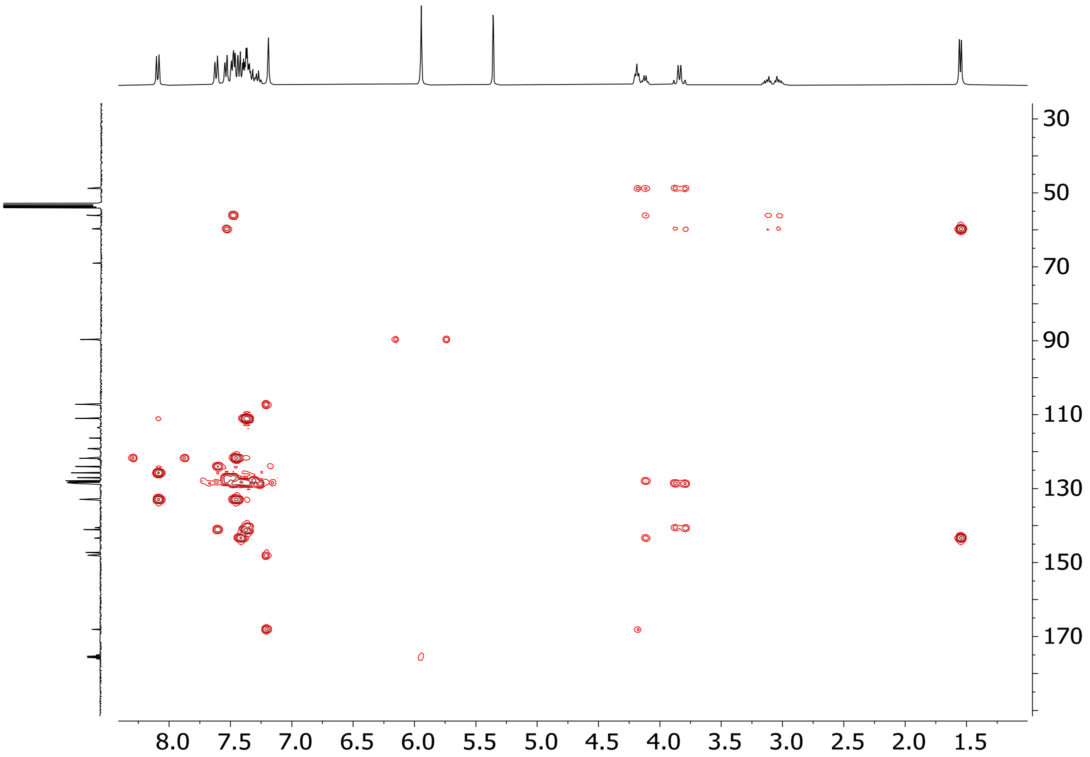

Figure A1-118. HMBC spectrum of [**L7**Y(hfac)$_3$] in CD_2Cl_2.

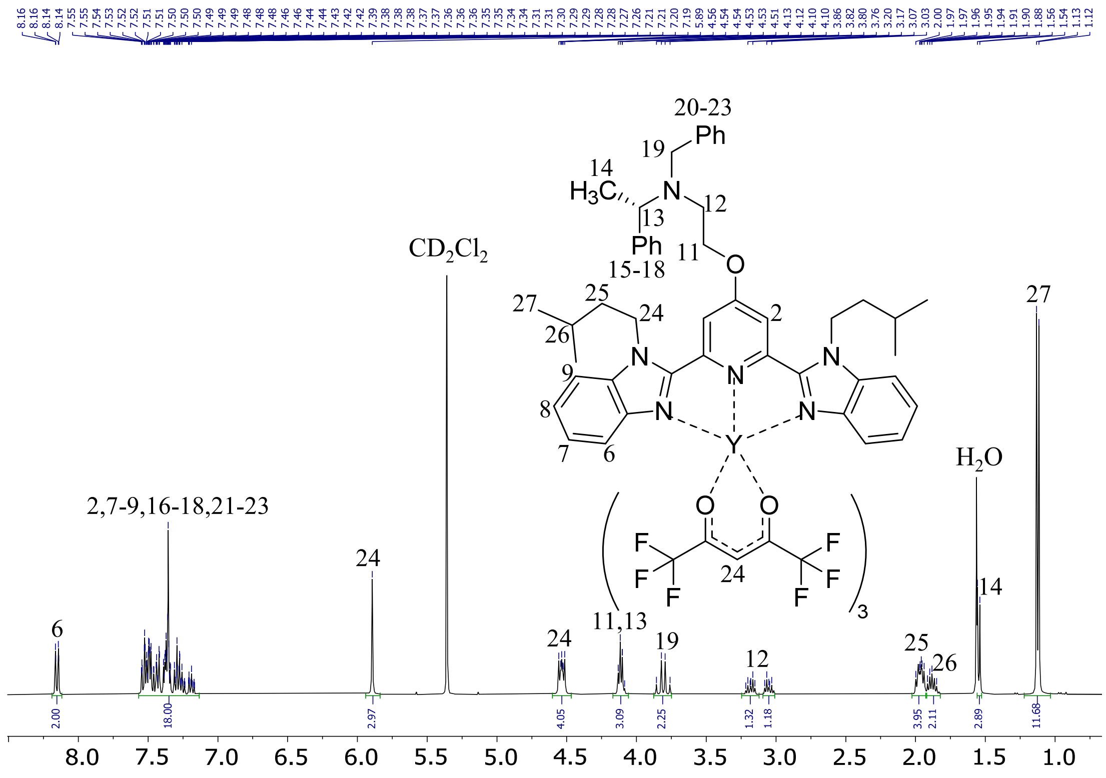

Figure A1-119. ^{1}H NMR spectrum of [**L8**Y(hfac)$_3$] in CD_2Cl_2.

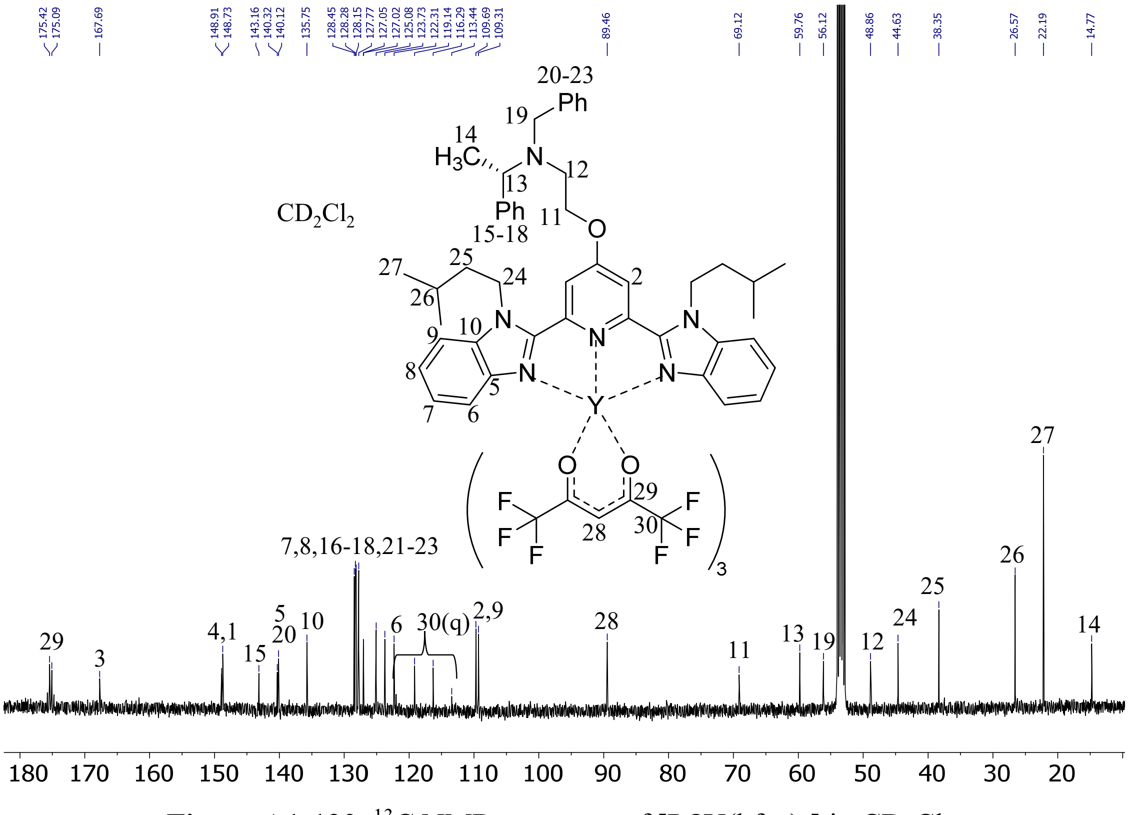

Figure A1-120. ^{13}C NMR spectrum of [**L8**Y(hfac)$_3$] in CD_2Cl_2.

HG489-Y-all.2.fid

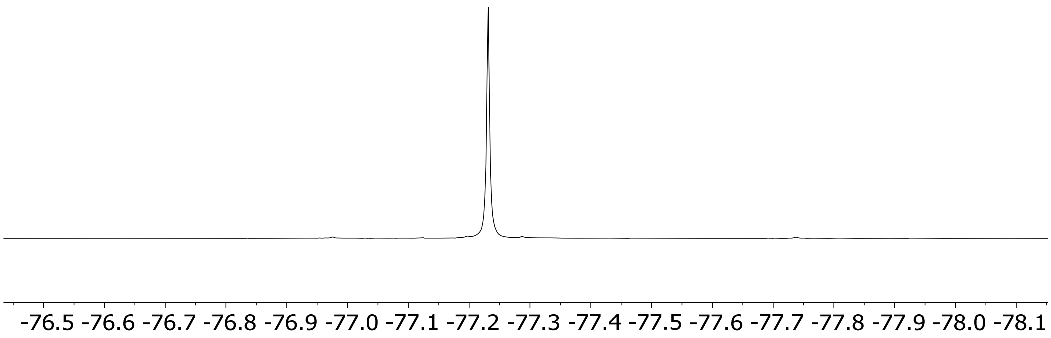

Figure A1-121. ^{19}F NMR spectrum of [**L8**Y(hfac)$_3$] in CD_2Cl_2.

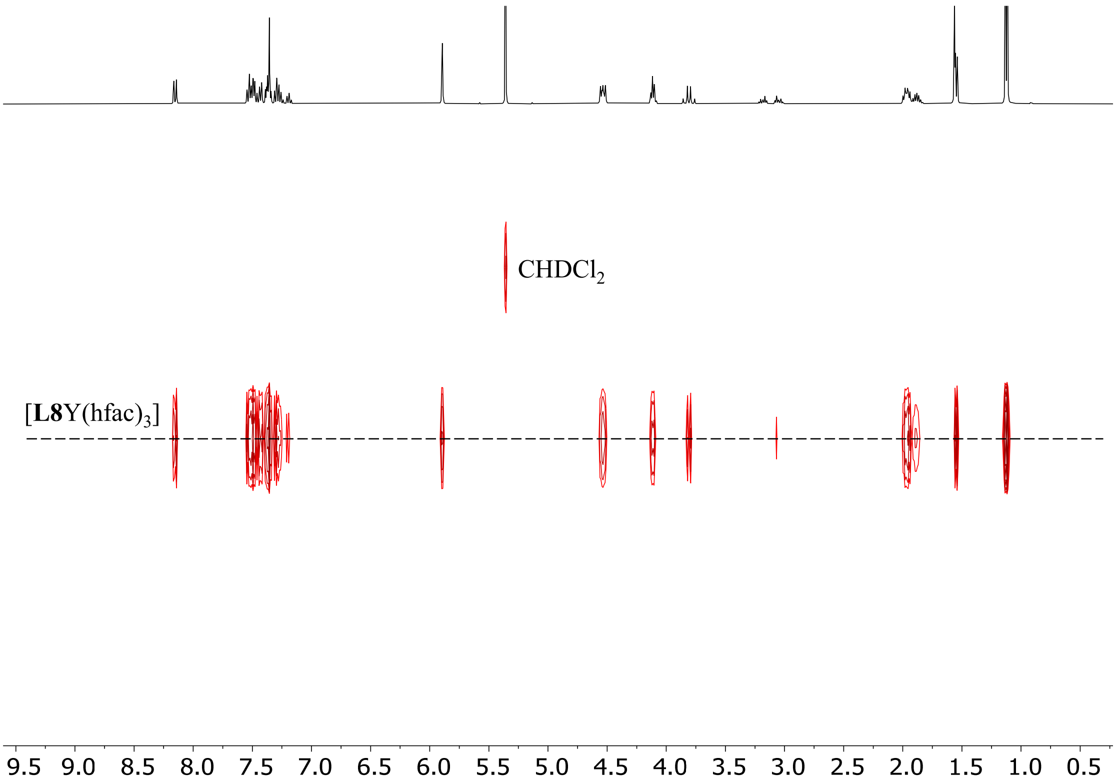

Figure A1-122. DOSY spectrum of [**L8**Y(hfac)$_3$] in CD_2Cl_2.

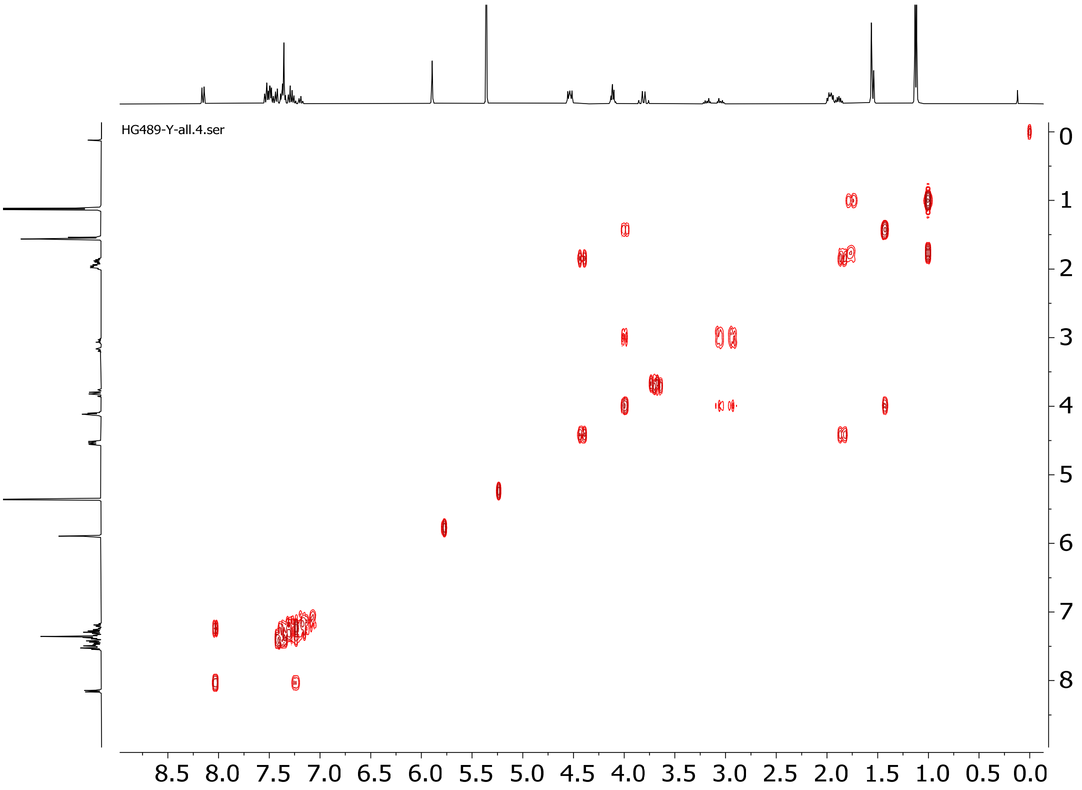

Figure A1-123. COSY spectrum of [**L8**Y(hfac)$_3$] in CD_2Cl_2.

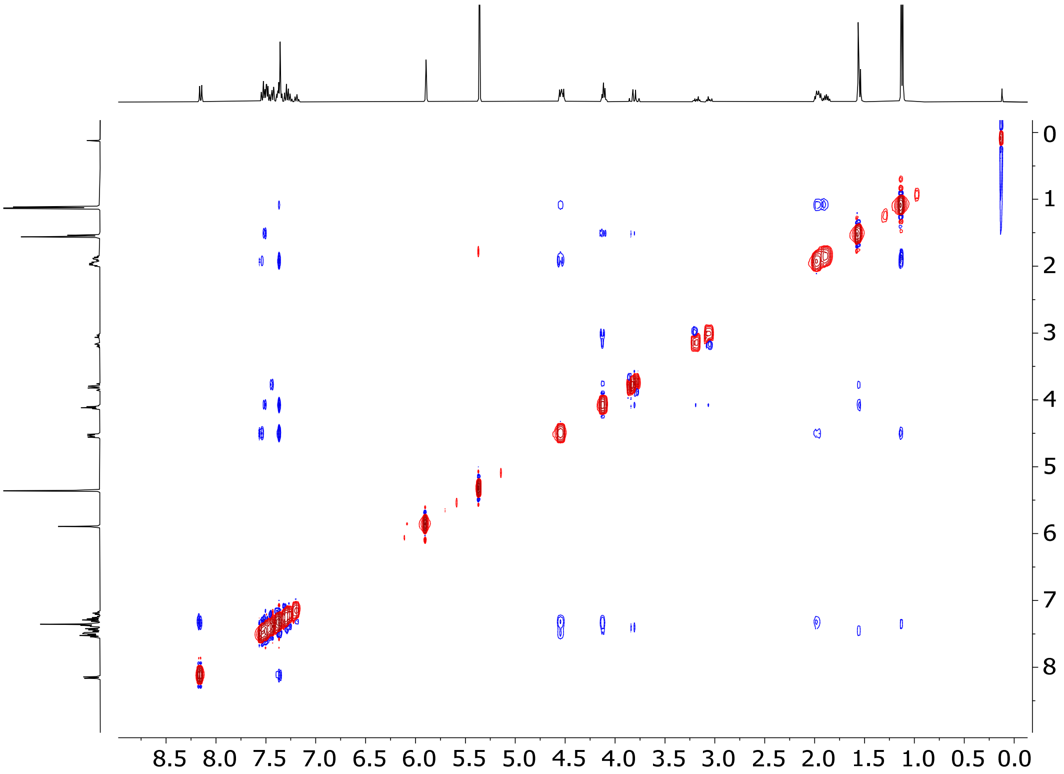

Figure A1-124. NOESY spectrum of [**L8**Y(hfac)$_3$] in CD_2Cl_2.

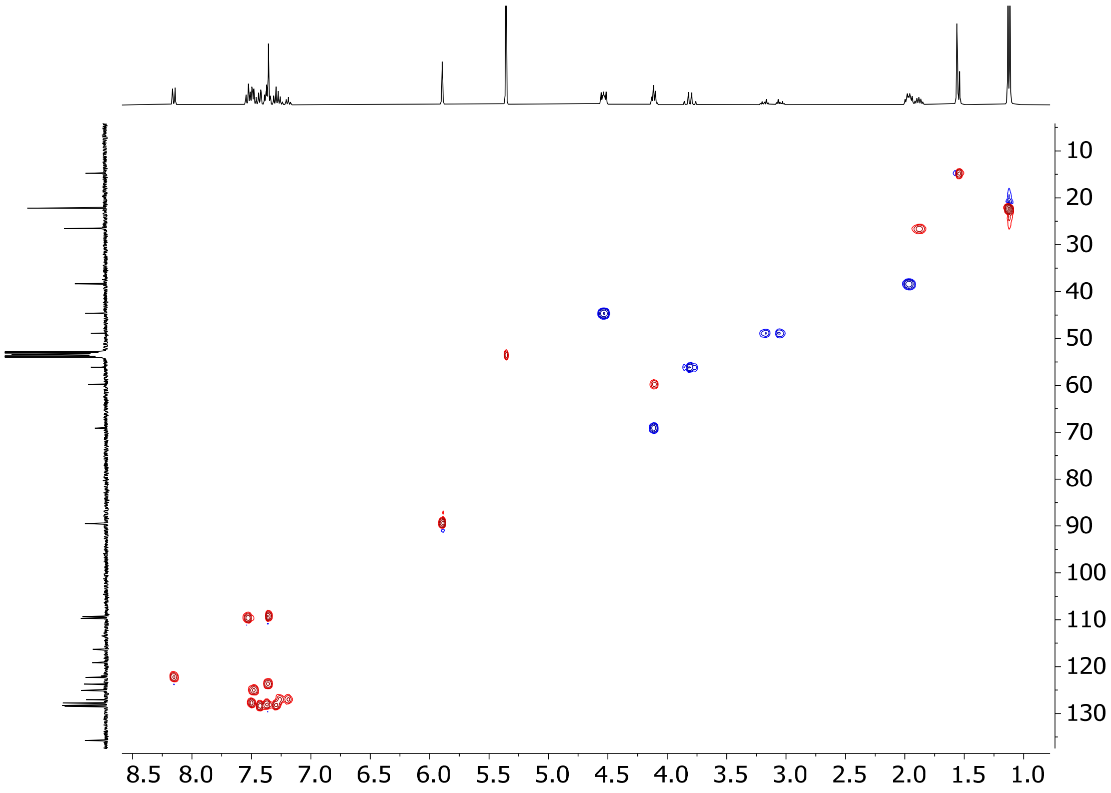

Figure A1-125. HSQC spectrum of [**L8**Y(hfac)$_3$] in CD_2Cl_2.

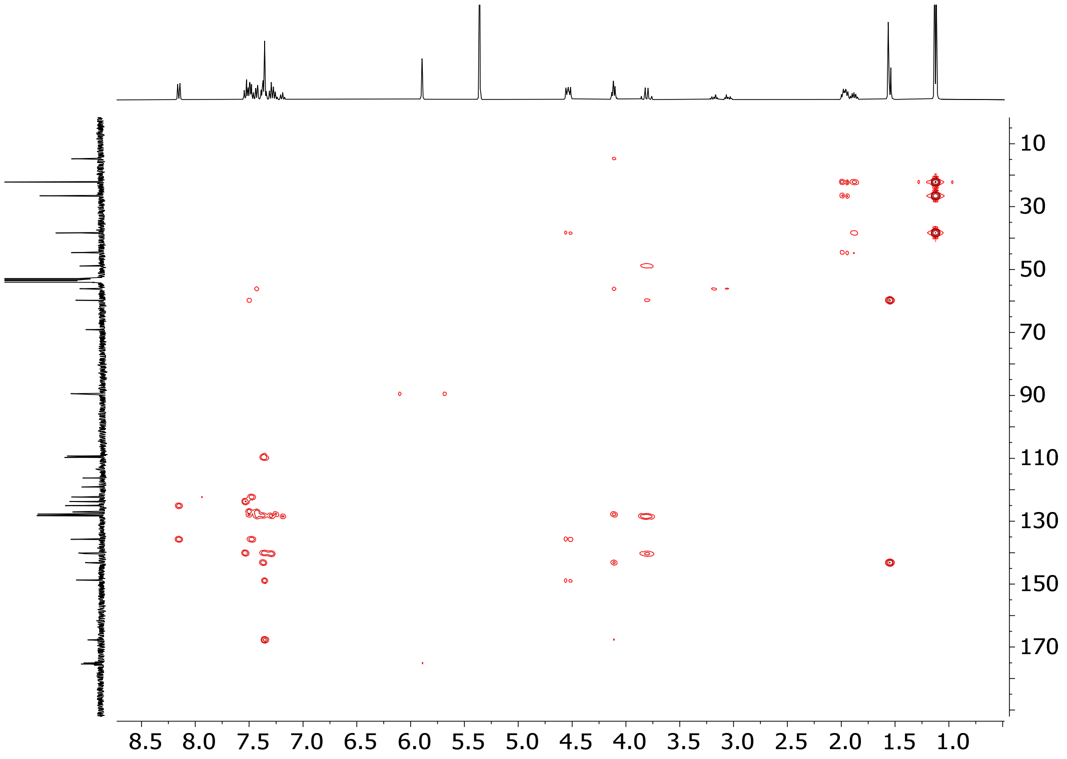

Figure A1-126. HMBC spectrum of [**L8**Y(hfac)$_3$] in CD_2Cl_2.

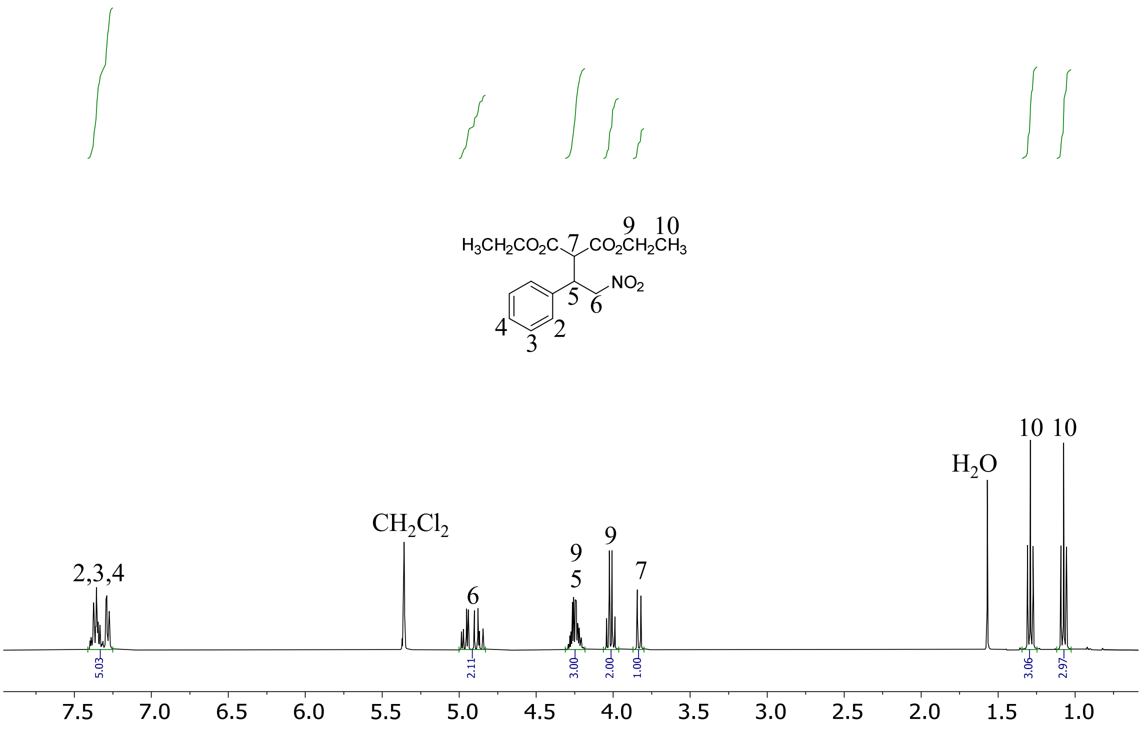

Figure A1-127. ^{1}H NMR spectrum of compound **3** in CD_2Cl_2.

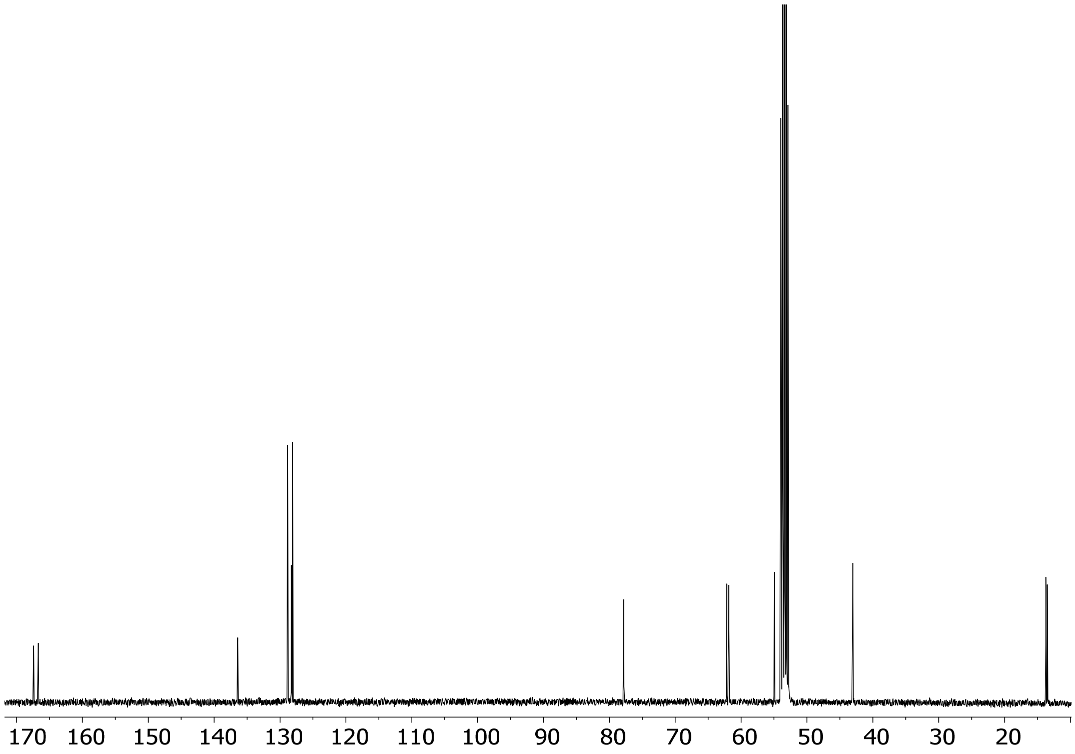

Figure A1-128. ^{13}C NMR spectrum of compound **3** in CD_2Cl_2.

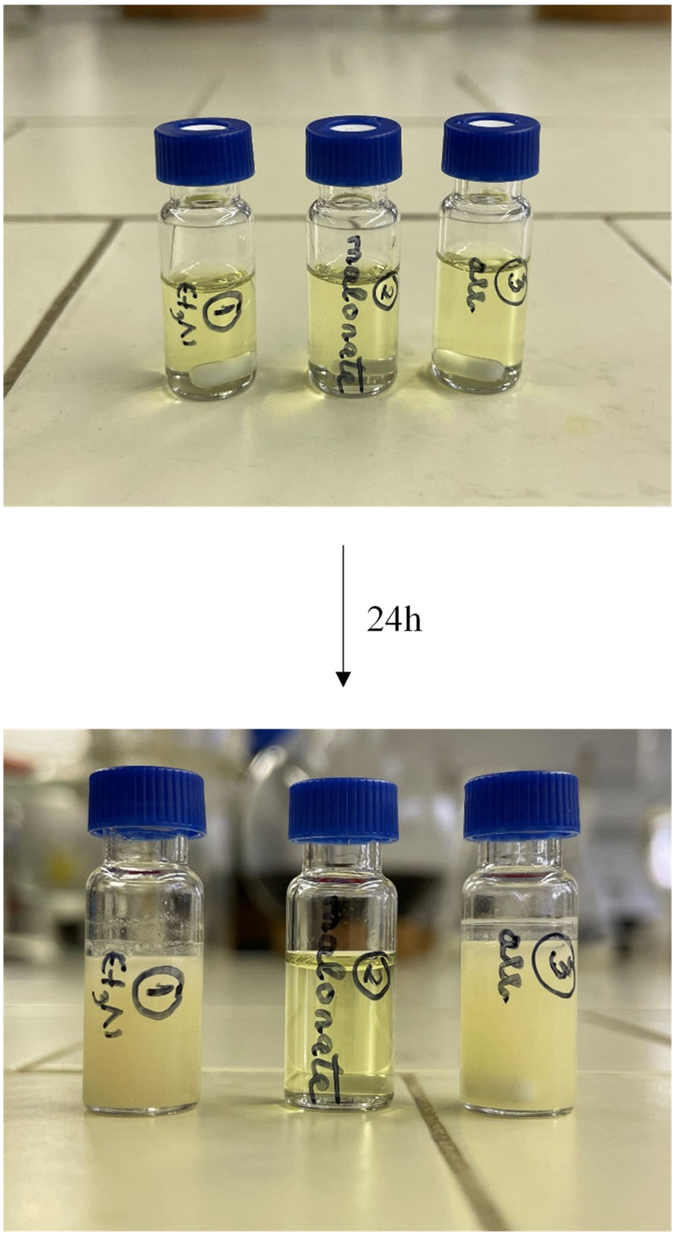

Figure A1-129. The appearance of insoluble solid observed in the reaction mixture of: compound **2** and Et_3N (left), compound **1** and Et_3N (middle), and entry 3 (Table 2) in the main text (right).

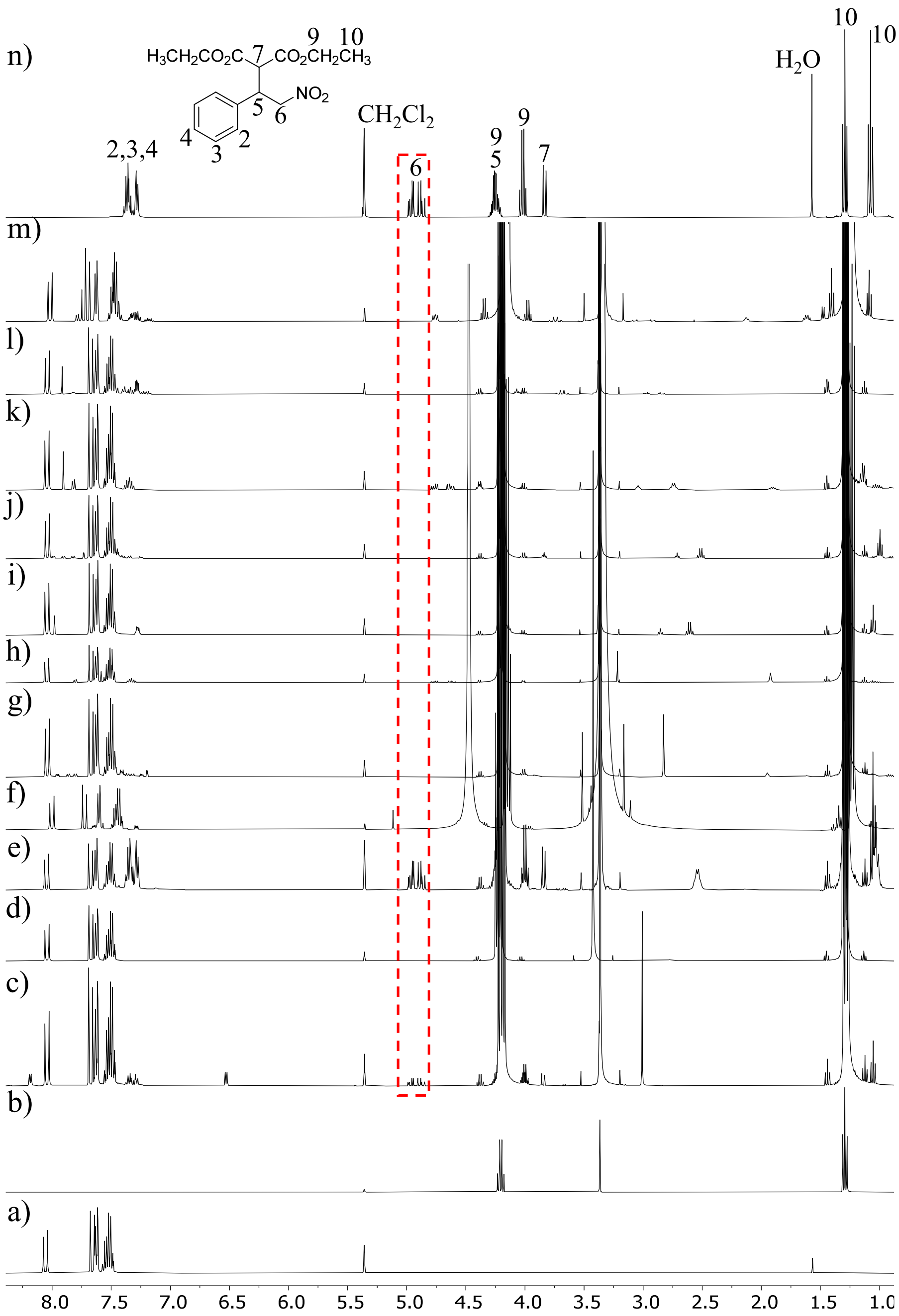

Figure A1-130. ¹H NMR spectra of a) nitrostyrene, b) diethyl malonate, reaction mixture from c) entry 1, d) entry 2, e) entry 3, f) entry 4, g) entry 5, h) entry 6, i) entry 7, j) entry 8, k) entry 9, l) entry 10, m) entry 11, and n) desired product **3**.

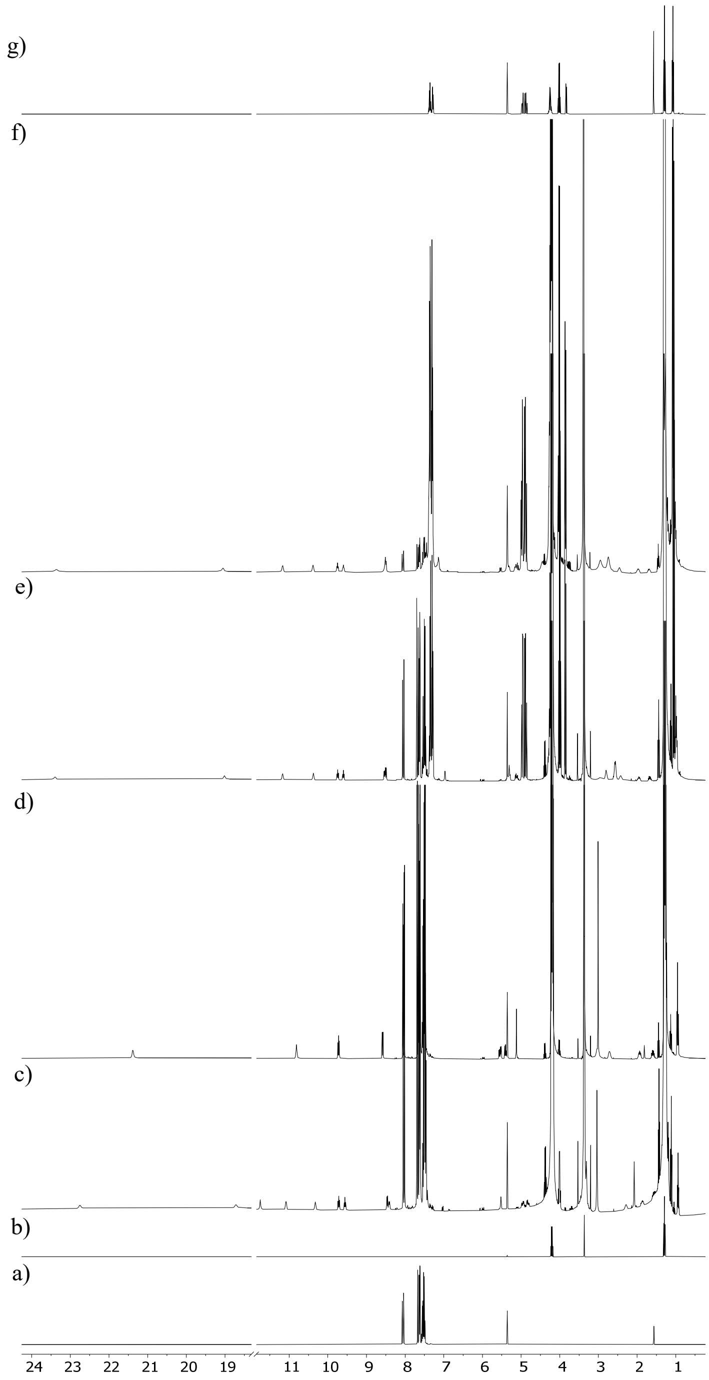

Figure A1-131. ^{1}H NMR spectra of a) nitrostyrene, b) diethyl malonate, reaction mixture from c) entry 12, d) entry 13, e) entry 14, f) entry 15, and g) desired product **3**.

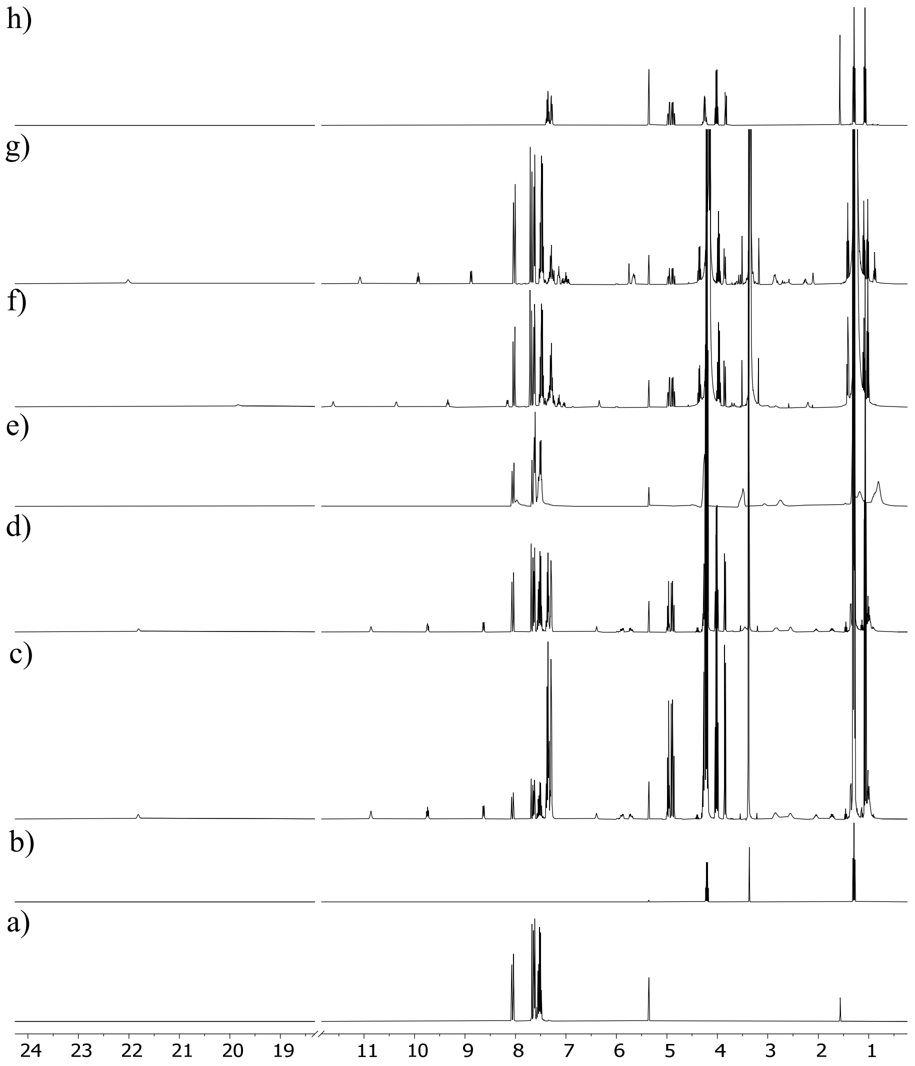

Figure A1-132. ^{1}H NMR spectra of a) nitrostyrene, b) diethyl malonate, reaction mixture from c) entry 17, d) entry 18, e) entry 19, f) entry 20, g) entry 21, and h) desired product **3**.

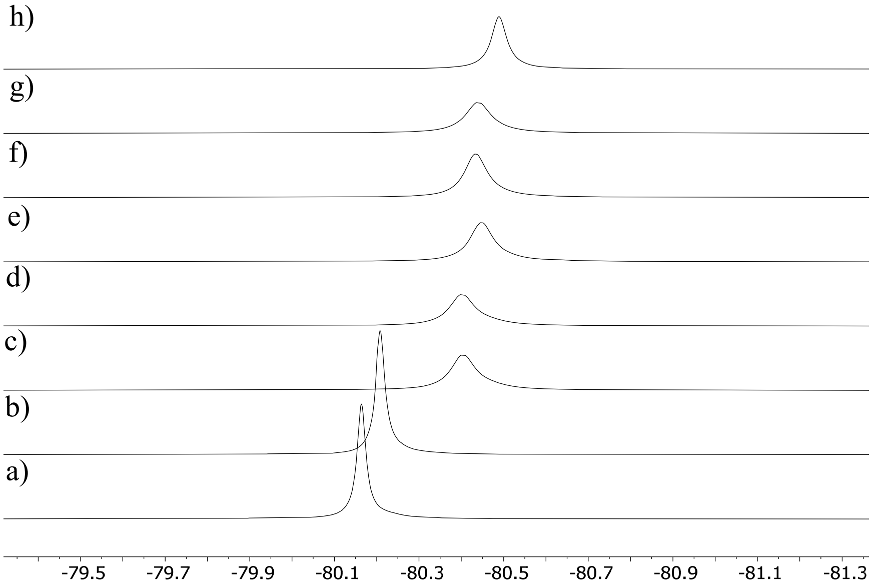

Figure A1-133. ^{19}F NMR spectra of reaction mixture from entry a) entry12, b) entry 13, c) entry 14, d) entry 15, e) entry 16, f) entry 17, g) entry 20, and h) entry 21.

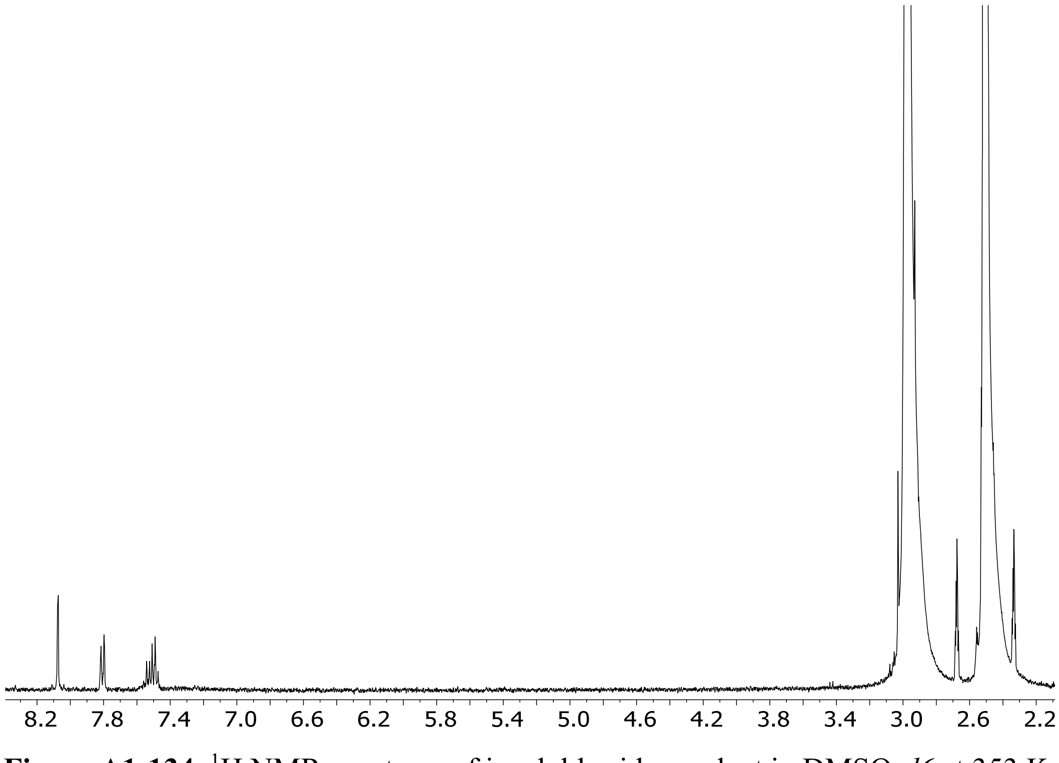

Figure A1-134. ^{1}H NMR spectrum of insoluble side-product in DMSO-*d6* at 353 K.

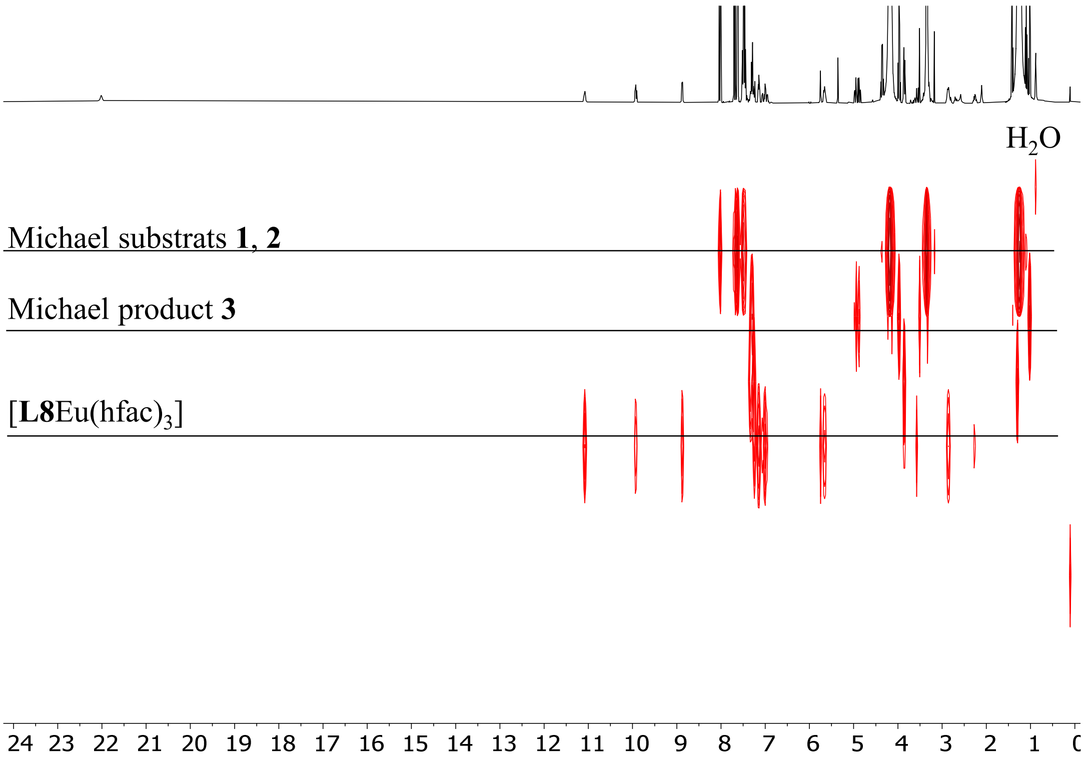

Figure A1-135. DOSY spectrum of reaction mixture from entry 21 in CD_2Cl_2.

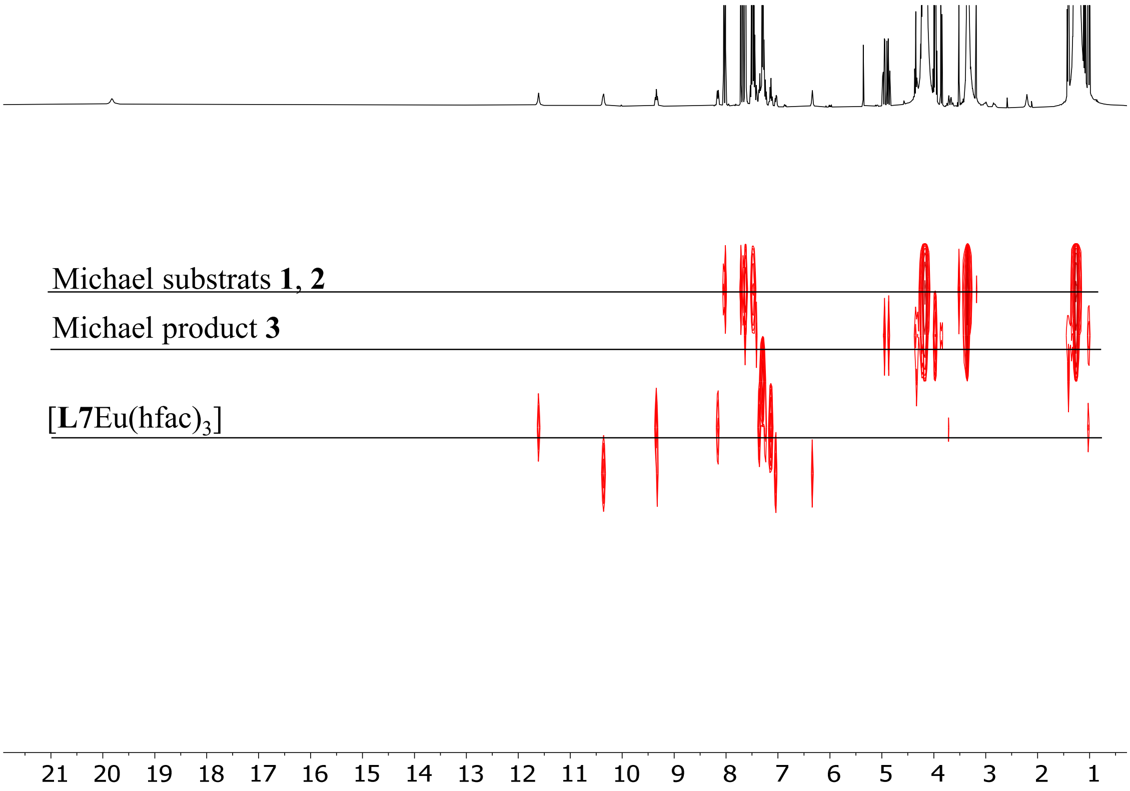

Figure A1-136. DOSY spectrum of reaction mixture from entry 20 in CD_2Cl_2.

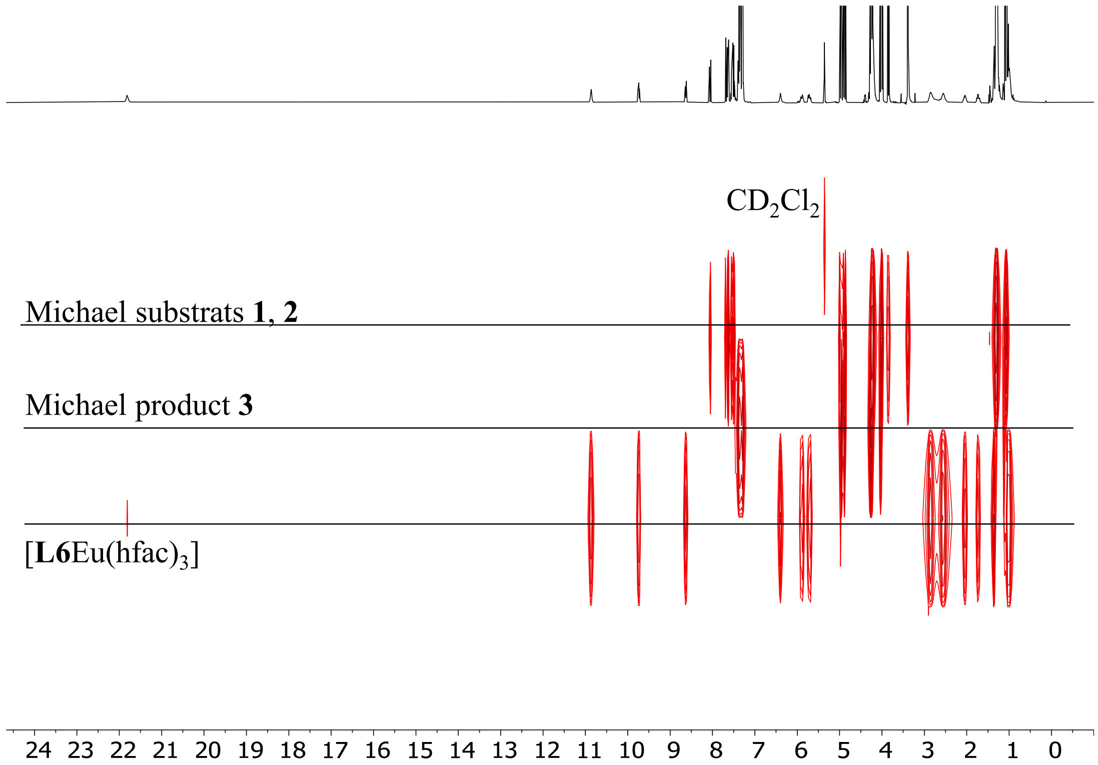

Figure A1-137. DOSY spectrum of reaction mixture from entry 17 in CD_2Cl_2.

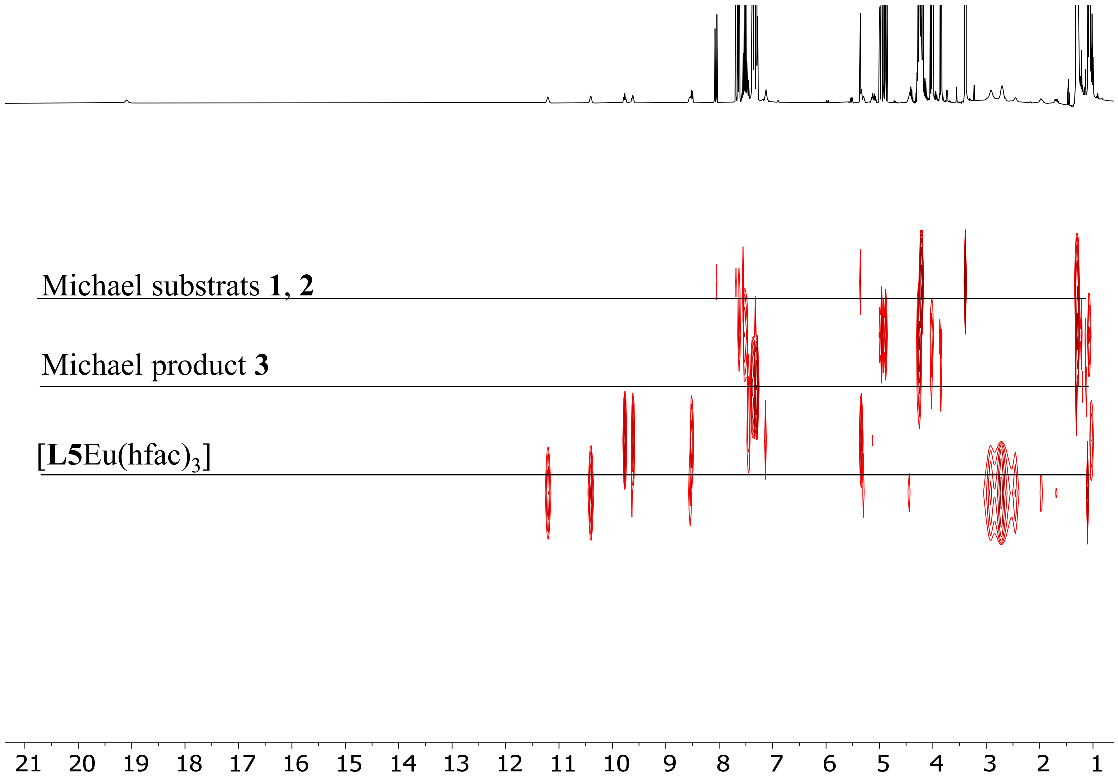

Figure A1-138. DOSY spectrum of reaction mixture from entry 15 in CD_2Cl_2.

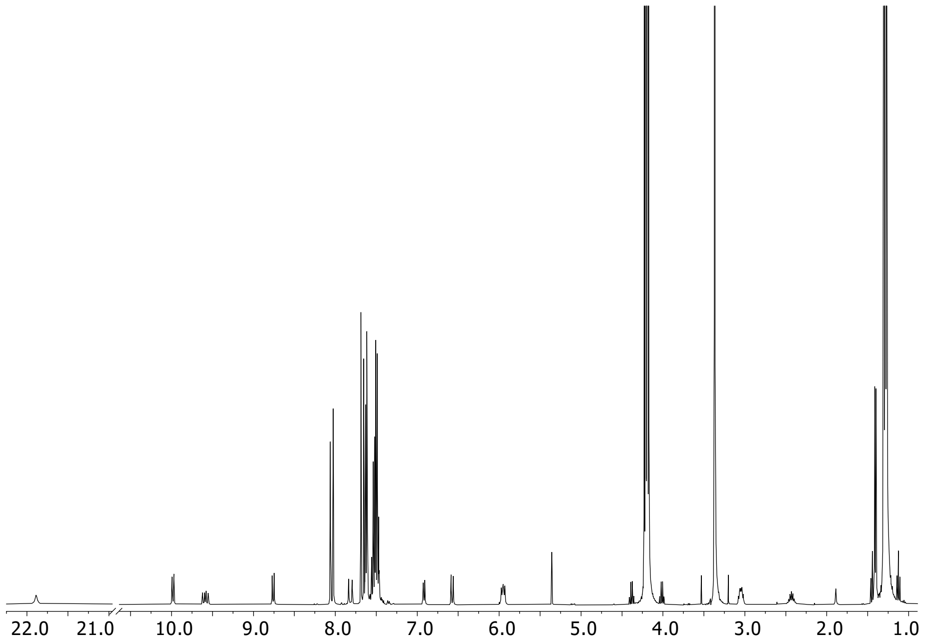

Figure A1-139. ^{1}H NMR spectrum of reaction mixture from entry 22.

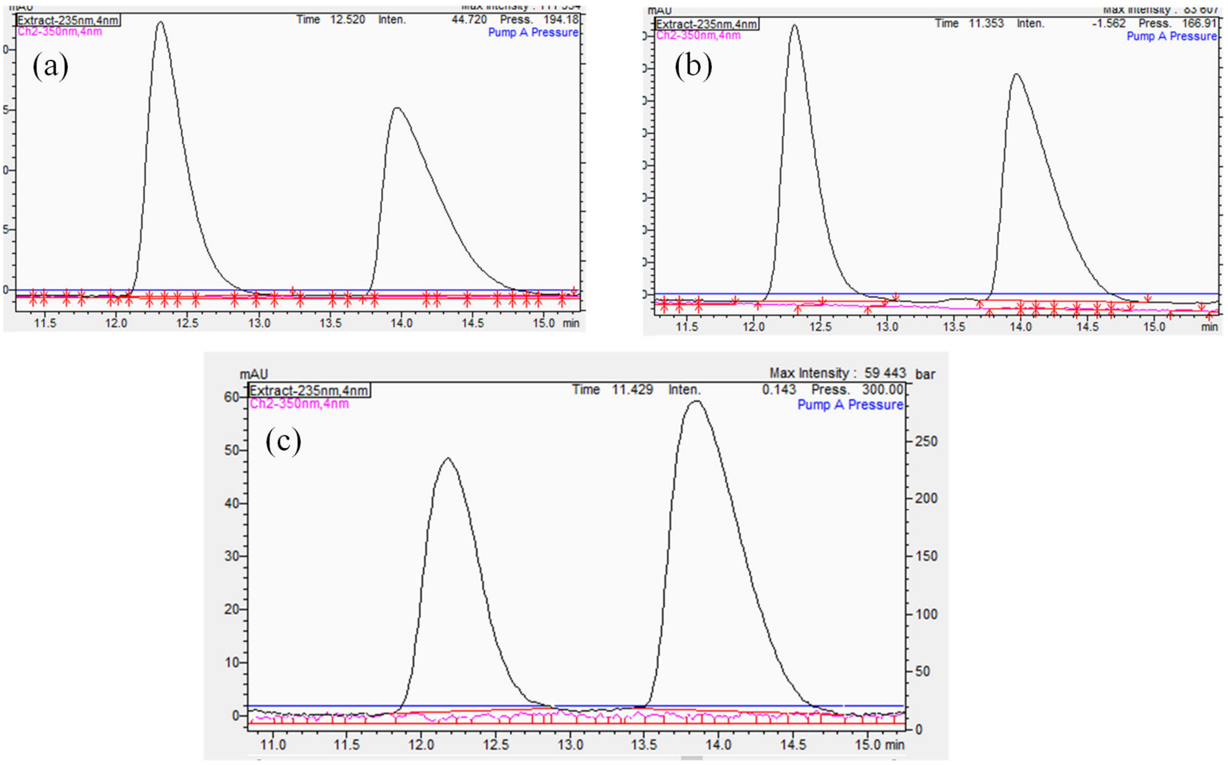

Figure A1-140. HPLC data of compound **3** from a) entry 17, b) entry 15, and c) entry 21.

Appendix 2: Crystal structures of ligands and lanthanide complexes

Table A2-1. Summary of crystal data, intensity measurements and structure refinements for **L1**·CH_2Cl_2.

CCDC number	2472888	
Empirical formula	$C_{22}H_{20}Cl_2N_6$	
Chemical formula moiety	$C_{21}H_{18}N_6 \cdot CH_2Cl_2$	
Formula weight	439.34	
Temperature	119.99(10) K	
Wavelength	1.54184 Å	
Crystal system	Monoclinic	
Space group	P 2_1/c	
Unit cell dimensions	a = 10.56341(18) Å	α = 90°.
	b = 21.4719(3) Å	β = 102.8037(14)°.
	c = 9.62625(11) Å	γ = 90°.
Volume	2129.10(6) $Å^3$	
Z	4	
Density (calculated)	1.371 Mg/m^3	
Absorption coefficient	2.914 mm^{-1}	
F(000)	912	
Crystal size	0.32 x 0.03 x 0.01 mm^3	
Theta range for data collection	4.118 to 75.662°.	
Index ranges	-13<=h<=12, -26<=k<=26, -11<=l<=6	
Reflections collected	32979	
Independent reflections	4307 [R(int) = 0.0513]	
Completeness to theta = 67.684°	100.0 %	
Absorption correction	Analytical	
Max. and min. transmission	0.958 and 0.640	
Refinement method	Full-matrix least-squares on F^2	
Data / restraints / parameters	4307 / 0 / 279	
Goodness-of-fit on F^2	1.081	
Final R indices [I>2sigma(I)]	R1 = 0.0541, wR2 = 0.1341	
R indices (all data)	R1 = 0.0613, wR2 = 0.1382	
Extinction coefficient	n/a	
Largest diff. peak and hole	0.398 and -0.681 e.$Å^{-3}$	

Table A2-2. Selected least-squares planes data of **L1**·CH_2Cl_2.

	Abbreviation	RMSD (Å)	Max deviation (Å) (Atom)
Benzimidazole (1)<br>N2 C7 N1 C6 C1 C2 C3 C4 C5	Bz1	0.008	C1 (0.014)
Benzimidazole (2)<br>N5 C15 N4 C16 C21 C20 C19 C18 C17	Bz2	0.003	C18, N14 (0.005)
Pyridine<br>N3 C8 C9 C10 C13 C14	Py	0.014	C10, C13 (0.020)

Table A2-3. Interplanar angles (°) for **L1**·CH_2Cl_2.

Plane	Benzimidazole (1)	Benzimidazole (2)
Pyridine	8.09(6)	3.12(6)
Benzimidazole (1)		8.41(5)

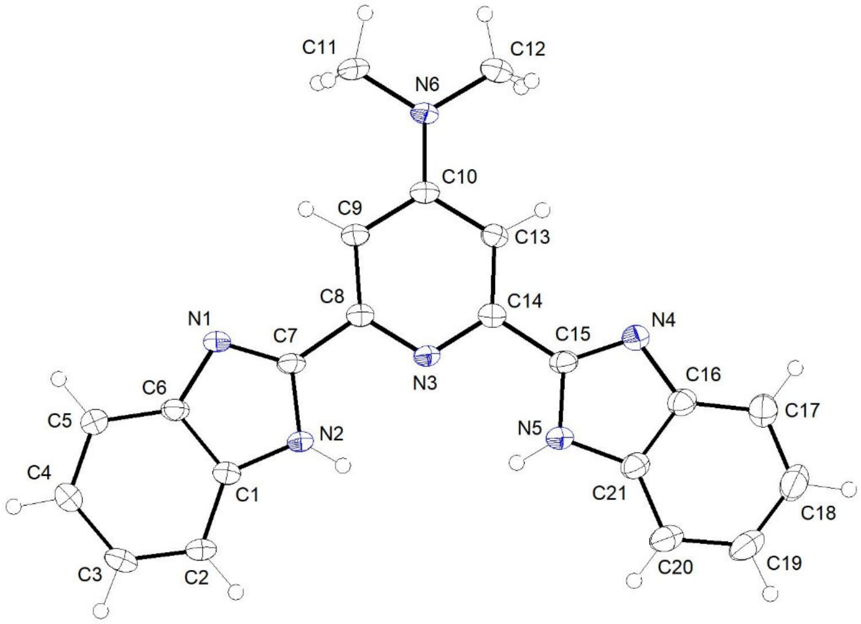

Figure A2-1. ORTEP view of **L1** (thermal ellipsoids are drawn at 50% probability level) with numbering scheme. Solvent CH_2Cl_2 molecule is omitted for clarity.

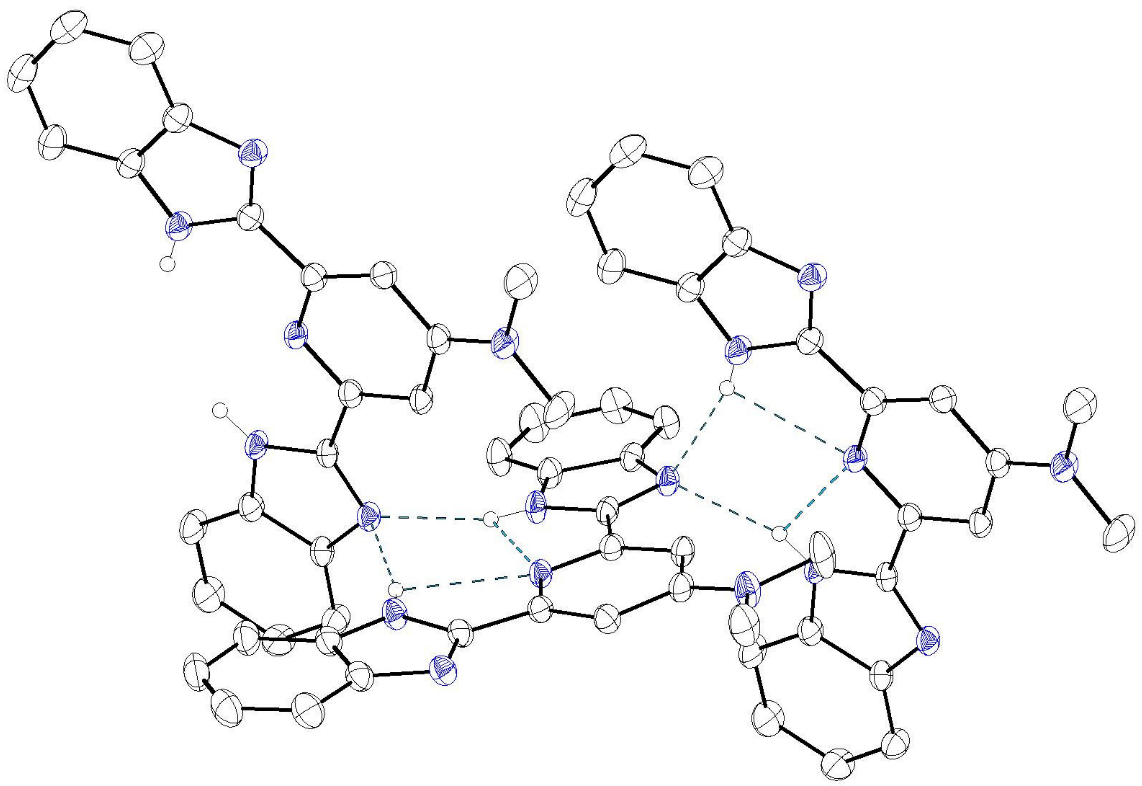

Figure A2-2. Hydrogen bonds in **L1**.

Table A2-4. Hydrogen bonds for **L1** [Å and °].

D-H...A	d(D-H)	d(H...A)	d(D...A)	<(DHA)
C(22)-H(22B)...N(4)	0.99	2.29	3.249(3)	161.6
N(2)-H(2)...N(1)#1	0.85(3)	2.10(3)	2.912(2)	161(2)
N(5)-H(5)...N(1)#1	0.87(3)	2.19(3)	3.038(3)	165(2)
C(9)-H(9)...N(3)#2	0.95	2.63	3.572(3)	172.7

Symmetry transformations used to generate equivalent atoms:
#1 x,-y+3/2,z+1/2 #2 x,-y+3/2,z-1/2

Table A2-5. Summary of crystal data, intensity measurements and structure refinements for **L2**·$0.5CH_2Cl_2$.

CCDC	2472889	
Empirical formula	$C_{26.5}H_{29}ClN_6$	
Chemical formula moiety	$C_{26}H_{28}N_6 \cdot 0.5CH_2Cl_2$	
Formula weight	467.01	
Temperature	119.99(10) K	
Wavelength	1.54184 Å	
Crystal system	Monoclinic	
Space group	$P\ 2_1$	
Unit cell dimensions	a = 9.59092(17) Å	α = 90°.
	b = 36.6694(6) Å	β = 105.614(2)°.
	c = 14.4428(4) Å	γ = 90°.
Volume	4891.99(18) $Å^3$	
Z	8	
Density (calculated)	1.268 Mg/m^3	
Absorption coefficient	1.583 mm^{-1}	
F(000)	1976	
Crystal size	0.36 x 0.03 x 0.02 mm^3	
Theta range for data collection	3.177 to 75.966°.	
Index ranges	-11<=h<=7, -45<=k<=44, -17<=l<=17	
Reflections collected	43792	
Independent reflections	17794 [R(int) = 0.1202]	
Completeness to theta = 67.684°	99.1 %	
Absorption correction	Analytical	
Max. and min. transmission	0.973 and 0.764	
Refinement method	Full-matrix least-squares on F^2	
Data / restraints / parameters	17794 / 1 / 1224	
Goodness-of-fit on F^2	1.124	
Final R indices [I>2sigma(I)]	R1 = 0.0903, wR2 = 0.2320	
R indices (all data)	R1 = 0.1495, wR2 = 0.2856	
Absolute structure parameter	-0.07(4)	
Extinction coefficient	n/a	
Largest diff. peak and hole	0.569 and -0.590 e.$Å^{-3}$	

Table A2-6. Selected least-squares planes data of **L2**·$0.5CH_2Cl_2$.

	Abbreviation	RMSD (Å)	Max deviation (Å) (Atom)
Benzimidazole (1) N2C C7C N1C C1C C6C C5C C4C C3C C2C	Bz1	0.009	N2C (0.018)
Benzimidazole (2) N5C C20C N6C C26C C21C C22C C23C C24C C25C	Bz2	0.011	C21C, C23C (0.019)
Pyridine N3C C17C C16C C15C C14C C13C	Py	0.008	C15C (0.014)

Table A2-7. Interplanar angles (°) for **L2**·$0.5CH_2Cl_2$.

Plane	Benzimidazole (1)	Benzimidazole (2)
Pyridine	42.5(3)	6.7(3)
Benzimidazole (1)		49.0(5)

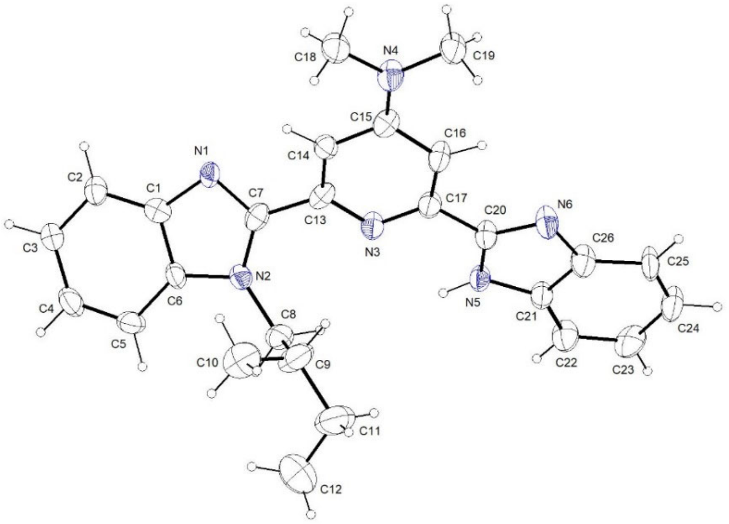

Figure A2-3. ORTEP view of **L2** (thermal ellipsoids are drawn at 50% probability level) with numbering scheme. Only one molecule among 4 (labelled B, C and D) in the asymmetric unit is shown here. All asymmetric carbon (C9, C9B, C9C and C9D) atoms have *S* configuration.

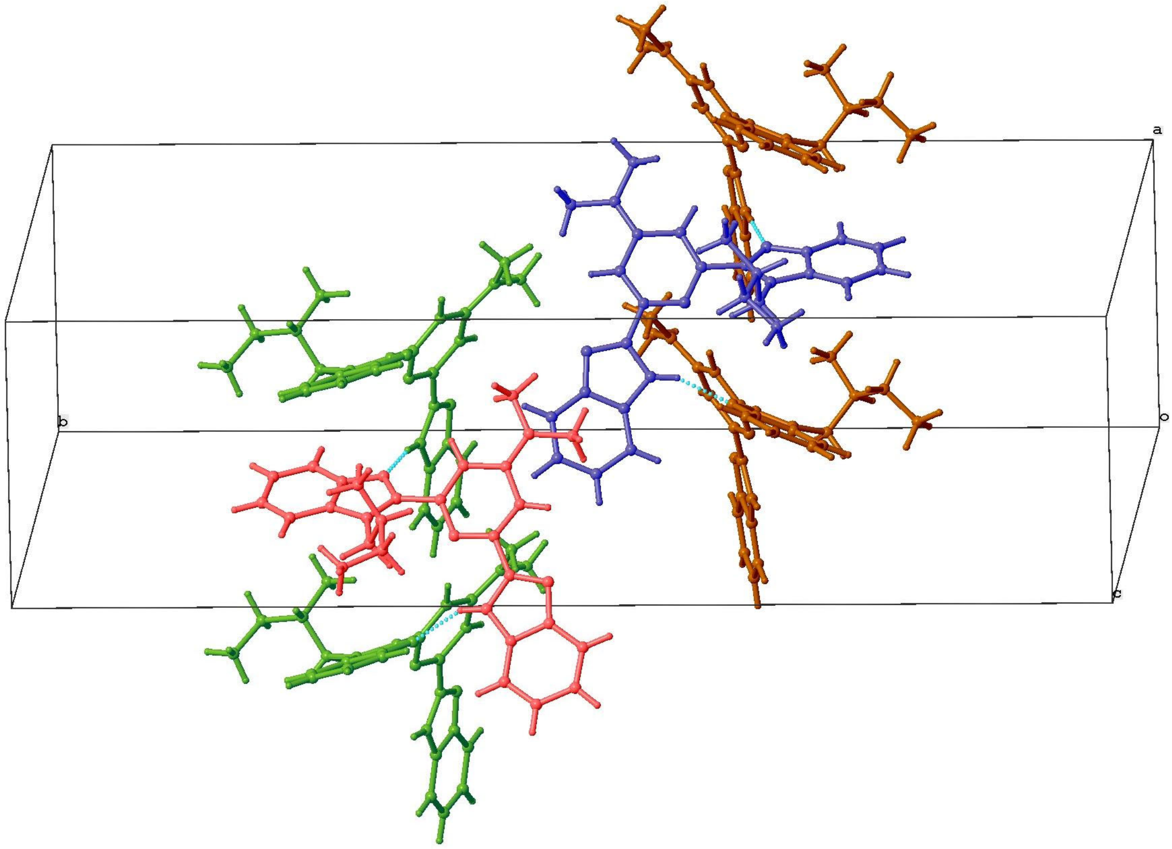

Figure A2-4. Hydrogen bonds in **L2** (cyan) show two polymeric networks along a direction: molA (red) connected with molB (green) and molC (blue) connected with molD (brown).

Table A2-8. Hydrogen bonds for **L2** [Å and °].

D-H...A	d(D-H)	d(H...A)	d(D...A)	<(DHA)
N(5C)-H(5C)...N(1D)#1	0.88	2.08	2.893(13)	153.6
N(5B)-H(5B)...N(1)	0.88	2.02	2.887(13)	168.6
N(5)-H(5)...N(1B)#1	0.88	2.12	2.904(12)	147.5
N(5D)-H(5D)...N(1C)	0.88	1.98	2.851(13)	168.9

Symmetry transformations used to generate equivalent atoms:
#1 x-1,y,z

Table A2-9. Summary of crystal data, intensity measurements and structure refinements for **L3**.

CCDC	2472890	
Empirical formula	$C_{31}H_{38}N_6$	
Chemical formula moiety	$C_{31}H_{38}N_6$	
Formula weight	494.67	
Temperature	120.00(10) K	
Wavelength	1.54184 Å	
Crystal system	Orthorhombic	
Space group	P 2_1 2_1 2_1	
Unit cell dimensions	a = 11.76783(10) Å	α = 90°.
	b = 14.68225(10) Å	β = 90°.
	c = 15.97162(14) Å	γ = 90°.
Volume	2759.55(4) $Å^3$	
Z	4	
Density (calculated)	1.191 Mg/m^3	
Absorption coefficient	0.558 mm^{-1}	
F(000)	1064	
Crystal size	0.282 x 0.204 x 0.159 mm^3	
Theta range for data collection	4.090 to 75.917°.	
Index ranges	-14<=h<=14, -18<=k<=16, -19<=l<=19	
Reflections collected	28155	
Independent reflections	5666 [R(int) = 0.0275]	
Completeness to theta = 67.684°	100.0 %	
Absorption correction	Analytical	
Max. and min. transmission	0.942 and 0.918	
Refinement method	Full-matrix least-squares on F^2	
Data / restraints / parameters	5666 / 0 / 341	
Goodness-of-fit on F^2	1.039	
Final R indices [I>2sigma(I)]	R_1 = 0.0317, wR_2 = 0.0812	
R indices (all data)	R_1 = 0.0334, wR_2 = 0.0826	
Absolute structure parameter	-0.1(3)	
Extinction coefficient	n/a	
Largest diff. peak and hole	0.195 and -0.189 e.$Å^{-3}$	

Table A2-10. Selected least-squares planes data of **L3**.

	Abbreviation	RMSD (Å)	Max deviation (Å) (Atom)
Benzimidazole (1) N1 C7 N2 C1 C2 C3 C4 C5 C6	Bz1	0.021	N2 (0.031)
Benzimidazole (2) N4 C13 N5 C19 C14 C15 C16 C17 C18	Bz2	0.028	C13 (0.043)
Pyridine N3 C12 C11 C10 C9 C8	Py	0.009	C9 (0.013)

Table A2-11. Interplanar angles (°) for **L3**.

Plane	Benzimidazole (1)	Benzimidazole (2)
Pyridine	44.38(5)	13.92(5)
Benzimidazole (1)		45.51(4)

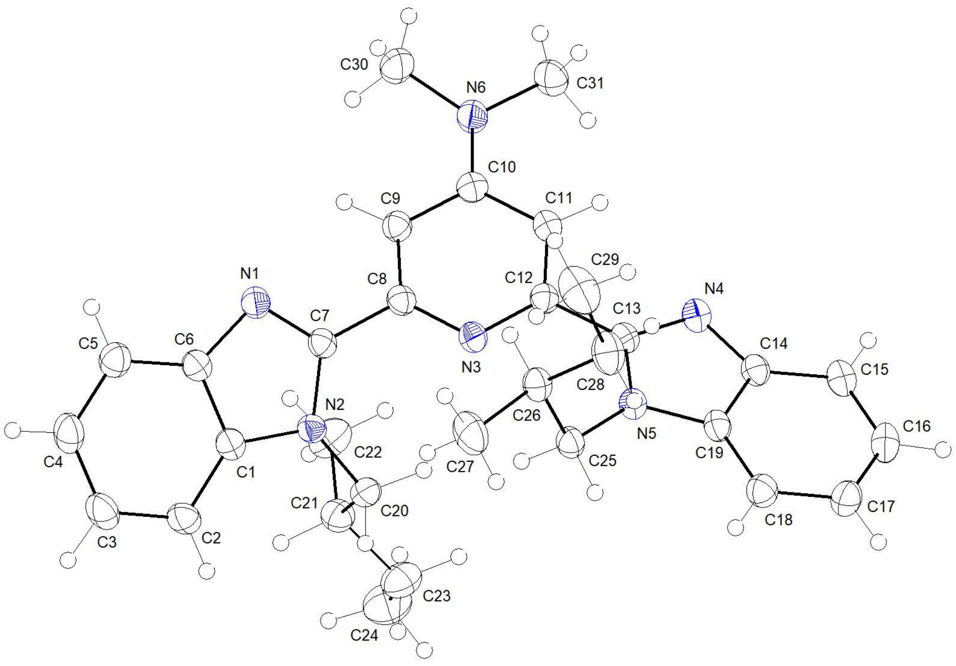

Figure A2-5. ORTEP view of **L3** (thermal ellipsoids are drawn at 50% probability level) with numbering scheme. Asymmetric C21 and C26 atoms have *S* configuration.

Table A2-12. Summary of crystal data, intensity measurements and structure refinements for **L4**·$CH_3OH·2H_2O$.

CCDC	2472891	
Empirical formula	$C_{26}H_{34}N_6O_4$	
Chemical formula moiety	$C_{25}H_{26}N_6O·CH_3OH·2H_2O$	
Formula weight	494.59	
Temperature	99.98(11) K	
Wavelength	1.54184 Å	
Crystal system	Monoclinic	
Space group	P 2_1/c	
Unit cell dimensions	a = 11.90101(10) Å	α = 90°.
	b = 13.07144(10) Å	β = 109.2762(9)°.
	c = 17.20864(13) Å	γ = 90°.
Volume	2526.95(4) Å^3	
Z	4	
Density (calculated)	1.300 Mg/m^3	
Absorption coefficient	0.730 mm^{-1}	
F(000)	1056	
Crystal size	0.26 x 0.23 x 0.13 mm^3	
Theta range for data collection	3.935 to 74.272°.	
Index ranges	-14<=h<=14, -16<=k<=15, -18<=l<=21	
Reflections collected	21712	
Independent reflections	5071 [R(int) = 0.0191]	
Completeness to theta = 67.684°	99.9 %	
Absorption correction	Analytical	
Max. and min. transmission	0.919 and 0.865	
Refinement method	Full-matrix least-squares on F^2	
Data / restraints / parameters	5071 / 0 / 349	
Goodness-of-fit on F^2	1.041	
Final R indices [I>2sigma(I)]	R1 = 0.0365, wR2 = 0.0930	
R indices (all data)	R1 = 0.0409, wR2 = 0.0958	
Extinction coefficient	n/a	
Largest diff. peak and hole	0.255 and -0.230 e.Å^{-3}	

Table A2-13. Selected least-squares planes data of **L4**·CH_3OH·$2H_2O$.

	Abbreviation	RMSD (Å)	Max deviation (Å) (Atom)
Benzimidazole (1)<br>N1 C7 N2 C6 C1 C2 C3 C4 C5	Bz1	0.015	N1 (0.020)
Benzimidazole (2)<br>N5 C13 N4 C19 C14 C15 C16 C17 C18	Bz2	0.019	C17 (0.027)
Pyridine<br>N3 C8 C9 C10 C11 C12	Py	0.005	C8 (0.007)

Table A2-14. Interplanar angles (°) for **L4**·CH_3OH·$2H_2O$.

Plane	Benzimidazole (1)	Benzimidazole (2)
Pyridine	8.20(3)	1.45(3)
Benzimidazole (1)		9.27(3)

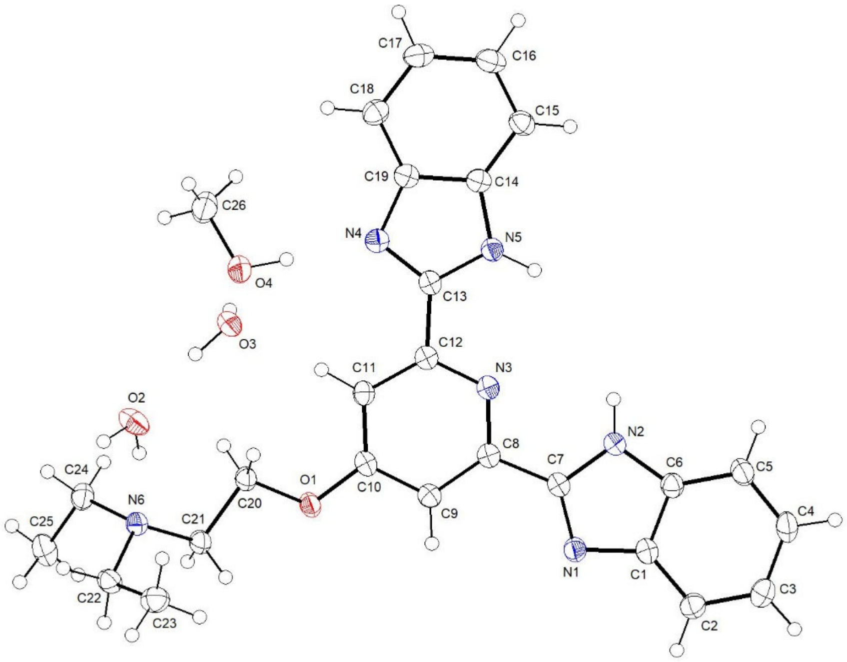

Figure A2-6. ORTEP view of **L4**·CH_3OH·$2H_2O$ (thermal ellipsoids are drawn at 50% probability level) with numbering scheme.

Table A2-15. Hydrogen bonds for **L4**·CH_3OH·$2H_2O$ [Å and °].

D-H...A	d(D-H)	d(H...A)	d(D...A)	<(DHA)
N(2)-H(2)...O(3)#1	0.897(15)	1.951(16)	2.8365(13)	169.1(13)
N(5)-H(5)...O(3)#1	0.918(15)	1.950(15)	2.8488(13)	166.0(13)
O(3)-H(3A)...O(2)	0.888(18)	1.779(18)	2.6602(12)	171.3(16)
O(3)-H(3B)...O(4)	0.890(18)	1.870(18)	2.7508(14)	170.2(16)
O(2)-H(2B)...N(6)	0.90(2)	1.92(2)	2.8149(13)	171.5(16)
O(4)-H(4A)...N(4)	0.929(19)	1.804(19)	2.7322(13)	179.7(18)

Symmetry transformations used to generate equivalent atoms:

#1 -x+1,-y+1,-z+1

Table A2-16. Summary of crystal data, intensity measurements and structure refinements for **L5**

CCDC	2472892	
Empirical formula	$C_{30}H_{36}N_6O$	
Chemical formula moiety	$C_{30}H_{36}N_6O$	
Formula weight	496.65	
Temperature	100.00(10) K	
Wavelength	1.54184 Å	
Crystal system	Monoclinic	
Space group	P 2_1	
Unit cell dimensions	a = 14.15207(8) Å	α = 90°.
	b = 20.97488(13) Å	β = 97.2245(5)°.
	c = 9.21700(5) Å	γ = 90°.
Volume	2714.23(3) $Å^3$	
Z	4	
Density (calculated)	1.215 Mg/m^3	
Absorption coefficient	0.599 mm^{-1}	
F(000)	1064	
Crystal size	0.17 x 0.12 x 0.09 mm^3	
Theta range for data collection	3.148 to 76.079°.	
Index ranges	-17<=h<=16, -25<=k<=24, -11<=l<=11	
Reflections collected	74036	
Independent reflections	10977 [R(int) = 0.0203]	
Completeness to theta = 67.684°	100.0 %	
Absorption correction	Analytical	
Max. and min. transmission	0.952 and 0.917	
Refinement method	Full-matrix least-squares on F^2	
Data / restraints / parameters	10977 / 7 / 675	
Goodness-of-fit on F^2	1.041	
Final R indices [I>2sigma(I)]	R1 = 0.0504, wR2 = 0.1435	
R indices (all data)	R1 = 0.0523, wR2 = 0.1454	
Absolute structure parameter	-0.1(4)	
Extinction coefficient	n/a	
Largest diff. peak and hole	0.350 and -0.317 e.$Å^{-3}$	

Table A2-17. Selected least-squares planes data of **L5**.

	Abbreviation	RMSD (Å)	Max deviation (Å) (Atom)
Benzimidazole (1)<br>N1 C7 N2 C6 C1 C2 C3 C4 C5	Bz1	0.014	C3 (0.025)
Benzimidazole (2)<br>N4 C19 C13 N5 C14 C15 C16 C17 C18	Bz2	0.010	C18 (0.015)
Pyridine<br>N3 C8 C9 C10 C11 C12	Py	0.010	C9 (0.015)

Table A2-18. Interplanar angles (°) for **L5**.

Plane	Benzimidazole (1)	Benzimidazole (2)
Pyridine	44.65(12)	7.88(11)
Benzimidazole (1)		51.25(10)

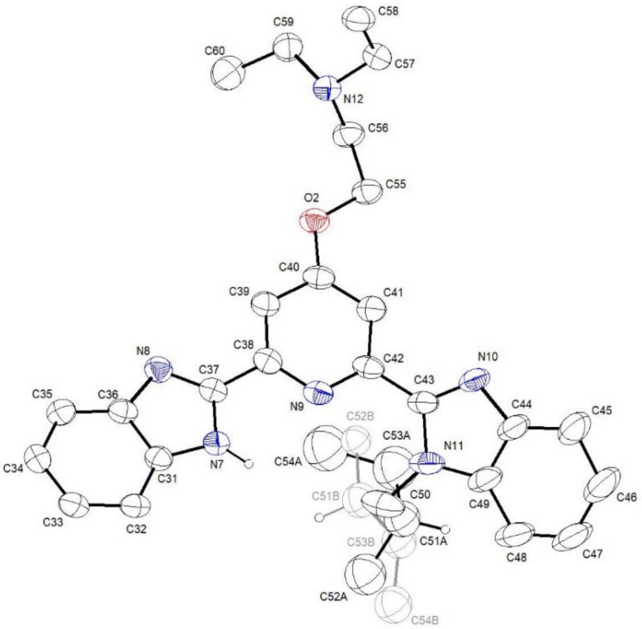

Figure A2-7. Ortep view of molecule 2 in **L5** (thermal ellipsoids are drawn at 50% probability level) with numbering scheme. Only H atoms on asymmetric C (C51A(*S*), C51B(*S*)) and on N atoms are shown here. Disordered alkyl chains are modeled in two parts are labeled A and B (in grey).

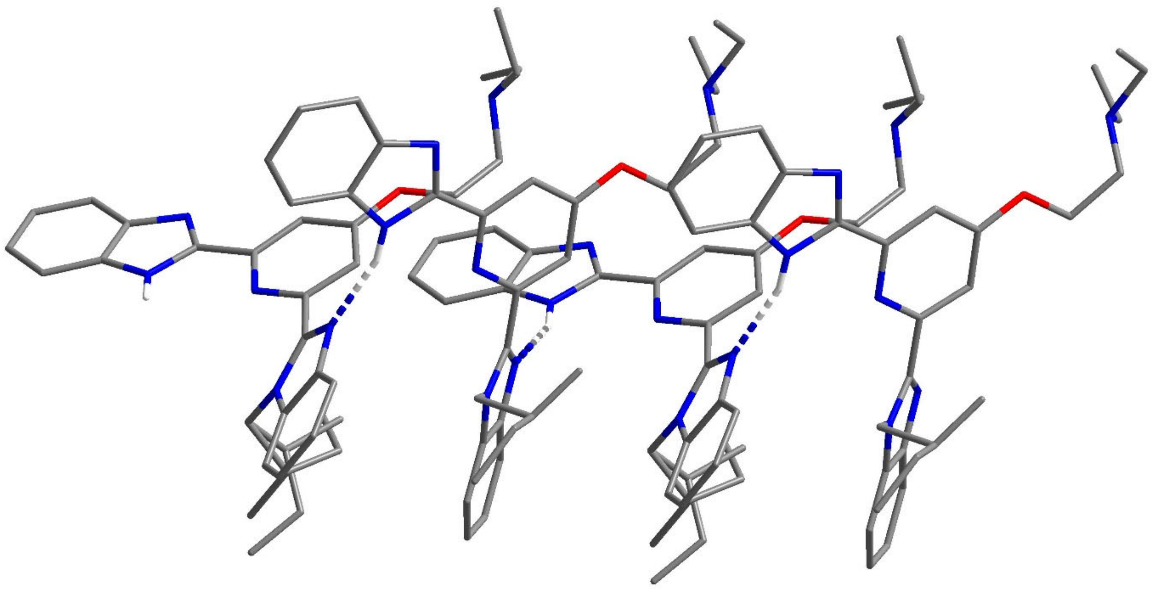

Figure A2-8. Hydrogen bonds in **L5**.

Table A2-19. Hydrogen bonds for **L5** [Å and °].

D-H...A	d(D-H)	d(H...A)	d(D...A)	<(DHA)
N(1)-H(1)...N(10)#1	0.88	2.04	2.882(5)	160.8
N(7)-H(7)...N(5)	0.88	2.03	2.865(4)	158.8

Symmetry transformations used to generate equivalent atoms:

#1 x,y,z-1

Table A2-20. Summary of crystal data, intensity measurements and structure refinements for [**L2**Eu(hfac)$_3$]·0.25CH_2Cl_2·0.5C_6H_{14}.

CCDC	2472893	
Empirical formula	$C_{85.25}H_{69.50}Cl_{0.50}Eu_2F_{36}N_{12}O_{12}$	
Chemical formula moiety	$2C_{41}H_{31}F_{18}N_6O_6Eu·0.25CH_2Cl_2·0.5C_6H_{14}$	
Formula weight	2459.67	
Temperature	99.99(14) K	
Wavelength	1.54184 Å	
Crystal system	Orthorhombic	
Space group	P b c a	
Unit cell dimensions	a = 14.50390(16) Å	α = 90°.
	b = 25.2261(3) Å	β = 90°.
	c = 52.9888(7) Å	γ = 90°.
Volume	19387.4(4) Å^3	
Z	8	
Density (calculated)	1.685 Mg/m^3	
Absorption coefficient	10.501 mm^{-1}	
F(000)	9756	
Crystal size	0.31 x 0.03 x 0.01 mm^3	
Theta range for data collection	3.474 to 74.514°.	
Index ranges	-17<=h<=10, -31<=k<=30, -65<=l<=64	
Reflections collected	104429	
Independent reflections	19499 [R(int) = 0.0975]	
Completeness to theta = 67.684°	99.8 %	
Absorption correction	Analytical	
Max. and min. transmission	0.891 and 0.255	
Refinement method	Full-matrix least-squares on F^2	
Data / restraints / parameters	19499 / 2 / 1378	
Goodness-of-fit on F^2	1.060	
Final R indices [I>2sigma(I)]	R1 = 0.0719, wR2 = 0.1872	
R indices (all data)	R1 = 0.1119, wR2 = 0.2103	
Extinction coefficient	n/a	
Largest diff. peak and hole	1.096 and -1.176 e.Å^{-3}	

Table A2-21. Selected least-squares planes data of molecule 1 in complex [**L2**Eu(hfac)$_3$]·0.25CH_2Cl_2·0.5C_6H_{14}.

	Abbreviation	RMSD (Å)	Max deviation (Å) (Atom)
Benzimidazole (1)<br>N1 C7 N2 C6 C1 C2 C3 C4 C5	Bz1	0.025	C1 (0.035)
Benzimidazole (2)<br>N5 C21 N6 C27 C22 C23 C24 C25 C26	Bz2	0.016	C23 (0.026)
Pyridine<br>N3 C18 C17 C16 C15 C14	Py	0.013	C18 (0.019)
Hexafluroacetylacetonate (1)<br>O1 C29 C30 C31 O2	Hfac1	0.017	C30 (0.024)
Hexafluroacetylacetonate (2)<br>O4 C36 C35 C34 O3	Hfac2	0.012	C34 (0.018)
Hexafluroacetylacetonate (3)<br>O5 C39 C40 C41 O6	Hfac3	0.014	C39 (0.020)

Table A2-22. Interplanar angles (°) for molecule 1 in [**L2**Eu(hfac)$_3$]·0.25CH_2Cl_2·0.5C_6H_{14}.

Plane	Benzimidazole (1)	Benzimidazole (2)	Hfac1	Hfac2	Hfac3
Pyridine	10.4(2)	5.6(2)	47.7(3)	44.9(3)	84.4(3)
Benzimidazole (1)		10.83(17)	45.5(3)	40.6(2)	87.2(2)
Benzimidazole (2)			42.1(3)	39.7(3)	88.5(2)
Hfac1				9.5(2)	58.6(3)
Hfac2					55.2(3)

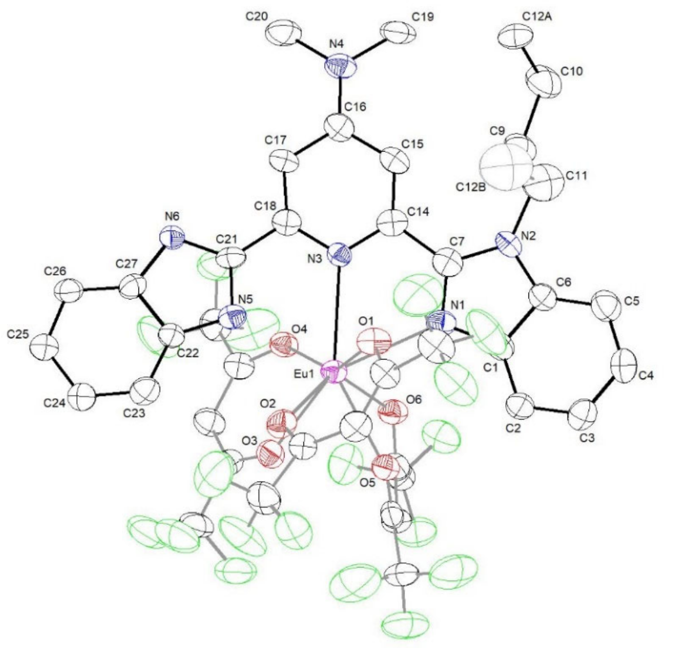

Figure A2-9. ORTEP view of molecule 1 in [**L2**Eu(hfac)$_3$]·0.25CH_2Cl_2·0.5C_6H_{14} (thermal ellipsoids are drawn at 50% probability level) with numbering scheme. Hydrogen atoms, solvent molecules and second complex is omitted for clarity.

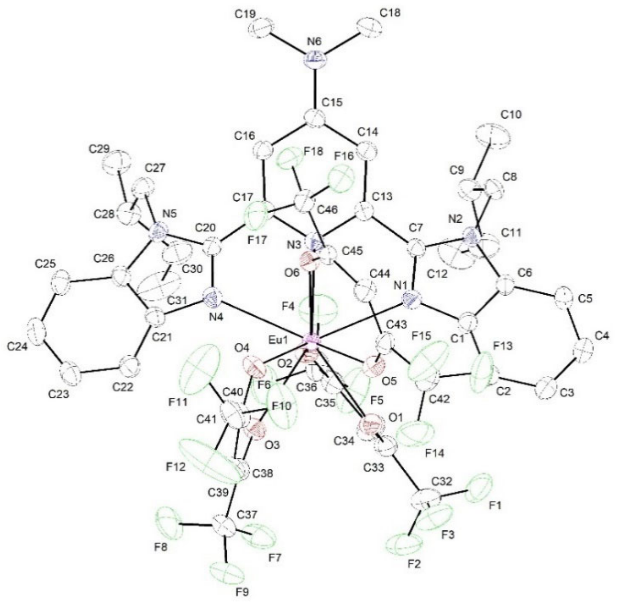

Figure A2-10. ORTEP view of molecule A in [**L3**Eu(hfac)$_3$] (thermal ellipsoids are drawn at 50% probability level) with numbering scheme. Hydrogen atoms are omitted for clarity purposes. All asymmetric carbons C9, C28 in molecule A and C9B, C28B in molecule B have *S* configuration.

Table A2-23. Summary of crystal data, intensity measurements and structure refinements for complex [**L3**Eu(hfac)$_3$].

CCDC	2472894	
Empirical formula	$C_{46}H_{41}F_{18}N_6O_6Eu$	
Chemical formula moiety	$C_{46}H_{41}F_{18}N_6O_6Eu$	
Formula weight	1268.00	
Temperature	120.00(10) K	
Wavelength	1.54184 Å	
Crystal system	Monoclinic	
Space group	P 2_1	
Unit cell dimensions	a = 10.60792(4) Å	α = 90°.
	b = 22.54293(8) Å	β = 102.7994(3)°.
	c = 21.89791(9) Å	γ = 90°.
Volume	5106.41(3) Å^3	
Z	4	
Density (calculated)	1.649 Mg/m^3	
Absorption coefficient	9.869 mm^{-1}	
F(000)	2528	
Crystal size	0.35 x 0.26 x 0.21 mm^3	
Theta range for data collection	2.850 to 75.987°.	
Index ranges	-13<=h<=10, -28<=k<=28, -25<=l<=27	
Reflections collected	89680	
Independent reflections	20661 [R(int) = 0.0386]	
Completeness to theta = 67.684°	100.0 %	
Absorption correction	Analytical	
Max. and min. transmission	0.313 and 0.131	
Refinement method	Full-matrix least-squares on F^2	
Data / restraints / parameters	20661 / 2 / 1404	
Goodness-of-fit on F^2	1.037	
Final R indices [I>2sigma(I)]	R1 = 0.0346, wR2 = 0.0905	
R indices (all data)	R1 = 0.0350, wR2 = 0.0907	
Absolute structure parameter	0.0028(16)	
Extinction coefficient	n/a	
Largest diff. peak and hole	0.692 and -0.968 e.Å^{-3}	

Table A2-24. Selected least-squares planes data of molecule A in [**L3**Eu(hfac)$_3$].

	Abbreviation	RMSD (Å)	Max deviation (Å) (Atom)
Benzimidazole (1)<br>N1 C7 N2 C6 C1 C2 C3 C4 C5	Bz1	0.023	N1 (0.038)
Benzimidazole (2)<br>N4 C20 N5 C26 C21 C22 C23 C24 C25	Bz2	0.019	C26 (0.032)
Pyridine<br>N3 C13 C14 C15 C16 C17	Py	0.021	C17 (0.028)
Hexafluroacetylacetonate (1)<br>O1 C33 C34 C35 O2	Hfac1	0.015	C33 (0.022)
Hexafluroacetylacetonate (2)<br>O3 C38 C39 C40 O4	Hfac2	0.008	C38 (0.013)
Hexafluroacetylacetonate (3)<br>O5 C43 C44 C45 O6	Hfac3	0.017	C45 (0.023)

Table A2-25. Interplanar angles (°) of molecule A in [**L3**Eu(hfac)$_3$].

Plane	Benzimidazole (1)	Benzimidazole (2)	Hfac1	Hfac2	Hfac3
Pyridine	27.33(13)	28.44(13)	88.99(19)	82.86(15)	68.92(19)
Benzimidazole (1)		22.51(10)	63.82(19)	76.91(11)	87.43(18)
Benzimidazole (2)			80.31(19)	80.88(12)	75.72(16)
Hfac1				53.42(18)	24.0(2)
Hfac2					51.20(18)

Table A2-26. Summary of crystal data, intensity measurements and structure refinements for [**L5**Eu(hfac)$_3$]·H_2O.

CCDC	2472895	
Empirical formula	$C_{45}H_{41}F_{18}N_6O_8Eu$	
Chemical formula moiety	$C_{45}H_{39}F_{18}N_6O_7Eu{\cdot}H_2O$	
Formula weight	1287.80	
Temperature	120.00(10) K	
Wavelength	1.54184 Å	
Crystal system	Monoclinic	
Space group	P 2_1	
Unit cell dimensions	a = 16.41248(9) Å	α = 90°.
	b = 26.89715(18) Å	β = 96.6752(6)°.
	c = 23.41428(16) Å	γ = 90°.
Volume	10266.14(11) Å^3	
Z	8	
Density (calculated)	1.666 Mg/m^3	
Absorption coefficient	9.858 mm^{-1}	
F(000)	5136	
Crystal size	0.67 x 0.06 x 0.02 mm^3	
Theta range for data collection	2.512 to 76.227°.	
Index ranges	-11<=h<=20, -33<=k<=33, -29<=l<=29	
Reflections collected	179643	
Independent reflections	41648 [R(int) = 0.0703]	
Completeness to theta = 67.684°	99.9 %	
Absorption correction	Analytical	
Max. and min. transmission	0.821 and 0.123	
Refinement method	Full-matrix least-squares on F^2	
Data / restraints / parameters	41648 / 6 / 2752	
Goodness-of-fit on F^2	1.064	
Final R indices [I>2sigma(I)]	R1 = 0.0599, wR2 = 0.1425	
R indices (all data)	R1 = 0.0704, wR2 = 0.1484	
Absolute structure parameter	0.004(3)	
Extinction coefficient	n/a	
Largest diff. peak and hole	0.891 and -0.897 e.Å^{-3}	

Table A2-27. Selected least-squares planes data of complex A in [**L5**Eu(hfac)$_3$]·H_2O.

	Abbreviation	RMSD (Å)	Max deviation (Å) (Atom)
Benzimidazole (1) N1B C7B N2B C6B C1B C2B C3B C4B C5B	Bz1	0.005	C1B (0.008)
Benzimidazole (2) N4B C13B N5B C19B C14B C15B C16B C17B C18B	Bz2	0.007	C14B (0.012)
Pyridine N3B C12B C11B C10B C9B C8B	Py	0.010	C10B (0.017)
Hexafluroacetylacetonate (1) O2B C32B C33B C34B O3B	Hfac1	0.001	C32B, C33B (0.002)
Hexafluroacetylacetonate (2) O4B C37B C38B C39B O5B	Hfac2	0.019	C37B (0.030)
Hexafluroacetylacetonate (3) O6B C42B C43B C44B O7B	Hfac3	0.019	C42B (0.025)

Table A2-28. Interplanar angles (°) for complex A in [**L5**Eu(hfac)$_3$]·H_2O.

Plane	Benzimidazole (1)	Benzimidazole (2)	Hfac1	Hfac2	Hfac3
Pyridine	1.1(3)	26.3(3)	71.5(4)	81.0(4)	81.0(3)
Benzimidazole (1)		26.1(3)	70.6(3)	80.2(4)	79.9(3)
Benzimidazole (2)			52.9(4)	60.2(4)	81.0(3)
Hfac1				10.9(4)	46.7(4)
Hfac2					51.9(4)

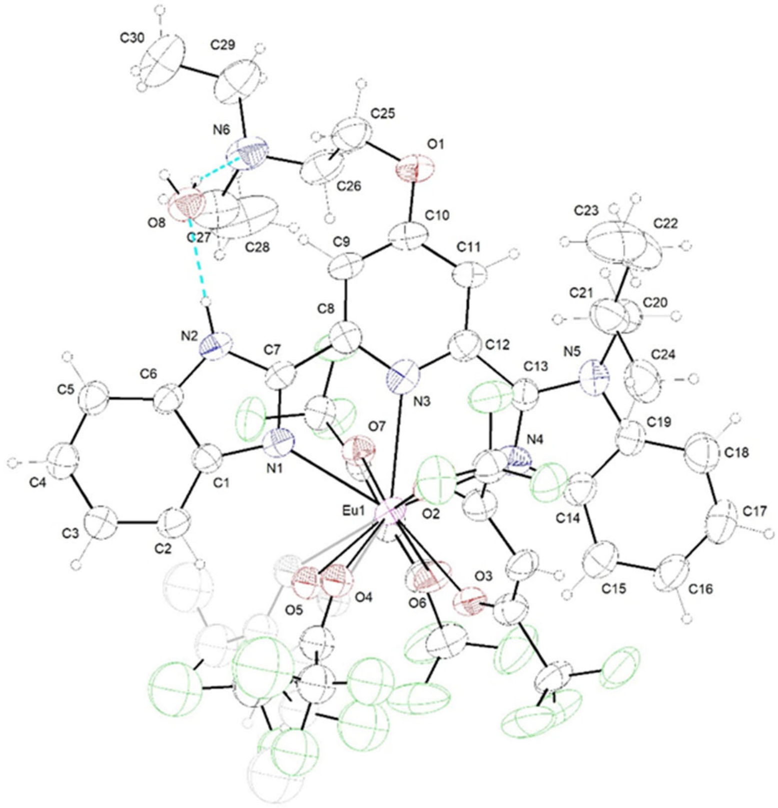

Figure A2-11. ORTEP view of molecule A in [**L5**Eu(hfac)$_3$]·H_2O (thermal ellipsoids are drawn at 50% probability level) with numbering scheme. Hydrogen bonds with water molecule are highlighted in blue. All asymmetric carbons in four independent molecules (C21 in molecule A) have *S* configuration.

Table A2-29. Hydrogen bonds for [**L5**Eu(hfac)$_3$]·H_2O [Å and °].

D-H...A	d(D-H)	d(H...A)	d(D...A)	<(DHA)
N(2)-H(2)...O(8)	0.88	1.95	2.828(11)	177.6
N(2C)-H(2C)...O(8C)	0.88	1.97	2.850(12)	177.2
N(2B)-H(2B)...O(8B)	0.88	1.91	2.793(12)	177.0
N(2D)-H(2D)...O(8D)	0.88	1.96	2.839(11)	178.3
O(8)-H(8A)...N(6)	0.87	2.08	2.906(13)	158.8
O(8C)-H(8CA)...N(6C)	0.87	2.45	2.820(13)	106.5
O(8D)-H(8DA)...N(6D)	0.87	2.06	2.896(13)	161.6
O(8B)-H(8BA)...N(6B)	0.87	2.35	2.837(13)	115.5

Table A2-30. Summary of crystal data, intensity measurements and structure refinements for [**L6**Eu(hfac)$_3$].

CCDC	2472896	
Empirical formula	$C_{50}H_{49}F_{18}N_6O_7Eu$	
Chemical formula moiety	$C_{50}H_{49}F_{18}N_6O_7Eu$	
Formula weight	1339.91	
Temperature	120.00(10) K	
Wavelength	1.54184 Å	
Crystal system	Monoclinic	
Space group	P 2_1	
Unit cell dimensions	a = 16.45120(12) Å	α = 90°.
	b = 19.51761(12) Å	β = 111.8517(8)°.
	c = 18.25669(13) Å	γ = 90°.
Volume	5440.82(7) Å^3	
Z	4	
Density (calculated)	1.636 Mg/m^3	
Absorption coefficient	9.312 mm^{-1}	
F(000)	2688	
Crystal size	0.32 x 0.18 x 0.04 mm^3	
Theta range for data collection	2.608 to 76.175°.	
Index ranges	-20<=h<=20, -24<=k<=24, -22<=l<=22	
Reflections collected	98820	
Independent reflections	21914 [R(int) = 0.0369]	
Completeness to theta = 67.684°	100.0 %	
Absorption correction	Analytical	
Max. and min. transmission	0.701 and 0.168	
Refinement method	Full-matrix least-squares on F^2	
Data / restraints / parameters	21914 / 1 / 1521	
Goodness-of-fit on F^2	1.041	
Final R indices [I>2sigma(I)]	R1 = 0.0333, wR2 = 0.0847	
R indices (all data)	R1 = 0.0343, wR2 = 0.0853	
Absolute structure parameter	-0.0054(16)	
Extinction coefficient	n/a	
Largest diff. peak and hole	0.416 and -1.158 e.Å^{-3}	

Table A2-31. Selected least-squares planes data of complex A in [**L6**Eu(hfac)$_3$].

	Abbreviation	RMSD (Å)	Max deviation (Å) (Atom)
Benzimidazole (1)<br>N1 C7 N2 C6 C1 C2 C3 C4 C5	Bz1	0.014	C2 (0.021)
Benzimidazole (2)<br>N4 C24 N5 C30 C25 C26 C27 C28 C29	Bz2	0.030	C24 (0.047)
Pyridine<br>N3 C17 C16 C15 C14 C13	Py	0.014	C16 (0.022)
Hexafluroacetylacetonate (1)<br>O2 C37 C38 C39 O3	Hfac1	0.044	C39 (0.059)
Hexafluroacetylacetonate (2)<br>O4 C42 C43 C44 O5	Hfac2	0.008	C42, C43 (0.011)
Hexafluroacetylacetonate (3)<br>O6 C47 C48 C49 O7	Hfac3	0.008	C47 (0.012)

Table A2-32. Interplanar angles (°) for complex A in [**L6**Eu(hfac)$_3$].

Plane	Benzimidazole (1)	Benzimidazole (2)	Hfac1	Hfac2	Hfac3
Pyridine	15.37(13)	31.23(13)	57.27(18)	39.08(14)	56.79(18)
Benzimidazole (1)		34.35(10)	57.09(16)	47.13(11)	72.12(18)
Benzimidazole (2)			88.36(18)	16.03(11)	60.06(17)
Hfac1				85.21(19)	80.12(16)
Hfac2					47.3(2)

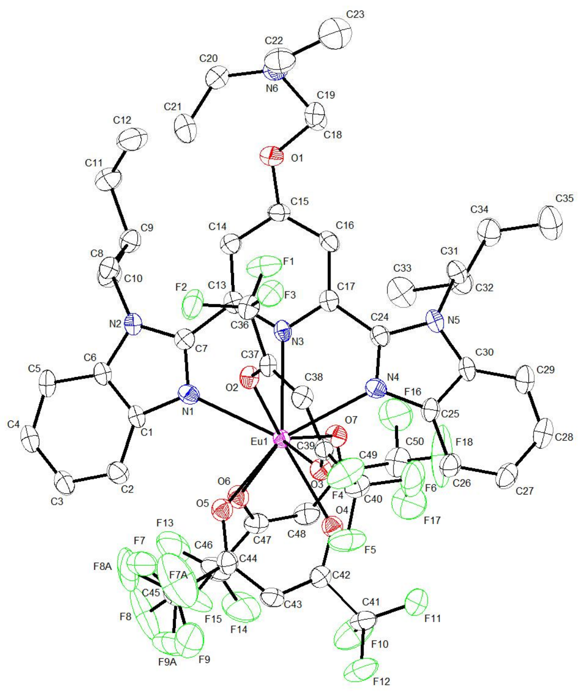

Figure A2-12. ORTEP view of complex A in [**L6**Eu(hfac)$_3$] (thermal ellipsoids are drawn at 50% probability level) with numbering scheme. Hydrogen atoms are omitted for clarity purposes. Asymmetric carbons (C9, C32 in molecule A and C9B, C32B in molecule B) have *S* configuration.

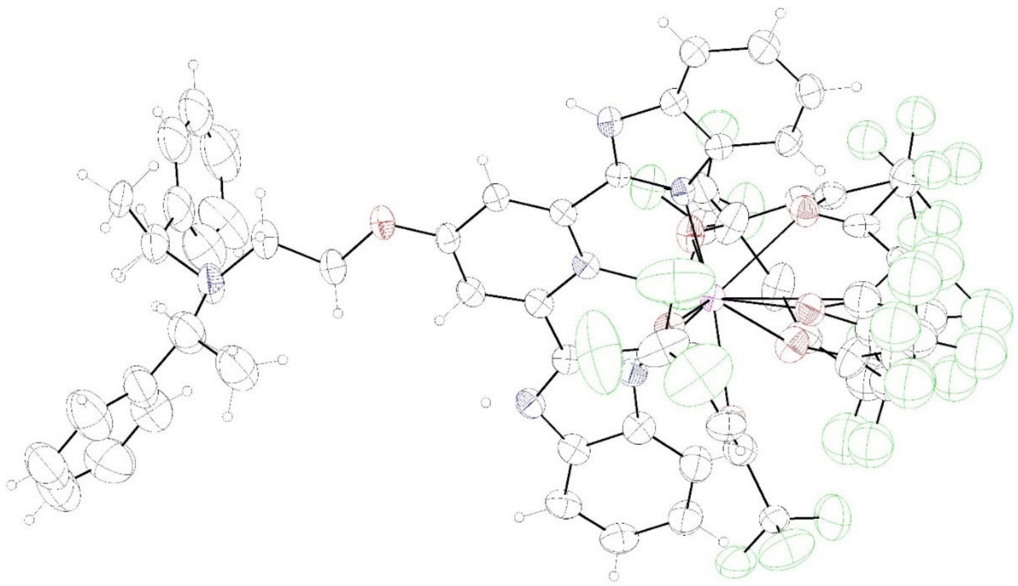

Figure A2-13. ORTEP view of [**L7**Eu(hfac)$_3$] (thermal ellipsoids are drawn at 50% probability level).

Table A2-33. Summary of crystal data, intensity measurements and structure refinements for [**L7**Eu(hfac)$_3$].

CCDC	2472897	
Empirical formula	$C_{54.75}H_{41.75}EuF_{18}N_6O_7$	
Formula weight	1389.65	
Temperature	99.98(13) K	
Wavelength	1.54184 Å	
Crystal system	Triclinic	
Space group	P -1	
Unit cell dimensions	a = 11.44460(10) Å	α = 102.8090(10)°.
	b = 12.73620(10) Å	β = 96.8480(10)°.
	c = 21.2364(2) Å	γ = 107.4540(10)°.
Volume	2821.45(5) Å^3	
Z	2	
Density (calculated)	1.636 Mg/m^3	
Absorption coefficient	9.008 mm^{-1}	
F(000)	1386	
Crystal size	0.61 x 0.15 x 0.07 mm^3	
Theta range for data collection	2.177 to 76.195°.	
Index ranges	-13<=h<=14, -15<=k<=15, -26<=l<=26	
Reflections collected	72066	
Independent reflections	11454 [R(int) = 0.0576]	
Completeness to theta = 67.684°	99.9 %	
Absorption correction	Semi-empirical from equivalents	
Max. and min. transmission	1.00000 and 0.37926	
Refinement method	Full-matrix least-squares on F^2	
Data / restraints / parameters	11454 / 5 / 798	
Goodness-of-fit on F^2	1.069	
Final R indices [I>2sigma(I)]	R1 = 0.0688, wR2 = 0.1935	
R indices (all data)	R1 = 0.0709, wR2 = 0.1960	
Extinction coefficient	n/a	
Largest diff. peak and hole	1.886 and -1.803 e.Å^{-3}	

Table A2-34. Selected least-squares planes data of complex [**L7**Eu(hfac)$_3$].

	Abbreviation	RMSD (Å)	Max deviation (Å) (Atom)
Benzimidazole (1)<br>N1 C7 N2 C6 C1 C2 C3 C4 C5	Bz1	0.010	C3 (0.014)
Benzimidazole (2)<br>N4 C13 N5 C14 C19 C18 C17 C16 C15	Bz2	0.005	C13 (0.009)
Pyridine<br>N3 C12 C11 C10 C9 C8	Py	0.002	C8, C9 (0.002)
Hexafluroacetylacetonate (1)<br>O2 C38 C39 C40 O3	Hfac1	0.037	C38 (0.047)
Hexafluroacetylacetonate (2)<br>O4 C43 C44 C45 O5	Hfac2	0.023	C45 (0.035)
Hexafluroacetylacetonate (3)<br>O6 C48 C49 C50 O7	Hfac3	0.007	C50 (0.011)

Table A2-35. Interplanar angles (°) for [**L7**Eu(hfac)$_3$].

Plane	Benzimidazole (1)	Benzimidazole (2)	Hfac1	Hfac2	Hfac3
Pyridine	1.67(19)	3.86(16)	60.0(2)	72.9(3)	39.1(2)
Benzimidazole (1)		2.57(14)	59.83(19)	71.3(3)	39.1(2)
Benzimidazole (2)			57.8(2)	69.6(2)	35.5(2)
Hfac1				76.1(2)	33.7(3)
Hfac2					50.7(4)

Table A2-36. Summary of crystal data, intensity measurements and structure refinements for Michael product **3** at 180 K (cooling).

CCDC	2472899
Empirical formula	$C_{15}H_{19}NO_6$
Crystal system	monoclinic
Space group	$P2_1/n$
a/Å	5.95539(13)
b/Å	30.6792(6)
c/Å	9.0684(2)
α/°	90
β/°	102.590(2)
γ/°	90
Volume/Å^3	1617.02(6)
Z	4
ρ_{calc}g/cm^3	1.271
μ/mm^{-1}	0.831
F(000)	656.0
Crystal size/mm^3	0.481 × 0.123 × 0.049
Radiation	Cu Kα (λ = 1.54184)
2Θ range for data collection/°	5.762 to 140.026
Index ranges	$-4 \leq h \leq 7$, $-37 \leq k \leq 36$, $-10 \leq l \leq 11$
Reflections collected	6849
Independent reflections	2985 [R_{int} = 0.0107, R_{sigma} = 0.0139]
Data/restraints/parameters	2985/0/201
Goodness-of-fit on F^2	1.064
Final R indexes [I>=2σ (I)]	R_1 = 0.0561, wR_2 = 0.1564
Final R indexes [all data]	R_1 = 0.0618, wR_2 = 0.1617
Largest diff. peak/hole / e Å^{-3}	0.72/-0.36

Table A2-37. Summary of crystal data, intensity measurements and structure refinements for Michael product **3** at 150 K.

CCDC	2472900
Formula weight	309.31
Temperature/K	150.06(15)
Crystal system	monoclinic
Space group	$P2_1/n$
a/Å	5.77540(15)
b/Å	30.4471(7)
c/Å	9.2827(2)
α/°	90
β/°	103.455(3)
γ/°	90
Volume/Å^3	1587.51(7)
Z	4
ρ_{calc}g/cm^3	1.294
μ/mm^{-1}	0.846
F(000)	656.0
Crystal size/mm^3	0.497 × 0.123 × 0.072
Radiation	Cu Kα (λ = 1.54184)
2Θ range for data collection/°	5.806 to 140.118
Index ranges	$-7 \le h \le 4$, $-36 \le k \le 36$, $-11 \le l \le 11$
Reflections collected	6657
Independent reflections	2839 [R_{int} = 0.0132, R_{sigma} = 0.0169]
Data/restraints/parameters	2839/0/285
Goodness-of-fit on F^2	1.049
Final R indexes [I>=2σ (I)]	R_1 = 0.0440, wR_2 = 0.1103
Final R indexes [all data]	R_1 = 0.0498, wR_2 = 0.1156
Largest diff. peak/hole / e Å^{-3}	0.23/-0.28

Table A2-38. Summary of crystal data, intensity measurements and structure refinements for Michael product **3** at 120 K.

CCDC	2472901
Formula weight	309.31
Temperature/K	119.98(16)
Crystal system	monoclinic
Space group	$P2_1/c$
a/Å	11.5027(3)
b/Å	30.3509(6)
c/Å	9.6889(2)
α/°	90
β/°	111.568(3)
γ/°	90
Volume/Å^3	3145.72(14)
Z	8
ρ_{calc}g/cm^3	1.306
μ/mm^{-1}	0.854
F(000)	1312.0
Crystal size/mm^3	0.499 × 0.133 × 0.059
Radiation	Cu Kα (λ = 1.54184)
2Θ range for data collection/°	5.824 to 140.09
Index ranges	$-8 \leq h \leq 13$, $-36 \leq k \leq 36$, $-11 \leq l \leq 9$
Reflections collected	12936
Independent reflections	5575 [R_{int} = 0.0211, R_{sigma} = 0.0260]
Data/restraints/parameters	5575/0/401
Goodness-of-fit on F^2	1.029
Final R indexes [I>=2σ (I)]	R_1 = 0.0880, wR_2 = 0.2315
Final R indexes [all data]	R_1 = 0.0957, wR_2 = 0.2426
Largest diff. peak/hole / e Å^{-3}	0.76/-0.32

Table A2-39. Summary of crystal data, intensity measurements and structure refinements for Michael product **3** at 180 K (heating).

Empirical formula	$C_{15}H_{19}NO_6$
Formula weight	309.31
Temperature/K	180.00(10)
Crystal system	monoclinic
Space group	$P2_1/n$
a/Å	5.9421(5)
b/Å	30.715(2)
c/Å	9.0666(10)
α/°	90
β/°	102.402(10)
γ/°	90
Volume/Å^3	1616.2(3)
Z	4
ρ_{calc}g/cm^3	1.271
μ/mm^{-1}	0.831
F(000)	656.0
Crystal size/mm^3	0.505 × 0.121 × 0.063
Radiation	Cu Kα (λ = 1.54184)
2Θ range for data collection/°	5.754 to 140.286
Index ranges	$-7 \leq h \leq 4$, $-37 \leq k \leq 36$, $-11 \leq l \leq 10$
Reflections collected	6313
Independent reflections	2889 [R_{int} = 0.0265, R_{sigma} = 0.0269]
Data/restraints/parameters	2889/0/201
Goodness-of-fit on F^2	1.141
Final R indexes [I>=2σ (I)]	R_1 = 0.0895, wR_2 = 0.2799
Final R indexes [all data]	R_1 = 0.1089, wR_2 = 0.3105
Largest diff. peak/hole / e Å^{-3}	0.53/-0.38

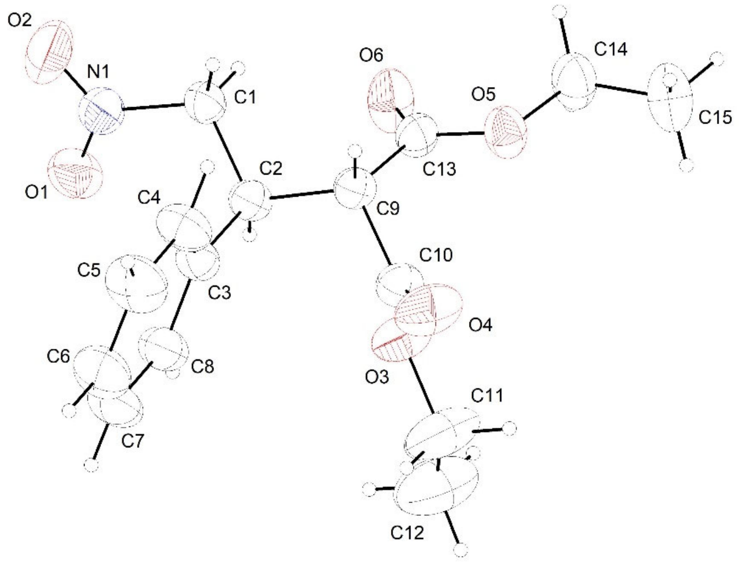

Figure A2-14. ORTEP view of compound **3** at 180 K (thermal ellipsoids are drawn at 50% probability level) with numbering scheme.

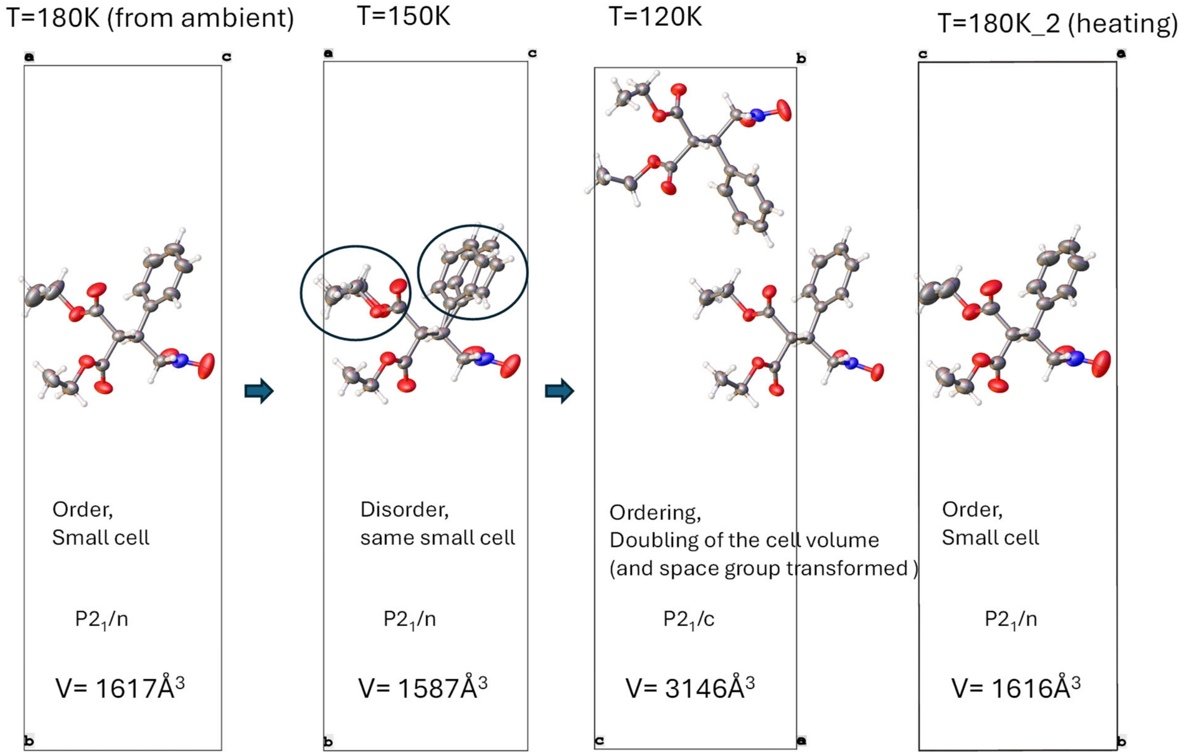

Figure A2-15. Crystal structure and asymmetric unit of compound **3** at different temperatures (viewed along *a*).

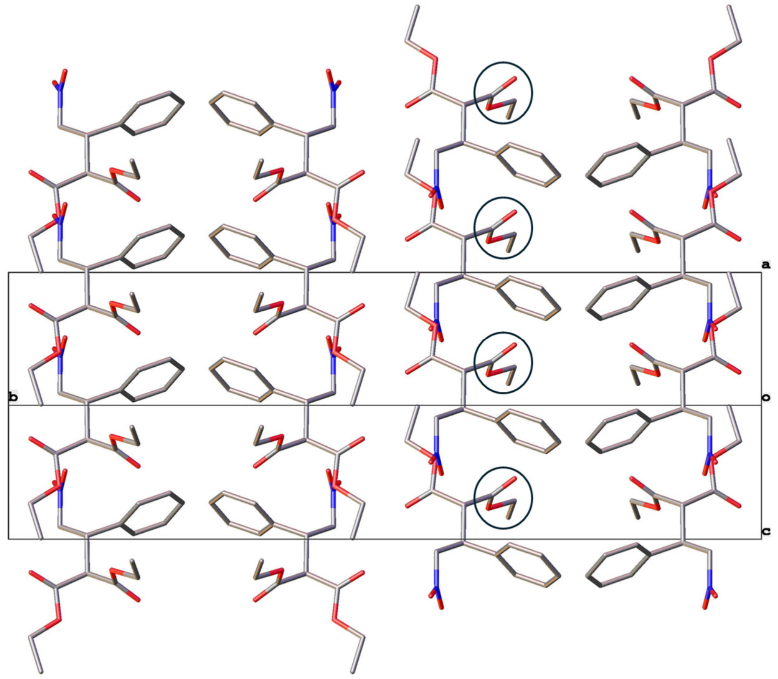

Figure A2-16. Crystal structure, asymmetric unit, and cell view along 101 direction of compound **3** at 180 K. Black circles highlighting periodic orientation of carboalkoxy group.

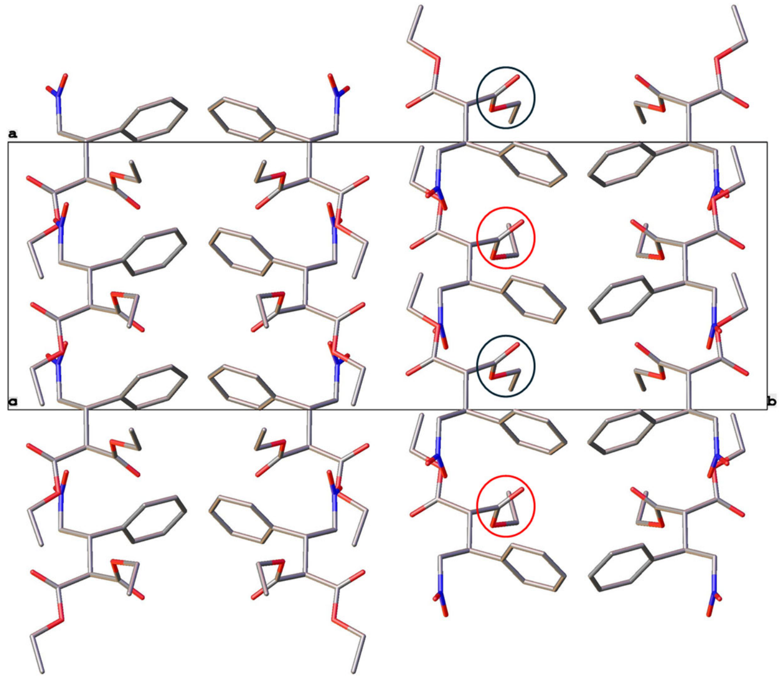

Figure A2-17. Crystal structure, asymmetric unit, and cell view along 001 direction of compound **3** at 120 K. Black and red circles highlighting periodic orientations of carboalkoxy group responsible for doubling cell volume.

Table A2-40. Summary of crystal data, intensity measurements and structure refinements for [**L8**Eu(hfac)$_3$]·0.25CH_2Cl_2.

CCDC	2472898	
Empirical formula	$C_{61.25}H_{53.50}Cl_{0.50}EuF_{18}N_6O_7$	
Chemical formula moiety	$C_{61}H_{53}F_{18}N_6O_7Eu \cdot 0.25CH_2Cl_2$	
Formula weight	1497.28	
Temperature	150.00(10) K	
Wavelength	1.54184 Å	
Crystal system	Triclinic	
Space group	P -1	
Unit cell dimensions	a = 13.84187(9) Å	α= 67.6660(7)°.
	b = 16.00060(12) Å	β= 77.4988(6)°.
	c = 16.99570(11) Å	γ = 66.8835(7)°.
Volume	3192.22(4) Å^3	
Z	2	
Density (calculated)	1.558 Mg/m^3	
Absorption coefficient	8.194 mm^{-1}	
F(000)	1505	
Crystal size	0.7 x 0.14 x 0.13 mm^3	
Theta range for data collection	2.820 to 76.009°.	
Index ranges	-17<=h<=16, -19<=k<=14, -21<=l<=20	
Reflections collected	87394	
Independent reflections	12967 [R(int) = 0.0474]	
Completeness to theta = 67.684°	99.9 %	
Absorption correction	Analytical	
Max. and min. transmission	0.447 and 0.116	
Refinement method	Full-matrix least-squares on F^2	
Data / restraints / parameters	12967 / 5 / 904	
Goodness-of-fit on F^2	1.021	
Final R indices [I>2sigma(I)]	R1 = 0.0618, wR2 = 0.1698	
R indices (all data)	R1 = 0.0637, wR2 = 0.1713	
Extinction coefficient	n/a	
Largest diff. peak and hole	1.570 and -1.184 e.Å^{-3}	

Table A2-41. Selected least-squares planes data of complex [**L8**Eu(hfac)$_3$]·0.25CH$_2$Cl$_2$.

	Abbreviation	RMSD (Å)	Max deviation (Å) (Atom)
Benzimidazole (1) N1 C7 N2 C6 C1 C2 C3 C4 C5	Bz1	0.017	C1, C7 (0.024)
Benzimidazole (2) N5 C18 N4 C19 C24 C23 C22 C21 C20	Bz2	0.022	C18 (0.030)
Pyridine N3 C17 C16 C15 C14 C13	Py	0.007	C15, C16 (0.010)
Hexafluroacetylacetonate (1) O2 C48 C49 C50 O3	Hfac1	0.014	C49 (0.020)
Hexafluroacetylacetonate (2) O4 C53A C54A C55A O5	Hfac2	0.021	C53A (0.033)
Hexafluroacetylacetonate (3) O6 C58A C59A C60A O7	Hfac3	0.002	C60A, O7 (0.003)

Table A2-42. Interplanar angles (°) for [**L8**Eu(hfac)$_3$]·0.25CH$_2$Cl$_2$.

Plane	Benzimidazole (1)	Benzimidazole (2)	Hfac1	Hfac2	Hfac3
Pyridine	9.20(19)	14.17(19)	54.2(3)	82.7(2)	64.1(3)
Benzimidazole (1)		18.72(15)	45.1(2)	88.1(2)	55.8(3)
Benzimidazole (2)			57.8(2)	88.29(19)	62.6(3)
Hfac1				63.0(3)	19.3(3)
Hfac2					44.0(3)

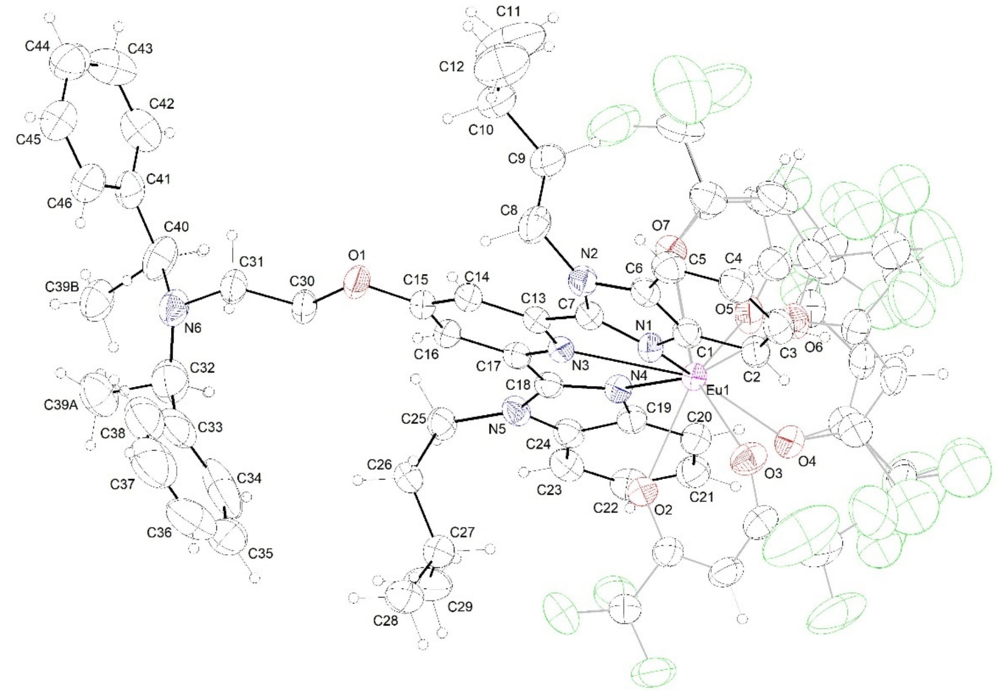

Figure A2-18. ORTEP view of [**L8**Eu(hfac)$_3$] (thermal ellipsoids are drawn at 50% probability level) with numbering scheme.

Table A2-43. Specific rotation $[\alpha]_D^{20}$ recorded in CH_2Cl_2 solution and absolute configuration found in the solid state crystal structures of the target compounds.

Compound	$[\alpha]_D^{20}$ /CH_2Cl_2	Absolute configuration (crystal)	Compound	$[\alpha]_D^{20}$ /CH_2Cl_2	Absolute configuration (crystal)
**L2**	+3.15 (c = 1.05)	*S*	[**L2**Eu(hfac)$_3$]	-3.97 (c = 0.37)	racemic
**L3**	-2.34 (c = 1.08)	S	[**L3**Eu(hfac)$_3$]	-9.38 (c = 0.98)	*S*
**L5**	-1.18 (c = 1.12)	*S*	[**L5**Eu(hfac)$_3$]	+1.39 (c = 0.52)	*S*
**L6**	-3.68 (c = 1.58)	[a]	[**L6**Eu(hfac)$_3$]	-5.27 (c = 0.57)	*S*
**L7**	-47.85 (c = 0.79)	[a]	[**L7**Eu(hfac)$_3$]	-17.01 (c = 0.46)	racemic
**L8**	-42.74 (c = 0.74)	[a]	[**L8**Eu(hfac)$_3$]	-14.10 (c = 0.68)	racemic

[a] No available crystal structure.

Appendix 3: Thermodynamic properties of L7 and L8 receptors with [Eu(hfac)3] guest in dichloromethane.

The binding isotherms rebuilt from obtained stability constant $\beta_{1,1,\mathrm{cond}}^{\mathbf{L}\boldsymbol{k},\mathrm{Eu}}$ (dashed-red traces in Figure 4a in main text and Figure A3-1a) differ from the experimental curves (black diamonds in Figure 4a in main text and Figure A3-1a) due to the variation in the activity coefficients γ_i occurring in non-ideal organic solutions according to eq (A3-1),[45] where c^{θ} = 1 M stands for the concentration of the reference state.

$$\beta_{1,1,\mathrm{cond}}^{\mathbf{L}\boldsymbol{k},\mathrm{Eu}} = \frac{a_{\mathbf{L}\boldsymbol{k}\mathrm{Eu}}^{\mathrm{eq}}}{a_{\mathrm{Eu}}^{\mathrm{eq}} a_{\mathbf{L}\boldsymbol{k}}^{\mathrm{eq}}} = \frac{\gamma_{\mathbf{L}\boldsymbol{k}\mathrm{Eu}}}{\gamma_{\mathrm{Eu}}\gamma_{\mathbf{L}\boldsymbol{k}}} \cdot \frac{\left(c_{\mathbf{L}\boldsymbol{k}\mathrm{Eu}}^{\mathrm{eq}}/c^{\theta}\right)}{\left(c_{\mathrm{Eu}}^{\mathrm{eq}}/c^{\theta}\right)\left(c_{\mathbf{L}\boldsymbol{k}}^{\mathrm{eq}}/c^{\theta}\right)} = \frac{\gamma_{\mathbf{L}\boldsymbol{k}\mathrm{Eu}}}{\gamma_{\mathrm{Eu}}\gamma_{\mathbf{L}\boldsymbol{k}}} \cdot \left(\frac{|\mathbf{L}\boldsymbol{k}\mathrm{Eu}|}{|\mathrm{Eu}||\mathbf{L}\boldsymbol{k}|}\right) \cdot c^{\theta}$$

$$= \frac{\gamma_{\mathbf{L}\boldsymbol{k}\mathrm{Eu}}}{\gamma_{\mathrm{Eu}} \cdot \gamma_{\mathbf{L}\boldsymbol{k}}} \cdot Q_{1,1,\mathrm{cond}}^{\mathbf{L}\boldsymbol{k},\mathrm{Eu}} \cdot c^{\theta} \qquad \text{(A3-1)}$$

Eggers and coworkers proposed eq (A3-2) to catch these variations during the host-guest interactions,[64,65] where the experimental quotients of reaction $Q_{1,1,\mathrm{cond}}^{\mathbf{L}\boldsymbol{k},\mathrm{Eu}} = \left(\frac{|\mathbf{L}\boldsymbol{k}\mathrm{Eu}|}{|\mathrm{Eu}||\mathbf{L}\boldsymbol{k}|}\right)$ can be easily estimated at each point of ^{1}H RMN titration.

$$-RT\ln(Q_{1,1,\mathrm{cond}}^{\mathbf{L}\boldsymbol{k},\mathrm{Eu}}) = -RT\ln(\beta_{1,1,\mathrm{cond}}^{\mathbf{L}\boldsymbol{k},\mathrm{Eu},\infty}) + \Delta G_{1,1,\mathrm{cond}}^{\mathbf{L}\boldsymbol{k},\mathrm{Eu,S}} \cdot (|\mathbf{L}\boldsymbol{k}\mathrm{Eu}|/c^{\theta}) \qquad \text{(A3-2)}$$

The plots of $-RT\ln(Q_{1,1,\mathrm{cond}}^{\mathbf{L}\boldsymbol{k},\mathrm{Eu}})$ versus the concentration of formed final complex $|\mathbf{L}\boldsymbol{k}\mathrm{Eu}|$ are close to linear (Fig. A3-1b) and provide free energy changes at infinite dilution $\Delta G_{1,1,\mathrm{cond}}^{\mathbf{L}\boldsymbol{k},\mathrm{Eu},\infty} = -RT\ln(\beta_{1,1,\mathrm{cond}}^{\mathbf{L}\boldsymbol{k},\mathrm{Eu},\infty})$ and solvation free energy changes $\Delta G_{1,1,\mathrm{cond}}^{\mathbf{L}\boldsymbol{k},\mathrm{Eu,S}}$ (Table A3-1), from which satisfying occupancy factors $\theta_{\mathbf{L}\boldsymbol{k}}^{\mathrm{Eu}}$ could be rebuilt with eq (A3-3) (green dashed trace in Figure A3-1a).

$$\theta_{\mathbf{L}\boldsymbol{k}}^{\mathrm{Eu}} = \frac{Q_{1,1,\mathrm{cond}}^{\mathrm{Eu},\mathbf{L}\boldsymbol{k}}\left|\mathrm{Eu}\right|}{1+Q_{1,1,\mathrm{cond}}^{\mathrm{Eu},\mathbf{L}\boldsymbol{k}}\left|\mathrm{Eu}\right|} = \frac{\left|\mathbf{L}\boldsymbol{k}\mathrm{Eu}\right|}{\left|\mathbf{L}\boldsymbol{k}\right|_{\mathrm{tot}}} = \frac{\left|\mathrm{Eu}\right|_{\mathrm{tot}}-\left|\mathrm{Eu}\right|}{\left|\mathbf{L}\boldsymbol{k}\right|_{\mathrm{tot}}}$$

$$= \frac{\exp\left[-\left(\Delta G_{1,1,\mathrm{cond}}^{\mathrm{Eu},\mathbf{L}\boldsymbol{k},\infty}+\left(\left|\mathrm{Eu}\right|_{\mathrm{tot}}-\left|\mathrm{Eu}\right|\right)\cdot\Delta G_{1,1,\mathrm{cond}}^{\mathrm{Eu},\mathbf{L}\boldsymbol{k},\mathrm{S}}\right)/RT\right]\cdot\left|\mathrm{Eu}\right|}{1+\left\{\exp\left[-\left(\Delta G_{1,1,\mathrm{cond}}^{\mathrm{Eu},\mathbf{L}\boldsymbol{k},\infty}+\left(\left|\mathrm{Eu}\right|_{\mathrm{tot}}-\left|\mathrm{Eu}\right|\right)\cdot\Delta G_{1,1,\mathrm{cond}}^{\mathrm{Eu},\mathbf{L}\boldsymbol{k},\mathrm{S}}\right)/RT\right]\cdot\left|\mathrm{Eu}\right|\right\}} \quad \text{(A3-3)}$$

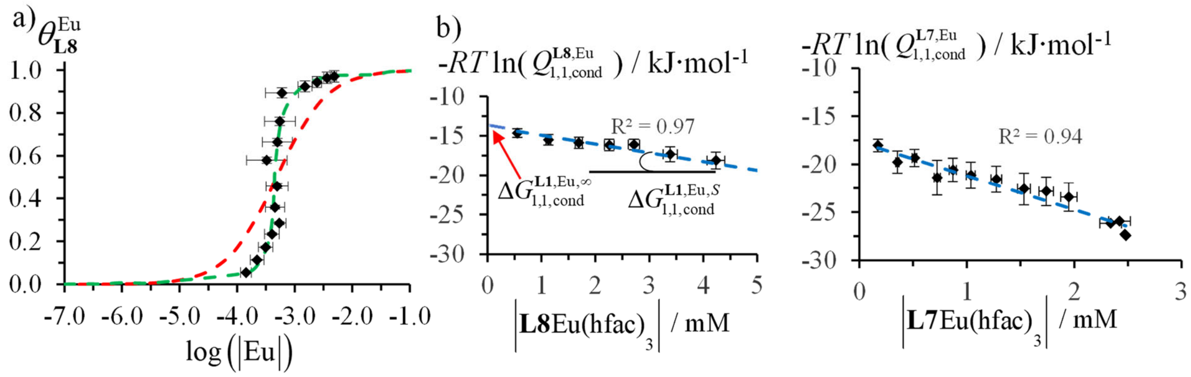

Figure A3-1 a) Experimental (black diamonds) and fitted binding isotherms using eq 3 in the main text (dashed-red trace) and A3-3 (dased-green trace) for the titration of **L8** with [**dig**Eu(hfac)3]. b) Plots of $-RT\ln(Q_{1,1,\mathrm{cond}}^{\mathbf{L}\boldsymbol{k},\mathrm{Ln}})$ as a function of $\left|\mathbf{L}\boldsymbol{k}\mathrm{Ln(hfac)}_3\right|$ for the titrations of **L7** and **L8** with [**dig**Eu(hfac)3] in CD_2Cl_2 + 0.14 M diglyme at 293K.

Table A3-1. Associated free energies $\Delta G_{1,1,\mathrm{cond}}^{\mathbf{L}\boldsymbol{k},\mathrm{Eu}} = -RT\ln(\beta_{1,1,\mathrm{cond}}^{\mathbf{L}\boldsymbol{k},\mathrm{Eu}})$, $\Delta G_{1,1,\mathrm{cond}}^{\mathbf{L}\boldsymbol{k},\mathrm{Eu},\infty}$ and $\Delta G_{1,1,\mathrm{cond}}^{\mathbf{L}\boldsymbol{k},\mathrm{Eu,S}}$ determined for the titrations of ligands **L7**, and **L8** with [**dig**Eu(hfac)3] in CD_2Cl_2 + 0.14 M diglyme at 293 K.

	**L7**	**L8**
$\Delta G_{1,1,\mathrm{cond}}^{\mathbf{L}\boldsymbol{k},\mathrm{Eu}}$ /kJ·mol^{-1}	-22.0(1)	-18.6(2)
$\Delta G_{1,1,\mathrm{cond}}^{\mathbf{L}\boldsymbol{k},\mathrm{Eu},\infty}$ /kJ·mol^{-1}	-17.7(4)	-14.0(3)
$\Delta G_{1,1,\mathrm{cond}}^{\mathbf{L}\boldsymbol{k},\mathrm{Eu,S}}$ /kJ·mol^{-1}	$-3.4(2)\cdot10^3$	$-10.5(5)\cdot10^2$

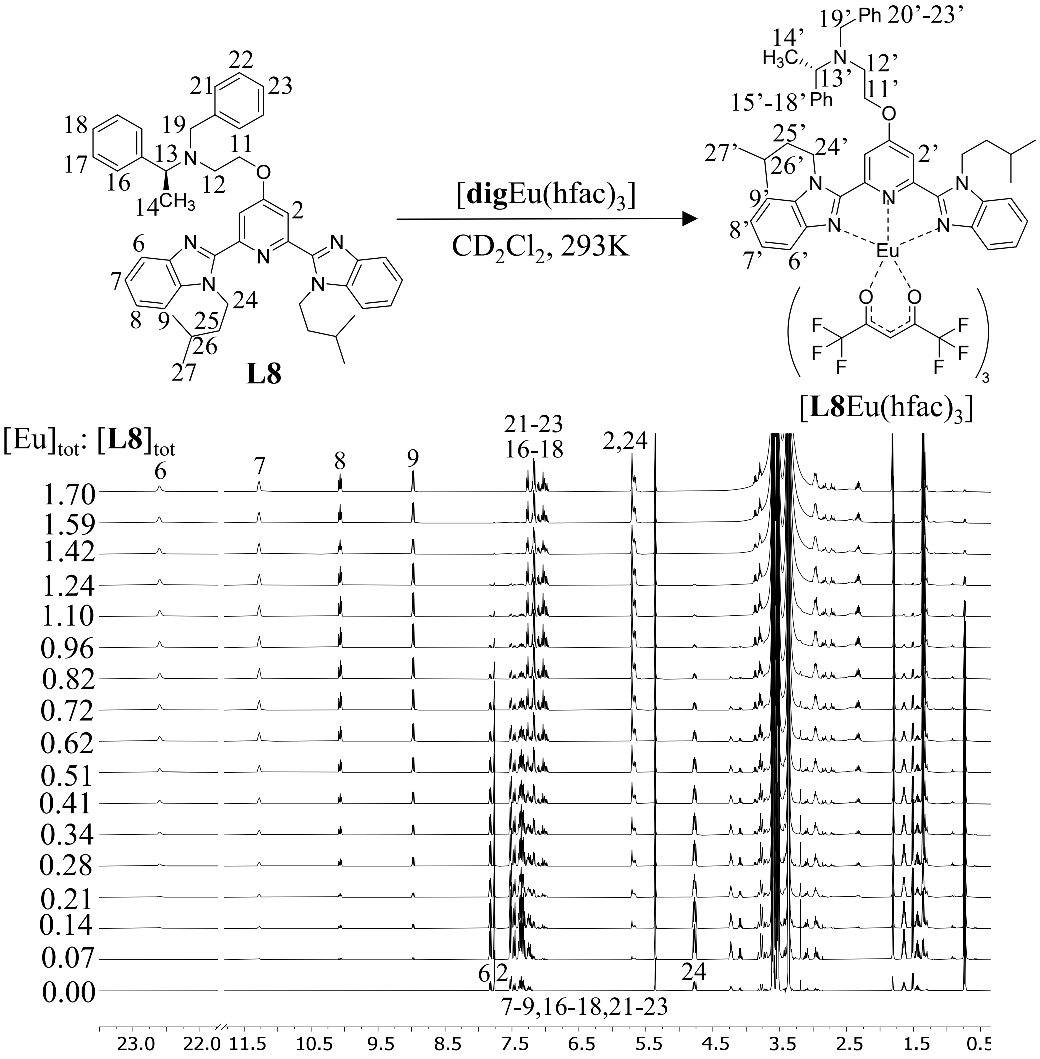

Figure A3-2. ^{1}H NMR titration of **L8** (10.1 mM) with [**dig**Eu(hfac)3] in CD_2Cl_2 + 0.14 M diglyme at 293 K.

Appendix 4: Photophysical parameters of L*k* ligands and their lanthanide complexes

Table A4-1 Photophysical parameters for free ligands in CH_2Cl_2 solution at various temperatures.

Compound	*T*/K	*E*/cm^{-1} Absorption $^1\pi^*\leftarrow{}^1\pi$	*E*/cm^{-1} Fluorescence $^1\pi^*\rightarrow{}^1\pi$	*E*/cm^{-1} Phosphorescence $^3\pi^*\rightarrow{}^1\pi$	*E*/cm^{-1} Stokes's shift
**L1**	293	33223 (45500)[a]	25000		8223
	77		20704		
**L2**	293	33557 (47915)	25063		8494
	77		25000 sh		
			21008		
**L3**	293	34014 (48020)	25510		8504
	77		25974		
			21097	20921	
**L4**	293	31348 (38301)	27548		3800
	77		27473 sh		
			24331		
**L5**	293	31646 (34587)	27473		4173
	77		27701 sh		
			24570		
**L6**	293	32154 (36423)	27548		4606
	77		27855		
**L7**	293	31546 (31890)	28818		3998
			27548		
	77		25000		
**L8**	293	32362 (32757)	27548		4814
	77		28571		
**L9**	293	30488 (55185)	24450		6038
			27027 sh		
	77		25773		
			24570 sh		
			18282 sh	18382	
**L10**	293	30864 (40628)	28249		3764
			27100		
	77		27933 sh		
			21277		
			20408		
			19841		

[a]Molar absorption coefficients are given in parenthesis

Table A4-2 Photophysical parameters for yttrium complexes in CH_2Cl_2 solution at various temperatures.

Compound	T/K	E/cm^{-1} Absorption $^1\pi^* \leftarrow {}^1\pi$	E/cm^{-1} Fluorescence $^1\pi^* \rightarrow {}^1\pi$	E/cm^{-1} Phosphorescence $^3\pi^* \rightarrow {}^1\pi$	E/cm^{-1} Stokes's shift
[**L3**Y(hfac)$_3$]	293	32895 (90278)[a]	24814		8081
	77		24510		
			21552	21598	
			20576	20243	
			20161		
[**L5**Y(hfac)$_3$]	293	32895 (66685)	25907		2829
		28736 (23073)			
	77		26247		
			21552		
			20121		
			17986		
[**L7**Y(hfac)$_3$]	293	33003 (57739)	26178		2640
		28818 (21988)	18083		10735
	77		26316		
			25641		
			25062		
			21598		
			21413		
			20161		
			19646		
[**L8**Y(hfac)$_3$]	293	32895 (64851)	25907		2746
		28653 (20802)	18051		10602
	77		26178		
			21552		
			19960		
			18622		
[**L9**Y(hfac)$_3$]	293	31746 (47049)	23810		3814
		27624 (25899)			
	77		25.641		
			24.390		
			17.575	18349	
				17271	
				16313	
[**L10**Y(hfac)$_3$]	293	32895 (57168)	24096		4233
		28329 (26023)			
	77		26110		
			25000		
			20534		
			19646		

[a]Molar absorption coefficients are given in parenthesis

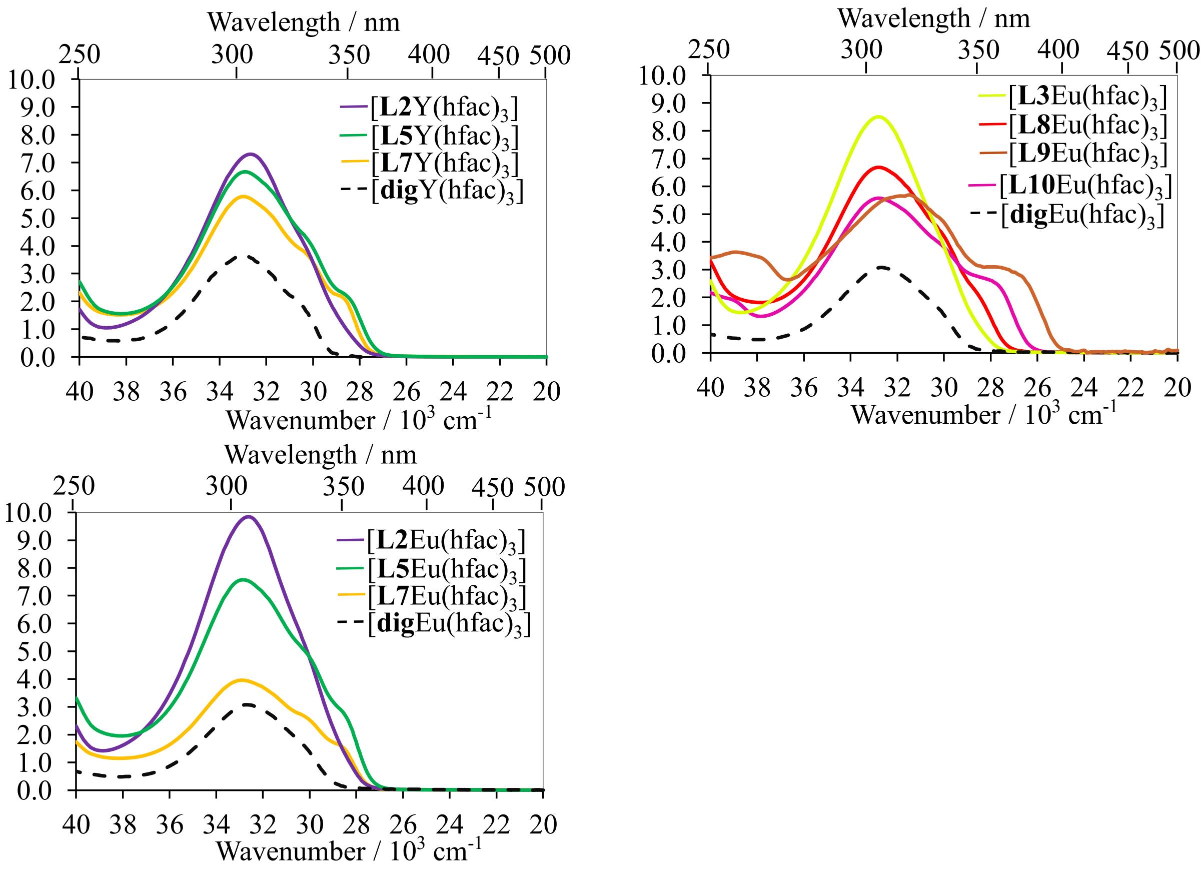

Figure A4-1. Absorption spectra of lanthanide complexes in CH_2Cl_2 at 293 K.

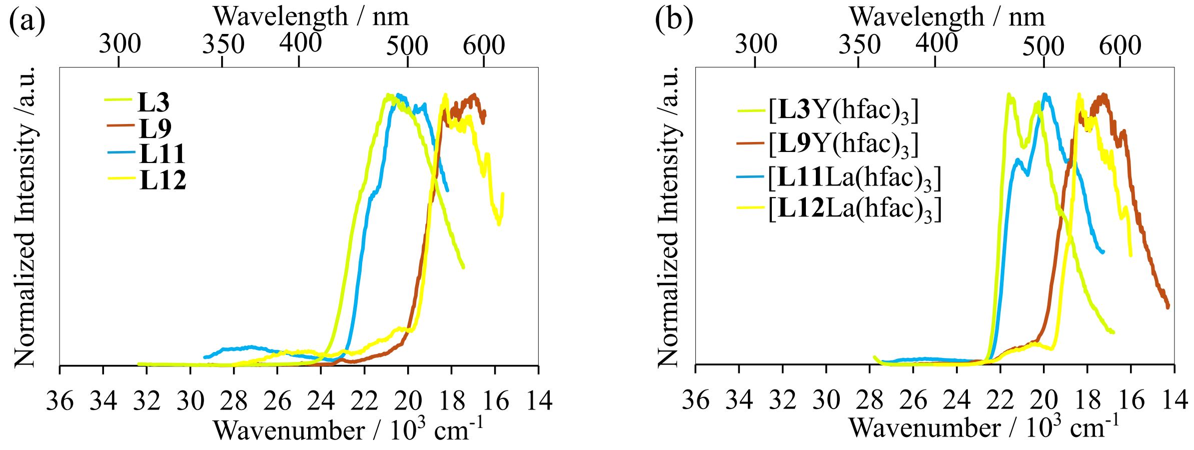

Figure A4-2. Normalized phosphorescence spectra in CH_2Cl_2 at 77 K of (a) **L12**,[28] **L11**,[28] **L9** (λ_{exc} = 310 nm), **L3** (λ_{exc} = 326 nm), and (b) their lanthanide complexes (λ_{exc} = 350 nm).

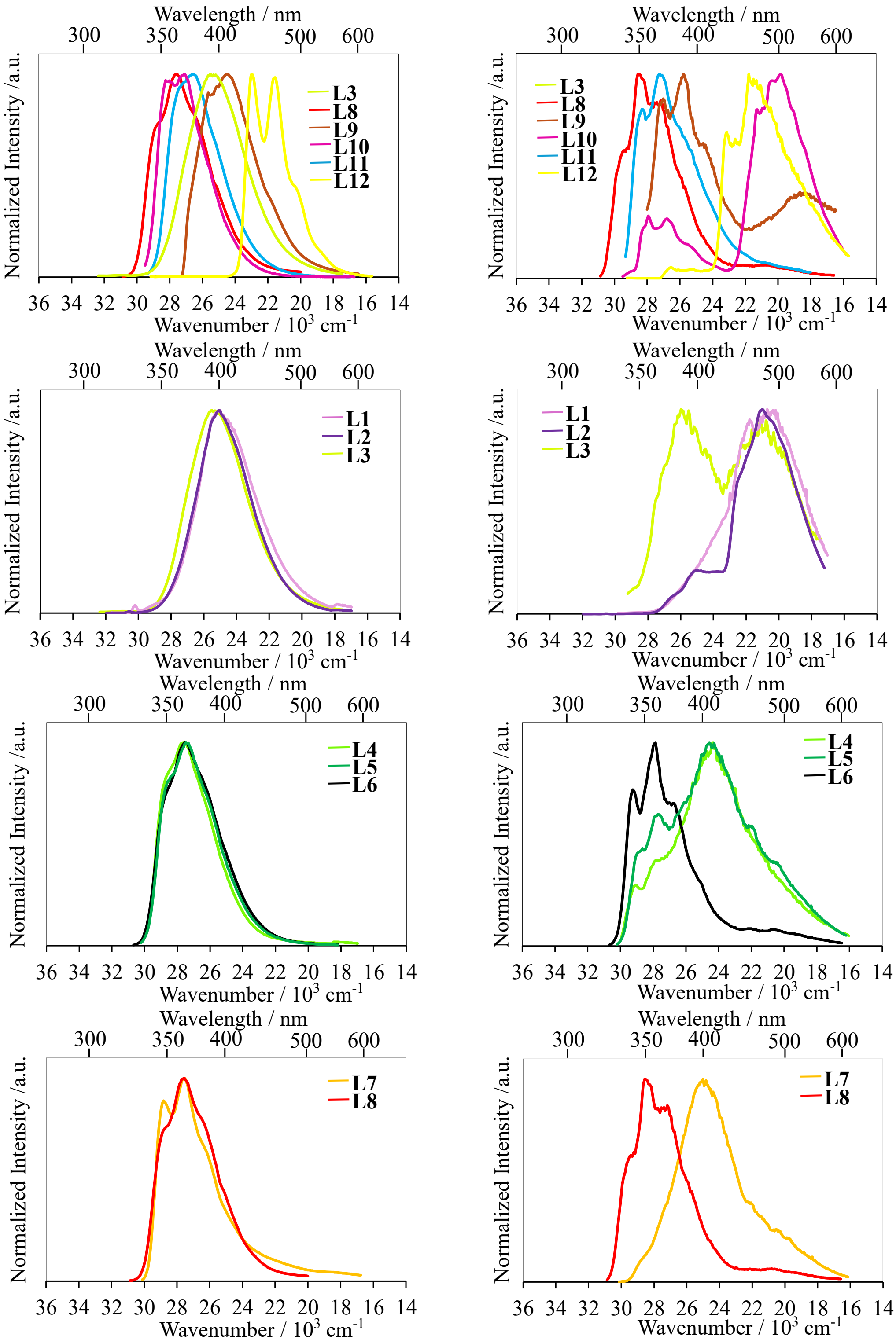

Figure A4-3. Normalized emission spectra in CH_2Cl_2 at 293 K (left) and 77 K (right) of **L11**,[28] **L12**,[28] **L2** (λ_{exc} = 327 nm), **L3** (λ_{exc} = 310 nm), **L4** (λ_{exc} = 326 nm), **L5** (λ_{exc} = 336 nm), **L6** (λ_{exc} = 327 nm), **L7** (λ_{exc} = 310 nm), **L8** (λ_{exc} = 326 nm), **L9** (λ_{exc} = 435 nm), and **L10** (λ_{exc} = 429 nm).

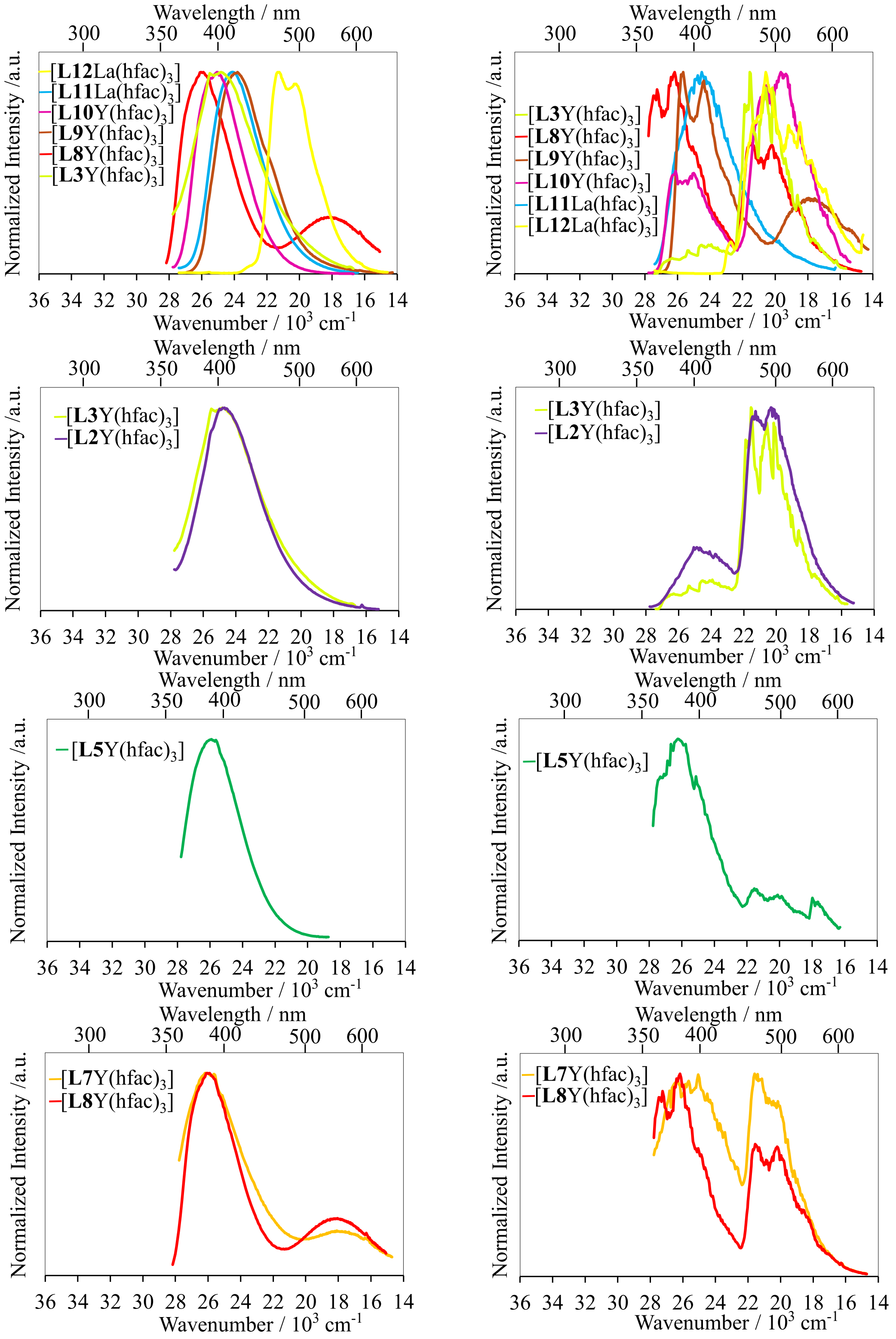

Figure A4-4. Normalized emission spectra (λ_{exc} = 350 nm) in CH_2Cl_2 at 293 K (left) and 77 K (right) of [**L12**La(hfac)$_3$],[28] [**L11**La(hfac)$_3$],[28] and [**L*k***Y(hfac)$_3$] (**L*k*** = **L2**, **L3**, **L5**, **L7**-**L10**) complexes.

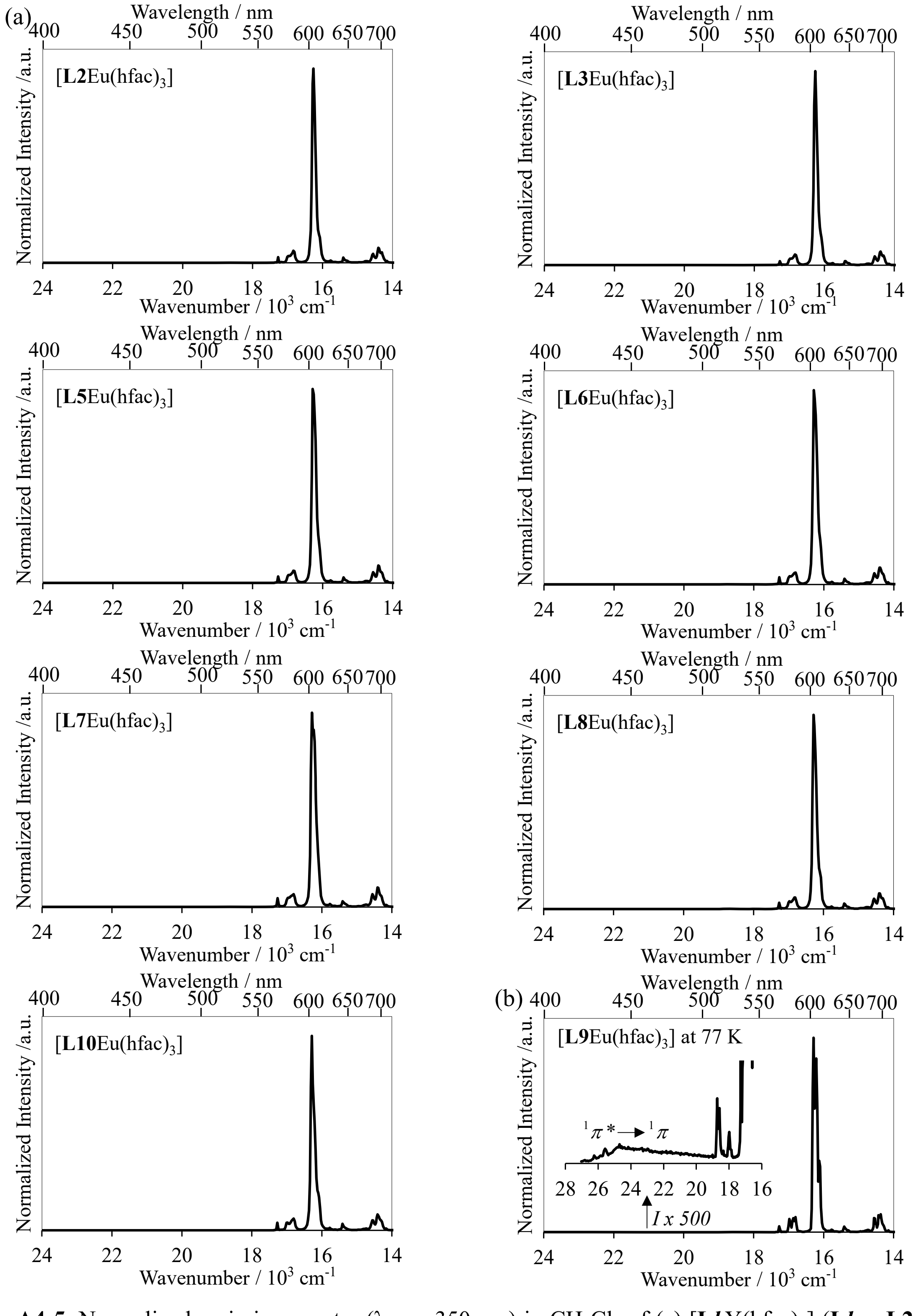

Figure A4-5. Normalized emission spectra (λ_{exc} = 350 nm) in CH_2Cl_2 of (a) [**L*k*** Y(hfac)$_3$] (**L*k*** = **L2**, **L3**, **L5**, **L7**, **L8**, **L10**) complexes at 293 K, and (b) [**L9**Eu(hfac)$_3$] at 77 K.

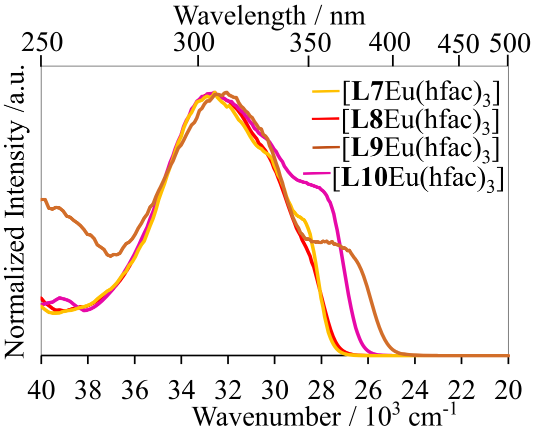

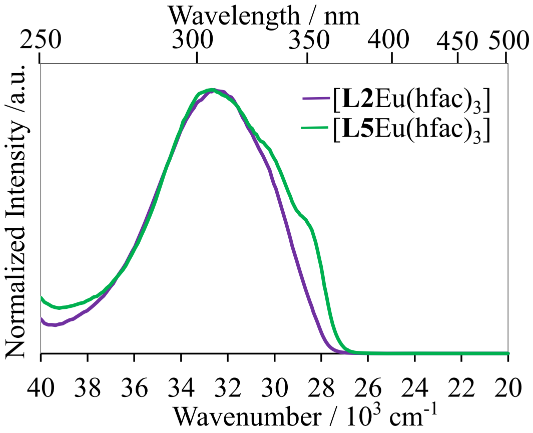

Figure A4-6. Normalized excitation spectra (λ_{exc} = 614 nm) in CH_2Cl_2 at 77 K of target europium complexes.
