# Supplementary material for: Catalytic and Photoluminescence Properties of the First‐ and Second‐Sphere Coordination of Lanthanide Complexes
Source: Chemistry. 2025 Oct 8;31(61):e02338. doi: 10.1002/chem.202502338 (PMC12587021; doi:10.1002/chem.202502338)

checkCIF/PLATON report

Structure factors have been supplied for datablock(s) 3_120K, 3_150K, 3_180K

THIS REPORT IS FOR GUIDANCE ONLY. IF USED AS PART OF A REVIEW PROCEDURE FOR PUBLICATION, IT SHOULD NOT REPLACE THE EXPERTISE OF AN EXPERIENCED CRYSTALLOGRAPHIC REFEREE.

No syntax errors found. CIF dictionary Interpreting this report

Datablock: 3_180K

```
Bond precision:    C-C = 0.0036 A           Wavelength=1.54184

Cell:              a=5.95539(13)    b=30.6792(6)       c=9.0684(2)
                   alpha=90         beta=102.590(2)    gamma=90
Temperature:       180 K

                  Calculated                  Reported
Volume            1617.02(6)                  1617.02(6)
Space group       P 21/n                      P 1 21/n 1
Hall group        -P 2yn                      -P 2yn
Moiety formula    C15 H19 N O6                C15 H19 N O6
Sum formula       C15 H19 N O6                C15 H19 N O6
Mr                309.31                      309.31
Dx,g cm-3         1.271                       1.271
Z                 4                           4
Mu (mm-1)         0.831                       0.831
F000              656.0                       656.0
F000'             658.30
h,k,lmax          7,37,11                     7,37,11
Nref              3065                        2985
Tmin,Tmax         0.885,0.960                 0.804,0.961
Tmin'             0.671

Correction method= # Reported T Limits: Tmin=0.804 Tmax=0.961
AbsCorr = ANALYTICAL

Data completeness= 0.974           Theta(max)= 70.013

                                                     wR2(reflections)=
R(reflections)= 0.0561( 2606)                        0.1617( 2985)

S = 1.064                 Npar= 201
```

The following ALERTS were generated. Each ALERT has the format
test-name_ALERT_alert-type_alert-level.
Click on the hyperlinks for more details of the test.

Alert level C

```
DIFMX02_ALERT_1_C  The maximum difference density is > 0.1*ZMAX*0.75
            The relevant atom site should be identified.
PLAT097_ALERT_2_C Large Reported Max.  (Positive) Residual Density       0.72 eA-3
PLAT220_ALERT_2_C NonSolvent   Resd 1  C   Ueq(max)/Ueq(min) Range        3.1 Ratio
PLAT360_ALERT_2_C Short  C(sp3)-C(sp3) Bond  C11      - C12      .       1.41 Ang.
PLAT906_ALERT_3_C Large K Value in the Analysis of Variance ......      2.653 Check
PLAT911_ALERT_3_C Missing FCF Refl Between Thmin & STh/L=    0.600         20 Report
                0 16  0,   2 20  0,  -4  8  1,   0 17  1,  -3 24  1,   2 16  2,
                1 21  2,  -3 33  2,  -3  7  5,   4 21  5,   0  0  6,   0  1  6,
               -5  4  6,   0 16  7,  -2 18  7,  -1 19  7,   1 26  7,   1 22  8,
                0 24  8,  -1 14  9,
```

Alert level G

```
PLAT793_ALERT_4_G Model has Chirality at C2          (Centro SpGr)          S Verify
PLAT912_ALERT_4_G Missing # of FCF Reflections Above STh/L=  0.600         60 Note
PLAT941_ALERT_3_G Average HKL Measurement Multiplicity ..........        2.3 Low
PLAT969_ALERT_5_G The 'Henn et al.' R-Factor-gap value ..........      7.740 Note
              Predicted wR2: Based on SigI**2  2.09 or SHELX Weight 15.19
PLAT978_ALERT_2_G Number C-C Bonds with Positive Residual Density.          6 Info
```

0 **ALERT level A** = Most likely a serious problem - resolve or explain
0 **ALERT level B** = A potentially serious problem, consider carefully
6 **ALERT level C** = Check. Ensure it is not caused by an omission or oversight
5 **ALERT level G** = General information/check it is not something unexpected

1 ALERT type 1 CIF construction/syntax error, inconsistent or missing data
4 ALERT type 2 Indicator that the structure model may be wrong or deficient
3 ALERT type 3 Indicator that the structure quality may be low
2 ALERT type 4 Improvement, methodology, query or suggestion
1 ALERT type 5 Informative message, check

Datablock: 3_150K

Bond precision: C-C = 0.0022 A   Wavelength=1.54184

Cell:	a=5.77540(15)	b=30.4471(7)	c=9.2827(2)
	alpha=90	beta=103.455(3)	gamma=90
Temperature:	150 K		

	Calculated	Reported
Volume	1587.51(7)	1587.51(7)
Space group	P 21/n	P 1 21/n 1
Hall group	-P 2yn	-P 2yn
Moiety formula	C15 H19 N O6	C15 H19 N O6
Sum formula	C15 H19 N O6	C15 H19 N O6
Mr	309.31	309.31
Dx,g cm-3	1.294	1.294
Z	4	4
Mu (mm-1)	0.846	0.846
F000	656.0	656.0
F000'	658.30	
h,k,lmax	7,37,11	7,36,11
Nref	3003	2839
Tmin,Tmax	0.883,0.941	0.790,0.943
Tmin'	0.657	

Correction method= # Reported T Limits: Tmin=0.790 Tmax=0.943
AbsCorr = ANALYTICAL

Data completeness= 0.945 Theta(max)= 70.059

R(reflections)= 0.0440(2474) wR2(reflections)= 0.1156(2839)

S = 1.049 Npar= 285

The following ALERTS were generated. Each ALERT has the format
test-name_ALERT_alert-type_alert-level.
Click on the hyperlinks for more details of the test.

Alert level C

```
PLAT029_ALERT_3_C _diffrn_measured_fraction_theta_full value Low .      0.961 Why?
PLAT088_ALERT_3_C Poor Data / Parameter Ratio ..................         9.96 Note
PLAT906_ALERT_3_C Large K Value in the Analysis of Variance ......      2.346 Check
PLAT911_ALERT_3_C Missing FCF Refl Between Thmin & STh/L=    0.600        111 Report
                 4  0  0,   4  1  0,   4  2  0,   4  3  0,   4  4  0,   3 12  0,
                 5 13  0,   3 29  0,   4  1  1,   6  1  1,   6  2  1,   6  3  1,
                -4  9  1,  -6 10  1,  -6 11  1,  -3 13  1,   1 16  1,   0 17  1,
                 2 18  1,  -4 19  1,   6  0  2,   6  1  2,   6  2  2,   6  3  2,
                 6  4  2,   3  7  2,   3  8  2,   6 11  2,   1 12  2,  -6 13  2,
                    (   81 More Missing: see the .ckf listing file)
```

Alert level G

```
PLAT230_ALERT_2_G Hirshfeld Test Diff for    O3B      --C10      .       15.5 s.u.
PLAT230_ALERT_2_G Hirshfeld Test Diff for    C2       --C3B      .        9.7 s.u.
PLAT301_ALERT_3_G Main Residue  Disorder .............(Resd    1)         41% Note
PLAT367_ALERT_2_G Long?  C(sp?)-C(sp?) Bond  C9       - C10      .       1.52 Ang.
```

```
PLAT793_ALERT_4_G Model has Chirality at C2         (Centro SpGr)          S Verify
PLAT811_ALERT_5_G No ADDSYM Analysis: Too Many Excluded Atoms ....          ! Info
PLAT912_ALERT_4_G Missing # of FCF Reflections Above STh/L=  0.600         53 Note
PLAT941_ALERT_3_G Average HKL Measurement Multiplicity ..........         2.3 Low
PLAT969_ALERT_5_G The 'Henn et al.' R-Factor-gap value ..........       4.583 Note
              Predicted wR2: Based on SigI**2  2.52 or SHELX Weight 11.02
PLAT978_ALERT_2_G Number C-C Bonds with Positive Residual Density.          4 Info
```

---

0 **ALERT level A** = Most likely a serious problem - resolve or explain
0 **ALERT level B** = A potentially serious problem, consider carefully
4 **ALERT level C** = Check. Ensure it is not caused by an omission or oversight
10 **ALERT level G** = General information/check it is not something unexpected

0 ALERT type 1 CIF construction/syntax error, inconsistent or missing data
4 ALERT type 2 Indicator that the structure model may be wrong or deficient
6 ALERT type 3 Indicator that the structure quality may be low
2 ALERT type 4 Improvement, methodology, query or suggestion
2 ALERT type 5 Informative message, check

Datablock: 3_120K

Bond precision: C-C = 0.0040 A Wavelength=1.54184

Cell:	a=11.5027(3)	b=30.3509(6)	c=9.6889(2)
	alpha=90	beta=111.568(3)	gamma=90
Temperature:	120 K		

	Calculated	Reported
Volume	3145.72(14)	3145.72(14)
Space group	P 21/c	P 1 21/c 1
Hall group	-P 2ybc	-P 2ybc
Moiety formula	C15 H19 N O6	C15 H19 N O6
Sum formula	C15 H19 N O6	C15 H19 N O6
Mr	309.31	309.31
Dx,g cm-3	1.306	1.306
Z	8	8
Mu (mm-1)	0.854	0.854
F000	1312.0	1312.0
F000'	1316.61	
h,k,lmax	14,37,11	13,36,11
Nref	5971	5575
Tmin,Tmax	0.873,0.951	0.786,0.957
Tmin'	0.653	

Correction method= # Reported T Limits: Tmin=0.786 Tmax=0.957
AbsCorr = ANALYTICAL

```
Data completeness= 0.934            Theta(max)= 70.045

                                                   wR2(reflections)=
R(reflections)= 0.0880( 4684)                      0.2426( 5575)

S = 1.029                 Npar= 401
```

```
The following ALERTS were generated. Each ALERT has the format
       test-name_ALERT_alert-type_alert-level.
Click on the hyperlinks for more details of the test.
```

Alert level B

```
PLAT029_ALERT_3_B _diffrn_measured_fraction_theta_full value Low .      0.951 Why?
```

Author Response: Compound 3 undergoes a phase transition below 150K with a doubling of the lattice volume accompanied by a slight deterioration of the crystal. Data collection's strategie was calculated for the small cell at 180K, that may explain the low completness of reflexions file.

Alert level C

```
DIFMX02_ALERT_1_C  The maximum difference density is > 0.1*ZMAX*0.75
            The relevant atom site should be identified.
PLAT094_ALERT_2_C Ratio of Maximum / Minimum Residual Density ....       2.36 Report
PLAT097_ALERT_2_C Large Reported Max.  (Positive) Residual Density       0.76 eA-3
PLAT906_ALERT_3_C Large K Value in the Analysis of Variance ......      3.181 Check
PLAT911_ALERT_3_C Missing FCF Refl Between Thmin & STh/L=    0.600        277 Report
                1   0   0,  11   0   0,  12   0   0,  11   1   0,  12   1   0,  11   2   0,
               12   2   0,   1   3   0,  11   3   0,  12   3   0,   9  13   0,   1  17   0,
                9  21   0,   7  23   0,   6  24   0,   7  24   0,   8  24   0,   6  25   0,
                7  25   0,   7  26   0,   6  28   0,   7   1   1,  11   1   1,   7   2   1,
               11   2   1,   7   3   1,  11   3   1,  11   4   1,   1   5   1,  11   5   1,
                    (  247 More Missing: see the .ckf listing file)
```

Alert level G

```
PLAT072_ALERT_2_G SHELXL First  Parameter in WGHT  Unusually Large       0.18 Report
PLAT720_ALERT_4_G Number of Unusual/Non-Standard Labels ..........          2 Note
              H1CA    H1CB
PLAT793_ALERT_4_G Model has Chirality at C2          (Centro SpGr)          S Verify
PLAT793_ALERT_4_G Model has Chirality at C2C         (Centro SpGr)          R Verify
PLAT912_ALERT_4_G Missing # of FCF Reflections Above STh/L=  0.600        116 Note
PLAT941_ALERT_3_G Average HKL Measurement Multiplicity ..........         2.3 Low
PLAT969_ALERT_5_G The 'Henn et al.' R-Factor-gap value ..........       6.323 Note
              Predicted wR2: Based on SigI**2  3.84 or SHELX Weight 23.57
PLAT978_ALERT_2_G Number C-C Bonds with Positive Residual Density.          0 Info
```

---

0 **ALERT level A** = Most likely a serious problem - resolve or explain
1 **ALERT level B** = A potentially serious problem, consider carefully
5 **ALERT level C** = Check. Ensure it is not caused by an omission or oversight

```
  8 ALERT level G = General information/check it is not something unexpected

  1 ALERT type 1 CIF construction/syntax error, inconsistent or missing data
  4 ALERT type 2 Indicator that the structure model may be wrong or deficient
  4 ALERT type 3 Indicator that the structure quality may be low
  4 ALERT type 4 Improvement, methodology, query or suggestion
  1 ALERT type 5 Informative message, check
```

It is advisable to attempt to resolve as many as possible of the alerts in all categories. Often the minor alerts point to easily fixed oversights, errors and omissions in your CIF or refinement strategy, so attention to these fine details can be worthwhile. In order to resolve some of the more serious problems it may be necessary to carry out additional measurements or structure refinements. However, the purpose of your study may justify the reported deviations and the more serious of these should normally be commented upon in the discussion or experimental section of a paper or in the "special_details" fields of the CIF. checkCIF was carefully designed to identify outliers and unusual parameters, but every test has its limitations and alerts that are not important in a particular case may appear. Conversely, the absence of alerts does not guarantee there are no aspects of the results needing attention. It is up to the individual to critically assess their own results and, if necessary, seek expert advice.

Publication of your CIF in IUCr journals

A basic structural check has been run on your CIF. These basic checks will be run on all CIFs submitted for publication in IUCr journals (*Acta Crystallographica*, *Journal of Applied Crystallography*, *Journal of Synchrotron Radiation*); however, if you intend to submit to *Acta Crystallographica Section C* or *E* or *IUCrData*, you should make sure that full publication checks are run on the final version of your CIF prior to submission.

Publication of your CIF in other journals

Please refer to the *Notes for Authors* of the relevant journal for any special instructions relating to CIF submission.

PLATON version of 04/06/2025; check.def file version of 30/05/2025

Datablock 3_180K - ellipsoid plot

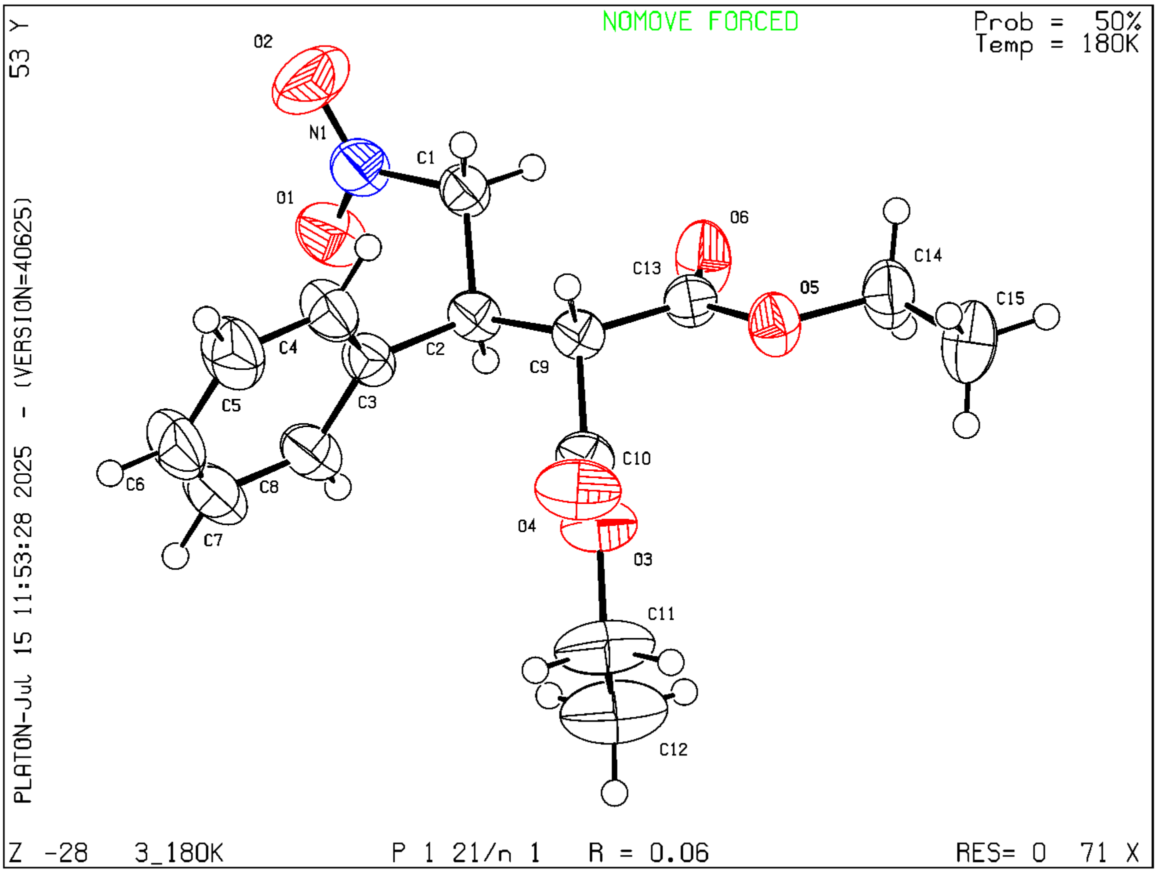

Datablock 3_150K - ellipsoid plot

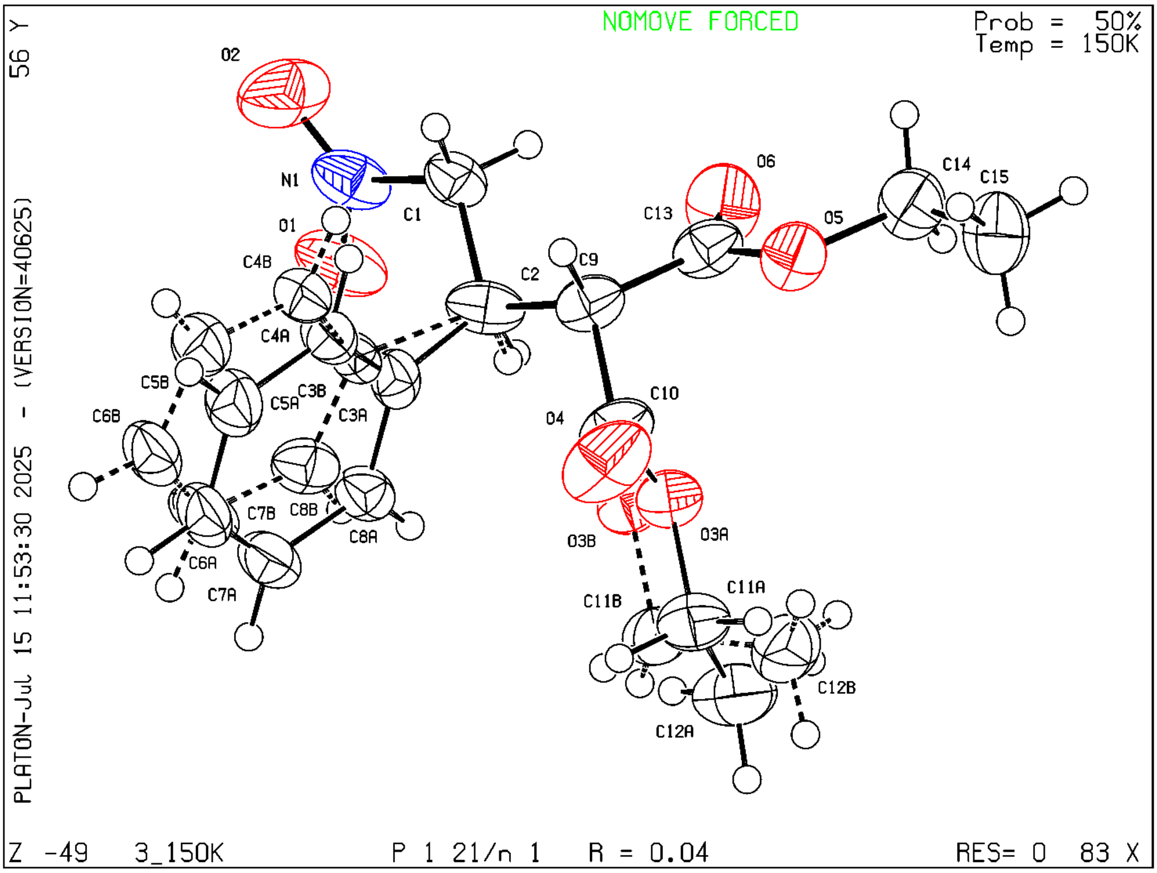

Datablock 3_120K - ellipsoid plot

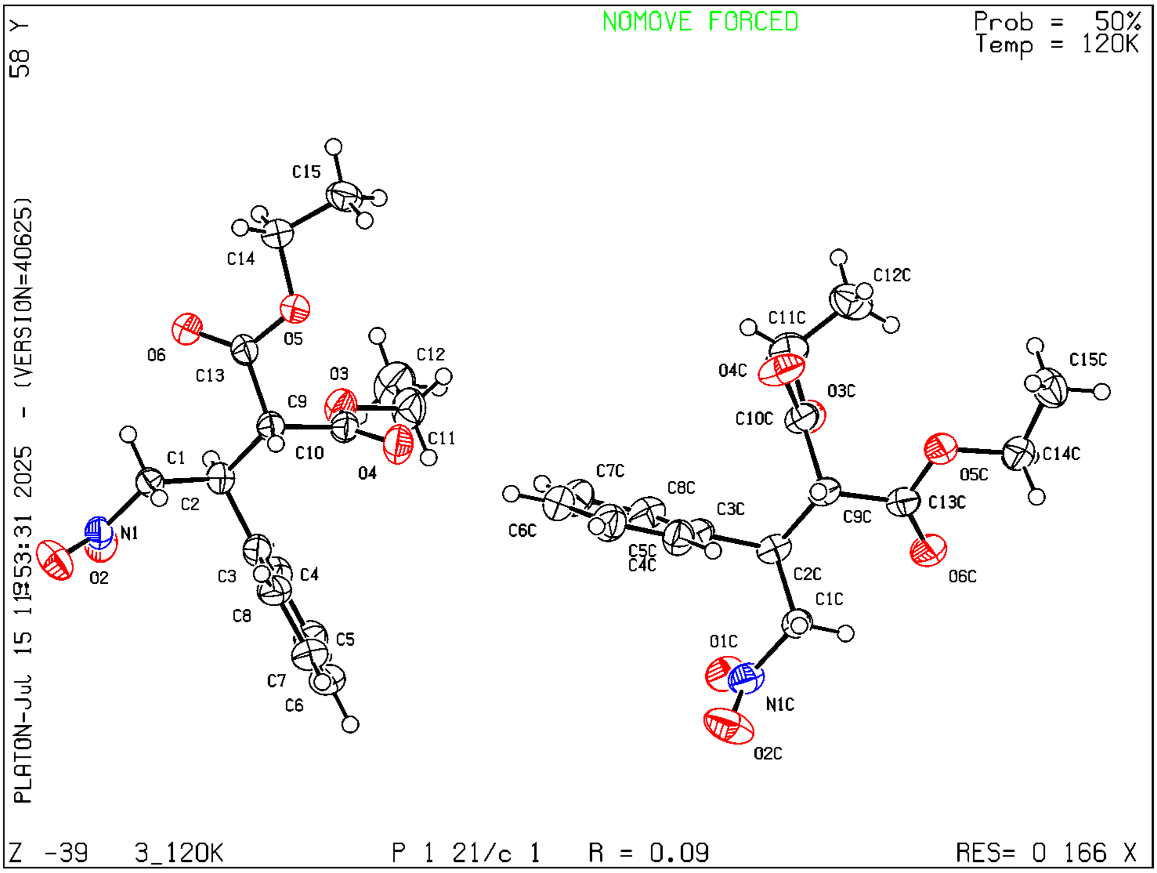

Supplement: Supplementary file 2 — Supporting Information [file CHEM-31-e02338-s002.zip › checkcif_compound3_temp.pdf]
