# Supplementary material for: Catalytic and Photoluminescence Properties of the First‐ and Second‐Sphere Coordination of Lanthanide Complexes
Source: Chemistry. 2025 Oct 8;31(61):e02338. doi: 10.1002/chem.202502338 (PMC12587021; doi:10.1002/chem.202502338)

checkCIF/PLATON report

Structure factors have been supplied for datablock(s) L1, L2, L3, L4, L5

THIS REPORT IS FOR GUIDANCE ONLY. IF USED AS PART OF A REVIEW PROCEDURE FOR PUBLICATION, IT SHOULD NOT REPLACE THE EXPERTISE OF AN EXPERIENCED CRYSTALLOGRAPHIC REFEREE.

No syntax errors found. CIF dictionary Interpreting this report

Datablock: L1

```
Bond precision:   C-C = 0.0031 A          Wavelength=1.54184

Cell:            a=10.56341(18)    b=21.4719(3)        c=9.62625(11)
                 alpha=90          beta=102.8037(14)    gamma=90
Temperature:     120 K

                  Calculated                     Reported
Volume            2129.10(5)                     2129.10(6)
Space group       P 21/c                         P 1 21/c 1
Hall group        -P 2ybc                        -P 2ybc
Moiety formula    C21 H18 N6, C H2 Cl2           C21 H18 N6, C H2 Cl2
Sum formula       C22 H20 Cl2 N6                 C22 H20 Cl2 N6
Mr                439.34                         439.34
Dx,g cm-3         1.371                          1.371
Z                 4                              4
Mu (mm-1)         2.914                          2.914
F000              912.0                          912.0
F000'             917.08
h,k,lmax          13,26,12                       13,26,11
Nref              4425                           4307
Tmin,Tmax         0.900,0.971                    0.640,0.958
Tmin'             0.394

Correction method= # Reported T Limits: Tmin=0.640 Tmax=0.958
AbsCorr = ANALYTICAL

Data completeness= 0.973           Theta(max)= 75.662

                                                          wR2(reflections)=
R(reflections)= 0.0541( 3843)                             0.1382( 4307)

S = 1.081                    Npar= 279
```

The following ALERTS were generated. Each ALERT has the format
test-name_ALERT_alert-type_alert-level.
Click on the hyperlinks for more details of the test.

Alert level C

```
PLAT244_ALERT_4_C Low    'Solvent' Ueq as Compared to Neighbors of        C22 Check
PLAT906_ALERT_3_C Large K Value in the Analysis of Variance ......      3.798 Check
PLAT934_ALERT_3_C Number of (Iobs-Icalc)/Sigma(W) > 10 Outliers ..          1 Check
               -4  3 11,
```

Alert level G

```
PLAT432_ALERT_2_G Short Inter X...Y Contact  Cl2      ..C8       .       3.25 Ang.
                                                  1-x,1-y,-z  =      3_665 Check
PLAT480_ALERT_4_G Long H...A H-Bond Reported H9       ..N3       .       2.63 Ang.
PLAT912_ALERT_4_G Missing # of FCF Reflections Above STh/L=  0.600        107 Note
PLAT933_ALERT_2_G Number of HKL-OMIT Records in Embedded .res File          1 Note
               -5  2 11,
PLAT969_ALERT_5_G The 'Henn et al.' R-Factor-gap value ..........      4.919 Note
              Predicted wR2: Based on SigI**2  2.81 or SHELX Weight 12.79
PLAT978_ALERT_2_G Number C-C Bonds with Positive Residual Density.          7 Info
```

0 **ALERT level A** = Most likely a serious problem - resolve or explain
0 **ALERT level B** = A potentially serious problem, consider carefully
3 **ALERT level C** = Check. Ensure it is not caused by an omission or oversight
6 **ALERT level G** = General information/check it is not something unexpected

0 ALERT type 1 CIF construction/syntax error, inconsistent or missing data
3 ALERT type 2 Indicator that the structure model may be wrong or deficient
2 ALERT type 3 Indicator that the structure quality may be low
3 ALERT type 4 Improvement, methodology, query or suggestion
1 ALERT type 5 Informative message, check

Datablock: L2

Bond precision:	C-C = 0.0163 A	Wavelength=1.54184	
Cell:	a=9.59092(17)	b=36.6694(6)	c=14.4428(4)
	alpha=90	beta=105.614(2)	gamma=90
Temperature:	120 K		

	Calculated	Reported
Volume	4891.99(18)	4891.99(18)
Space group	P 21	P 1 21 1
Hall group	P 2yb	P 2yb
Moiety formula	2(C26 H28 N6), C H2 Cl2	0.5(C H2 Cl2), C26 H28 N6
Sum formula	C53 H58 Cl2 N12	C26.50 H29 Cl N6
Mr	934.01	467.01
Dx,g cm-3	1.268	1.268
Z	4	8
Mu (mm-1)	1.583	1.583
F000	1976.0	1976.0
F000'	1983.82	
h,k,lmax	12,46,18	11,45,17
Nref	20371[10351]	17794
Tmin,Tmax	0.945,0.969	0.764,0.973
Tmin'	0.566	

Correction method= # Reported T Limits: Tmin=0.764 Tmax=0.973
AbsCorr = ANALYTICAL

Data completeness= 1.72/0.87 Theta(max)= 75.966

R(reflections)= 0.0903(10609) wR2(reflections)= 0.2856(17794)

S = 1.124 Npar= 1224

The following ALERTS were generated. Each ALERT has the format
test-name_ALERT_alert-type_alert-level.
Click on the hyperlinks for more details of the test.

Alert level B

```
PLAT340_ALERT_3_B Low Bond Precision on  C-C Bonds ...............    0.01633 Ang.
```

Alert level C

```
RINTA01_ALERT_3_C  The value of Rint is greater than 0.12
            Rint given   0.120
PLAT041_ALERT_1_C Calc. and Reported SumFormula    Strings  Differ     Please Check
              Calc: C53 H58 Cl2 N12
              Rep.: C26.50 H29 Cl N6
PLAT042_ALERT_1_C Calc. and Reported MoietyFormula Strings  Differ     Please Check
              Calc: 2(C26 H28 N6), C H2 Cl2
              Rep.: 0.5(C H2 Cl2), C26 H28 N6
PLAT084_ALERT_3_C High wR2 Value (i.e. > 0.25) ...................       0.29 Report
PLAT244_ALERT_4_C Low    'Solvent' Ueq as Compared to Neighbors of        C1S Check
PLAT244_ALERT_4_C Low    'Solvent' Ueq as Compared to Neighbors of        C2S Check
PLAT360_ALERT_2_C Short  C(sp3)-C(sp3) Bond  C9       - C10      .       1.43 Ang.
PLAT412_ALERT_2_C Short Intra XH3 .. XHn     H16C     ..H19B     .       1.85 Ang.
```

```
                                                   x,y,z  =      1_555 Check
PLAT906_ALERT_3_C Large K Value in the Analysis of Variance ......      2.343 Check
PLAT911_ALERT_3_C Missing FCF Refl Between Thmin & STh/L=    0.600         80 Report
                0  8  0,   0 10  0,   4 34  0,   0  1  1,   0  3  1,   0  4  1,
               -6 29  1,  -4 34  1,   0  0  2,   0  4  2,   0  5  2,   6 26  2,
                6 27  2,   0  0  3,   5  5  3,   5 23  3,   4  0  4,   6 22  4,
                6 23  4,   6 24  4,   6 25  4,   8  0  5,   8  1  5,   8  2  5,
                8  3  5,   5 24  5,   5 25  5,   5 26  5,   5 27  5, -11  0  7,
                   (   50 More Missing: see the .ckf listing file)
PLAT915_ALERT_3_C No Flack x Check Done: Low Friedel Pair Coverage         79 %
```

Alert level G

```
PLAT007_ALERT_5_G Number of Unrefined Donor-H Atoms .............          4 Report
              H5    H5B   H5C   H5D
PLAT045_ALERT_1_G Calculated and Reported Z Differ by a Factor ...      0.500 Check
PLAT072_ALERT_2_G SHELXL First  Parameter in WGHT  Unusually Large       0.16 Report
PLAT111_ALERT_2_G ADDSYM Detects New (Pseudo) Centre of Symmetry .         97 %Fit
PLAT112_ALERT_2_G ADDSYM Detects New (Pseudo) Symm. Elem         n         91 %Fit
PLAT113_ALERT_2_G ADDSYM Suggests Possible Pseudo/New  Space Group      P21/n Check
              Check Model Parameter Symmetry for Reflection Data Support
PLAT432_ALERT_2_G Short Inter X...Y Contact  C17      ..C18B     .       3.18 Ang.
                                                -1+x,y,z  =      1_455 Check
PLAT432_ALERT_2_G Short Inter X...Y Contact  C17C     ..C18D     .       3.16 Ang.
                                                -1+x,y,z  =      1_455 Check
PLAT720_ALERT_4_G Number of Unusual/Non-Standard Labels ..........         13 Note
              H1SA    H1SB    H2SA    H2SB    H5CA    H8CA    H8CB    H5BA
              H8BA    H8BB    H5DA    H8DA    H8DB
PLAT790_ALERT_4_G Centre of Gravity not Within Unit Cell: Resd.  #          4 Note
              C26 H28 N6
PLAT790_ALERT_4_G Centre of Gravity not Within Unit Cell: Resd.  #          6 Note
              C H2 Cl2
PLAT791_ALERT_4_G Model has Chirality at C9         (Sohncke SpGr)          S Verify
PLAT791_ALERT_4_G Model has Chirality at C9B        (Sohncke SpGr)          S Verify
PLAT791_ALERT_4_G Model has Chirality at C9C        (Sohncke SpGr)          S Verify
PLAT791_ALERT_4_G Model has Chirality at C9D        (Sohncke SpGr)          S Verify
PLAT910_ALERT_3_G Missing FCF Reflection(s) Below Theta(Min)[Deg]=       3.18 Note
                0  2  0,
PLAT912_ALERT_4_G Missing # of FCF Reflections Above STh/L=  0.600        331 Note
PLAT933_ALERT_2_G Number of HKL-OMIT Records in Embedded .res File          3 Note
               -5-13 12,  -5-11 13,  -5-10 12,
PLAT941_ALERT_3_G Average HKL Measurement Multiplicity ..........         4.4 Low
PLAT969_ALERT_5_G The 'Henn et al.' R-Factor-gap value ..........       3.267 Note
              Predicted wR2: Based on SigI**2  8.74 or SHELX Weight 25.41
PLAT978_ALERT_2_G Number C-C Bonds with Positive Residual Density.          3 Info
```

 0 **ALERT level A** = Most likely a serious problem - resolve or explain
 1 **ALERT level B** = A potentially serious problem, consider carefully
11 **ALERT level C** = Check. Ensure it is not caused by an omission or oversight
21 **ALERT level G** = General information/check it is not something unexpected

 3 ALERT type 1 CIF construction/syntax error, inconsistent or missing data
10 ALERT type 2 Indicator that the structure model may be wrong or deficient
 8 ALERT type 3 Indicator that the structure quality may be low
10 ALERT type 4 Improvement, methodology, query or suggestion
 2 ALERT type 5 Informative message, check

Datablock: L3

Bond precision:	C-C = 0.0025 A	Wavelength=1.54184	
Cell:	a=11.76783(10)	b=14.68225(10)	c=15.97162(14)
	alpha=90	beta=90	gamma=90
Temperature:	120 K		

	Calculated	Reported
Volume	2759.55(4)	2759.55(4)
Space group	P 21 21 21	P 21 21 21
Hall group	P 2ac 2ab	P 2ac 2ab
Moiety formula	C31 H38 N6	C31 H38 N6
Sum formula	C31 H38 N6	C31 H38 N6
Mr	494.67	494.67
Dx,g cm-3	1.191	1.191
Z	4	4
Mu (mm-1)	0.558	0.558
F000	1064.0	1064.0
F000'	1066.76	
h,k,lmax	14,18,20	14,18,19
Nref	5750[3231]	5666
Tmin,Tmax	0.872,0.915	0.918,0.942
Tmin'	0.854	

Correction method= # Reported T Limits: Tmin=0.918 Tmax=0.942
AbsCorr = ANALYTICAL

Data completeness= 1.75/0.99 Theta(max)= 75.917

R(reflections)= 0.0317(5430) wR2(reflections)= 0.0826(5666)

S = 1.039 Npar= 341

The following ALERTS were generated. Each ALERT has the format
test-name_ALERT_alert-type_alert-level.
Click on the hyperlinks for more details of the test.

Alert level G

PLAT032_ALERT_4_G Std. Uncertainty on Flack Parameter Value High . 0.300 Report
PLAT142_ALERT_4_G s.u. on b - Axis Small or Missing .... 0.00010 Ang.
PLAT143_ALERT_4_G s.u. on c - Axis Small or Missing .... 0.00014 Ang.
PLAT791_ALERT_4_G Model has Chirality at C21 (Sohncke SpGr) S Verify

```
PLAT791_ALERT_4_G Model has Chirality at C26        (Sohncke SpGr)          S Verify
PLAT912_ALERT_4_G Missing # of FCF Reflections Above STh/L=  0.600         24 Note
PLAT969_ALERT_5_G The 'Henn et al.' R-Factor-gap value ..........      3.733 Note
              Predicted wR2: Based on SigI**2  2.21 or SHELX Weight  7.95
PLAT978_ALERT_2_G Number C-C Bonds with Positive Residual Density.          1 Info
```

```
   0 ALERT level A = Most likely a serious problem - resolve or explain
   0 ALERT level B = A potentially serious problem, consider carefully
   0 ALERT level C = Check. Ensure it is not caused by an omission or oversight
   8 ALERT level G = General information/check it is not something unexpected

   0 ALERT type 1 CIF construction/syntax error, inconsistent or missing data
   1 ALERT type 2 Indicator that the structure model may be wrong or deficient
   0 ALERT type 3 Indicator that the structure quality may be low
   6 ALERT type 4 Improvement, methodology, query or suggestion
   1 ALERT type 5 Informative message, check
```

Datablock: L4

Bond precision: C-C = 0.0018 A Wavelength=1.54184

Cell:	a=11.90101(10)	b=13.07144(10)	c=17.20864(13)
	alpha=90	beta=109.2762(9)	gamma=90
Temperature:	100 K		

	Calculated	Reported
Volume	2526.95(4)	2526.95(4)
Space group	P 21/c	P 1 21/c 1
Hall group	-P 2ybc	-P 2ybc
Moiety formula	C25 H26 N6 O, C H4 O, 2(H2 O)	C25 H26 N6 O, C H4 O, 2(H2 O)
Sum formula	C26 H34 N6 O4	C26 H34 N6 O4
Mr	494.59	494.59
Dx,g cm-3	1.300	1.300
Z	4	4
Mu (mm-1)	0.730	0.730
F000	1056.0	1056.0
F000'	1059.19	
h,k,lmax	14,16,21	14,16,21
Nref	5139	5071
Tmin,Tmax	0.827,0.909	0.865,0.919
Tmin'	0.827	

Correction method= # Reported T Limits: Tmin=0.865 Tmax=0.919
AbsCorr = ANALYTICAL

```
Data completeness= 0.987          Theta(max)= 74.272

R(reflections)= 0.0365( 4597)     wR2(reflections)= 0.0958( 5071)

S = 1.041                  Npar= 349
```

```
The following ALERTS were generated. Each ALERT has the format
       test-name_ALERT_alert-type_alert-level.
Click on the hyperlinks for more details of the test.
```

Alert level C

```
PLAT911_ALERT_3_C Missing FCF Refl Between Thmin & STh/L=    0.600          3 Report
               -4 14  8,  -5 13 10,  -4 13 11,
```

Alert level G

```
PLAT142_ALERT_4_G s.u. on b - Axis Small or Missing .............    0.00010 Ang.
PLAT143_ALERT_4_G s.u. on c - Axis Small or Missing .............    0.00013 Ang.
PLAT912_ALERT_4_G Missing # of FCF Reflections Above STh/L=  0.600         64 Note
PLAT933_ALERT_2_G Number of HKL-OMIT Records in Embedded .res File          2 Note
               -3 14 11,  -1 14 10,
PLAT941_ALERT_3_G Average HKL Measurement Multiplicity ..........         4.3 Low
PLAT969_ALERT_5_G The 'Henn et al.' R-Factor-gap value ..........       5.969 Note
              Predicted wR2: Based on SigI**2  1.61 or SHELX Weight  9.20
PLAT978_ALERT_2_G Number C-C Bonds with Positive Residual Density.         17 Info
```

```
   0 ALERT level A = Most likely a serious problem - resolve or explain
   0 ALERT level B = A potentially serious problem, consider carefully
   1 ALERT level C = Check. Ensure it is not caused by an omission or oversight
   7 ALERT level G = General information/check it is not something unexpected

   0 ALERT type 1 CIF construction/syntax error, inconsistent or missing data
   2 ALERT type 2 Indicator that the structure model may be wrong or deficient
   2 ALERT type 3 Indicator that the structure quality may be low
   3 ALERT type 4 Improvement, methodology, query or suggestion
   1 ALERT type 5 Informative message, check
```

Datablock: L5

```
Bond precision:   C-C = 0.0060 A           Wavelength=1.54184

Cell:             a=14.15207(8)     b=20.97488(13)      c=9.21700(5)
                  alpha=90          beta=97.2245(5)     gamma=90
Temperature:      100 K
```

	Calculated	Reported
Volume	2714.24(3)	2714.23(3)
Space group	P 21	P 1 21 1
Hall group	P 2yb	P 2yb
Moiety formula	C30 H36 N6 O	C30 H36 N6 O
Sum formula	C30 H36 N6 O	C30 H36 N6 O
Mr	496.65	496.65
Dx,g cm-3	1.215	1.215
Z	4	4
Mu (mm-1)	0.599	0.599
F000	1064.0	1064.0
F000'	1066.88	
h,k,lmax	17,26,11	17,25,11
Nref	11344[5832]	10977
Tmin,Tmax	0.917,0.948	0.917,0.952
Tmin'	0.903	

Correction method= # Reported T Limits: Tmin=0.917 Tmax=0.952
AbsCorr = ANALYTICAL

Data completeness= 1.88/0.97 Theta(max)= 76.079

R(reflections)= 0.0504(10395) wR2(reflections)= 0.1454(10977)

S = 1.041 Npar= 675

The following ALERTS were generated. Each ALERT has the format
test-name_ALERT_alert-type_alert-level.
Click on the hyperlinks for more details of the test.

Alert level B

PLAT201_ALERT_2_B Isotropic non-H Atoms in Main Residue(s) .... 4 Report
C51A C52A C53A C54A

Author Response: An alkyl chain is disordered and was refined with two components with refined aoocupancies 0.73/0.27 and with isotropic ADPs.

Alert level C

PLAT230_ALERT_2_C Hirshfeld Test Diff for C21 --C22 . 5.2 s.u.
PLAT230_ALERT_2_C Hirshfeld Test Diff for C21 --C23 . 5.7 s.u.
PLAT340_ALERT_3_C Low Bond Precision on C-C Bonds .... 0.00602 Ang.

Alert level G

```
PLAT002_ALERT_2_G Number of Distance or Angle Restraints on AtSite          9 Note
PLAT007_ALERT_5_G Number of Unrefined Donor-H Atoms .............           2 Report
              H7    H1
PLAT032_ALERT_4_G Std. Uncertainty on Flack Parameter Value High .      0.400 Report
PLAT066_ALERT_1_G Predicted and Reported Tmin&Tmax Range Identical          ? Check
PLAT111_ALERT_2_G ADDSYM Detects New (Pseudo) Centre of Symmetry .         94 %Fit
PLAT112_ALERT_2_G ADDSYM Detects New (Pseudo) Symm. Elem        c          94 %Fit
PLAT113_ALERT_2_G ADDSYM Suggests Possible Pseudo/New  Space Group      P21/c Check
              WARNING: Disordered Atoms Excluded from Analysis
              Check Model Parameter Symmetry for Reflection Data Support
PLAT142_ALERT_4_G s.u. on b - Axis Small or Missing .............     0.00013 Ang.
PLAT143_ALERT_4_G s.u. on c - Axis Small or Missing .............     0.00005 Ang.
PLAT172_ALERT_4_G The CIF-Embedded .res File Contains DFIX Records          4 Report
PLAT301_ALERT_3_G Main Residue  Disorder .............(Resd    1)         11% Note
PLAT773_ALERT_2_G Check long C-C Bond in CIF: C50      --C51B            1.73 Ang.
PLAT791_ALERT_4_G Model has Chirality at C21        (Sohncke SpGr)          S Verify
PLAT791_ALERT_4_G Model has Chirality at C51A       (Sohncke SpGr)          S Verify
PLAT860_ALERT_3_G Number of Least-Squares Restraints ............           7 Note
PLAT912_ALERT_4_G Missing # of FCF Reflections Above STh/L=  0.600         60 Note
PLAT969_ALERT_5_G The 'Henn et al.' R-Factor-gap value ..........      10.221 Note
              Predicted wR2: Based on SigI**2  1.42 or SHELX Weight 13.97
PLAT978_ALERT_2_G Number C-C Bonds with Positive Residual Density.          2 Info
PLAT992_ALERT_5_G Repd & Actual _reflns_number_gt Values Differ by          3 Check
```

```
   0 ALERT level A = Most likely a serious problem - resolve or explain
   1 ALERT level B = A potentially serious problem, consider carefully
   3 ALERT level C = Check. Ensure it is not caused by an omission or oversight
  19 ALERT level G = General information/check it is not something unexpected

   1 ALERT type 1 CIF construction/syntax error, inconsistent or missing data
   9 ALERT type 2 Indicator that the structure model may be wrong or deficient
   3 ALERT type 3 Indicator that the structure quality may be low
   7 ALERT type 4 Improvement, methodology, query or suggestion
   3 ALERT type 5 Informative message, check
```

It is advisable to attempt to resolve as many as possible of the alerts in all categories. Often the minor alerts point to easily fixed oversights, errors and omissions in your CIF or refinement strategy, so attention to these fine details can be worthwhile. In order to resolve some of the more serious problems it may be necessary to carry out additional measurements or structure refinements. However, the purpose of your study may justify the reported deviations and the more serious of these should normally be commented upon in the discussion or experimental section of a paper or in the "special_details" fields of the CIF. checkCIF was carefully designed to identify outliers and unusual parameters, but every test has its limitations and alerts that are not important in a particular case may appear. Conversely, the absence of alerts does not guarantee there are no aspects of the results needing attention. It is up to the individual to critically assess their own results and, if necessary, seek expert advice.

Publication of your CIF in IUCr journals

A basic structural check has been run on your CIF. These basic checks will be run on all CIFs submitted for publication in IUCr journals (*Acta Crystallographica*, *Journal of Applied Crystallography*, *Journal of Synchrotron Radiation*); however, if you intend to submit to *Acta Crystallographica Section C* or *E* or *IUCrData*, you should make sure that full publication checks are run on the final version of your CIF prior to submission.

Publication of your CIF in other journals

Please refer to the *Notes for Authors* of the relevant journal for any special instructions relating to CIF submission.

PLATON version of 04/06/2025; check.def file version of 30/05/2025

Datablock L1 - ellipsoid plot

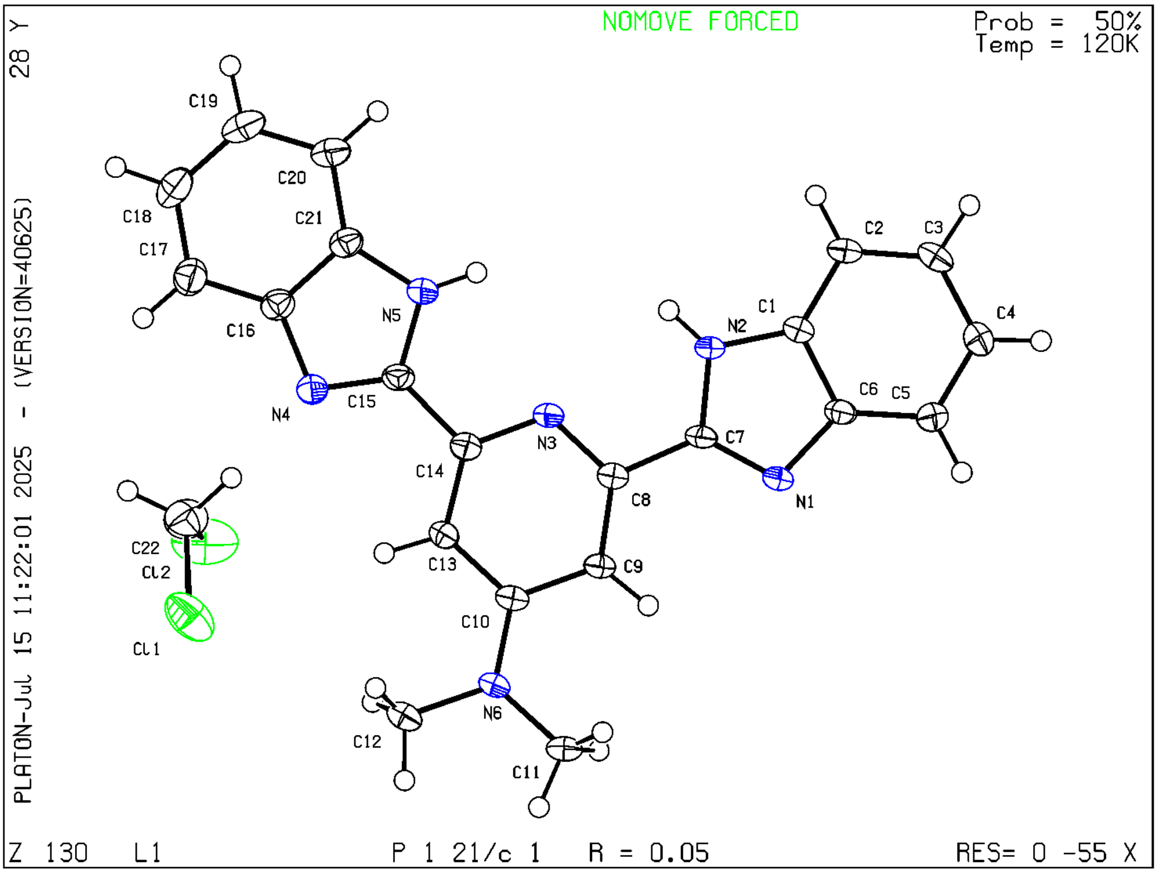

Datablock L2 - ellipsoid plot

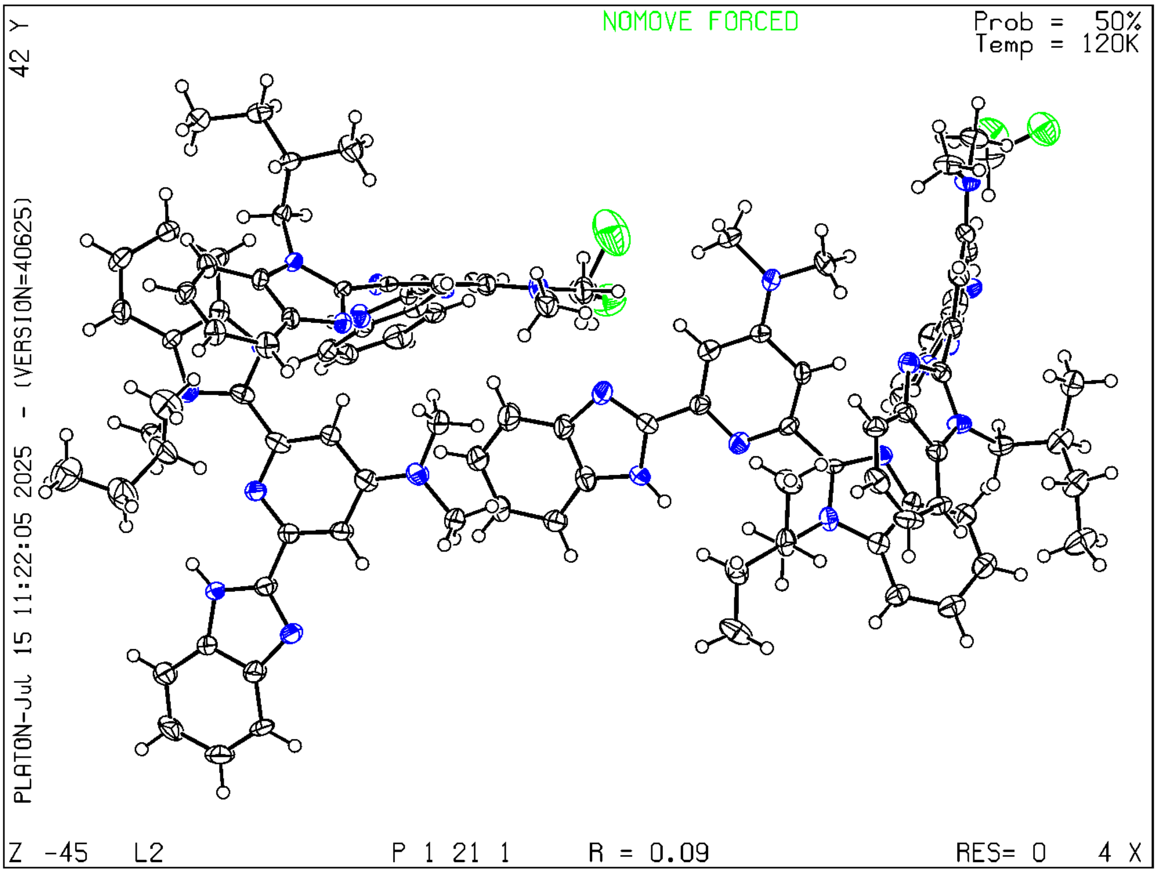

Datablock L3 - ellipsoid plot

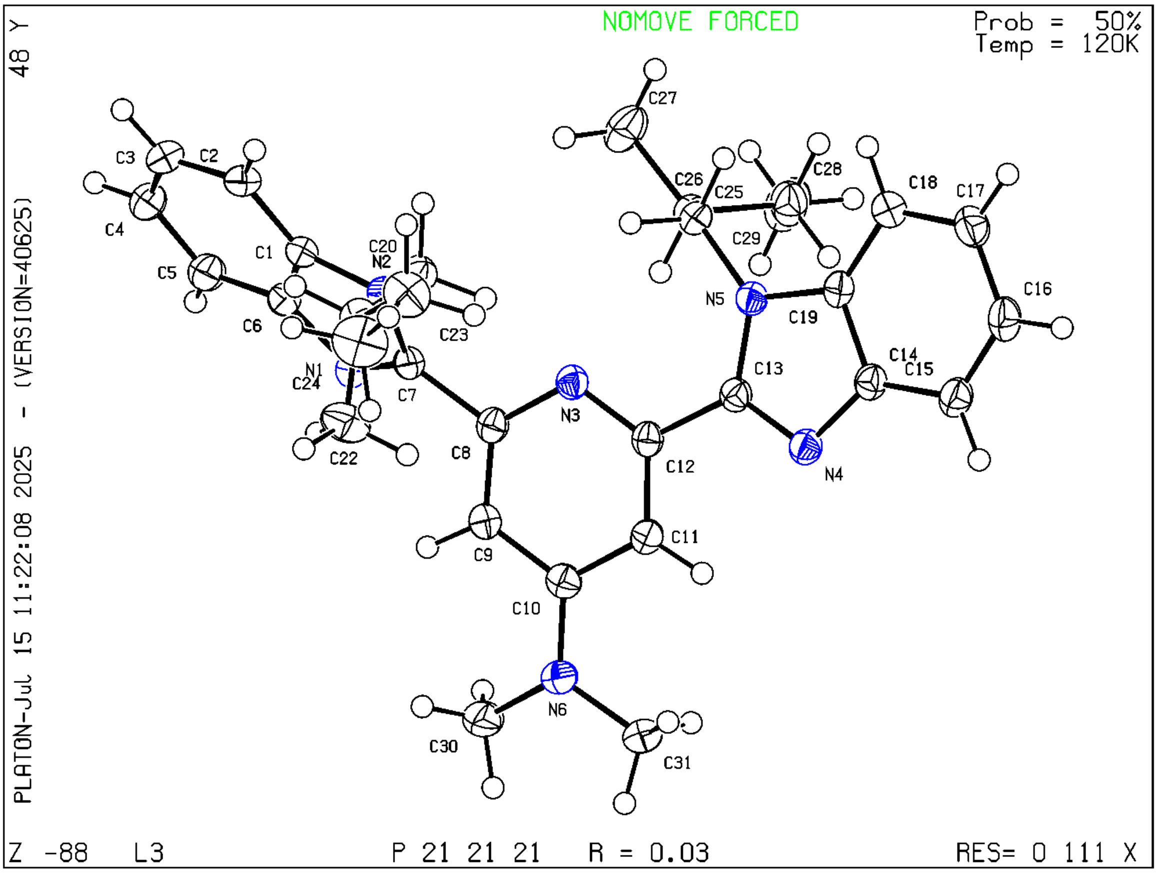

Datablock L4 - ellipsoid plot

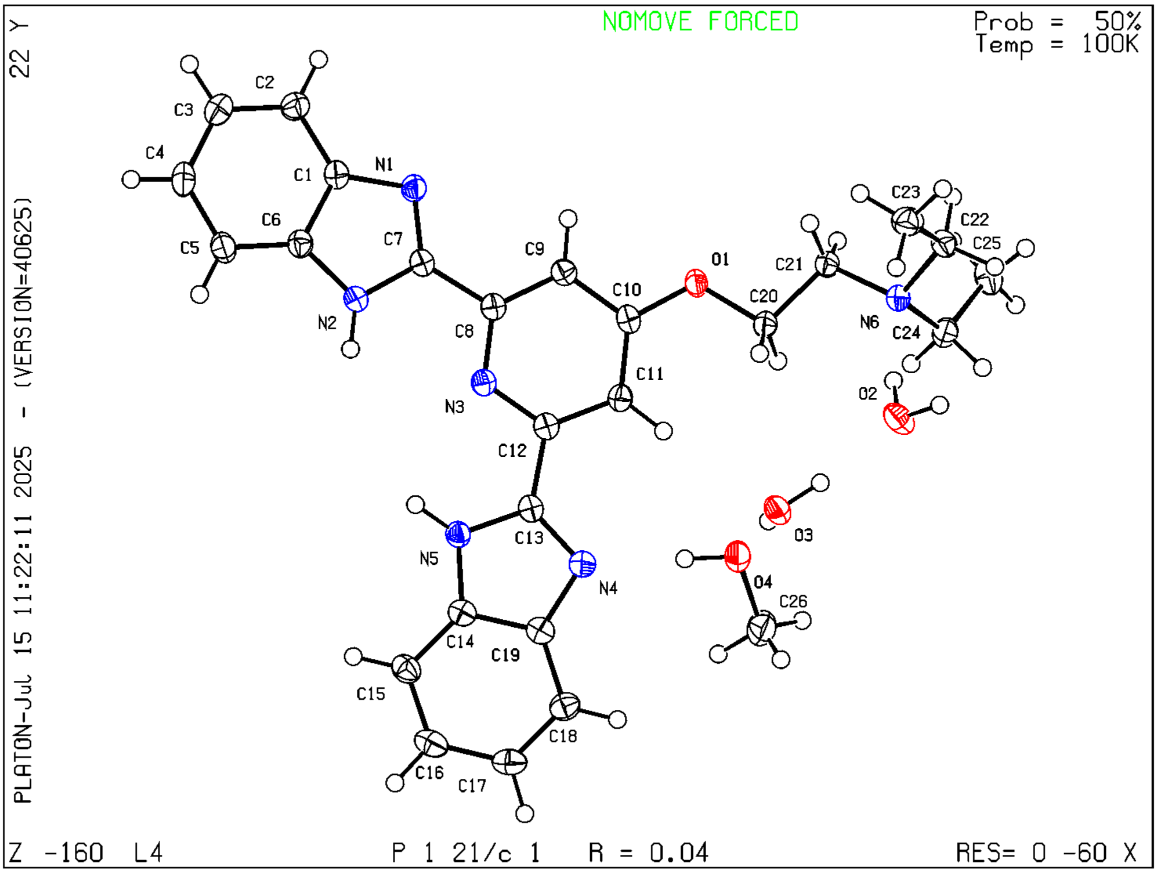

Datablock L5 - ellipsoid plot

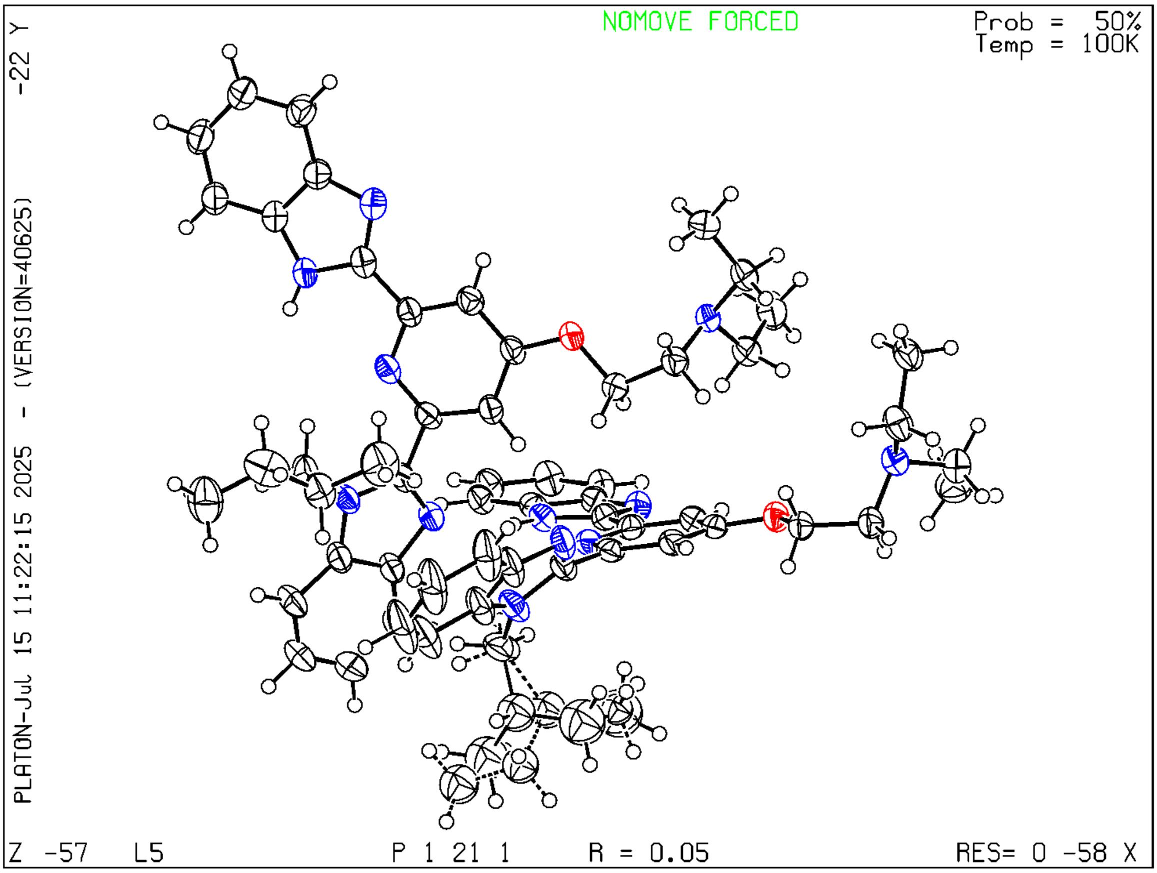

Supplement: Supplementary file 2 — Supporting Information [file CHEM-31-e02338-s002.zip › checkcif_ligands.pdf]
