# Supplementary material for: Catalytic and Photoluminescence Properties of the First‐ and Second‐Sphere Coordination of Lanthanide Complexes
Source: Chemistry. 2025 Oct 8;31(61):e02338. doi: 10.1002/chem.202502338 (PMC12587021; doi:10.1002/chem.202502338)

checkCIF/PLATON report

Structure factors have been supplied for datablock(s) L2Eu, L3Eu, L5Eu, L6Eu, L7Eu, L8Eu

THIS REPORT IS FOR GUIDANCE ONLY. IF USED AS PART OF A REVIEW PROCEDURE FOR PUBLICATION, IT SHOULD NOT REPLACE THE EXPERTISE OF AN EXPERIENCED CRYSTALLOGRAPHIC REFEREE.

No syntax errors found. CIF dictionary Interpreting this report

Datablock: L2Eu

```
Bond precision:    C-C = 0.0112 A           Wavelength=1.54184

Cell:              a=14.50390(16)      b=25.2261(3)      c=52.9888(7)
                   alpha=90            beta=90           gamma=90
Temperature:       100 K
```

	Calculated	Reported
Volume	19387.4(4)	19387.4(4)
Space group	P b c a	P b c a
Hall group	-P 2ac 2ab	-P 2ac 2ab
Moiety formula	4(C40.58 H29.75 Eu F18 N6 O6), 4(C41 H31 Eu F18 N6 O6), 2(C6 H1	2(C41 H31 Eu F18 N6 O6), 0.5(C6 H14), 0.25(C H2 Cl2)
Sum formula	C341 H278 Cl2 Eu8 F144 N48 O48	C85.25 H69.50 Cl0.50 Eu2 F36 N12 O12
Mr	9838.78	2459.67
Dx,g cm-3	1.685	1.685
Z	2	8
Mu (mm-1)	10.501	10.501
F000	9756.0	9756.0
F000'	9656.05	
h,k,lmax	18,31,66	17,31,65
Nref	19833	19499
Tmin,Tmax	0.719,0.900	0.255,0.891
Tmin'	0.037	

```
Correction method= # Reported T Limits: Tmin=0.255 Tmax=0.891
AbsCorr = ANALYTICAL

Data completeness= 0.983            Theta(max)= 74.514
```

R(reflections)= 0.0719(12671) wR2(reflections)= 0.2103(19499)

S = 1.060 Npar= 1378

The following ALERTS were generated. Each ALERT has the format
test-name_ALERT_alert-type_alert-level.
Click on the hyperlinks for more details of the test.

Alert level B

```
PLAT201_ALERT_2_B Isotropic non-H Atoms in Main Residue(s) .......          2 Report
              C53A    C54A
```

Author Response: Alkyl chains are disordered and refined in two components with isotropic ADPs for closest carbon atoms. See _refine_special_details section.

Alert level C

```
PLAT041_ALERT_1_C Calc. and Reported SumFormula    Strings  Differ     Please Check
              Calc: C341 H278 Cl2 Eu8 F144 N48 O48
              Rep.: C85.25 H69.50 Cl0.50 Eu2 F36 N12 O12
PLAT042_ALERT_1_C Calc. and Reported MoietyFormula Strings  Differ     Please Check
              Calc: 4(C40.58 H29.75 Eu F18 N6 O6), 4(C41 H31 Eu F1
                    8 N6 O6), 2(C6 H14), C H2 Cl2, 1.664(C H3)
              Rep.: 2(C41 H31 Eu F18 N6 O6), 0.5(C6 H14), 0.25(C H
                    2 Cl2)
PLAT220_ALERT_2_C NonSolvent   Resd 2  C   Ueq(max)/Ueq(min) Range        3.5 Ratio
PLAT222_ALERT_3_C NonSolvent Resd 2  H   Uiso(max)/Uiso(min) Range        4.2 Ratio
PLAT234_ALERT_4_C Large Hirshfeld Difference C10      --C12A     .       0.17 Ang.
PLAT234_ALERT_4_C Large Hirshfeld Difference C11      --C12B     .       0.21 Ang.
PLAT241_ALERT_2_C High   'MainMol' Ueq as Compared to Neighbors of        C10 Check
PLAT260_ALERT_2_C Large Average Ueq of Residue Including       Cl1      0.139 Check
PLAT342_ALERT_3_C Low Bond Precision on  C-C Bonds ...............    0.01121 Ang.
PLAT410_ALERT_2_C Short Intra H...H Contact  H8A      ..H15      .       1.95 Ang.
                                                x,y,z  =      1_555 Check
PLAT906_ALERT_3_C Large K Value in the Analysis of Variance ......      2.699 Check
PLAT911_ALERT_3_C Missing FCF Refl Between Thmin & STh/L=    0.600         28 Report
                6 20  0,   0 26  0,  15  1  1,  15  2  1,   0 26  1,  15  0  2,
               17  0  2,  15  1  2,  15  2  2,  15  1  3,  15  0  4,  17  0  4,
               17  0  6,   0  6  6,   0  6  7,  17  0  8,  17  0 10,  14  5 13,
               14  5 14,  14  6 14,  14  4 15,  14  5 15,  14  6 15,   0 28 15,
               14  4 16,  14  5 16,   0 28 16,   9  0 54,
```

Alert level G

```
PLAT002_ALERT_2_G Number of Distance or Angle Restraints on AtSite          3 Note
PLAT007_ALERT_5_G Number of Unrefined Donor-H Atoms ..............          2 Report
              H12   H6
PLAT045_ALERT_1_G Calculated and Reported Z Differ by a Factor ...      0.250 Check
PLAT072_ALERT_2_G SHELXL First  Parameter in WGHT  Unusually Large       0.10 Report
PLAT083_ALERT_2_G SHELXL Second Parameter in WGHT  Unusually Large      47.28 Why ?
PLAT171_ALERT_4_G The CIF-Embedded .res File Contains EADP Records          4 Report
```

```
PLAT172_ALERT_4_G The CIF-Embedded .res File Contains DFIX Records          2 Report
PLAT230_ALERT_2_G Hirshfeld Test Diff for    F36      --C86      .        7.8 s.u.
PLAT242_ALERT_2_G Low    'MainMol' Ueq as Compared to Neighbors of        C72 Check
PLAT242_ALERT_2_G Low    'MainMol' Ueq as Compared to Neighbors of        C76 Check
PLAT242_ALERT_2_G Low    'MainMol' Ueq as Compared to Neighbors of        C77 Check
PLAT242_ALERT_2_G Low    'MainMol' Ueq as Compared to Neighbors of        C81 Check
PLAT242_ALERT_2_G Low    'MainMol' Ueq as Compared to Neighbors of        C82 Check
PLAT242_ALERT_2_G Low    'MainMol' Ueq as Compared to Neighbors of        C86 Check
PLAT242_ALERT_2_G Low    'MainMol' Ueq as Compared to Neighbors of        C28 Check
PLAT242_ALERT_2_G Low    'MainMol' Ueq as Compared to Neighbors of        C32 Check
PLAT242_ALERT_2_G Low    'MainMol' Ueq as Compared to Neighbors of        C33 Check
PLAT242_ALERT_2_G Low    'MainMol' Ueq as Compared to Neighbors of        C37 Check
PLAT242_ALERT_2_G Low    'MainMol' Ueq as Compared to Neighbors of        C38 Check
PLAT242_ALERT_2_G Low    'MainMol' Ueq as Compared to Neighbors of        C42 Check
PLAT299_ALERT_4_G Atom Site Occupancy Constrained at ............         0.5 Check
              F36     F37     C87     C88     C89     C90     C91     C92
              H87A    H87B    H87C    H88A    H88B    H89A    H89B    H90A
              H90B    H91A    H91B    H92A    H92B    H92C
PLAT300_ALERT_4_G Atom Site Occupancy of Cl1        Constrained at       0.25 Check
PLAT300_ALERT_4_G Atom Site Occupancy of Cl2        Constrained at       0.25 Check
PLAT300_ALERT_4_G Atom Site Occupancy of C93        Constrained at       0.25 Check
PLAT300_ALERT_4_G Atom Site Occupancy of H93A       Constrained at       0.25 Check
PLAT300_ALERT_4_G Atom Site Occupancy of H93B       Constrained at       0.25 Check
PLAT301_ALERT_3_G Main Residue  Disorder .............(Resd    1)          6% Note
PLAT301_ALERT_3_G Main Residue  Disorder .............(Resd    2)          1% Note
PLAT302_ALERT_4_G Anion/Solvent/Minor-Residue Disorder (Resd    3)        100% Note
PLAT302_ALERT_4_G Anion/Solvent/Minor-Residue Disorder (Resd    4)        100% Note
PLAT302_ALERT_4_G Anion/Solvent/Minor-Residue Disorder (Resd    5)        100% Note
PLAT304_ALERT_4_G Non-Integer Number of Atoms in ..... (Resd    1)     101.34 Check
PLAT304_ALERT_4_G Non-Integer Number of Atoms in ..... (Resd    4)       1.25 Check
PLAT304_ALERT_4_G Non-Integer Number of Atoms in ..... (Resd    5)       1.66 Check
PLAT410_ALERT_2_G Short Intra H...H Contact  H50B     ..H59      .       2.01 Ang.
                                                  x,y,z  =      1_555 Check
PLAT410_ALERT_2_G Short Intra H...H Contact  H59      ..H50D     .       1.69 Ang.
                                                  x,y,z  =      1_555 Check
PLAT432_ALERT_2_G Short Inter X...Y Contact  F37      ..C12A     .       2.37 Ang.
                                        1/2-x,-1/2+y,z  =      8_655 Check
PLAT432_ALERT_2_G Short Inter X...Y Contact  C50      ..C54B     .       2.51 Ang.
                                                  x,y,z  =      1_555 Check
PLAT434_ALERT_2_G Short Inter HL..HL Contact F18      ..F20      .       2.84 Ang.
                                        1/2-x,-1/2+y,z  =      8_655 Check
PLAT773_ALERT_2_G Check long C-C Bond in CIF: C51A     --C54A            1.71 Ang.
PLAT773_ALERT_2_G Check long C-C Bond in CIF: C51B     --C54B            1.88 Ang.
PLAT793_ALERT_4_G Model has Chirality at C9         (Centro SpGr)           R Verify
PLAT793_ALERT_4_G Model has Chirality at C51A       (Centro SpGr)           S Verify
PLAT860_ALERT_3_G Number of Least-Squares Restraints ............           2 Note
PLAT910_ALERT_3_G Missing FCF Reflection(s) Below Theta(Min)[Deg]=       3.47 Note
                0  0  2,   0  0  4,
PLAT912_ALERT_4_G Missing # of FCF Reflections Above STh/L=  0.600        274 Note
PLAT969_ALERT_5_G The 'Henn et al.' R-Factor-gap value ..........      2.890 Note
              Predicted wR2: Based on SigI**2  7.28 or SHELX Weight 19.84
PLAT978_ALERT_2_G Number C-C Bonds with Positive Residual Density.          0 Info
```

---

0 **ALERT level A** = Most likely a serious problem - resolve or explain
1 **ALERT level B** = A potentially serious problem, consider carefully
12 **ALERT level C** = Check. Ensure it is not caused by an omission or oversight

48 **ALERT level G** = General information/check it is not something unexpected

```
 3 ALERT type 1 CIF construction/syntax error, inconsistent or missing data
29 ALERT type 2 Indicator that the structure model may be wrong or deficient
 8 ALERT type 3 Indicator that the structure quality may be low
19 ALERT type 4 Improvement, methodology, query or suggestion
 2 ALERT type 5 Informative message, check
```

Datablock: L3Eu

Bond precision: C-C = 0.0075 A Wavelength=1.54184

Cell:	a=10.60792(4)	b=22.54293(8)	c=21.89791(9)
	alpha=90	beta=102.7994(3)	gamma=90
Temperature:	120 K		

	Calculated	Reported
Volume	5106.41(3)	5106.41(3)
Space group	P 21	P 1 21 1
Hall group	P 2yb	P 2yb
Moiety formula	C46 H41 Eu F18 N6 O6	C46 H41 Eu F18 N6 O6
Sum formula	C46 H41 Eu F18 N6 O6	C46 H41 Eu F18 N6 O6
Mr	1267.99	1268.00
Dx,g cm-3	1.649	1.649
Z	4	4
Mu (mm-1)	9.869	9.869
F000	2528.3	2528.0
F000'	2503.17	
h,k,lmax	13,28,27	13,28,27
Nref	21348[10957]	20661
Tmin,Tmax	0.087,0.126	0.131,0.313
Tmin'	0.017	

Correction method= # Reported T Limits: Tmin=0.131 Tmax=0.313
AbsCorr = ANALYTICAL

Data completeness= 1.89/0.97 Theta(max)= 75.987

R(reflections)= 0.0346(20383) wR2(reflections)= 0.0907(20661)

S = 1.037 Npar= 1404

The following ALERTS were generated. Each ALERT has the format
test-name_ALERT_alert-type_alert-level.
Click on the hyperlinks for more details of the test.

Alert level B

```
PLAT410_ALERT_2_B Short Intra H...H Contact  H16B     ..H27A     .       1.89 Ang.
                                                      x,y,z  =      1_555 Check
```

Alert level C

```
PLAT090_ALERT_3_C Poor Data / Parameter Ratio (Zmax > 18) ........       7.63 Note
PLAT213_ALERT_2_C Atom F7B             has ADP max/min Ratio .....        3.6 prolat
PLAT220_ALERT_2_C NonSolvent   Resd 1  C   Ueq(max)/Ueq(min) Range        3.2 Ratio
PLAT410_ALERT_2_C Short Intra H...H Contact  H8BB     ..H14B     .       1.96 Ang.
                                                      x,y,z  =      1_555 Check
PLAT410_ALERT_2_C Short Intra H...H Contact  H8B      ..H14      .       1.98 Ang.
                                                      x,y,z  =      1_555 Check
PLAT911_ALERT_3_C Missing FCF Refl Between Thmin & STh/L=    0.600          2 Report
              -12  0 14, -11  0 17,
```

Alert level G

```
PLAT068_ALERT_1_G Reported F000 Differs from Calcd (or Missing)...     Please Check
PLAT142_ALERT_4_G s.u. on b - Axis Small or Missing .............    0.00008 Ang.
PLAT143_ALERT_4_G s.u. on c - Axis Small or Missing .............    0.00009 Ang.
PLAT145_ALERT_4_G s.u. on beta     Small or Missing .............     0.0003 Degree
PLAT171_ALERT_4_G The CIF-Embedded .res File Contains EADP Records          1 Report
PLAT242_ALERT_2_G Low    'MainMol' Ueq as Compared to Neighbors of       C32B Check
PLAT242_ALERT_2_G Low    'MainMol' Ueq as Compared to Neighbors of       C36B Check
PLAT242_ALERT_2_G Low    'MainMol' Ueq as Compared to Neighbors of       C37B Check
PLAT242_ALERT_2_G Low    'MainMol' Ueq as Compared to Neighbors of       C41B Check
PLAT242_ALERT_2_G Low    'MainMol' Ueq as Compared to Neighbors of       C42B Check
PLAT242_ALERT_2_G Low    'MainMol' Ueq as Compared to Neighbors of       C46B Check
PLAT242_ALERT_2_G Low    'MainMol' Ueq as Compared to Neighbors of        C32 Check
PLAT242_ALERT_2_G Low    'MainMol' Ueq as Compared to Neighbors of        C36 Check
PLAT242_ALERT_2_G Low    'MainMol' Ueq as Compared to Neighbors of        C37 Check
PLAT242_ALERT_2_G Low    'MainMol' Ueq as Compared to Neighbors of        C41 Check
PLAT242_ALERT_2_G Low    'MainMol' Ueq as Compared to Neighbors of        C42 Check
PLAT242_ALERT_2_G Low    'MainMol' Ueq as Compared to Neighbors of        C46 Check
PLAT301_ALERT_3_G Main Residue  Disorder ..............(Resd    1)         4% Note
PLAT304_ALERT_4_G Non-Integer Number of Atoms in ..... (Resd    1)     118.02 Check
PLAT434_ALERT_2_G Short Inter HL..HL Contact F8       ..F18      .       2.83 Ang.
                                                   -1+x,y,z  =      1_455 Check
PLAT720_ALERT_4_G Number of Unusual/Non-Standard Labels .........           4 Note
              H8BA    H8BB    C041    H041
PLAT790_ALERT_4_G Centre of Gravity not Within Unit Cell: Resd.  #          2 Note
              C46 H41 Eu F18 N6 O6
PLAT791_ALERT_4_G Model has Chirality at C9         (Sohncke SpGr)          S Verify
PLAT791_ALERT_4_G Model has Chirality at C9B        (Sohncke SpGr)          S Verify
PLAT791_ALERT_4_G Model has Chirality at C28        (Sohncke SpGr)          S Verify
PLAT791_ALERT_4_G Model has Chirality at C28B       (Sohncke SpGr)          S Verify
PLAT860_ALERT_3_G Number of Least-Squares Restraints ............           2 Note
PLAT910_ALERT_3_G Missing FCF Reflection(s) Below Theta(Min)[Deg]=       2.85 Note
                0  0  1,
PLAT912_ALERT_4_G Missing # of FCF Reflections Above STh/L=  0.600        244 Note
```

```
PLAT969_ALERT_5_G The 'Henn et al.' R-Factor-gap value ..........      4.093 Note
              Predicted wR2: Based on SigI**2  2.22 or SHELX Weight  8.75
PLAT978_ALERT_2_G Number C-C Bonds with Positive Residual Density.          0 Info
```

---

   0 **ALERT level A** = Most likely a serious problem - resolve or explain
   1 **ALERT level B** = A potentially serious problem, consider carefully
   6 **ALERT level C** = Check. Ensure it is not caused by an omission or oversight
  31 **ALERT level G** = General information/check it is not something unexpected

```
   1 ALERT type 1 CIF construction/syntax error, inconsistent or missing data
  19 ALERT type 2 Indicator that the structure model may be wrong or deficient
   5 ALERT type 3 Indicator that the structure quality may be low
  12 ALERT type 4 Improvement, methodology, query or suggestion
   1 ALERT type 5 Informative message, check
```

Datablock: L5Eu

Bond precision: C-C = 0.0174 A Wavelength=1.54184

Cell:	a=16.41248(9)	b=26.89715(18)	c=23.41428(16)
	alpha=90	beta=96.6752(6)	gamma=90
Temperature:	120 K		

	Calculated	Reported
Volume	10266.14(11)	10266.14(11)
Space group	P 21	P 1 21 1
Hall group	P 2yb	P 2yb
Moiety formula	C45 H39 Eu F18 N6 O7, H2 O	C45 H39 Eu F18 N6 O7, H2 O
Sum formula	C45 H41 Eu F18 N6 O8	C45 H41 Eu F18 N6 O8
Mr	1287.81	1287.80
Dx,g cm-3	1.666	1.666
Z	8	8
Mu (mm-1)	9.858	9.858
F000	5136.0	5136.0
F000'	5086.33	
h,k,lmax	20,33,29	20,33,29
Nref	42985[21967]	41648
Tmin,Tmax	0.516,0.821	0.123,0.821
Tmin'	0.001	

Correction method= # Reported T Limits: Tmin=0.123 Tmax=0.821
AbsCorr = ANALYTICAL

Data completeness= 1.90/0.97 Theta(max)= 76.227

R(reflections)= 0.0599(35490) wR2(reflections)= 0.1484(41648)

S = 1.064 Npar= 2752

The following ALERTS were generated. Each ALERT has the format
test-name_ALERT_alert-type_alert-level.
Click on the hyperlinks for more details of the test.

Alert level B

```
PLAT201_ALERT_2_B Isotropic non-H Atoms in Main Residue(s) .......          8 Report
             F9      F11     F12     F6D     F7D     F9D     F16D    F18D
```

Author Response: A few number of CF3 groups of hfac ions and flexible alkyl chains are disordered and modeled in two parts with isotropic ADPs.

```
PLAT220_ALERT_2_B NonSolvent   Resd 3  C   Ueq(max)/Ueq(min) Range        6.6 Ratio
PLAT241_ALERT_2_B High   'MainMol' Ueq as Compared to Neighbors of         F9 Check
PLAT241_ALERT_2_B High   'MainMol' Ueq as Compared to Neighbors of        F12 Check
PLAT241_ALERT_2_B High   'MainMol' Ueq as Compared to Neighbors of        F7D Check
```

Alert level C

```
PLAT090_ALERT_3_C Poor Data / Parameter Ratio (Zmax > 18) ........       7.82 Note
PLAT213_ALERT_2_C Atom F7C             has ADP max/min Ratio .....        3.4 prolat
PLAT213_ALERT_2_C Atom C17C            has ADP max/min Ratio .....        3.1 prolat
PLAT220_ALERT_2_C NonSolvent   Resd 2  C   Ueq(max)/Ueq(min) Range        3.7 Ratio
PLAT220_ALERT_2_C NonSolvent   Resd 2  F   Ueq(max)/Ueq(min) Range        3.1 Ratio
PLAT220_ALERT_2_C NonSolvent   Resd 3  F   Ueq(max)/Ueq(min) Range        3.3 Ratio
PLAT220_ALERT_2_C NonSolvent   Resd 4  C   Ueq(max)/Ueq(min) Range        3.1 Ratio
PLAT222_ALERT_3_C NonSolvent Resd 3  H   Uiso(max)/Uiso(min) Range        7.9 Ratio
PLAT241_ALERT_2_C High   'MainMol' Ueq as Compared to Neighbors of        F11 Check
PLAT241_ALERT_2_C High   'MainMol' Ueq as Compared to Neighbors of         O2 Check
PLAT241_ALERT_2_C High   'MainMol' Ueq as Compared to Neighbors of       C17C Check
PLAT241_ALERT_2_C High   'MainMol' Ueq as Compared to Neighbors of        F6D Check
PLAT241_ALERT_2_C High   'MainMol' Ueq as Compared to Neighbors of       C18D Check
PLAT242_ALERT_2_C Low    'MainMol' Ueq as Compared to Neighbors of        N6D Check
PLAT342_ALERT_3_C Low Bond Precision on  C-C Bonds ...............    0.01745 Ang.
PLAT410_ALERT_2_C Short Intra H...H Contact  H11       ..H20G     .       1.97 Ang.
                                                       x,y,z  =      1_555 Check
PLAT410_ALERT_2_C Short Intra H...H Contact  H11B      ..H20F     .       1.98 Ang.
                                                       x,y,z  =      1_555 Check
PLAT911_ALERT_3_C Missing FCF Refl Between Thmin & STh/L=    0.600         10 Report
              17   0   0,  19   0   0, -19   0   1, -19   1   1, -19   0   2, -19   1   2,
             -19   2   2, -19   0   3, -19   1   3, -17   0   6,
```

Alert level G

```
PLAT002_ALERT_2_G Number of Distance or Angle Restraints on AtSite          9 Note
PLAT007_ALERT_5_G Number of Unrefined Donor-H Atoms ..............         12 Report
             H2    H2C    H2D    H2B    H8A    H8B    H8BA   H8BB   H8CA   H8CB   H8DA
             H8DB
PLAT063_ALERT_4_G Crystal Size Possibly too Large for Beam Size ..       0.67 mm
```

```
PLAT083_ALERT_2_G SHELXL Second Parameter in WGHT  Unusually Large      20.20 Why ?
PLAT142_ALERT_4_G s.u. on b - Axis Small or Missing .............    0.00018 Ang.
PLAT143_ALERT_4_G s.u. on c - Axis Small or Missing .............    0.00016 Ang.
PLAT171_ALERT_4_G The CIF-Embedded .res File Contains EADP Records        18 Report
PLAT172_ALERT_4_G The CIF-Embedded .res File Contains DFIX Records         6 Report
PLAT242_ALERT_2_G Low    'MainMol' Ueq as Compared to Neighbors of       C31 Check
PLAT242_ALERT_2_G Low    'MainMol' Ueq as Compared to Neighbors of       C35 Check
PLAT242_ALERT_2_G Low    'MainMol' Ueq as Compared to Neighbors of       C41 Check
PLAT242_ALERT_2_G Low    'MainMol' Ueq as Compared to Neighbors of       C45 Check
PLAT242_ALERT_2_G Low    'MainMol' Ueq as Compared to Neighbors of      C31C Check
PLAT242_ALERT_2_G Low    'MainMol' Ueq as Compared to Neighbors of      C35C Check
PLAT242_ALERT_2_G Low    'MainMol' Ueq as Compared to Neighbors of      C36C Check
PLAT242_ALERT_2_G Low    'MainMol' Ueq as Compared to Neighbors of      C40C Check
PLAT242_ALERT_2_G Low    'MainMol' Ueq as Compared to Neighbors of      C41C Check
PLAT242_ALERT_2_G Low    'MainMol' Ueq as Compared to Neighbors of      C31D Check
PLAT242_ALERT_2_G Low    'MainMol' Ueq as Compared to Neighbors of      C36D Check
PLAT242_ALERT_2_G Low    'MainMol' Ueq as Compared to Neighbors of      C40D Check
PLAT242_ALERT_2_G Low    'MainMol' Ueq as Compared to Neighbors of      C41D Check
PLAT242_ALERT_2_G Low    'MainMol' Ueq as Compared to Neighbors of      C45D Check
PLAT242_ALERT_2_G Low    'MainMol' Ueq as Compared to Neighbors of      C31B Check
PLAT242_ALERT_2_G Low    'MainMol' Ueq as Compared to Neighbors of      C35B Check
PLAT242_ALERT_2_G Low    'MainMol' Ueq as Compared to Neighbors of      C36B Check
PLAT242_ALERT_2_G Low    'MainMol' Ueq as Compared to Neighbors of      C40B Check
PLAT242_ALERT_2_G Low    'MainMol' Ueq as Compared to Neighbors of      C41B Check
PLAT242_ALERT_2_G Low    'MainMol' Ueq as Compared to Neighbors of      C45B Check
PLAT299_ALERT_4_G Atom Site Occupancy Constrained at ............        0.5 Check
              F7      F8      F8E     F10     F10E    F47     O4      O4E
              O5      O5E     C36     C36E    C37     C37E    C38     C38E
              C39     C39E    C40     C40E    H38     H38E    C20E    C20F
              C21E    C21F    C22E    C22F    C23E    C23F    C24E    C24F
              H20A    H20B    H20C    H20D    H21E    H21F    H22A    H22B
              H22C    H22D    H23A    H23B    H23C    H23D    H23E    H23F
              H24A    H24B    H24C    H24D    H24E    H24F    F4D     F4E
              F5D     F5E     F8D     F17D    F45D    F46     C35D    C35E
              C27E    C27F    C28E    C28F    C29E    C29F    C30E    C30F
              H27C    H27D    H27E    H27F    H28D    H28E    H28F    H28G
              H28H    H28I    H29C    H29D    H29E    H29F    H30D    H30E
              H30F    H30G    H30H    H30I
PLAT301_ALERT_3_G Main Residue  Disorder ..............(Resd    1)        13% Note
PLAT301_ALERT_3_G Main Residue  Disorder ..............(Resd    2)         6% Note
PLAT301_ALERT_3_G Main Residue  Disorder ..............(Resd    3)         6% Note
PLAT301_ALERT_3_G Main Residue  Disorder ..............(Resd    4)         5% Note
PLAT367_ALERT_2_G Long?  C(sp?)-C(sp?) Bond  C36D     - C37D     .       1.52 Ang.
PLAT410_ALERT_2_G Short Intra H...H Contact  H11C     ..H20B     .       1.89 Ang.
                                                    x,y,z  =     1_555 Check
PLAT410_ALERT_2_G Short Intra H...H Contact  H26C     ..H27D     .       2.10 Ang.
                                                    x,y,z  =     1_555 Check
PLAT412_ALERT_2_G Short Intra XH3 .. XHn     H26C     ..H28D     .       2.11 Ang.
                                                    x,y,z  =     1_555 Check
PLAT432_ALERT_2_G Short Inter X...Y Contact  F9       ..C29F     .       2.96 Ang.
                                          1-x,1/2+y,1-z  =     2_656 Check
PLAT434_ALERT_2_G Short Inter HL..HL Contact F8B      ..F13      .       2.83 Ang.
                                                    x,y,z  =     1_555 Check
PLAT434_ALERT_2_G Short Inter HL..HL Contact F15B     ..F45D     .       2.64 Ang.
                                                    x,y,z  =     1_555 Check
PLAT720_ALERT_4_G Number of Unusual/Non-Standard Labels .........          9 Note
              H2CA    H2BA    H2DA    H8CA    H8CB    H8DA    H8DB    H8BA
```

```
              H8BB
PLAT721_ALERT_1_G Bond    Calc     0.99000, Rep     0.97990 Dev...       0.01 Ang.
              C24E     -H24C           1_555   1_555 ........     #  236 Check
PLAT721_ALERT_1_G Bond    Calc     0.97000, Rep     0.98010 Dev...       0.01 Ang.
              C24F     -H24F           1_555   1_555 ........     #  250 Check
PLAT773_ALERT_2_G Check long C-C Bond in CIF: C22F     --C23F           1.73 Ang.
PLAT779_ALERT_4_G Suspect or Irrelevant (Bond) Angle(s) in CIF ...      32.10 Deg.
              O5   -C39  -EU1     1_555   1_555   1_555 ......    #  265 Check
PLAT779_ALERT_4_G Suspect or Irrelevant (Bond) Angle(s) in CIF ...      44.50 Deg.
              C36D -F8D  -F7D     1_555   1_555   1_555 ......    #  889 Check
PLAT791_ALERT_4_G Model has Chirality at C21        (Sohncke SpGr)          S Verify
PLAT791_ALERT_4_G Model has Chirality at C21B       (Sohncke SpGr)          S Verify
PLAT791_ALERT_4_G Model has Chirality at C21D       (Sohncke SpGr)          S Verify
PLAT860_ALERT_3_G Number of Least-Squares Restraints ............           6 Note
PLAT910_ALERT_3_G Missing FCF Reflection(s) Below Theta(Min)[Deg]=       2.51 Note
                0  0  1,
PLAT912_ALERT_4_G Missing # of FCF Reflections Above STh/L=  0.600        444 Note
PLAT969_ALERT_5_G The 'Henn et al.' R-Factor-gap value ..........      2.991 Note
              Predicted wR2: Based on SigI**2  4.96 or SHELX Weight 13.95
PLAT978_ALERT_2_G Number C-C Bonds with Positive Residual Density.          0 Info
```

```
   0 ALERT level A = Most likely a serious problem - resolve or explain
   5 ALERT level B = A potentially serious problem, consider carefully
  18 ALERT level C = Check. Ensure it is not caused by an omission or oversight
  54 ALERT level G = General information/check it is not something unexpected

   2 ALERT type 1 CIF construction/syntax error, inconsistent or missing data
  50 ALERT type 2 Indicator that the structure model may be wrong or deficient
  10 ALERT type 3 Indicator that the structure quality may be low
  13 ALERT type 4 Improvement, methodology, query or suggestion
   2 ALERT type 5 Informative message, check
```

Datablock: L6Eu

```
Bond precision:   C-C = 0.0084 A           Wavelength=1.54184

Cell:            a=16.45120(12)    b=19.51761(12)      c=18.25669(13)
                 alpha=90          beta=111.8517(8)    gamma=90
Temperature:     120 K
```

	Calculated	Reported
Volume	5440.82(7)	5440.82(7)
Space group	P 21	P 1 21 1
Hall group	P 2yb	P 2yb
Moiety formula	C50 H49 Eu F18 N6 O7	C50 H49 Eu F18 N6 O7
Sum formula	C50 H49 Eu F18 N6 O7	C50 H49 Eu F18 N6 O7
Mr	1339.93	1339.91
Dx,g cm-3	1.636	1.636
Z	4	4
Mu (mm-1)	9.312	9.312
F000	2688.0	2688.0
F000'	2663.29	
h,k,lmax	20,24,22	20,24,22
Nref	22787[11739]	21914
Tmin,Tmax	0.167,0.689	0.168,0.701
Tmin'	0.041	

Correction method= # Reported T Limits: Tmin=0.168 Tmax=0.701
AbsCorr = ANALYTICAL

Data completeness= 1.87/0.96 Theta(max)= 76.175

R(reflections)= 0.0333(21332) wR2(reflections)= 0.0853(21914)

S = 1.041 Npar= 1521

The following ALERTS were generated. Each ALERT has the format
test-name_ALERT_alert-type_alert-level.
Click on the hyperlinks for more details of the test.

Alert level B

PLAT201_ALERT_2_B Isotropic non-H Atoms in Main Residue(s) .... 2 Report
C34B C35B

Author Response: The molecule labeled B has a slighlty disordered ethyl chain (C34B-C35B) that was modeled in two parts with refined occupancies 0.6/0.4, and with isotropic ADPs.

PLAT410_ALERT_2_B Short Intra H...H Contact H8A ..H14 . 1.89 Ang.
x,y,z = 1_555 Check

Alert level C

PLAT090_ALERT_3_C Poor Data / Parameter Ratio (Zmax > 18) .... 7.53 Note
PLAT213_ALERT_2_C Atom F4B has ADP max/min Ratio ..... 3.4 prolat
PLAT213_ALERT_2_C Atom F18B has ADP max/min Ratio ..... 3.5 prolat

```
PLAT213_ALERT_2_C Atom F8              has ADP max/min Ratio .....        3.2 prolat
PLAT213_ALERT_2_C Atom F18             has ADP max/min Ratio .....        3.1 prolat
PLAT213_ALERT_2_C Atom F8A             has ADP max/min Ratio .....        3.5 prolat
PLAT220_ALERT_2_C NonSolvent   Resd 1  C   Ueq(max)/Ueq(min) Range        3.8 Ratio
PLAT220_ALERT_2_C NonSolvent   Resd 1  F   Ueq(max)/Ueq(min) Range        3.7 Ratio
PLAT222_ALERT_3_C NonSolvent Resd 1  H   Uiso(max)/Uiso(min) Range        4.2 Ratio
PLAT342_ALERT_3_C Low Bond Precision on  C-C Bonds ..............    0.00841 Ang.
PLAT410_ALERT_2_C Short Intra H...H Contact  H8BA     ..H14B     .       1.91 Ang.
                                                     x,y,z  =     1_555 Check
PLAT601_ALERT_2_C Unit Cell Contains Solvent Accessible VOIDS   <=         45 Ang**3
```

Alert level G

```
PLAT142_ALERT_4_G s.u. on b - Axis Small or Missing .............    0.00012 Ang.
PLAT143_ALERT_4_G s.u. on c - Axis Small or Missing .............    0.00013 Ang.
PLAT171_ALERT_4_G The CIF-Embedded .res File Contains EADP Records          6 Report
PLAT232_ALERT_2_G Hirshfeld Test Diff (M-X)  Eu1      --N1       .        5.6 s.u.
PLAT242_ALERT_2_G Low    'MainMol' Ueq as Compared to Neighbors of       C36B Check
PLAT242_ALERT_2_G Low    'MainMol' Ueq as Compared to Neighbors of       C40B Check
PLAT242_ALERT_2_G Low    'MainMol' Ueq as Compared to Neighbors of       C41B Check
PLAT242_ALERT_2_G Low    'MainMol' Ueq as Compared to Neighbors of       C45B Check
PLAT242_ALERT_2_G Low    'MainMol' Ueq as Compared to Neighbors of       C50B Check
PLAT242_ALERT_2_G Low    'MainMol' Ueq as Compared to Neighbors of        C36 Check
PLAT242_ALERT_2_G Low    'MainMol' Ueq as Compared to Neighbors of        C40 Check
PLAT242_ALERT_2_G Low    'MainMol' Ueq as Compared to Neighbors of        C41 Check
PLAT242_ALERT_2_G Low    'MainMol' Ueq as Compared to Neighbors of        C45 Check
PLAT242_ALERT_2_G Low    'MainMol' Ueq as Compared to Neighbors of        C46 Check
PLAT242_ALERT_2_G Low    'MainMol' Ueq as Compared to Neighbors of        C50 Check
PLAT299_ALERT_4_G Atom Site Occupancy Constrained at ............         0.5 Check
              F13C    F14C    F15C
PLAT300_ALERT_4_G Atom Site Occupancy of F13D       Constrained at       0.25 Check
PLAT300_ALERT_4_G Atom Site Occupancy of F14D       Constrained at       0.25 Check
PLAT300_ALERT_4_G Atom Site Occupancy of F15D       Constrained at       0.25 Check
PLAT300_ALERT_4_G Atom Site Occupancy of F19        Constrained at       0.25 Check
PLAT300_ALERT_4_G Atom Site Occupancy of F20        Constrained at       0.25 Check
PLAT300_ALERT_4_G Atom Site Occupancy of F21        Constrained at       0.25 Check
PLAT301_ALERT_3_G Main Residue  Disorder ..............(Resd    1)         6% Note
PLAT301_ALERT_3_G Main Residue  Disorder ..............(Resd    2)         4% Note
PLAT410_ALERT_2_G Short Intra H...H Contact  H16B     ..H32B     .       2.14 Ang.
                                                     x,y,z  =     1_555 Check
PLAT410_ALERT_2_G Short Intra H...H Contact  H16B     ..H32A     .       2.14 Ang.
                                                     x,y,z  =     1_555 Check
PLAT412_ALERT_2_G Short Intra XH3 .. XHn     H33B     ..H35C     .       2.13 Ang.
                                                     x,y,z  =     1_555 Check
PLAT434_ALERT_2_G Short Inter HL..HL Contact F17B     ..F7A      .       2.67 Ang.
                                         1-x,-1/2+y,1-z  =     2_646 Check
PLAT720_ALERT_4_G Number of Unusual/Non-Standard Labels .........          2 Note
              H8BA    H8BB
PLAT791_ALERT_4_G Model has Chirality at C9         (Sohncke SpGr)          S Verify
PLAT791_ALERT_4_G Model has Chirality at C9B        (Sohncke SpGr)          S Verify
PLAT791_ALERT_4_G Model has Chirality at C32        (Sohncke SpGr)          S Verify
PLAT791_ALERT_4_G Model has Chirality at C32B       (Sohncke SpGr)          S Verify
PLAT912_ALERT_4_G Missing # of FCF Reflections Above STh/L=  0.600        285 Note
PLAT969_ALERT_5_G The 'Henn et al.' R-Factor-gap value ..........      3.972 Note
              Predicted wR2: Based on SigI**2  2.15 or SHELX Weight  8.19
PLAT978_ALERT_2_G Number C-C Bonds with Positive Residual Density.          0 Info
```

```
 0 ALERT level A = Most likely a serious problem - resolve or explain
 2 ALERT level B = A potentially serious problem, consider carefully
12 ALERT level C = Check. Ensure it is not caused by an omission or oversight
36 ALERT level G = General information/check it is not something unexpected

 0 ALERT type 1 CIF construction/syntax error, inconsistent or missing data
28 ALERT type 2 Indicator that the structure model may be wrong or deficient
 5 ALERT type 3 Indicator that the structure quality may be low
16 ALERT type 4 Improvement, methodology, query or suggestion
 1 ALERT type 5 Informative message, check
```

Datablock: L7Eu

Bond precision: C-C = 0.0125 A Wavelength=1.54184

Cell:	a=11.4446(1)	b=12.7362(1)	c=21.2364(2)
	alpha=102.809(1)	beta=96.848(1)	gamma=107.454(1)
Temperature:	100 K		

	Calculated	Reported
Volume	2821.45(5)	2821.45(5)
Space group	P -1	P -1
Hall group	-P 1	-P 1
Moiety formula	C51 H33 Eu F18 N6 O7 [+ solvent]	C51 H33 Eu F18 N6 O7, 0.625[C6H14]
Sum formula	C51 H33 Eu F18 N6 O7 [+ solvent]	C54.75 H41.75 Eu F18 N6 O7
Mr	1335.81	1389.65
Dx,g cm-3	1.572	1.636
Z	2	2
Mu (mm-1)	8.984	9.008
F000	1324.0	1386.0
F000’	1311.69	
h,k,lmax	14,16,26	14,15,26
Nref	11798	11454
Tmin,Tmax	0.247,0.532	0.379,1.000
Tmin’	0.003	

Correction method= # Reported T Limits: Tmin=0.379 Tmax=1.000
AbsCorr = MULTI-SCAN

Data completeness= 0.971 Theta(max)= 76.195

R(reflections)= 0.0688(10833) wR2(reflections)= 0.1960(11454)

S = 1.069 Npar= 798

The following ALERTS were generated. Each ALERT has the format
test-name_ALERT_alert-type_alert-level.
Click on the hyperlinks for more details of the test.

Alert level A

```
PLAT201_ALERT_2_A Isotropic non-H Atoms in Main Residue(s) .......         12 Report
                F4      F5      F6      F13A    F14A    F15A    F16A    F17A
                F18A    C47A    C48     C51A
```

Author Response: hfac ions and their CF3 groups are disordered around Eu atom. They were refined in two components with isotropic ADPs. See refine_special_details section.

Alert level C

```
PLAT220_ALERT_2_C NonSolvent   Resd 1  C   Ueq(max)/Ueq(min) Range        4.6 Ratio
PLAT234_ALERT_4_C Large Hirshfeld Difference F9       --C42      .       0.19 Ang.
PLAT234_ALERT_4_C Large Hirshfeld Difference O7       --C30      .       0.16 Ang.
PLAT234_ALERT_4_C Large Hirshfeld Difference C22      --C30B     .       0.16 Ang.
PLAT234_ALERT_4_C Large Hirshfeld Difference C34      --C35      .       0.18 Ang.
PLAT241_ALERT_2_C High   'MainMol' Ueq as Compared to Neighbors of         F3 Check
PLAT342_ALERT_3_C Low Bond Precision on  C-C Bonds ...............    0.01246 Ang.
PLAT431_ALERT_2_C Short Inter HL..A Contact  F7       ..O1       .       2.88 Ang.
                                                1-x,-y,1-z   =      2_656 Check
PLAT911_ALERT_3_C Missing FCF Refl Between Thmin & STh/L=    0.600          8 Report
                 3  0  0,   0  1  0,  -1 -1  2,  -1  0  2,  -9 -8  7,  -9 -7  7,
                -8 -9  9,  -8 -9 10,
PLAT918_ALERT_3_C Reflection(s) with I(obs) much Smaller I(calc) .          2 Check
PLAT971_ALERT_2_C Check Calcd Resid. Dens.  1.48Ang From F9              1.84 eA-3
PLAT971_ALERT_2_C Check Calcd Resid. Dens.  1.07Ang From F7              1.52 eA-3
PLAT972_ALERT_2_C Check Calcd Resid. Dens.  1.01Ang From Eu1            -1.83 eA-3
PLAT972_ALERT_2_C Check Calcd Resid. Dens.  1.00Ang From Eu1            -1.78 eA-3
PLAT977_ALERT_2_C Check Negative Difference Density on H5        .      -0.32 eA-3
```

Alert level G

```
FORMU01_ALERT_2_G  There is a discrepancy between the atom counts in the
            _chemical_formula_sum and the formula from the _atom_site* data.
            Atom count from _chemical_formula_sum:C54.75 H41.75 Eu1 F18 N6 O7
            Atom count from the _atom_site data:  C51 H33 Eu1 F18 N6 O7
CELLZ01_ALERT_1_G Difference between formula and atom_site contents detected.
CELLZ01_ALERT_1_G ALERT: Large difference may be due to a
            symmetry error - see SYMMG tests
           From the CIF: _cell_formula_units_Z    2
           From the CIF: _chemical_formula_sum  C54.75 H41.75 Eu F18 N6 O7
           TEST: Compare cell contents of formula and atom_site data
```

```
          atom    Z*formula  cif sites diff
          C        109.50    102.00    7.50
          H         83.50     66.00   17.50
          Eu         2.00      2.00    0.00
          F         36.00     36.00   -0.00
          N         12.00     12.00    0.00
          O         14.00     14.00    0.00
PLAT002_ALERT_2_G Number of Distance or Angle Restraints on AtSite          8 Note
PLAT007_ALERT_5_G Number of Unrefined Donor-H Atoms .............          2 Report
             H2    H5
PLAT041_ALERT_1_G Calc. and Reported SumFormula    Strings  Differ     Please Check
             Calc: C51 H33 Eu F18 N6 O7
             Rep.: C54.75 H41.75 Eu F18 N6 O7
PLAT042_ALERT_1_G Calc. and Reported MoietyFormula Strings  Differ     Please Check
             Calc: C51 H33 Eu F18 N6 O7
             Rep.: C51 H33 Eu F18 N6 O7, 0.625[C6H14]
PLAT063_ALERT_4_G Crystal Size Possibly too Large for Beam Size ..       0.61 mm
PLAT072_ALERT_2_G SHELXL First  Parameter in WGHT  Unusually Large       0.14 Report
PLAT083_ALERT_2_G SHELXL Second Parameter in WGHT  Unusually Large       6.22 Why ?
PLAT154_ALERT_1_G The s.u.'s on the Cell Angles are Equal ..(Note)      0.001 Degree
PLAT171_ALERT_4_G The CIF-Embedded .res File Contains EADP Records          6 Report
PLAT172_ALERT_4_G The CIF-Embedded .res File Contains DFIX Records          6 Report
PLAT242_ALERT_2_G Low    'MainMol' Ueq as Compared to Neighbors of        C41 Check
PLAT242_ALERT_2_G Low    'MainMol' Ueq as Compared to Neighbors of        C42 Check
PLAT242_ALERT_2_G Low    'MainMol' Ueq as Compared to Neighbors of        C46 Check
PLAT301_ALERT_3_G Main Residue  Disorder .............(Resd    1)        23% Note
PLAT412_ALERT_2_G Short Intra XH3 .. XHn     H20A     ..H30C     .       1.75 Ang.
                                                   x,y,z  =      1_555 Check
PLAT432_ALERT_2_G Short Inter X...Y Contact  F17B     ..C27      .       2.95 Ang.
                                              -x,1-y,1-z  =      2_566 Check
PLAT605_ALERT_4_G Largest Solvent Accessible VOID in the Structure        258 A**3
PLAT773_ALERT_2_G Check long C-C Bond in CIF: C47B     --C53             2.03 Ang.
PLAT779_ALERT_4_G Suspect or Irrelevant (Bond) Angle(s) in CIF ...      29.40 Deg.
             O6   -C54  -EU1     1_555   1_555   1_555 ......     #  275 Check
PLAT779_ALERT_4_G Suspect or Irrelevant (Bond) Angle(s) in CIF ...      43.70 Deg.
             C54  -C47B -C53     1_555   1_555   1_555 ......     #  281 Check
PLAT793_ALERT_4_G Model has Chirality at C29        (Centro SpGr)          R Verify
PLAT860_ALERT_3_G Number of Least-Squares Restraints ............          5 Note
PLAT868_ALERT_4_G ALERTS Due to the Use of _smtbx_masks Suppressed          ! Info
PLAT912_ALERT_4_G Missing # of FCF Reflections Above STh/L=  0.600        330 Note
PLAT933_ALERT_2_G Number of HKL-OMIT Records in Embedded .res File          4 Note
              -1 -1  2,  -1  0  2,   0  1  0,   3  0  0,
PLAT969_ALERT_5_G The 'Henn et al.' R-Factor-gap value ..........      8.062 Note
             Predicted wR2: Based on SigI**2  2.43 or SHELX Weight 18.34
PLAT978_ALERT_2_G Number C-C Bonds with Positive Residual Density.          0 Info
```

---

1 **ALERT level A** = Most likely a serious problem - resolve or explain
0 **ALERT level B** = A potentially serious problem, consider carefully
15 **ALERT level C** = Check. Ensure it is not caused by an omission or oversight
30 **ALERT level G** = General information/check it is not something unexpected

5 ALERT type 1 CIF construction/syntax error, inconsistent or missing data
21 ALERT type 2 Indicator that the structure model may be wrong or deficient
5 ALERT type 3 Indicator that the structure quality may be low
13 ALERT type 4 Improvement, methodology, query or suggestion
2 ALERT type 5 Informative message, check

Datablock: L8Eu

Bond precision: C-C = 0.0104 A Wavelength=1.54184

Cell:	a=13.84187(9)	b=16.00060(12)	c=16.99570(11)
	alpha=67.6660(7)	beta=77.4988(6)	gamma=66.8835(7)
Temperature:	150 K		

	Calculated	Reported
Volume	3192.22(4)	3192.22(4)
Space group	P -1	P -1
Hall group	-P 1	-P 1
Moiety formula	2(C61 H53 Eu F18 N6 O7), C0.50 H Cl	C61 H53 Eu F18 N6 O7, C0.25 H0.5 Cl0.5
Sum formula	C122.50 H107 Cl Eu2 F36 N12 O14	C61.25 H53.50 Cl0.50 Eu F18 N6 O7
Mr	2994.60	1497.28
Dx,g cm-3	1.558	1.558
Z	1	2
Mu (mm-1)	8.194	8.194
F000	1505.0	1505.0
F000'	1493.39	
h,k,lmax	17,20,21	17,19,21
Nref	13325	12967
Tmin,Tmax	0.290,0.345	0.116,0.447
Tmin'	0.003	

Correction method= # Reported T Limits: Tmin=0.116 Tmax=0.447
AbsCorr = ANALYTICAL

Data completeness= 0.973 Theta(max)= 76.009

R(reflections)= 0.0618(12400) wR2(reflections)= 0.1713(12967)

S = 1.021 Npar= 904

The following ALERTS were generated. Each ALERT has the format
test-name_ALERT_alert-type_alert-level.
Click on the hyperlinks for more details of the test.

Alert level B

PLAT201_ALERT_2_B Isotropic non-H Atoms in Main Residue(s) .... 7 Report
F15A F16A F17A F18A C53A C56A C57A

Author Response: Hfac ions are disordered aurond Europium. Closest C and F atoms (almost superimposed) were refined with isotropic ADPs.

```
PLAT230_ALERT_2_B Hirshfeld Test Diff for    C33      --C34      .        8.6 s.u.
PLAT241_ALERT_2_B High   'MainMol' Ueq as Compared to Neighbors of        F14 Check
PLAT410_ALERT_2_B Short Intra H...H Contact  H8B      ..H14      .       1.85 Ang.
                                                      x,y,z  =      1_555 Check
```

Alert level C

```
PLAT041_ALERT_1_C Calc. and Reported SumFormula    Strings  Differ     Please Check
             Calc: C122.50 H107 Cl Eu2 F36 N12 O14
             Rep.: C61.25 H53.50 Cl0.50 Eu F18 N6 O7
PLAT042_ALERT_1_C Calc. and Reported MoietyFormula Strings  Differ     Please Check
             Calc: 2(C61 H53 Eu F18 N6 O7), C0.50 H Cl
             Rep.: C61 H53 Eu F18 N6 O7, C0.25 H0.5 Cl0.5
PLAT213_ALERT_2_C Atom F5              has ADP max/min Ratio .....        3.7 prolat
PLAT220_ALERT_2_C NonSolvent   Resd 1  C   Ueq(max)/Ueq(min) Range        3.8 Ratio
PLAT220_ALERT_2_C NonSolvent   Resd 1  F   Ueq(max)/Ueq(min) Range        3.2 Ratio
PLAT222_ALERT_3_C NonSolvent Resd 1  H   Uiso(max)/Uiso(min) Range        4.4 Ratio
PLAT234_ALERT_4_C Large Hirshfeld Difference C10      --C11      .       0.19 Ang.
PLAT234_ALERT_4_C Large Hirshfeld Difference C32      --C33      .       0.16 Ang.
PLAT234_ALERT_4_C Large Hirshfeld Difference C35      --C36      .       0.16 Ang.
PLAT234_ALERT_4_C Large Hirshfeld Difference C37      --C38      .       0.18 Ang.
PLAT241_ALERT_2_C High   'MainMol' Ueq as Compared to Neighbors of        F13 Check
PLAT241_ALERT_2_C High   'MainMol' Ueq as Compared to Neighbors of        C34 Check
PLAT241_ALERT_2_C High   'MainMol' Ueq as Compared to Neighbors of        C37 Check
PLAT260_ALERT_2_C Large Average Ueq of Residue Including       Cl1      0.114 Check
PLAT331_ALERT_2_C Small Aver Phenyl C-C Dist C33      --C38      .       1.36 Ang.
PLAT332_ALERT_2_C Large Phenyl C-C Range     C33       -C38      .       0.18 Ang.
PLAT342_ALERT_3_C Low Bond Precision on  C-C Bonds ..............     0.0104 Ang.
PLAT360_ALERT_2_C Short  C(sp3)-C(sp3) Bond  C10      - C12      .       1.41 Ang.
PLAT410_ALERT_2_C Short Intra H...H Contact  H16      ..H25B     .       1.98 Ang.
                                                      x,y,z  =      1_555 Check
PLAT911_ALERT_3_C Missing FCF Refl Between Thmin & STh/L=    0.600         14 Report
              11 13  0,  11 18  7,   9 16  8,  10 18  8,  11 18  8,  10 18  9,
              11 18  9,  10 18 10,  11 18 10,  10 18 11,  12  9 12,  12 10 12,
              12 11 12,  10 18 12,
PLAT918_ALERT_3_C Reflection(s) with I(obs) much Smaller I(calc) .          8 Check
PLAT971_ALERT_2_C Check Calcd Resid. Dens.  0.95Ang From F16A           1.57 eA-3
PLAT975_ALERT_2_C Check Calcd Resid. Dens.  0.49Ang From C62     .       0.94 eA-3
PLAT975_ALERT_2_C Check Calcd Resid. Dens.  0.62Ang From C62     .       0.92 eA-3
PLAT975_ALERT_2_C Check Calcd Resid. Dens.  1.05Ang From C30     .       0.63 eA-3
PLAT975_ALERT_2_C Check Calcd Resid. Dens.  1.06Ang From C30     .       0.63 eA-3
```

Alert level G

```
PLAT002_ALERT_2_G Number of Distance or Angle Restraints on AtSite          8 Note
PLAT045_ALERT_1_G Calculated and Reported Z Differ by a Factor ...      0.500 Check
PLAT063_ALERT_4_G Crystal Size Possibly too Large for Beam Size ..       0.70 mm
PLAT072_ALERT_2_G SHELXL First  Parameter in WGHT  Unusually Large       0.11 Report
PLAT083_ALERT_2_G SHELXL Second Parameter in WGHT  Unusually Large       8.45 Why ?
PLAT169_ALERT_4_G The CIF-Embedded .res File Contains AFIX 1 Recds          2 Report
PLAT171_ALERT_4_G The CIF-Embedded .res File Contains EADP Records          4 Report
PLAT172_ALERT_4_G The CIF-Embedded .res File Contains DFIX Records          3 Report
```

```
PLAT173_ALERT_4_G The CIF-Embedded .res File Contains DANG Records          2 Report
PLAT180_ALERT_4_G Check Cell Rounding: # of Values Ending with 0 =          3 Note
PLAT242_ALERT_2_G Low    'MainMol' Ueq as Compared to Neighbors of        C47 Check
PLAT242_ALERT_2_G Low    'MainMol' Ueq as Compared to Neighbors of        C51 Check
PLAT242_ALERT_2_G Low    'MainMol' Ueq as Compared to Neighbors of        C52 Check
PLAT242_ALERT_2_G Low    'MainMol' Ueq as Compared to Neighbors of        C61 Check
PLAT299_ALERT_4_G Atom Site Occupancy Constrained at ............         0.5 Check
              F10A    F10B    F11A    F11B    F12A    F12B    Cl1
PLAT300_ALERT_4_G Atom Site Occupancy of C62        Constrained at       0.25 Check
PLAT300_ALERT_4_G Atom Site Occupancy of H62A       Constrained at       0.25 Check
PLAT300_ALERT_4_G Atom Site Occupancy of H62B       Constrained at       0.25 Check
PLAT301_ALERT_3_G Main Residue  Disorder ..............(Resd    1)        17% Note
PLAT302_ALERT_4_G Anion/Solvent/Minor-Residue Disorder (Resd    2)       100% Note
PLAT304_ALERT_4_G Non-Integer Number of Atoms in ..... (Resd    2)       2.50 Check
PLAT343_ALERT_2_G Unusual sp?      Angle Range in Main Residue for        C30 Check
PLAT367_ALERT_2_G Long?  C(sp?)-C(sp?) Bond  C30      - C31      .       1.52 Ang.
PLAT793_ALERT_4_G Model has Chirality at C32         (Centro SpGr)          R Verify
PLAT860_ALERT_3_G Number of Least-Squares Restraints ............           5 Note
PLAT912_ALERT_4_G Missing # of FCF Reflections Above STh/L=  0.600        330 Note
PLAT969_ALERT_5_G The 'Henn et al.' R-Factor-gap value ..........       8.302 Note
              Predicted wR2: Based on SigI**2  2.06 or SHELX Weight 16.78
PLAT978_ALERT_2_G Number C-C Bonds with Positive Residual Density.          0 Info
```

---

0 **ALERT level A** = Most likely a serious problem - resolve or explain
4 **ALERT level B** = A potentially serious problem, consider carefully
26 **ALERT level C** = Check. Ensure it is not caused by an omission or oversight
28 **ALERT level G** = General information/check it is not something unexpected

3 ALERT type 1 CIF construction/syntax error, inconsistent or missing data
30 ALERT type 2 Indicator that the structure model may be wrong or deficient
6 ALERT type 3 Indicator that the structure quality may be low
18 ALERT type 4 Improvement, methodology, query or suggestion
1 ALERT type 5 Informative message, check

It is advisable to attempt to resolve as many as possible of the alerts in all categories. Often the minor alerts point to easily fixed oversights, errors and omissions in your CIF or refinement strategy, so attention to these fine details can be worthwhile. In order to resolve some of the more serious problems it may be necessary to carry out additional measurements or structure refinements. However, the purpose of your study may justify the reported deviations and the more serious of these should normally be commented upon in the discussion or experimental section of a paper or in the "special_details" fields of the CIF. checkCIF was carefully designed to identify outliers and unusual parameters, but every test has its limitations and alerts that are not important in a particular case may appear. Conversely, the absence of alerts does not guarantee there are no aspects of the results needing attention. It is up to the individual to critically assess their own results and, if necessary, seek expert advice.

Publication of your CIF in IUCr journals

A basic structural check has been run on your CIF. These basic checks will be run on all CIFs submitted for publication in IUCr journals (*Acta Crystallographica*, *Journal of Applied Crystallography*, *Journal of Synchrotron Radiation*); however, if you intend to submit to *Acta Crystallographica Section C* or *E* or *IUCrData*, you should make sure that full publication checks are run on the final version of your CIF prior to submission.

Publication of your CIF in other journals

Please refer to the *Notes for Authors* of the relevant journal for any special instructions relating to CIF submission.

Validation response form

Please find below a validation response form (VRF) that can be filled in and pasted into your CIF.

```
# start Validation Reply Form
_vrf_PLAT410_L3Eu
;
PROBLEM: Short Intra H...H Contact  H16B     ..H27A     .       1.89 Ang.
RESPONSE: ...
;
_vrf_PLAT220_L5Eu
;
PROBLEM: NonSolvent   Resd 3  C   Ueq(max)/Ueq(min) Range        6.6 Ratio
RESPONSE: ...
;
_vrf_PLAT241_L5Eu
;
PROBLEM: High   'MainMol' Ueq as Compared to Neighbors of         F9 Check
RESPONSE: ...
;
_vrf_PLAT410_L6Eu
;
PROBLEM: Short Intra H...H Contact  H8A      ..H14      .       1.89 Ang.
RESPONSE: ...
;
_vrf_PLAT230_L8Eu
```

```
;
PROBLEM: Hirshfeld Test Diff for    C33      --C34      .         8.6 s.u.
RESPONSE: ...
;
_vrf_PLAT241_L8Eu
;
PROBLEM: High   'MainMol' Ueq as Compared to Neighbors of         F14 Check
RESPONSE: ...
;
_vrf_PLAT410_L8Eu
;
PROBLEM: Short Intra H...H Contact  H8B      ..H14      .        1.85 Ang.
RESPONSE: ...
;
# end Validation Reply Form
```

PLATON version of 04/06/2025; check.def file version of 30/05/2025

Datablock L2Eu - ellipsoid plot

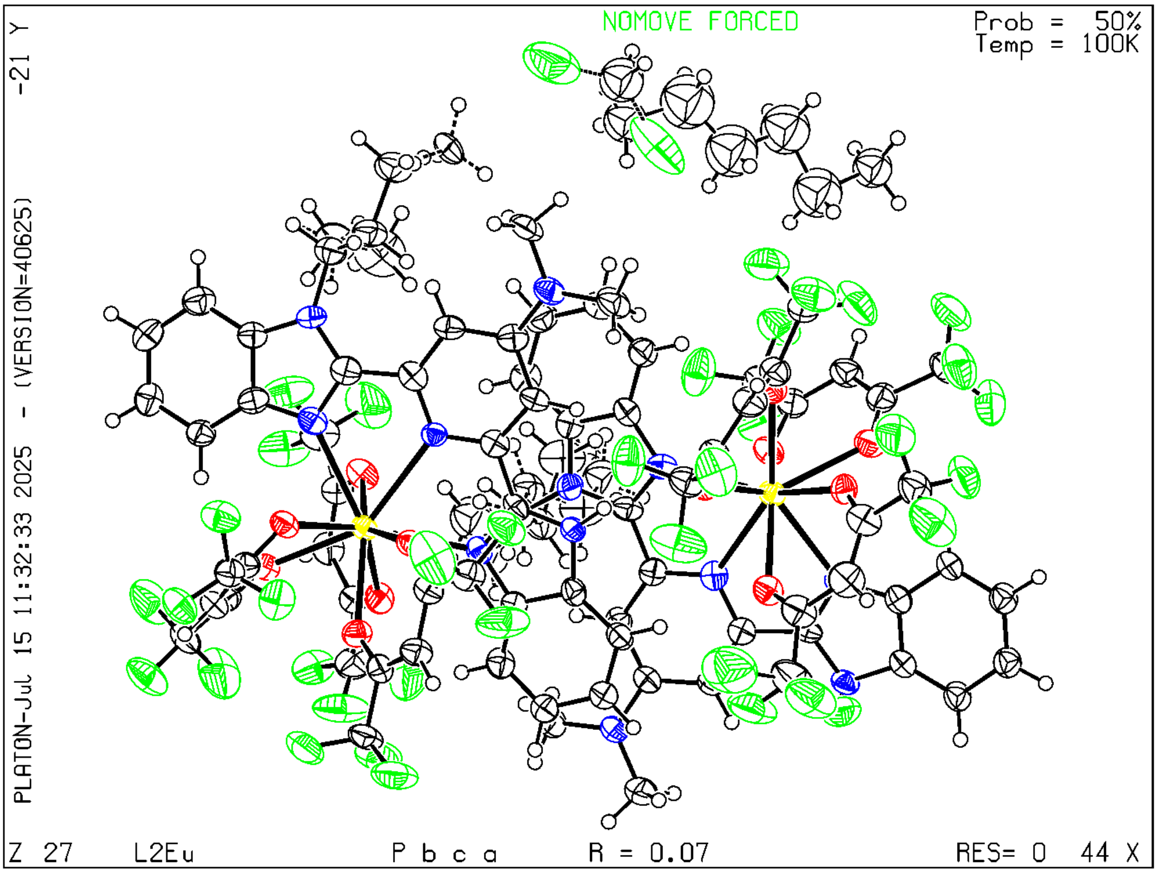

Datablock L3Eu - ellipsoid plot

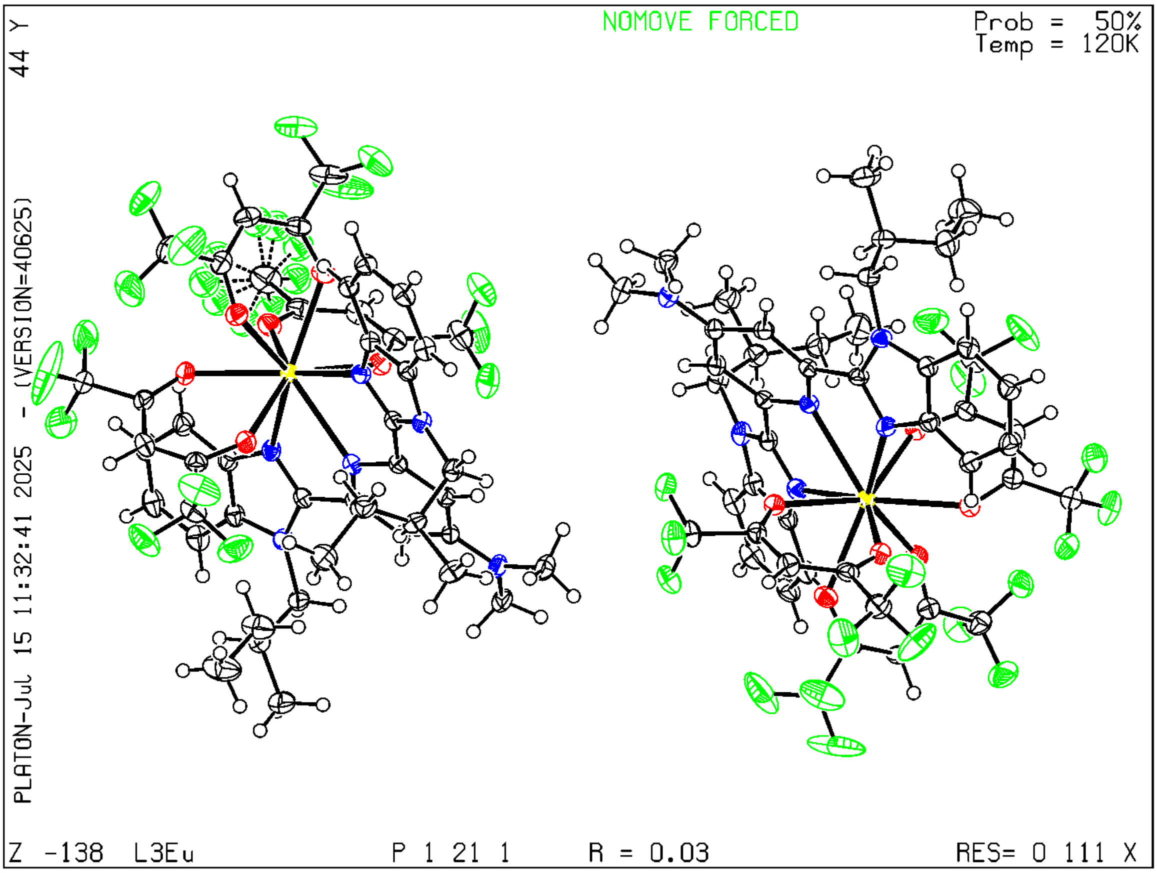

Datablock L5Eu - ellipsoid plot

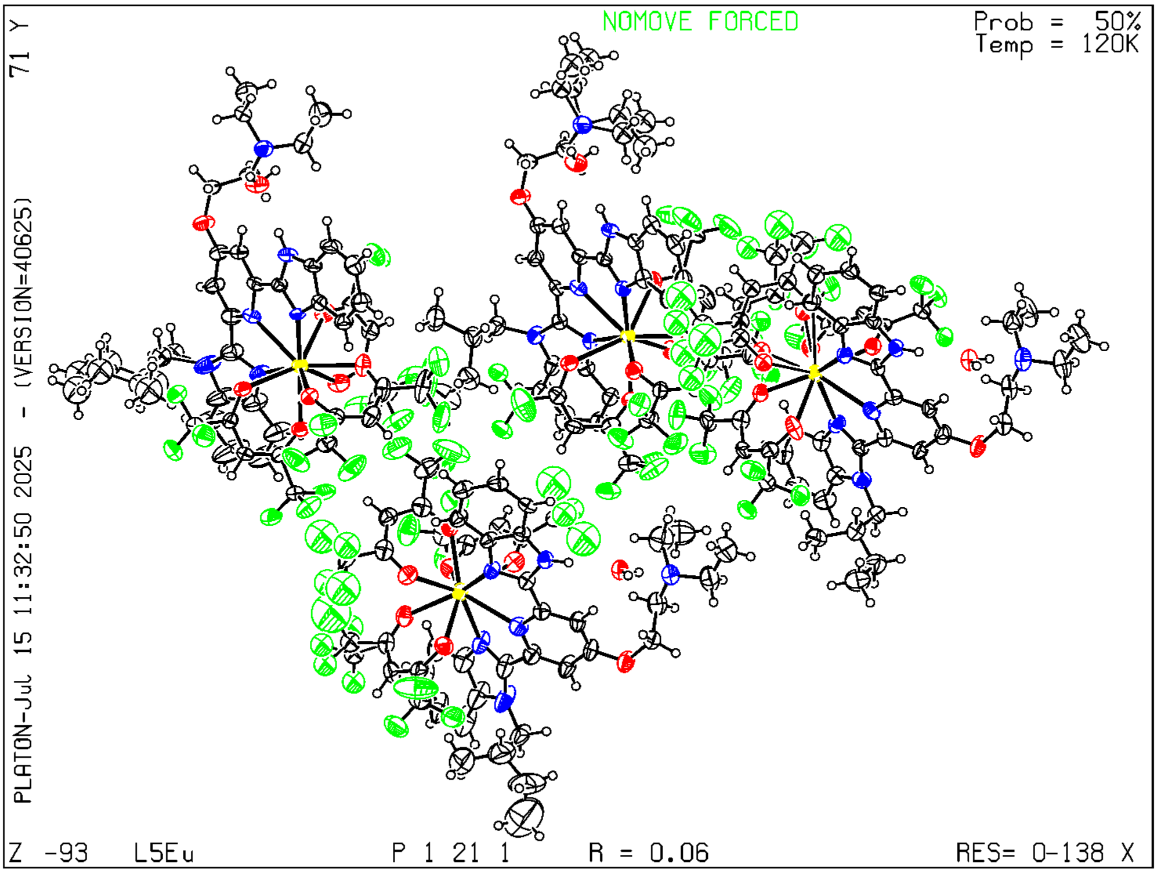

Datablock L6Eu - ellipsoid plot

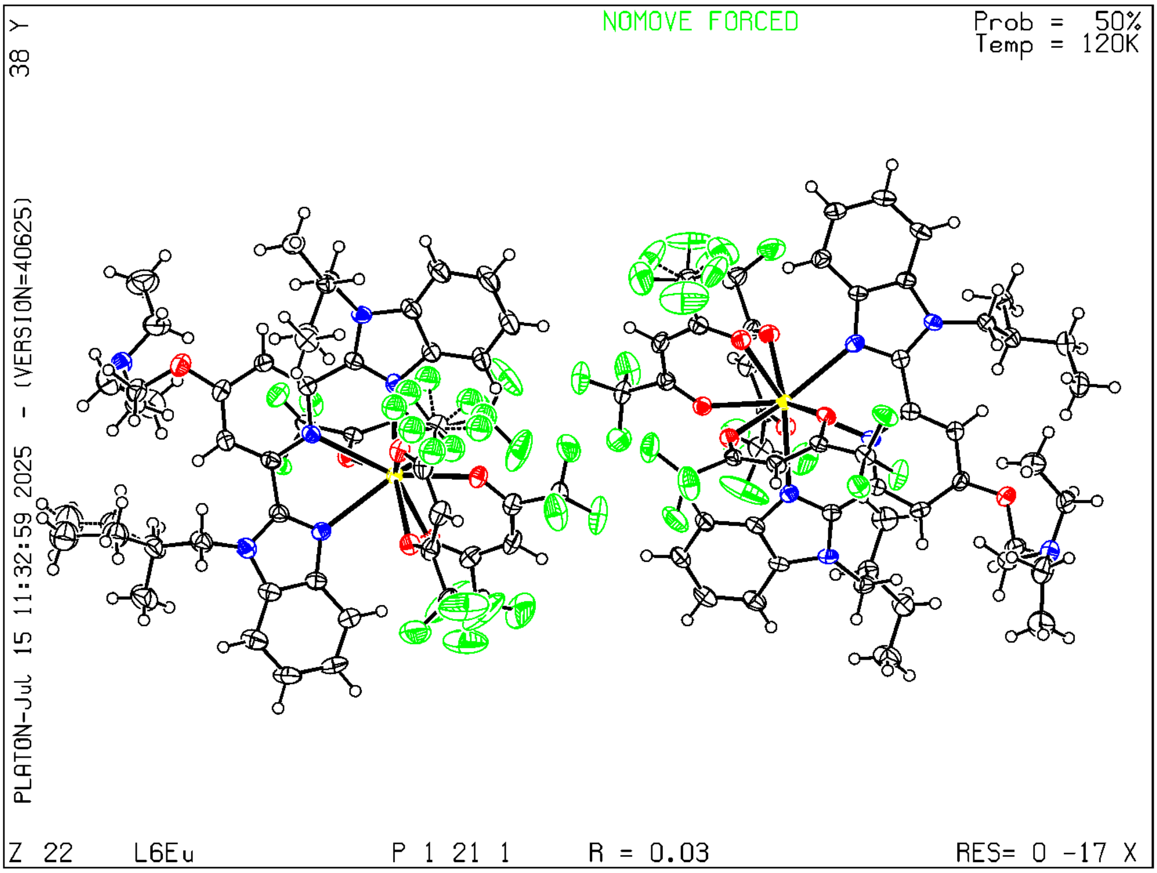

Datablock L7Eu - ellipsoid plot

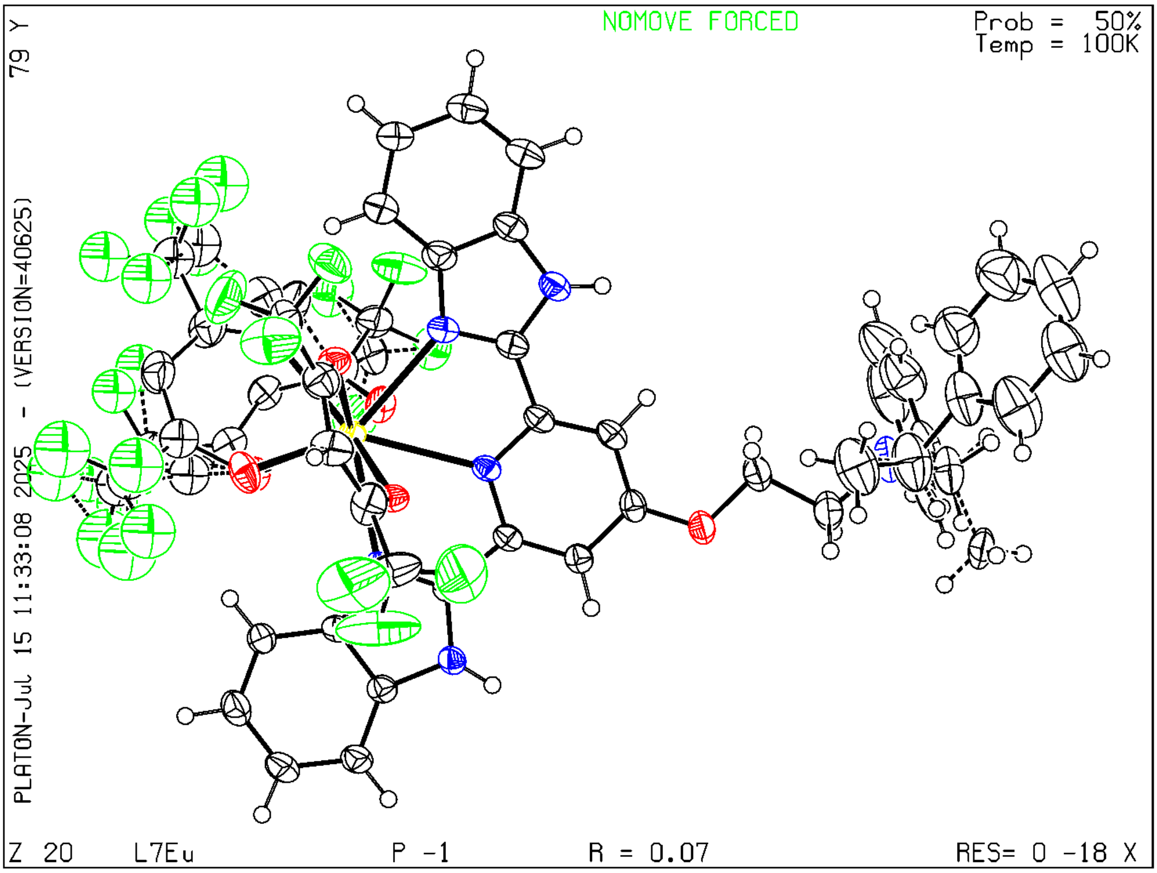

Datablock L8Eu - ellipsoid plot

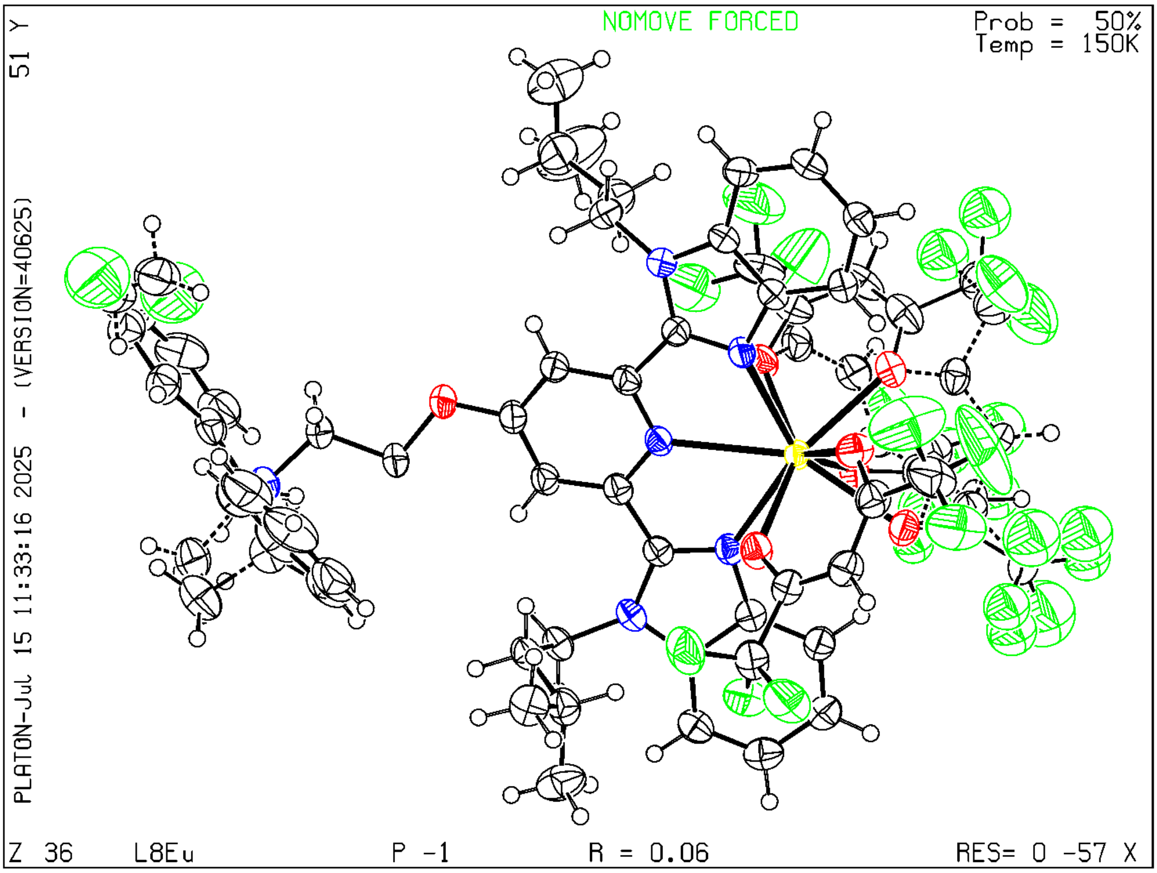

Supplement: Supplementary file 2 — Supporting Information [file CHEM-31-e02338-s002.zip › checkcif_complexes.pdf]
